# Unraveling the Role of *Scutellaria baicalensis* for the Treatment of Breast Cancer Using Network Pharmacology, Molecular Docking, and Molecular Dynamics Simulation

**DOI:** 10.3390/ijms24043594

**Published:** 2023-02-10

**Authors:** Yanqi Jiao, Chengcheng Shi, Yao Sun

**Affiliations:** 1 School of Science, Harbin Institute of Technology (Shenzhen), Shenzhen 518055, China; 2 State Key Lab of Urban Water Resource and Environment, School of Science, Harbin Institute of Technology (Shenzhen), Shenzhen 518055, China

**Keywords:** *Scutellaria baicalensis*, breast cancer, network pharmacology, molecular docking, molecular dynamics simulation

## Abstract

*Scutellaria baicalensis* is often used to treat breast cancer, but the molecular mechanism behind the action is unclear. In this study, network pharmacology, molecular docking, and molecular dynamics simulation are combined to reveal the most active compound in *Scutellaria baicalensis* and to explore the interaction between the compound molecule and the target protein in the treatment of breast cancer. In total, 25 active compounds and 91 targets were screened out, mainly enriched in lipids in atherosclerosis, the AGE–RAGE signal pathway of diabetes complications, human cytomegalovirus infection, Kaposi-sarcoma-associated herpesvirus infection, the IL-17 signaling pathway, small-cell lung cancer, measles, proteoglycans in cancer, human immunodeficiency virus 1 infection, and hepatitis B. Molecular docking shows that the two most active compounds, i.e., stigmasterol and coptisine, could bind well to the target AKT1. According to the MD simulations, the coptisine–AKT1 complex shows higher conformational stability and lower interaction energy than the stigmasterol–AKT1 complex. On the one hand, our study demonstrates that Scutellaria baicalensis has the characteristics of multicomponent and multitarget synergistic effects in the treatment of breast cancer. On the other hand, we suggest that the best effective compound is coptisine targeting AKT1, which can provide a theoretical basis for the further study of the drug-like active compounds and offer molecular mechanisms behind their roles in the treatment of breast cancer.

## 1. Introduction

Cancer is a group of diseases with rapid pathological proliferation and growth, leading to the uncontrolled division of abnormal cells in the body. The angiogenesis of these cells helps them invade surrounding body cells or tissues [1]. Breast cancer is associated with several cancer-related deaths in women and contributes to high mortality worldwide [2,3]. In fact, breast cancer is a heterogeneous disease involving multiple gene mutations and epigenetic changes in the breast tissue [4]. It occurs when ducts become abnormal and begin to divide uncontrollably. These abnormal cells may spread to the lymph nodes, blood vessels, lungs, bones, liver, and brain and can invade nearby breast tissue [3,5].

There are three main types of breast cancer. The first type is hormone-receptor-positive breast cancer [6], which is the most common type and accounts for approximately 70% of the existing breast cancer cases. Since the growth of hormone-receptor-positive breast cancer is inseparable from hormones, drugs that inhibit estrogen activity in the body can usually inhibit cancer growth. The most commonly adopted drugs are antiestrogens (such as tamoxifen) or aromatase inhibitors [7]. The second type is called HER2-positive breast cancer, which accounts for about 20% of breast cancer cases in which the human epidermal growth factor receptor 2 (HER2) oncogene protein is overexpressed. Several new targeted drugs have been specifically used to effectively treat HER2-positive breast cancer in the past decade, including the famous HER2-targeted drug trastuzumab (Herceptin) and the second-generation HER2-targeted drug pertuzumab. The third is triple-negative breast cancer (TNBC), which is the least common type, accounting for only about 10% of all breast cancer cases. This type is negative for hormone receptors and HER2, and thus, both endocrine therapy and HER2-targeted therapy are ineffective for its treatment. Generally, only chemotherapy drugs can be used [6,7,8].

Compared to the normally used radiotherapy, surgery, and chemotherapy, treatment using drugs derived from herbal sources is cost-effective, less toxic, and easily available [9]. In fact, plants have long been used in folk medicine to treat different types of cancer. Compounds from medicinal plants account for more than 70% of the cancer drugs currently in use [10]. An excellent example is traditional Chinese medicine (TCM), which has developed from plants used in folk medicine. TCM is a favorable resource for studying the anticancer effects of bioactive compounds in plants known for their long history of therapeutic applications [11,12]. *Scutellaria baicalensis* is a perennial plant belonging to the Lamiaceae family, and its dried root is an important TCM [13,14,15]. It has the functions of reducing heat and dampness, purging fire, and detoxifying, and its roots have been used in China for more than two thousand years to treat jaundice, hepatitis, diarrhea, and respiratory and gastrointestinal infections [16,17]. In the past few decades, the clinical applications of *Scutellaria baicalensis* have been extended to treat diseases such as inflammation, hypertension, cardiovascular disease, and tumors [18,19] based on its antipyretic, anti-inflammatory, antibacterial, and antitumor effects [20,21]. At the present stage, more than 126 small-molecule compounds and six polysaccharides have been isolated from *Scutellaria baicalensis*. The small-molecule compounds are usually divided into four different structural types including free flavonoids, flavonoid glycosides, phenylethanoid glycosides, and other small molecules [22].

Choi et al. reported that baicalein from *Scutellaria baicalensis* significantly inhibited cell migration and invasion by inhibiting the activities of MMP-2 and MMP-9 (via the AKT1 pathway) [23], which showed great future prospects for the treatment of breast cancer. Shang et al. found that baicalein could suppress 17-β-estradiol-induced migration, adhesion, and invasion of breast cancer cells via the G-protein-coupled receptor 30 signaling pathway [24]. Ma et al. demonstrated that baicalein could inhibit epithelial–mesenchymal transition (EMT) by downregulating SATB1 and the Wnt/β-catenin pathway and further inhibited the metastasis of MDA-MB-231 breast cancer cells in vivo [25]. Moreover, Zhang and co-workers reported that baicalin from *Scutellaria baicalensis* could inhibit the anti-apoptotic potential of breast cancer cells through in vitro and endogenous pathways, increase the expression of p38 and MAPK, and inhibit the activation of nf-κb-mediated anti-apoptotic protein (Bcl2) [26]. Gao et al. demonstrated that baicalin showed the ability to reduce inflammatory injury, inhibit tumor necrosis factor (TNF)-alpha and beta secretion of interleukin (IL)-1, and inhibit I ĸ kinase B nuclear factor (NF)-ĸB–p65 activation [27]. In addition, baicalin was found to inhibit the activity, migration, and invasion of BC cells and to promote apoptosis by regulating miR-338-3p and MORC4, showing a good pharmacological value in the treatment of breast cancer [28]. Liu et al. revealed that baicalin might have a potential therapeutic effect on breast cancer metastasis by regulating TGF-β1-dependent EMT progression [29]. Yang and co-workers revealed that baicalin could increase the expression of E-cadherin mRNA and decrease the expression of vimentin, β-catenin, c-Myc, and MMP-7 mRNA in LPS-induced MDA-MB-231 cells [30].

**Figure 1:**
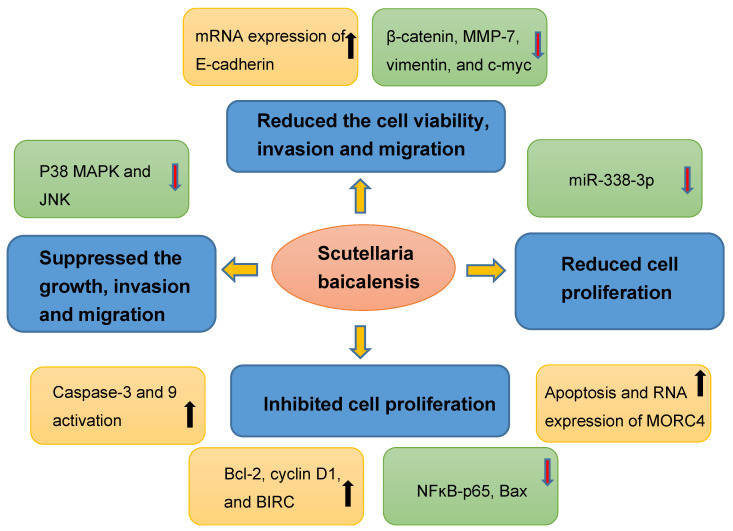
Some anticancer activities associated with *Scutellaria baicalensis* in the literature. The arrows in the figure indicate specific directional effects of *Scutellaria baicalensis* against breast cancer.

In addition to the overwhelming published work regarding the effects of baicalein and baicalin on breast cancer, AmeliMojarad and co-workers demonstrated that stigmasterol could inhibit breast tumor growth by inducing apoptosis in a Balb/c mouse model [31]. Stigmasterol is a natural phytosterol compound that also exists in *Scutellaria baicalensis* and has been proven capable of reducing cholesterol and inducing anti-inflammatory as well as anticancer properties. Research has shown that phytosterols tend to be beneficial for breast cancer treatment. However, to date, research on the association of phytosterols and cancer has been limited to certain breast cancer cell lines and animals. Sirirat et al. once estimated the correlation between the intake of phytosterol and the incidence of breast cancer by adopting data from 52,734 females from North America and found that it was uncertain to set up the inverse relationship between the intake of phytosterol and breast cancer incidence. This uncertainty was possibly due to a lack of statistical power or measurement error [32]. Coptisine is another small-molecule compound present in *Scutellaria baicalensis* that has been used for thousands of years. Wu and co-workers found that coptisine was potentially a compound showing anticancer, anti-inflammatory, and antibacterial effects through regulation of the signaling transduction of pathways including NF-κB, MAPK, PI3K/Akt, NLRP3 inflammasome, RANKL/RANK, and Beclin 1/Sirt1 [33]. Furthermore, Li et al. experimentally showed that coptisine could suppress the adhesion, migration, and invasion of MDA-MB-231 breast cancer cells in vitro, downregulating MMP-9 in combination with an increase in TIMP-1, which possibly contributed to the antimetastatic function [34]. There are also published studies revealing other compounds such as wogonin, wogonoside, β-sitosterol, and norwogonin that exist in *Scutellaria baicalensis* as active compounds for the treatment of breast cancer [35,36,37,38]. Although *Scutellaria baicalensis* is believed to be promising to treat breast cancer, the systematic investigations of the active compounds in *Scutellaria baicalensis* and the molecular mechanisms behind their actions remain to be undertaken. Figure 1 summarizes some of the anticancer activities associated with *Scutellaria baicalensis* in the literature.

Network pharmacology is a method used to predict the active compounds and the disease targets at the system level and to establish a multilevel network such as drug–ingredient–target–disease [39]. This bioinformatics method can be used to identify key targets related to drugs and diseases. Molecular docking is a technique that uses flexible and semiflexible models to evaluate the binding surface and interaction force between the receptor and ligand and, hence, for predicting the binding mode and affinity of the receptor–ligand complex [40]. Molecular dynamics simulation allows for the estimation of the structural stability of the receptor and ligand, as well as the dynamics of receptor–ligand interaction based on a proper forcefield [41]. In this study, network pharmacology, molecular docking, and molecular dynamics simulation were combined to systematically explore the biological pathways related to the treatment of breast cancer using *Scutellaria baicalensis*.

## 2. Results

### 2.1. Active Compounds in Scutellaria baicalensis and Their Targets

A total of 143 compounds were detected and screened by the TCMSP database, of which 36 compounds met the OB ≥ 30% and DL ≥ 0.18 standards. In addition, another two compounds were detected and screened by the Batman database. Then, the 38 compounds were input into the SwissTargetPrediction database to predict the protein targets of all active compounds. We also added some metabolites that had been identified as the most potent compounds for the treatment of breast cancer from 3-(cystein-S-yl) acetaminophen (xenobiotics pathway), 4-acetylphenol sulfate (xenobiotics pathway), and cysteine s-sulfate (amino acid pathway) in the literature [42], even though they did not meet the OB and DL standards. These compounds were also put into the SwissTargetPrediction database to predict the targets of all active compounds. Then, all the protein targets were input into the UniProt database for normalization with repeated targets removed. Finally, a total of 95 gene targets were obtained.

### 2.2. Effective Targets for Breast Cancer

**Figure 2:**
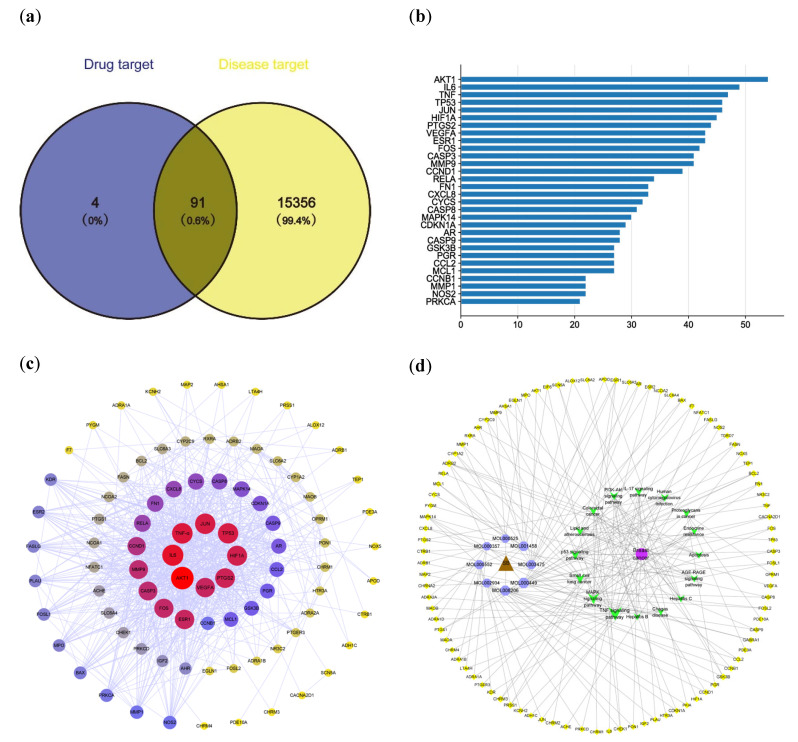
Potential targets of *Scutellaria baicalensis* against breast cancer and PPI network. (**a**) Venn diagram of potential gene targets. (**b**) The top 30 breast cancer targets by degree. (**c**) The PPI network of *Scutellaria baicalensis* for the treatment of breast cancer. (**d**) The compounds–targets–pathways network showing potential mechanism of *Scutellaria baicalensis* for the treatment of breast cancer.

A total of 15,606 and 878 targets were obtained from the GeneCards and OMIM databases, respectively. After deleting duplicates, 15,447 related targets were kept. The 15,447 breast-cancer-related targets and the 95 *Scutellaria baicalensis* gene targets were mapped to each other using the online tool Venny 2.1.0 software (https://bioinfogp.cnb.csic.es/tools/venny/index.html), and 91 *Scutellaria baicalensis*–breast cancer intersections were obtained (Figure 2a). All of the intersection targets were located between differentially expressed genes in the breast cancer dataset.

### 2.3. PPI Network Analysis

The 91 predicted targets were imported into STRING for PPI network analysis. The PPI network complex consisted of 60 nodes and 394 edges (Figure 2c). The node could be designed as a hub node if the degree, betweenness, and closeness satisfied specific criteria, such as the median of the corresponding parameters. The network centrality was used to define the network properties (degree centrality, betweenness centrality (BC), and closeness centrality (CC)) of the compounds separately or collectively and to judge the importance of nodes. Nodes with higher ranks (larger size) were considered to have a more critical role within the network. The node ranking of the main active compounds in *Scutellaria baicalensis* is summarized in Table 1, including stigmasterol, moslosooflavone, coptisine, 5,2′-dihydroxy-6,7,8-trimethoxyflavone, 9-cedranone, neobaicalein, norwogonin, sitogluside, salvigenin, and beta-sitosterol. The top 10 targets were AKT1 (RAC-α serine/threonine protein kinase, degree = 54), IL6 (interleukin-6, degree = 49), TNF (tumor necrosis factor, degree = 47), TP53 (tumor protein p53, degree = 46), JUN (transcription factor Jun, degree = 46), HIF1A (hypoxia inducible factor 1 subunit alpha, degree = 45), PTGS2 (interleukin-6, degree = 44), VEGFA (vascular endothelial growth factor A, degree = 43), ESR1 (estrogen receptor 1, degree = 43), and FOS (fos proto-oncogene, AP-1 transcription factor subunit, degree = 42) (Figure 2b). To explain the mechanism of *Scutellaria baicalensis* against breast cancer, a compound–target pathway network was constructed based on the above compounds, targets, and pathway information, as shown in Figure 2d. The yellow circles, light blue circles, green V-shape, brown triangle, and purple circle represent the target proteins, active compounds, potential pathways, *Scutellaria baicalensis*, and breast cancer involved in the process of *Scutellaria baicalensis* against breast cancer, respectively.

### 2.4. GO and KEGG Pathway Enrichment Analysis

Gene ontology (GO) analysis can be used for the essential annotation of gene products. Biological process (BP) enrichment showed the coupling effect and transport mode of proteins in biological pathways. The CC analysis showed that cross-proteins were involved in the cellular environment. Using molecular function (MF) analysis, the activity of certain protein receptors regulated by drugs could be demonstrated.

**Figure 3:**
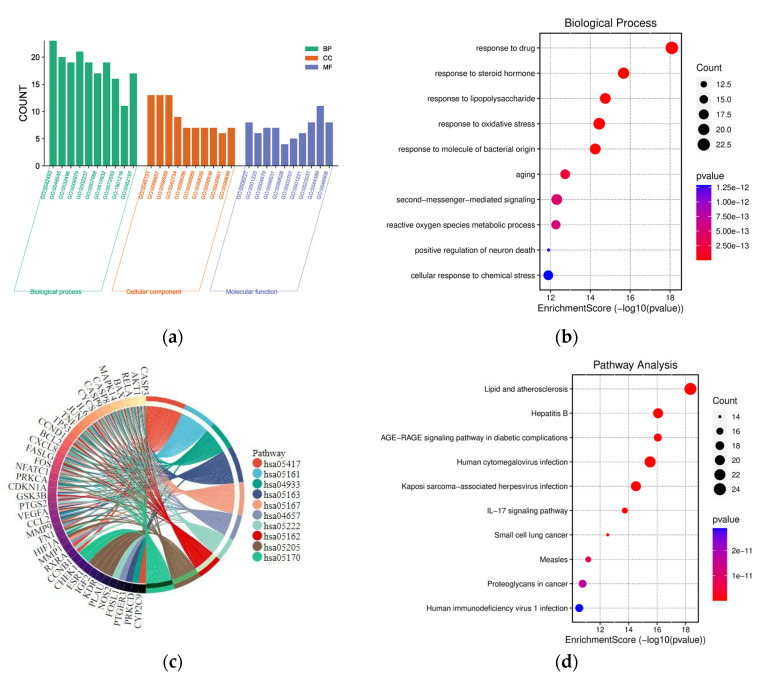
GO function and KEGG pathway enrichment analyses of *Scutellaria baicalensis* in the treatment of breast cancer. (**a**) GO functional analysis, including BP, CC, and MF. (**b**) Bubble diagram of BP enrichment. (**c**) Gene ontology of the top 10 pathways in *Scutellaria baicalensis* against breast cancer and (**d**) bubble diagram of KEGG pathway enrichment.

The 91 *Scutellaria baicalensis*–breast cancer intersections were imported into the Metascape platform, and GO functional enrichment analysis was carried out on the targets of active compounds in the treatment of breast cancer from the levels of BP, CC, and MF (Figure 3a). There were 3348 BP items (Table S1 in the Supplementary Materials), and the top 10 items were selected for visual analysis (Figure 3b). The size of the circle indicated the count of the targets, and the color scale (blue to red) represented the size of the log *p*-value of the BP item. It was found that the BP was related to drug response, steroid hormones, lipopolysaccharide, oxidative stress, bacterial-derived molecules, aging, second-messenger-mediated signaling, reactive oxygen species metabolism processes, and the neuronal death cell positive regulation of the chemical stress response. There were 221 items in the CC analysis (Table S2 in the Supplementary Materials), and the top 10 items were selected for visual analysis (Figure S1 in the Supplementary Materials), including the membrane raft, membrane microdomain, membrane region, and presynaptic membrane components. In addition, there were a total of 392 items in the MF analysis (Table S3 in the Supplementary Materials), and the top 10 entries were selected for visualization analysis, which were related to G-protein-coupled receptor activity, transcriptional binding, the auxiliary activation of nuclear receptor activity, ligand-activated transcription factor activity, the G-protein-coupling activity of neurotransmitter receptors, steroid hormone receptor activity, auxiliary transcription factor binding, heme binding, ubiquitin-like protein ligase binding, and tetrapyrrole binding (Figure S2 in the Supplementary Materials). According to the log *p*-values in Figure 3d, 10 signaling pathways with high probability were screened out based on enrichment factor values and the number of genes involved in each pathway, which were closely related to the mechanism of the treatment of breast cancer. The size of the circle indicated the count of the targets, and the color scale (blue to red) represented the size of the log *p*-value of the pathway.

To analyze the significance and importance of key targets in the pathways involved in the treatment of breast cancer, the 10 top pathways determined according to gene counts and adjusted *p*-values from the Kyoto Encyclopedia of Genes and Genomes (KEGG) enrichment analysis and related targets were used to construct a KEGG key pathway network (Figure 3c). According to Figure 3c and Table S4 in the Supplementary Materials, the role of *Scutellaria baicalensis* in the treatment of breast cancer could be mainly related to lipids and atherosclerosis (Figure S3 in the Supplementary Materials), the AGE–RAGE signal pathway of diabetes complications, the IL-17 signaling pathway, Kaposi-sarcoma-associated herpesvirus infection, measles, human cytomegalovirus infection, proteoglycans in cancer, small-cell lung cancer, human immunodeficiency virus 1 infection, and hepatitis B. Therefore, *Scutellaria baicalensis* could target multiple functional and biological factors in the treatment of breast cancer. However, the effects and far-reaching impacts still need to be further verified.

### 2.5. Molecular Docking of Compound and Target

**Figure 4:**
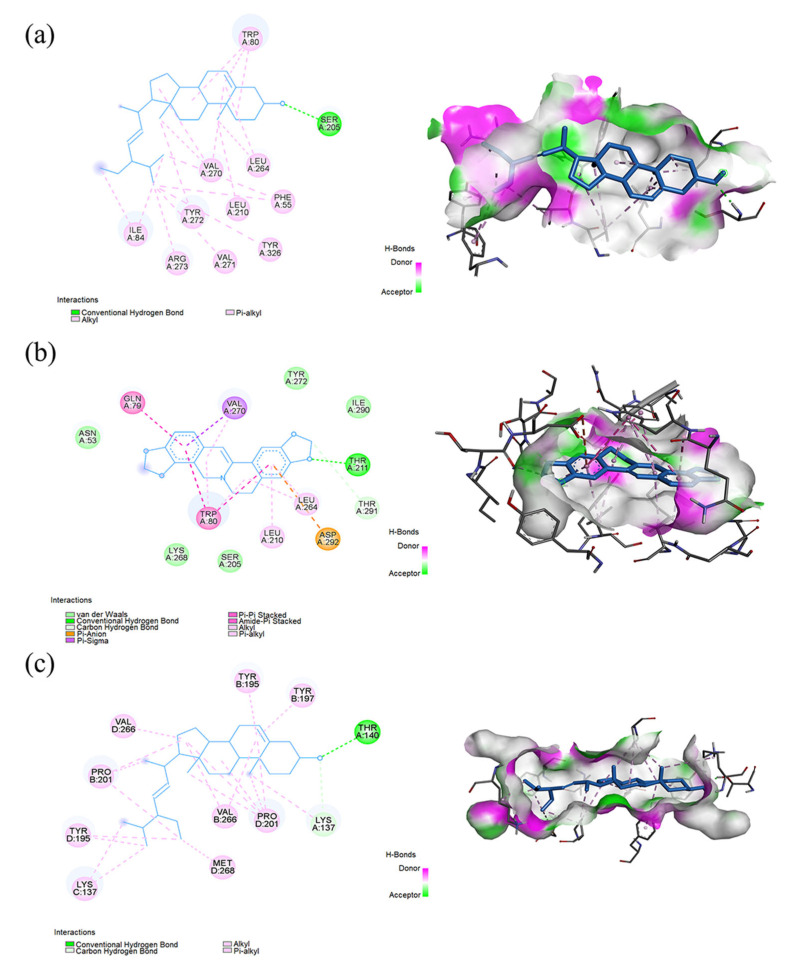
Molecular docking diagrams with 2D and 3D plots. The (**a**) stigmasterol–AKT1, (**b**) coptisine–AKT1, and (**c**) coptisine–PTGS2 complexes.

We performed the molecular docking according to the breast-cancer-related targets and selected compounds from the PPI network. The interactions between the potential active compounds and key targets were analyzed by using AutoDockTools-1.5.6, Discovery Studio 4.5 Client, and PyMOL software. The selected top five active compounds included 5,2′-dihydroxy-6,7,8-trimethoxyflavone (MOL000552), moslosooflavone (MOL008206), stigmasterol (MOL000449), coptisine (MOL001458), and 9-cedranone (MOL003475). The protein structures of key targets were acquired online from RCSB PDB, including AKT1 (PDB ID: 4EJN), IL6 (PDB ID: 4O9H), TNF (PDB ID: 2AZ5), TP53 (PDB ID: 7BWN), JUN (PDB ID: 5T01), HIF1A (PDB ID: 1H2K), PTGS2 (PDB ID: 5F19), VEGFA (PDB ID: 1VPF), ESR1 (PDB ID: 1A52), and FOS (PDB ID: 1A02). The binding scores were obtained from the docking analysis (Table 2). Notably, a lower value indicated a stronger binding ability. We found that the van der Waals force, hydrogen bonding, and aromatic stacking (Pi–Sigma, Pi–alkyl, and alkyl interactions) were involved between the active site residues of key targets and the potential active compounds. The binding scores of all the docking results were less than −6 kcal/mol, indicating the stable binding pattens between the active compounds and protein targets. The top three compound–target dockings with the lowest values of docking scores are visualized in Figure 4. The best three affinity modes (red marked) were coptisine–AKT1, stigmasterol–AKT1, and coptisine–PTGS2 (Table 2). The stigmasterol–AKT1 complex was stabilized by one hydrogen bond (1H-bond) with residue SER 205, 10 alkyls and Pi–alkyl interactions with TRP 80, LEU 264, VAL 270, PHE 55, LEU 210, TYR 272, TYR 326, VAL 271, ARG 273, and ILE 84, respectively (Figure 4a). Meanwhile, coptisine–AKT1 presented five van der Waals forces with ASN 53, LYS 268, SER 205, TYR 272, and ILE 290. In addition, the coptisine–AKT1 complex was stabilized by 1H-bond with residue THR 211, one Pi–Sigma bond with VAL 270, and two alkyls and Pi–alkyl interactions with LEU 210 and LEU 264 (Figure 4b). The coptisine–PTGS2 complex had four alkyl and Pi–alkyl interactions with CYS 47, CYS 36, PRO 153, and VAL 36, respectively. There was also one Pi–Sigma bond with LEU 152 (Figure 4c).

### 2.6. Structural Stability and Interaction Energy by Molecular Dynamics Simulation

**Figure 5:**
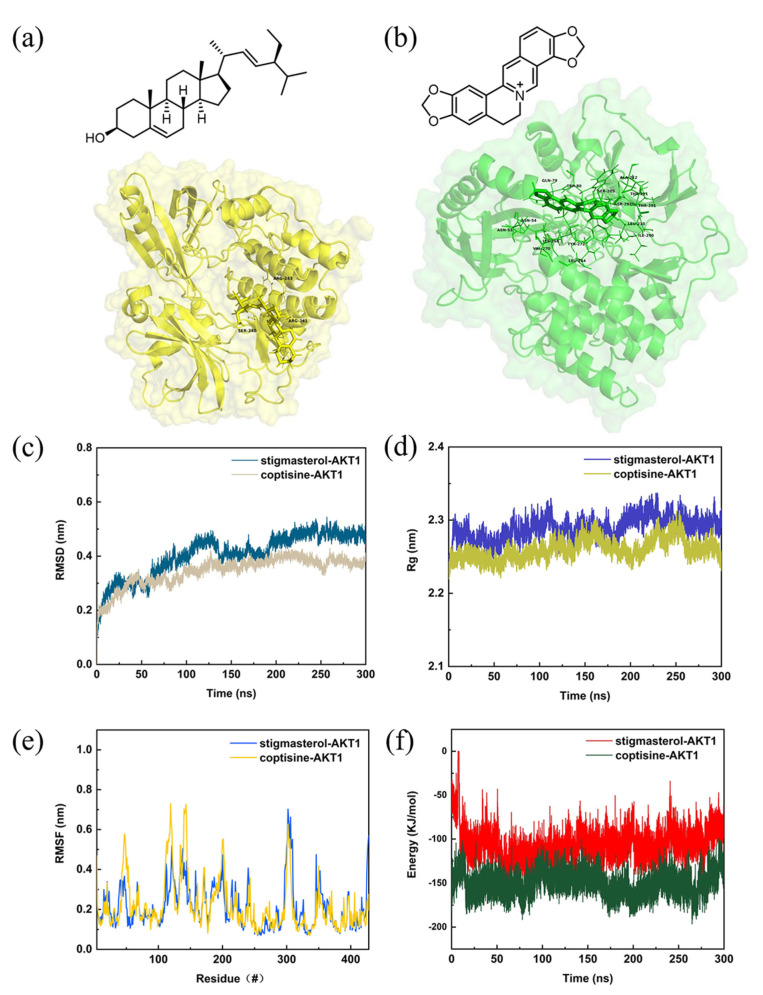
The results of molecular dynamics simulations. (**a**) The chemical structures of stigmasterol and coptisine and the conformations of the stigmasterol-AKT1 and coptisine-AKT1 complexes and their contact residues. The (**b**) coptisine-AKT1 complexes, (**c**) RMSD curves, (**d**) radius of rotation curves, (**e**) RMSF curves, and (**f**) interaction energy curves for the stigmasterol-AKT1 and coptisine-AKT1 complexes during the 300 ns simulations.

**Figure 6:**
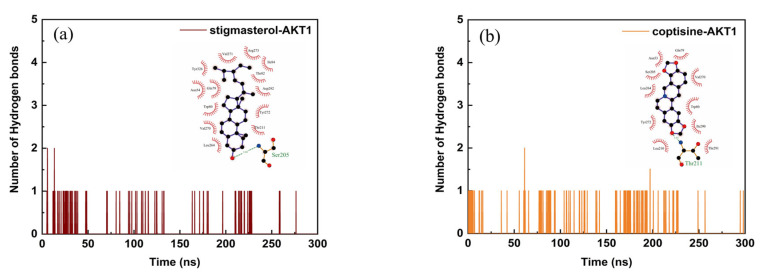
Number of hydrogen bonds between the (**a**) stigmasterol–AKT1 and (**b**) coptisine–AKT1 complexes with 2D interaction diagrams plotted inside the figures.

We selected the top two compound–target dockings (coptisine–AKT1 and stigmasterol–AKT1) to conduct the molecular dynamics simulations. After 300 ns of MD simulations, the dynamic evolutions of the stigmasterol–AKT1 and coptisine–AKT1 complexes could be analyzed. The chemical structures of stigmasterol and coptisine and the conformations of the stigmasterol–AKT1 and coptisine–AKT1 complexes and their contact residues are shown in Figure 5a,b. The root-mean-square deviation (RMSD) curve represents positional deviations in the protein. As can be seen from Figure 5c, the RMSD curves reached relatively stable stages after 250 ns, and the RMSD of the coptisine–AKT1 complex was smaller than that of the stigmasterol–AKT1 complex. The radius of the rotation curve indicates the tightness of the overall structure of the protein. As can be seen from Figure 5d, both the stigmasterol–AKT1 and coptisine–AKT1 complexes had stable rotation radii, and the coptisine–AKT1 complex was more tightly folded than the stigmasterol-AKT1 complex. The root-mean-square fluctuation (RMSF) curve represents the fluctuations of amino acid residues in the protein. In Figure 5e, the RMSF values of residue numbers 50–200 in the AKT1 upon binding of coptisine showed larger flexibility than the same regions in AKT1 bound with stigmasterol. In addition, the interaction energy curves for the stigmasterol–AKT1 and coptisine–AKT1 complexes were calculated (Figure 5f), from which the average interaction energy of coptisine–AKT1 was −151.794 kcal/mol with an energy drift of 0.61 kcal/mol, lower than that of the stigmasterol–AKT1 complex (−108.116 kcal/mol with an energy drift of 6.32 kcal/mol). From Figure 6, it can be seen that the interaction binding sites of the stigmasterol–AKT1 and coptisine–AKT1 formed hydrophilic environments with strong hydrophilicity, while the hydrogen and π bonds formed could help to maintain their stabilities. Moreover, more hydrogen bonds formed between the coptisine and AKT1 than between the stigmasterol and AKT1 during the 300 ns simulations.

## 3. Discussion

*Scutellaria baicalensis* is one of the most influential plants that attracts attention in cancer chemotherapy studies [43]. Despite the fact that there are in vitro and in vivo animal studies and several clinical case studies of *Scutellaria baicalensis* in cancer treatment, integrated and systematic studies using a computational pharmacological approach, together with molecular dynamics and docking methods to explore the effective substances, putative targets, and potential pharmacological mechanism of *Scutellaria baicalensis* in the treatment of breast cancer remain deficient. In addition, the multiple targets and pathways of its antitumor effects are quite unclear. Therefore, we adopted network pharmacology to investigate the potential pharmacological and molecular mechanism of *Scutellaria baicalensis* against breast cancer. A total of 91 potential targets associated with breast cancer were identified. Many targets were found to be hit by multiple compounds. The results suggest that the bioactive compounds of *Scutellaria baicalensis* may modulate multiple targets and synergistically affect these targets. Therefore, the active compounds of *Scutellaria baicalensis* have therapeutic effects not only on breast cancer but also on other diseases, which can be confirmed by the multicomponent, multitarget, and multidisease nature of the plant medicine.

The PPI analysis of the 91 targets shows that the top 10 central targets, including AKT1, IL6, TNF, TP53, JUN, HIF1A, PTGS2, VEGFA, ESR1, and FOS, may be the key targets of the treatment of breast cancer. We selected the top five bioactive compounds, namely, stigmasterol, moslosooflavone, coptisine, 5,2′-dihydroxy-6,7,8-trimethoxyflavone, and 9-cedranone, and the 10 key targets in this study. The results of molecular docking showed that these bioactive compounds could effectively bind to the 10 target proteins. To better understand the multiple effects of *Scutellaria baicalensis* against breast cancer from a systematic perspective, we further performed GO enrichment analysis on 91 selected targets. The top 10 GO functional categories indicate that *Scutellaria baicalensis* may exert its effects by participating in the BP, MF, and CC. The target genes enriched for BP are mainly concentrated in response to various phosphorylations [44]. Protein phosphorylation is an important cellular regulatory mechanism through which many enzymes and receptors are activated or deactivated. It plays a key role in controlling BPs such as proliferation, differentiation, and apoptosis [44]. MF is closely associated with different kinase activities, including transmembrane receptor protein tyrosine kinase activity, protein kinase activity, and protein tyrosine kinase activity. Functional enrichment analysis suggests that *Scutellaria baicalensis* plays an anti–breast cancer role in regulating transcription such as different kinase activities and, therefore, leads to phosphorylation changes in the cell signaling pathways.

The KEGG pathway enrichment analysis shows that 91 target proteins are significantly enriched in 232 related signaling pathways (Table S4 in the Supplementary Materials). Considering the results of these well-known cancer-related pathways, *Scutellaria baicalensis* can target multiple pathways simultaneously. Among the 232 signaling pathways obtained, lipid and atherosclerosis (HSA05417) is the most critical one in regulating the genetic stability, proliferation, and apoptosis of breast cancer cells. In addition, we found that *Scutellaria baicalensis* may play a therapeutic role in breast cancer through a variety of other signaling pathways. For example, we identified the FoxO pathway (HSA04068) toward breast cancer, which was also reported in the literature to be involved in cell cycle regulation, the proliferation process, and apoptosis in human breast cancer [45]. In addition, we identified the PI3K/AKT pathway (HSA04151) as playing an important role in the development and progression of breast cancer, which was also reported by other researchers who found that the activation of PI3K/AKT signaling reduced apoptosis, stimulated cell growth, and increased proliferation [46]. As a well-known transcription factor and tumor suppressor, we identified the tumor protein p53 (HSA04115) that was reported to regulate the expression of a variety of genes involved in apoptosis, growth arrest, or senescence in response to genotoxicity or cellular stress [47]. These findings could support our KEGG pathway enrichment analysis.

The network pharmacology results were verified by the molecular docking of the top 10 targets and the five selected active compounds. The stigmasterol–AKT1 and coptisine–AKT1 complexes have the lowest binding scores (−11.1 kcal/mol). The results indicate that the two complexes may be the key targets and active compounds of *Scutellaria baicalensis* in treating breast cancer. It is worth noting that the binding scores of baicalein–AKT1 and baicalin–AKT1 were reported to be approximately −6.5 kcal/mol and −6.1 kcal/mol, both of which were higher than that of stigmasterol–AKT1 and coptisine–AKT1 [48,49,50]. These findings demonstrate that stigmasterol and coptisine possess better binding affinities to AKT1 than the experimentally investigated baicalin and baicalein. In addition, the results of MD simulations show that the binding of coptisine–AKT1 is more stable than that of stigmasterol–AKT1 since the average interaction energy of the coptisine–AKT1 was −151.794 kcal/mol (energy drift: 0.61 kcal/mol), lower than the −108.116 kcal/mol of the stigmasterol–AKT1 complex (energy drift: 6.32 kcal/mol). The underlying mechanism of *Scutellaria baicalensis* has been revealed by network pharmacological and molecular analyses in this study. However, our work could not determine the optimal dose to induce a response with low toxicity. Therefore, further animal and cell models are needed to validate the relevant pathways and targets.

## 4. Materials and Methods

### 4.1. Screening of Active Compounds and Gene Targets of Scutellaria baicalensis

The TCMSP platform (http://tcmspw.com/tcmsp.php) was used to search for the drug compounds of *Scutellaria baicalensis*. According to the absorption of exogenous chemicals (ADMET) by the pharmacokinetic body, compounds with oral availability (OB) ≥30% and drug-likeness (DL) ≥0.18 were screened out, and then, the targets of the effective compounds were searched for via their MOL.ID. At the same time, we used the BATMAN and PubChem databases (https://pubchem.ncbi.nlm.nih.gov) to obtain the SMILES (Simplified Molecular Input Line Entry System) of the compounds and then imported them into the structural similarity forecast target database SwissTargetPrediction (http://www.swisstargetprediction.ch) to predict the targets. The Excel data of the compounds were downloaded from the UniProt (http://www.uniprot.org/) database and optimized with the TRIM function, while the target gene name was matched with the VLOOKU function. Unmatched gene names were supplemented by a literature review. Finally, the related target proteins obtained by the above methods were annotated using UniProt (https://www.uniprot.org).

### 4.2. Constructing a “Compound–Target” Network

The “network” file and Type file of the gene targets were prepared, and the related files were imported into the system using Cytoscape 3.7.2 software to perform network topology analysis. The graph, color, transparency, and size of the target points were adjusted according to the Degree (the number of gene connections) to construct the “TCM compound–target” network map.

### 4.3. Predicting the Disease Targets

The GeneCards (https://www.genecards.org/), OMIM (https://www.omim.org/), and DrugBank (https://go.drugbank.com/) platforms were used for disease-related targets, and the disease name was set as “*Scutellaria baicalensis*” to search for breast cancer disease-related targets. The object and function were set as “human” and “VLOOKUP” to match the target gene name and screen the intersection genes of the drugs and the disease.

### 4.4. Acquitting the Intersection Targets

The Venny (https://bioinfogp.cnb.csic.es/tools/venny/) software was used to obtain the intersection targets of the active compounds from TCM and breast cancer disease targets, which could act as potential targets for the treatment of breast cancer.

### 4.5. PPI Network Construction and Cluster Analysis

The intersection gene was imported into the String (https://string-db.org/) platform. The protein interaction relationship could be obtained by setting the object, highest confidence, and the free gene node as homo sapiens, 0.900, and hidden, respectively. The results were then imported into Cytoscape 3.7.2, and the network analyzer was selected to obtain the network topology parameters. The BC and CC represented the shortest path crossing a single node and the ease of communication between nodes. A value greater than twice the median of Degree and the median of the BC and CC was used as the criterion to screen the core compound targets of *Scutellaria baicalensis* and disease and to create a PPI network interaction map [51].

### 4.6. GO and KEGG Analysis

The GO function and KEGG pathway enrichment analysis were performed on the Metascape (http://metascape.org/) platform. GO was a major bioinformatics tool for annotating genes and analyzing the BP of these genes [52]. KEGG was used to understand high-level functional and biological systems from large-scale molecular datasets generated by high-throughput experimental techniques [53]. The obtained intersection targets were imported into the gene list; the object was set as “Human” and personalized analysis was selected, and Min Overlap, *p*-value, and the minimum concentration were set as 3, 0.05, and 1.5, respectively. After the results were obtained, the GO bubble map and KEGG pathway map were made. R software was used for the GO and KEGG enrichment analyses of the target sites. Key targets were imported into R software, and clusterProfiler was used for the enrichment analysis. In order to analyze the function of medicinal materials, *p* < 0.05 was considered statistically significant.

### 4.7. Molecular Docking

We used the AutoDockTools 1.5.6, Discovery Studio 4.5 Client, and PyMOL software to reveal the interaction between active compounds and target proteins. The 2D structures of the compounds were downloaded from the PubChem database and converted into 3D structures with minimal energy using Chem3D software. The 3D structures of the proteins were downloaded from the Protein Database (PDB, http://www.rcsb.org/). The PyMOL software was used to dehydrate and remove ligand residues from proteins. The AutoDockTools 1.5.6 software was used to hydrogenate the receptor protein and save it into the PDBQT format. The compounds were also stored in the PDBQT format. The active pocket sites were established over the entire protein. Finally, AutoDock Vina was used to dock and identify the best construct. The active site of the protein was concentrated on the site of the active amino acid of the original ligand in the crystal structure. The residue information was available from the literature [54].

### 4.8. Molecular Dynamics Simulation

The particle mesh Ewald (PME) method was used to calculate the long-range electrostatic interactions. The simulation box was filled with TIP3P. The protein was placed at the center of the box, 1.2 nm away from the box boundary. Thirty-three Na^+^ ions were introduced into the water box to neutralize the charge of the entire system. The energy minimization and equilibration processes were performed in three steps: (i) We used the steepest descent algorithm to minimize the entire system including ions, solvents, and proteins by up to 100,000 steps. (ii) We balanced targets and compounds to 310 K (NVT equilibration, 100 ps, v-rescale (velocity rescale method)) with backbone restrained. (iii) We further ran the NPT equilibration at a constant pressure (1 bar) and temperature (310 K). Finally, a 300 ns molecular dynamics test was performed after all constraints were removed. The results were analyzed by the Gromacs built-in tools and our internal scripts.

## 5. Conclusions

Combining bioinformatics, molecular docking, and molecular dynamics techniques, our work revealed that *Scutellaria baicalensis* is effective in the treatment of breast cancer with the characteristics of multicomponent, multitarget, and multipathway properties from a systematic perspective. We identified the most effective target protein, AKT1, and two effective compounds, i.e., stigmasterol and coptisine, that bind well to AKT1. Using molecular dynamics, we further determined that coptisine and AKT1 are the best compound and target due to their low binding energy and high structural stability. Our study provides a theoretical basis for the further utilization of *Scutellaria baicalensis* in the treatment of breast cancer and, hopefully, can guide more advanced experimental research in the future.

## Figures and Tables

**Table 1:** Node ranking of the main active compounds.

ID	Name	Degree
MOL000449	stigmasterol	12
MOL008206	moslosooflavone	11
MOL001458	coptisine	10
MOL000552	5, 2′-dihydroxy-6, 7, 8-trimethoxyflavone	9
MOL003475	9-cedranone	9
MOL002934	neobaicalein	8
MOL000525	norwogonin	7
MOL000357	sitogluside	7
MOL002915	salvigenin	6
MOL000358	beta-sitosterol	6
MOL000073	ent-epicatechin	6
MOL002917	5, 2′, 6′-trihydroxy-7, 8-dimethoxyflavone	5
MOL002927	skullcapflavone II	5
MOL002897	epiberberine	5
MOL002933	5, 7, 4′-trihydroxy-8-methoxyflavone	4
MOL002937	dihydrooroxylin	4
MOL000007	cosmetin	3
MOL002925	5, 7, 2′, 6′-tetrahydroxyflavone	2
MOL002928	oroxylin a	2
MOL002932	panicolin	2
MOL000359	sitosterol	2
MOL001490	bis[(2S)-2-ethylhexyl] benzene-1, 2-dicarboxylate	2
MOL002879	diop	2
MOL010415	11, 13-eicosadienoic acid, methyl ester	2
MOL000458	campesterol	2

**Table 2:** The docking scores (kcal/mol) of the active compounds and key targets. The red color has the stronger binding ability than the green color, and the darker color indicates the stronger binding ability than the light color.

ID	MOL000552	MOL008206	MOL000449	MOL001458	MOL003475
Target	5, 2′-Dihydroxy-6, 7, 8-trimethoxy	Moslosooflavone	Stigmasterol	Coptisine	9-Cedranone
AKT1	−9.1	−9.5	−11.1	−11.1	−7.9
IL6	−7.8	−7.1	−7.5	−8.4	−6.6
TNF	−7.7	−7.8	−8.9	−9.3	−7.4
TP53	−8	−8	−9.7	−9.1	−7.5
JUN	−7.1	−6.9	−7.9	−9.6	−6.2
HIF1A	−6.8	−6.8	−7.6	−7.9	−6.5
PTGS2	−8.5	−9	−8.6	−11	−7.1
VEGFA	−6.7	−6.7	−6.9	−7.8	−5.8
ESR1	−8.1	−8	−7.5	−7.7	−8
FOS	−7.6	−7.5	−8	−8.2	−6.1

## Data Availability

The protein structure of key targets were acquired online from RCSB Protein Data Bank (https://www.rcsb.org/), including AKT1 (PDB ID: 4EJN), IL6 (PDB ID: 4O9H), TNF (PDB ID: 2AZ5), TP53 (PDB ID: 7BWN), JUN (PDB ID: 5T01), HIF1A (PDB ID: 1H2K), PTGS2 (PDB ID: 5F19), VEGFA (PDB ID: 1VPF), ESR1 (PDB ID: 1A52), and FOS (PDB ID: 1A02).

## Supplementary Materials

The following supporting information can be downloaded at: https://www.mdpi.com/article/10.3390/ijms24043594/s1, Figure S1: Bubble diagram of Cellular Component (CC) enrichment; Figure S2: Bubble diagram of Molecular Funtion (MF) enrichment; Figure S3: The Lipid and atherosclerosis (HSA05417) pathway by the KEGG mapper, the red represents target of Scutellaria baicalensis in the Lipid and atherosclerosis pathway; Table S1: Results of BP GO terms enrichment of Scutellaria baicalensis in breast cancer; Table S2: Results of CC GO terms enrichment of Scutellaria baicalensis in breast cancer; Table S3: Results of MF GO terms enrichment of Scutellaria baicalensis in breast cancer; Table S4: Results of KEGG terms enrichment of Scutellaria baicalensis in breast cancer.
