# Supplementary material for: Unraveling the Role of Scutellaria baicalensis for the Treatment of Breast Cancer Using Network Pharmacology, Molecular Docking, and Molecular Dynamics Simulation
Source: Int J Mol Sci. 2023 Feb 10;24(4):3594. doi: 10.3390/ijms24043594 (PMC9964558; doi:10.3390/ijms24043594)
Supplement: Supplementary file 1 [file ijms-24-03594-s001.zip › ijms-2128726-supplementary.pdf]

Supplementary Materials

Unraveling the role of Scutellaria baicalensis for the treatment of breast cancer using network pharmacology, molecular docking, and molecular dynamics simulation

Yanqi Jiao[1], Chengcheng Shi[2], Yao Sun[1,]*

[1]School of Science, Harbin Institute of Technology (Shenzhen), Shenzhen, Guangdong, 518055, China.

[2]State Key Lab of Urban Water Resource and Environment, School of Science, Harbin Institute of Technology (Shenzhen), Shenzhen, Guangdong, 518055, China.

[*]To whom correspondence should be addressed. Email: sunyao0819@hit.edu.cn (Dr. Yao Sun).

*) Corresponding author:

Dr. Yao Sun (sunyao0819@hit.edu.cn)

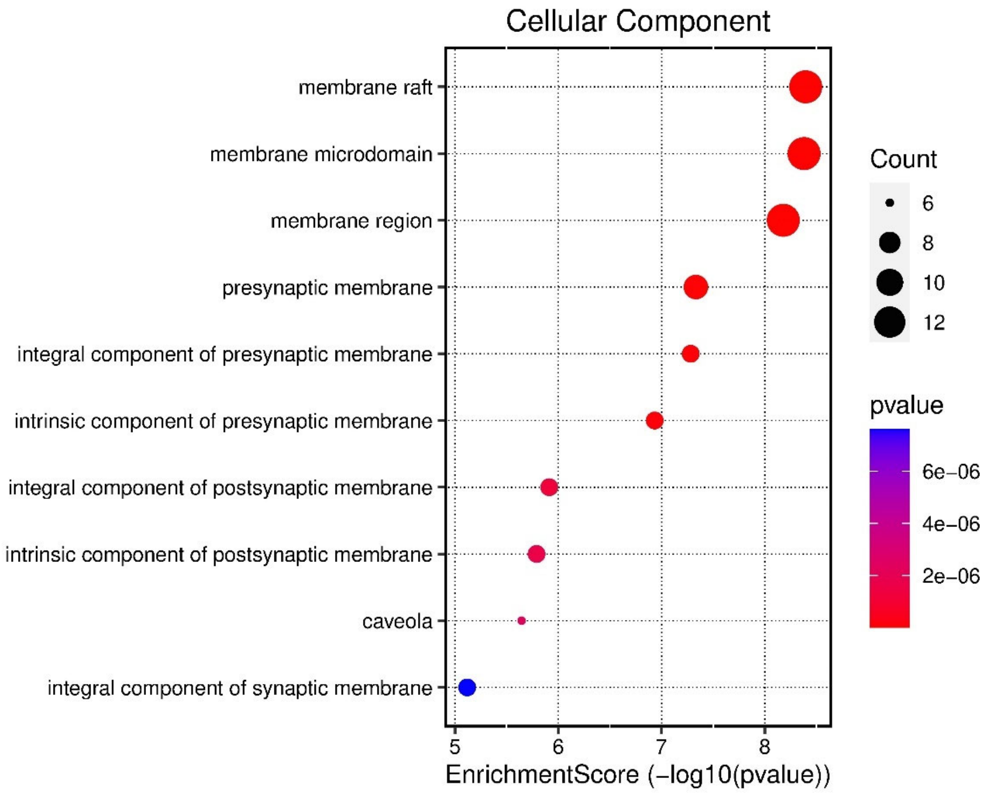

Figure S1 Bubble diagram of Cellular Component (CC) enrichment.

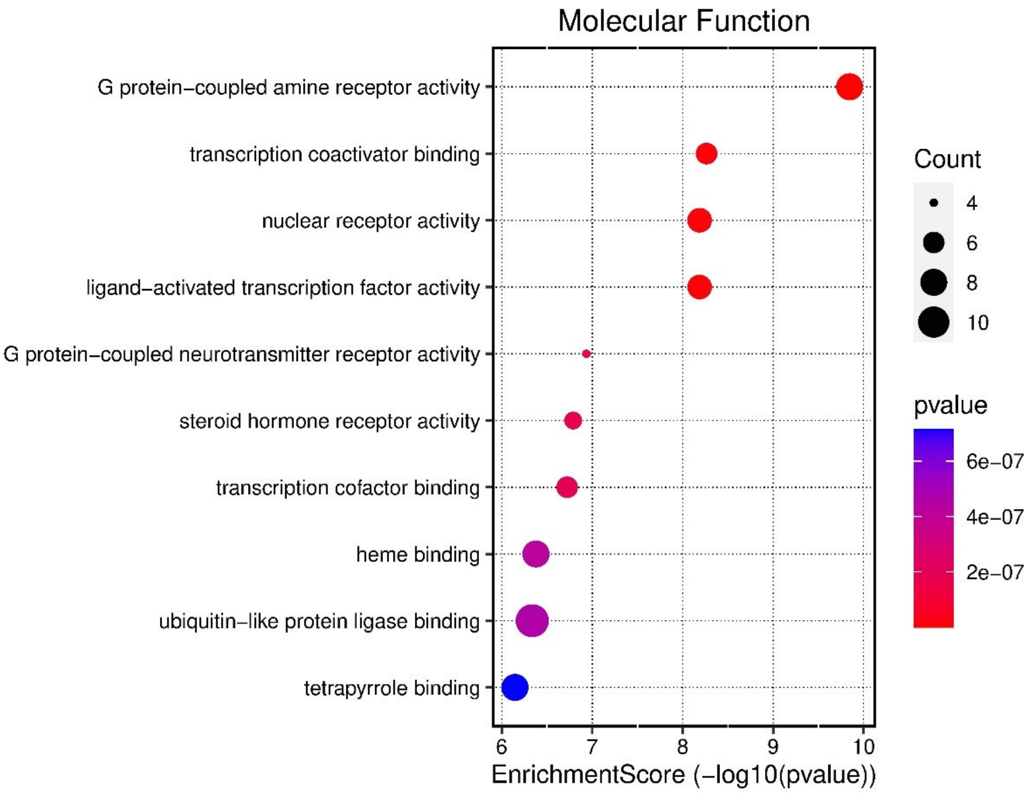

Figure S2 Bubble diagram of Molecular Funtion (MF) enrichment.

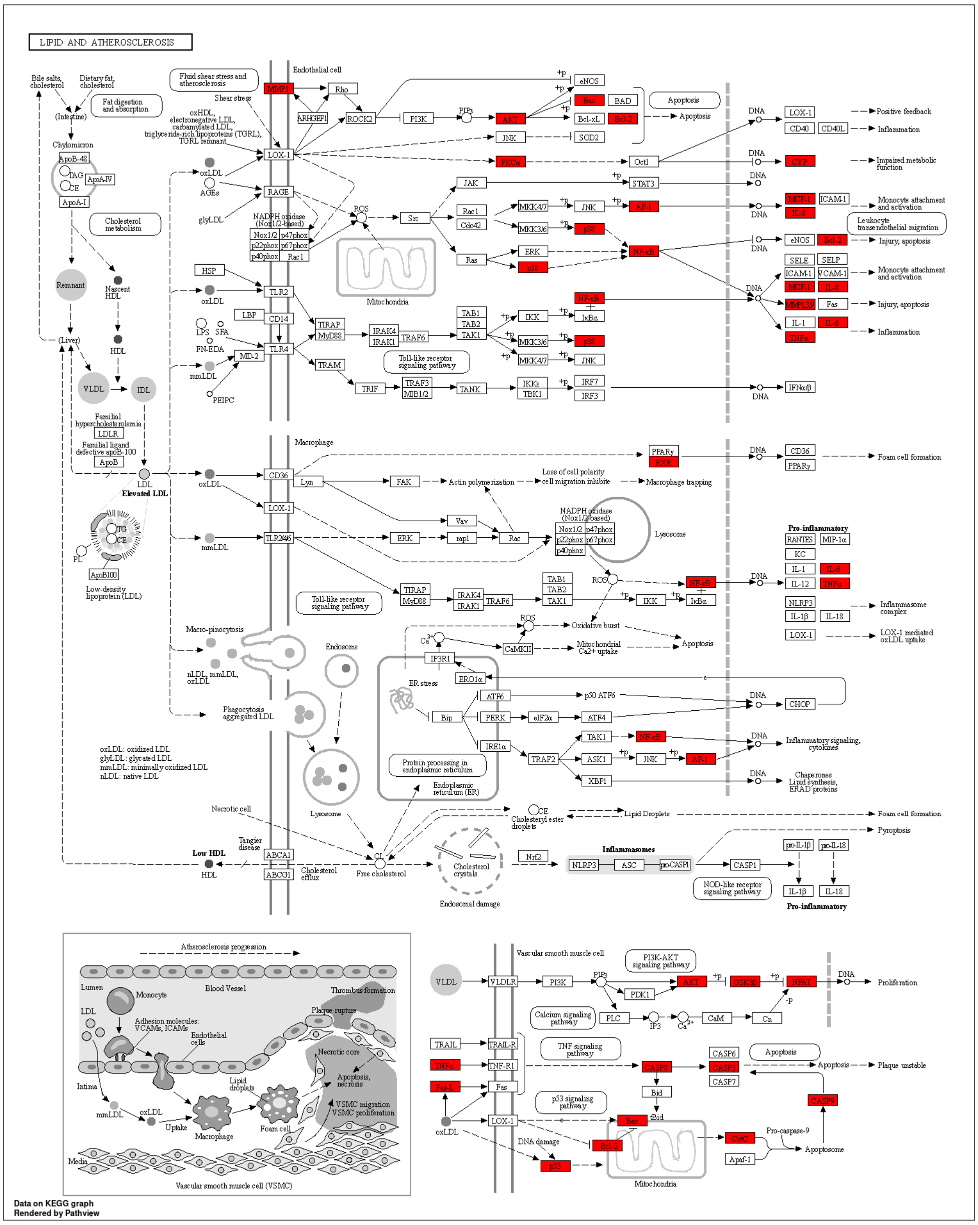

Figure S3 The Lipid and atherosclerosis (HSA05417) pathway by the KEGG mapper, the red represents target of Scutellaria baicalensis in the Lipid and atherosclerosis pathway.

Table S1. Results of BP GO terms enrichment of Scutellaria baicalensis in breast cancer.

ID	Description	pvalue	qvalue	Count
GO:0042493	response to drug	8.16198E-19	1.47689E-15	23
GO:0048545	response to steroid hormone	2.14771E-16	1.94311E-13	21
GO:0032496	response to lipopolysaccharide	1.77376E-15	1.06986E-12	20
GO:0006979	response to oxidative stress	3.59148E-15	1.62467E-12	19
GO:0002237	response to molecule of bacterial origin	5.67948E-15	2.05538E-12	19
GO:0007568	aging	1.83192E-13	5.52467E-11	19
GO:0019932	second-messenger-mediated signaling	4.82922E-13	1.20972E-10	18
GO:0072593	reactive oxygen species metabolic process	5.34839E-13	1.20972E-10	18
GO:1901216	positive regulation of neuron death	1.25039E-12	2.33344E-10	18
GO:0062197	cellular response to chemical stress	1.28957E-12	2.33344E-10	17
GO:1901214	regulation of neuron death	2.79866E-12	4.55218E-10	17
GO:0048608	reproductive structure development	3.27047E-12	4.55218E-10	17
GO:0048732	gland development	3.27047E-12	4.55218E-10	16
GO:0061458	reproductive system development	3.79982E-12	4.9112E-10	16
GO:0000302	response to reactive oxygen species	6.71133E-12	8.09598E-10	16
GO:1901654	response to ketone	1.34143E-11	1.51705E-09	16
GO:0070997	neuron death	1.576E-11	1.67749E-09	16
GO:0010038	response to metal ion	2.01817E-11	2.02879E-09	16
GO:0034599	cellular response to oxidative stress	2.18591E-11	2.08176E-09	15
GO:0050673	epithelial cell proliferation	4.89034E-11	4.42447E-09	15
GO:0034349	glial cell apoptotic process	5.14895E-11	4.43661E-09	15

GO:0009411	response to UV	7.20491E-11	5.92595E-09	15
GO:0032355	response to estradiol	7.78408E-11	6.12395E-09	14
GO:0030879	mammary gland development	8.4048E-11	6.33678E-09	14
GO:2001234	negative regulation of apoptotic signaling pathway	9.10494E-11	6.59006E-09	14
GO:0003018	vascular process in circulatory system	1.21104E-10	8.1953E-09	14
GO:0051384	response to glucocorticoid	1.22286E-10	8.1953E-09	13
GO:0048638	regulation of developmental growth	1.35771E-10	8.77407E-09	13
GO:0006352	DNA-templated transcription, initiation	2.07491E-10	1.27262E-08	13
GO:0048511	rhythmic process	2.16252E-10	1.27262E-08	13
GO:0009636	response to toxic substance	2.18026E-10	1.27262E-08	13
GO:0042391	regulation of membrane potential	3.38765E-10	1.91559E-08	13
GO:0043525	positive regulation of neuron apoptotic process	3.88256E-10	2.11364E-08	13
GO:0031960	response to corticosteroid	3.97152E-10	2.11364E-08	13
GO:1902895	positive regulation of pri-miRNA transcription by RNA polymerase II	7.86857E-10	4.06799E-08	13
GO:0031667	response to nutrient levels	8.77387E-10	4.37367E-08	13
GO:0044706	multi-multicellular organism process	8.94326E-10	4.37367E-08	13
GO:0097191	extrinsic apoptotic signaling pathway	1.09256E-09	5.2025E-08	12
GO:2001233	regulation of apoptotic signaling pathway	1.18792E-09	5.51156E-08	12
GO:0097305	response to alcohol	1.32964E-09	5.93153E-08	12
GO:0097193	intrinsic apoptotic signaling pathway	1.344E-09	5.93153E-08	12
GO:0035296	regulation of tube diameter	1.73608E-09	7.30555E-08	12
GO:0097746	regulation of blood vessel diameter	1.73608E-09	7.30555E-08	12

GO:0006367	transcription initiation from RNA polymerase II promoter	1.79627E-09	7.38707E-08	12
GO:0035150	regulation of tube size	1.85834E-09	7.47247E-08	12
GO:0033002	muscle cell proliferation	2.13838E-09	8.41163E-08	12
GO:2001237	negative regulation of extrinsic apoptotic signaling pathway	2.29746E-09	8.84508E-08	12
GO:0007565	female pregnancy	2.63575E-09	9.93609E-08	12
GO:0038034	signal transduction in absence of ligand	2.77439E-09	1.00404E-07	12
GO:0097192	extrinsic apoptotic signaling pathway in absence of ligand	2.77439E-09	1.00404E-07	12
GO:0019933	cAMP-mediated signaling	3.09229E-09	1.09714E-07	12
GO:0009314	response to radiation	3.4837E-09	1.21224E-07	12
GO:0001890	placenta development	4.04777E-09	1.38195E-07	12
GO:0009416	response to light stimulus	4.25154E-09	1.42464E-07	11
GO:1902893	regulation of pri-miRNA transcription by RNA polymerase II	4.681E-09	1.54003E-07	11
GO:0007187	G protein-coupled receptor signaling pathway, coupled to cyclic nucleotide second messenger	4.98268E-09	1.61001E-07	11
GO:0009612	response to mechanical stimulus	5.16969E-09	1.64113E-07	11
GO:0050678	regulation of epithelial cell proliferation	6.11762E-09	1.87395E-07	11
GO:0061614	pri-miRNA transcription by RNA polymerase II	6.18384E-09	1.87395E-07	11
GO:0048145	regulation of fibroblast proliferation	6.2861E-09	1.87395E-07	11
GO:0070482	response to oxygen levels	6.31737E-09	1.87395E-07	11
GO:0043523	regulation of neuron apoptotic process	6.61719E-09	1.93123E-07	11
GO:0048144	fibroblast proliferation	6.92061E-09	1.96406E-07	11
GO:0043281	regulation of cysteine-type endopeptidase activity involved in apoptotic process	6.94676E-09	1.96406E-07	11
GO:0019935	cyclic-nucleotide-mediated signaling	1.06415E-08	2.9624E-07	11

GO:0043270	positive regulation of ion transport	1.17636E-08	3.22513E-07	11
GO:0031649	heat generation	1.3811E-08	3.72994E-07	11
GO:0007188	adenylate cyclase-modulating G protein-coupled receptor signaling pathway	1.60009E-08	4.25782E-07	11
GO:0001666	response to hypoxia	1.7384E-08	4.55883E-07	11
GO:2000116	regulation of cysteine-type endopeptidase activity	2.07998E-08	5.37667E-07	11
GO:0036293	response to decreased oxygen levels	2.56242E-08	6.38417E-07	11
GO:0060749	mammary gland alveolus development	2.57558E-08	6.38417E-07	11
GO:0061377	mammary gland lobule development	2.57558E-08	6.38417E-07	11
GO:0051402	neuron apoptotic process	2.68429E-08	6.56372E-07	11
GO:0071216	cellular response to biotic stimulus	2.79896E-08	6.75286E-07	11
GO:0018105	peptidyl-serine phosphorylation	3.10234E-08	7.38632E-07	11
GO:0051412	response to corticosterone	3.42107E-08	8.03939E-07	11
GO:0001935	endothelial cell proliferation	4.16246E-08	9.65624E-07	11
GO:2000377	regulation of reactive oxygen species metabolic process	4.36439E-08	9.99651E-07	11
GO:0061180	mammary gland epithelium development	4.97338E-08	1.1249E-06	11
GO:0071887	leukocyte apoptotic process	5.08668E-08	1.13632E-06	11
GO:0018209	peptidyl-serine modification	6.79557E-08	1.49956E-06	11
GO:0050679	positive regulation of epithelial cell proliferation	7.22707E-08	1.57557E-06	11
GO:2001236	regulation of extrinsic apoptotic signaling pathway	8.72146E-08	1.87872E-06	11
GO:0042310	vasoconstriction	9.61378E-08	2.04657E-06	11
GO:0043536	positive regulation of blood vessel endothelial cell migration	1.05074E-07	2.2108E-06	10
GO:0010822	positive regulation of mitochondrion organization	1.0854E-07	2.25748E-06	10

GO:0002064	epithelial cell development	1.11516E-07	2.29302E-06	10
GO:0034614	cellular response to reactive oxygen species	1.31844E-07	2.65366E-06	10
GO:0051222	positive regulation of protein transport	1.31988E-07	2.65366E-06	10
GO:0030099	myeloid cell differentiation	1.46758E-07	2.91818E-06	10
GO:0022612	gland morphogenesis	1.4941E-07	2.93862E-06	10
GO:0048660	regulation of smooth muscle cell proliferation	1.53098E-07	2.97059E-06	10
GO:0032409	regulation of transporter activity	1.54318E-07	2.97059E-06	10
GO:0008637	apoptotic mitochondrial changes	1.59002E-07	3.02852E-06	10
GO:0048146	positive regulation of fibroblast proliferation	1.61197E-07	3.03836E-06	10
GO:0048659	smooth muscle cell proliferation	1.68871E-07	3.15019E-06	10
GO:0010634	positive regulation of epithelial cell migration	1.77275E-07	3.27321E-06	10
GO:0097756	negative regulation of blood vessel diameter	1.89522E-07	3.464E-06	10
GO:0071880	adenylate cyclase-activating adrenergic receptor signaling pathway	2.10364E-07	3.7696E-06	10
GO:0045862	positive regulation of proteolysis	2.12492E-07	3.7696E-06	10
GO:1904951	positive regulation of establishment of protein localization	2.12492E-07	3.7696E-06	10
GO:2001235	positive regulation of apoptotic signaling pathway	2.46984E-07	4.33895E-06	10
GO:0001936	regulation of endothelial cell proliferation	2.58669E-07	4.50053E-06	10
GO:0046683	response to organophosphorus	2.71921E-07	4.66042E-06	10
GO:0051090	regulation of DNA-binding transcription factor activity	2.7301E-07	4.66042E-06	10
GO:0051899	membrane depolarization	2.79977E-07	4.73468E-06	10
GO:0042133	neurotransmitter metabolic process	3.02718E-07	5.07186E-06	10
GO:0001701	in utero embryonic development	3.07557E-07	5.10565E-06	10

GO:0010821	regulation of mitochondrion organization	3.54783E-07	5.78351E-06	10
GO:0035265	organ growth	3.54783E-07	5.78351E-06	10
GO:0052547	regulation of peptidase activity	3.58282E-07	5.7884E-06	10
GO:0007589	body fluid secretion	3.76315E-07	5.97309E-06	10
GO:0097194	execution phase of apoptosis	3.76315E-07	5.97309E-06	10
GO:0051385	response to mineralocorticoid	4.24533E-07	6.62226E-06	10
GO:0071875	adrenergic receptor signaling pathway	4.24533E-07	6.62226E-06	10
GO:0001836	release of cytochrome c from mitochondria	4.32792E-07	6.63667E-06	10
GO:0019229	regulation of vasoconstriction	4.32792E-07	6.63667E-06	10
GO:0071496	cellular response to external stimulus	4.79114E-07	7.28525E-06	10
GO:1903799	negative regulation of production of miRNAs involved in gene silencing by miRNA	4.8861E-07	7.36773E-06	10
GO:0010039	response to iron ion	4.98445E-07	7.45391E-06	10
GO:0010952	positive regulation of peptidase activity	5.45221E-07	8.08659E-06	10
GO:0007189	adenylate cyclase-activating G protein-coupled receptor signaling pathway	5.52519E-07	8.1282E-06	10
GO:0045639	positive regulation of myeloid cell differentiation	5.72197E-07	8.29462E-06	10
GO:0033157	regulation of intracellular protein transport	5.76705E-07	8.29462E-06	9
GO:0042759	long-chain fatty acid biosynthetic process	5.82167E-07	8.29462E-06	9
GO:0045907	positive regulation of vasoconstriction	5.82167E-07	8.29462E-06	9
GO:0030522	intracellular receptor signaling pathway	5.96973E-07	8.43911E-06	9
GO:0014074	response to purine-containing compound	6.12415E-07	8.59029E-06	9
GO:0060135	maternal process involved in female pregnancy	6.38202E-07	8.88315E-06	9
GO:0006606	protein import into nucleus	6.44387E-07	8.89821E-06	9

GO:0008630	intrinsic apoptotic signaling pathway in response to DNA damage	6.54036E-07	8.89821E-06	9
GO:0048661	positive regulation of smooth muscle cell proliferation	6.54036E-07	8.89821E-06	9
GO:1901030	positive regulation of mitochondrial outer membrane permeabilization involved in apoptotic signaling pathway	6.76619E-07	9.13675E-06	9
GO:0007200	phospholipase C-activating G protein-coupled receptor signaling pathway	6.98507E-07	9.36244E-06	9
GO:0071222	cellular response to lipopolysaccharide	7.26335E-07	9.66385E-06	9
GO:0002573	myeloid leukocyte differentiation	7.86804E-07	1.03504E-05	9
GO:0032091	negative regulation of protein binding	7.95094E-07	1.03504E-05	9
GO:2000379	positive regulation of reactive oxygen species metabolic process	7.95094E-07	1.03504E-05	9
GO:0002685	regulation of leukocyte migration	8.51585E-07	1.10066E-05	9
GO:0098926	postsynaptic signal transduction	9.01648E-07	1.1571E-05	9
GO:0015980	energy derivation by oxidation of organic compounds	9.23229E-07	1.17645E-05	9
GO:0071276	cellular response to cadmium ion	1.03432E-06	1.3088E-05	9
GO:0007623	circadian rhythm	1.07441E-06	1.35008E-05	9
GO:0043467	regulation of generation of precursor metabolites and energy	1.15432E-06	1.43102E-05	9
GO:0046822	regulation of nucleocytoplasmic transport	1.15464E-06	1.43102E-05	9
GO:0032386	regulation of intracellular transport	1.20214E-06	1.47976E-05	9
GO:0001938	positive regulation of endothelial cell proliferation	1.2261E-06	1.49906E-05	9
GO:0032388	positive regulation of intracellular transport	1.24952E-06	1.50732E-05	9
GO:0071219	cellular response to molecule of bacterial origin	1.24952E-06	1.50732E-05	9
GO:0043535	regulation of blood vessel endothelial cell migration	1.26619E-06	1.51731E-05	9
GO:0008406	gonad development	1.29697E-06	1.53667E-05	9
GO:0022407	regulation of cell-cell adhesion	1.29933E-06	1.53667E-05	9

GO:0043620	regulation of DNA-templated transcription in response to stress	1.38019E-06	1.61939E-05	9
GO:2001242	regulation of intrinsic apoptotic signaling pathway	1.38718E-06	1.61939E-05	9
GO:0035994	response to muscle stretch	1.47176E-06	1.70712E-05	9
GO:0006801	superoxide metabolic process	1.51717E-06	1.74859E-05	9
GO:0051100	negative regulation of binding	1.58708E-06	1.81758E-05	9
GO:0045137	development of primary sexual characteristics	1.6157E-06	1.83872E-05	9
GO:0051170	import into nucleus	1.73356E-06	1.96052E-05	9
GO:1903524	positive regulation of blood circulation	1.77668E-06	1.9968E-05	9
GO:0007213	G protein-coupled acetylcholine receptor signaling pathway	1.85736E-06	2.07459E-05	9
GO:0006633	fatty acid biosynthetic process	1.8914E-06	2.08692E-05	9
GO:0010632	regulation of epithelial cell migration	1.89146E-06	2.08692E-05	9
GO:0008631	intrinsic apoptotic signaling pathway in response to oxidative stress	1.94626E-06	2.13436E-05	9
GO:1903522	regulation of blood circulation	2.00717E-06	2.18791E-05	9
GO:0006690	icosanoid metabolic process	2.05328E-06	2.22017E-05	9
GO:1901568	fatty acid derivative metabolic process	2.06131E-06	2.22017E-05	9
GO:0060965	negative regulation of gene silencing by miRNA	2.31314E-06	2.47667E-05	9
GO:0035094	response to nicotine	2.45156E-06	2.59417E-05	9
GO:1901028	regulation of mitochondrial outer membrane permeabilization involved in apoptotic signaling pathway	2.45156E-06	2.59417E-05	9
GO:2000134	negative regulation of G1/S transition of mitotic cell cycle	2.54808E-06	2.68063E-05	9
GO:0034644	cellular response to UV	2.58706E-06	2.70591E-05	9
GO:1900180	regulation of protein localization to nucleus	2.68623E-06	2.79349E-05	9
GO:0032570	response to progesterone	2.74011E-06	2.83323E-05	8

GO:0090316	positive regulation of intracellular protein transport	2.76219E-06	2.83983E-05	8
GO:1903409	reactive oxygen species biosynthetic process	2.83058E-06	2.89371E-05	8
GO:0006953	acute-phase response	3.05469E-06	3.10527E-05	8
GO:2000045	regulation of G1/S transition of mitotic cell cycle	3.11937E-06	3.15331E-05	8
GO:0006631	fatty acid metabolic process	3.18913E-06	3.1882E-05	8
GO:0045787	positive regulation of cell cycle	3.18913E-06	3.1882E-05	8
GO:0034612	response to tumor necrosis factor	3.26709E-06	3.24819E-05	8
GO:0008217	regulation of blood pressure	3.37868E-06	3.30466E-05	8
GO:0048639	positive regulation of developmental growth	3.37868E-06	3.30466E-05	8
GO:0061138	morphogenesis of a branching epithelium	3.37868E-06	3.30466E-05	8
GO:0010595	positive regulation of endothelial cell migration	3.47447E-06	3.35847E-05	8
GO:1902807	negative regulation of cell cycle G1/S phase transition	3.47447E-06	3.35847E-05	8
GO:0051051	negative regulation of transport	3.48937E-06	3.35847E-05	8
GO:0009895	negative regulation of catabolic process	3.54934E-06	3.39811E-05	8
GO:0042770	signal transduction in response to DNA damage	3.65324E-06	3.44663E-05	8
GO:0043534	blood vessel endothelial cell migration	3.65606E-06	3.44663E-05	8
GO:0001776	leukocyte homeostasis	3.6762E-06	3.44663E-05	8
GO:0006919	activation of cysteine-type endopeptidase activity involved in apoptotic process	3.6762E-06	3.44663E-05	8
GO:0007595	lactation	3.76865E-06	3.4906E-05	8
GO:0070231	T cell apoptotic process	3.76865E-06	3.4906E-05	8
GO:0045926	negative regulation of growth	3.78097E-06	3.4906E-05	8
GO:0043280	positive regulation of cysteine-type endopeptidase activity involved in apoptotic process	3.83961E-06	3.52674E-05	8

GO:0034765	regulation of ion transmembrane transport	3.95933E-06	3.61833E-05	8
GO:0097300	programmed necrotic cell death	4.17153E-06	3.72689E-05	8
GO:0060149	negative regulation of posttranscriptional gene silencing	4.1811E-06	3.72689E-05	8
GO:0060967	negative regulation of gene silencing by RNA	4.1811E-06	3.72689E-05	8
GO:1901522	positive regulation of transcription from RNA polymerase II promoter involved in cellular response to chemical stimulus	4.1811E-06	3.72689E-05	8
GO:1903798	regulation of production of miRNAs involved in gene silencing by miRNA	4.1811E-06	3.72689E-05	8
GO:0071248	cellular response to metal ion	4.26917E-06	3.78674E-05	8
GO:0060562	epithelial tube morphogenesis	4.40805E-06	3.85326E-05	8
GO:0071214	cellular response to abiotic stimulus	4.40805E-06	3.85326E-05	8
GO:0104004	cellular response to environmental stimulus	4.40805E-06	3.85326E-05	8
GO:0001892	embryonic placenta development	4.49145E-06	3.8886E-05	8
GO:0008625	extrinsic apoptotic signaling pathway via death domain receptors	4.49145E-06	3.8886E-05	8
GO:0001503	ossification	4.65596E-06	4.01183E-05	8
GO:1903351	cellular response to dopamine	4.79374E-06	4.11097E-05	8
GO:1903350	response to dopamine	5.11236E-06	4.36353E-05	8
GO:1903829	positive regulation of cellular protein localization	5.30199E-06	4.50413E-05	8
GO:0017038	protein import	5.55243E-06	4.69485E-05	8
GO:0001763	morphogenesis of a branching structure	5.75998E-06	4.82525E-05	8
GO:0031099	regeneration	5.75998E-06	4.82525E-05	8
GO:0015850	organic hydroxy compound transport	5.84428E-06	4.8733E-05	8
GO:0050927	positive regulation of positive chemotaxis	5.92909E-06	4.89887E-05	8
GO:0070920	regulation of production of small RNA involved in gene silencing by RNA	5.92909E-06	4.89887E-05	8

GO:0010212	response to ionizing radiation	6.18092E-06	5.08139E-05	8
GO:0051091	positive regulation of DNA-binding transcription factor activity	6.20616E-06	5.08139E-05	8
GO:0050727	regulation of inflammatory response	6.2526E-06	5.09636E-05	8
GO:0033135	regulation of peptidyl-serine phosphorylation	6.4692E-06	5.24926E-05	8
GO:0010506	regulation of autophagy	6.67944E-06	5.37427E-05	8
GO:0045785	positive regulation of cell adhesion	6.68267E-06	5.37427E-05	8
GO:0097345	mitochondrial outer membrane permeabilization	6.72042E-06	5.38072E-05	8
GO:0042542	response to hydrogen peroxide	6.76857E-06	5.3954E-05	8
GO:1902806	regulation of cell cycle G1/S phase transition	6.89911E-06	5.47533E-05	8
GO:0050926	regulation of positive chemotaxis	6.98131E-06	5.51638E-05	8
GO:0055123	digestive system development	7.07937E-06	5.56954E-05	8
GO:0019216	regulation of lipid metabolic process	7.13836E-06	5.59164E-05	8
GO:0060688	regulation of morphogenesis of a branching structure	7.35134E-06	5.7052E-05	8
GO:0071456	cellular response to hypoxia	7.40529E-06	5.7052E-05	8
GO:0042100	B cell proliferation	7.40559E-06	5.7052E-05	8
GO:0022898	regulation of transmembrane transporter activity	7.40946E-06	5.7052E-05	8
GO:0052548	regulation of endopeptidase activity	7.62097E-06	5.82412E-05	8
GO:0034504	protein localization to nucleus	7.62828E-06	5.82412E-05	8
GO:2001243	negative regulation of intrinsic apoptotic signaling pathway	7.85819E-06	5.97445E-05	8
GO:0034764	positive regulation of transmembrane transport	7.94254E-06	5.99805E-05	8
GO:0006913	nucleocytoplasmic transport	7.95553E-06	5.99805E-05	8
GO:0035264	multicellular organism growth	8.08382E-06	6.06948E-05	8

GO:0001963	synaptic transmission, dopaminergic	8.16529E-06	6.10532E-05	8
GO:0043393	regulation of protein binding	8.22329E-06	6.12338E-05	8
GO:0007548	sex differentiation	8.31828E-06	6.16873E-05	8
GO:0000077	DNA damage checkpoint	8.44383E-06	6.18579E-05	8
GO:0061041	regulation of wound healing	8.44383E-06	6.18579E-05	8
GO:2001056	positive regulation of cysteine-type endopeptidase activity	8.44383E-06	6.18579E-05	8
GO:0051169	nuclear transport	8.56392E-06	6.24846E-05	8
GO:0002763	positive regulation of myeloid leukocyte differentiation	8.75175E-06	6.33443E-05	8
GO:0019369	arachidonic acid metabolic process	8.75175E-06	6.33443E-05	8
GO:0008585	female gonad development	8.83074E-06	6.36613E-05	8
GO:0046651	lymphocyte proliferation	9.06104E-06	6.50624E-05	8
GO:0006006	glucose metabolic process	9.11637E-06	6.52009E-05	8
GO:1901342	regulation of vasculature development	9.44157E-06	6.68095E-05	8
GO:0095500	acetylcholine receptor signaling pathway	9.49111E-06	6.68095E-05	8
GO:0031663	lipopolysaccharide-mediated signaling pathway	9.52589E-06	6.68095E-05	8
GO:0042306	regulation of protein import into nucleus	9.52589E-06	6.68095E-05	8
GO:0046456	icosanoid biosynthetic process	9.52589E-06	6.68095E-05	8
GO:0006936	muscle contraction	9.66751E-06	6.75409E-05	8
GO:0032943	mononuclear cell proliferation	9.85986E-06	6.86199E-05	8
GO:0048754	branching morphogenesis of an epithelial tube	1.00199E-05	6.94663E-05	8
GO:0010631	epithelial cell migration	1.0387E-05	7.17366E-05	8
GO:0036294	cellular response to decreased oxygen levels	1.04335E-05	7.1765E-05	8

GO:1901570	fatty acid derivative biosynthetic process	1.04704E-05	7.1765E-05	8
GO:0051098	regulation of binding	1.0892E-05	7.40613E-05	8
GO:0043271	negative regulation of ion transport	1.0895E-05	7.40613E-05	7
GO:0048568	embryonic organ development	1.09316E-05	7.40613E-05	7
GO:0001782	B cell homeostasis	1.09692E-05	7.40613E-05	7
GO:0002027	regulation of heart rate	1.10687E-05	7.44556E-05	7
GO:0090132	epithelium migration	1.11523E-05	7.474E-05	7
GO:1902110	positive regulation of mitochondrial membrane permeability involved in apoptotic process	1.12342E-05	7.50109E-05	7
GO:0071241	cellular response to inorganic substance	1.15236E-05	7.66607E-05	7
GO:0046545	development of primary female sexual characteristics	1.16944E-05	7.75115E-05	7
GO:0062012	regulation of small molecule metabolic process	1.21177E-05	7.98094E-05	7
GO:0002260	lymphocyte homeostasis	1.21734E-05	7.98094E-05	7
GO:1904589	regulation of protein import	1.21734E-05	7.98094E-05	7
GO:0071868	cellular response to monoamine stimulus	1.23483E-05	8.01504E-05	7
GO:0071870	cellular response to catecholamine stimulus	1.23483E-05	8.01504E-05	7
GO:0001844	protein insertion into mitochondrial membrane involved in apoptotic signaling pathway	1.26102E-05	8.01504E-05	7
GO:1905144	response to acetylcholine	1.26102E-05	8.01504E-05	7
GO:1905145	cellular response to acetylcholine	1.26102E-05	8.01504E-05	7
GO:0051146	striated muscle cell differentiation	1.26279E-05	8.01504E-05	7
GO:0001660	fever generation	1.27126E-05	8.01504E-05	7
GO:0019614	catechol-containing compound catabolic process	1.27126E-05	8.01504E-05	7
GO:0032025	response to cobalt ion	1.27126E-05	8.01504E-05	7

GO:0034350	regulation of glial cell apoptotic process	1.27126E-05	8.01504E-05	7
GO:0042424	catecholamine catabolic process	1.27126E-05	8.01504E-05	7
GO:0090130	tissue migration	1.28301E-05	8.03683E-05	7
GO:0031570	DNA integrity checkpoint	1.2836E-05	8.03683E-05	7
GO:0031571	mitotic G1 DNA damage checkpoint	1.31728E-05	8.19098E-05	7
GO:1902686	mitochondrial outer membrane permeabilization involved in programmed cell death	1.31728E-05	8.19098E-05	7
GO:0044783	G1 DNA damage checkpoint	1.4235E-05	8.73148E-05	7
GO:0044819	mitotic G1/S transition checkpoint	1.4235E-05	8.73148E-05	7
GO:0046686	response to cadmium ion	1.4235E-05	8.73148E-05	7
GO:0070265	necrotic cell death	1.4235E-05	8.73148E-05	7
GO:0002675	positive regulation of acute inflammatory response	1.44251E-05	8.76881E-05	7
GO:0006939	smooth muscle contraction	1.44897E-05	8.76881E-05	7
GO:0033138	positive regulation of peptidyl-serine phosphorylation	1.44897E-05	8.76881E-05	7
GO:0043618	regulation of transcription from RNA polymerase II promoter in response to stress	1.44897E-05	8.76881E-05	7
GO:0003012	muscle system process	1.5128E-05	9.11434E-05	7
GO:0050890	cognition	1.52164E-05	9.11434E-05	7
GO:0071867	response to monoamine	1.52667E-05	9.11434E-05	7
GO:0071869	response to catecholamine	1.52667E-05	9.11434E-05	7
GO:0006940	regulation of smooth muscle contraction	1.53629E-05	9.11434E-05	7
GO:0035794	positive regulation of mitochondrial membrane permeability	1.53629E-05	9.11434E-05	7
GO:0002526	acute inflammatory response	1.6077E-05	9.49644E-05	7
GO:0001654	eye development	1.61119E-05	9.49644E-05	7

GO:0034250	positive regulation of cellular amide metabolic process	1.62784E-05	9.5634E-05	7
GO:0010165	response to X-ray	1.64251E-05	9.6184E-05	7
GO:0046824	positive regulation of nucleocytoplasmic transport	1.65592E-05	9.63456E-05	7
GO:1902108	regulation of mitochondrial membrane permeability involved in apoptotic process	1.65592E-05	9.63456E-05	7
GO:0009266	response to temperature stimulus	1.68964E-05	9.79923E-05	7
GO:0031652	positive regulation of heat generation	1.74188E-05	0.000100378	7
GO:0042135	neurotransmitter catabolic process	1.74188E-05	0.000100378	7
GO:0150063	visual system development	1.7614E-05	0.000101181	7
GO:0001676	long-chain fatty acid metabolic process	1.78018E-05	0.000101296	7
GO:1900015	regulation of cytokine production involved in inflammatory response	1.78269E-05	0.000101296	7
GO:1905710	positive regulation of membrane permeability	1.78269E-05	0.000101296	7
GO:0001505	regulation of neurotransmitter levels	1.79698E-05	0.000101296	7
GO:0045444	fat cell differentiation	1.79698E-05	0.000101296	7
GO:0071453	cellular response to oxygen levels	1.79698E-05	0.000101296	7
GO:0031331	positive regulation of cellular catabolic process	1.84093E-05	0.00010313	7
GO:0042692	muscle cell differentiation	1.84093E-05	0.00010313	7
GO:0032411	positive regulation of transporter activity	1.87185E-05	0.000104539	7
GO:0040014	regulation of multicellular organism growth	1.9169E-05	0.000106725	7
GO:0010594	regulation of endothelial cell migration	1.96871E-05	0.000109274	7
GO:0001667	ameboidal-type cell migration	1.98848E-05	0.000110034	7
GO:0048880	sensory system development	2.0093E-05	0.000110847	7
GO:0070661	leukocyte proliferation	2.01953E-05	0.000111072	7

GO:0006937	regulation of muscle contraction	2.04512E-05	0.000112139	7
GO:0042698	ovulation cycle	2.05883E-05	0.00011255	7
GO:0010959	regulation of metal ion transport	2.09834E-05	0.000114364	7
GO:0001659	temperature homeostasis	2.12257E-05	0.000114992	7
GO:0010469	regulation of signaling receptor activity	2.12257E-05	0.000114992	7
GO:0018107	peptidyl-threonine phosphorylation	2.16994E-05	0.000117207	7
GO:0002534	cytokine production involved in inflammatory response	2.20882E-05	0.000118952	7
GO:0007569	cell aging	2.27739E-05	0.000122281	7
GO:1904031	positive regulation of cyclin-dependent protein kinase activity	2.36531E-05	0.000126626	7
GO:0046660	female sex differentiation	2.38909E-05	0.000127522	7
GO:0045765	regulation of angiogenesis	2.43724E-05	0.000129709	7
GO:0072330	monocarboxylic acid biosynthetic process	2.49543E-05	0.000132417	7
GO:0048469	cell maturation	2.54705E-05	0.000134761	7
GO:0045742	positive regulation of epidermal growth factor receptor signaling pathway	2.65119E-05	0.000139051	7
GO:1901099	negative regulation of signal transduction in absence of ligand	2.65119E-05	0.000139051	7
GO:2001240	negative regulation of extrinsic apoptotic signaling pathway in absence of ligand	2.65119E-05	0.000139051	7
GO:0070227	lymphocyte apoptotic process	2.71024E-05	0.000140923	6
GO:0072401	signal transduction involved in DNA integrity checkpoint	2.71024E-05	0.000140923	6
GO:0072422	signal transduction involved in DNA damage checkpoint	2.71024E-05	0.000140923	6
GO:0010950	positive regulation of endopeptidase activity	2.73535E-05	0.000141821	6
GO:0045471	response to ethanol	2.75092E-05	0.000142221	6
GO:0051881	regulation of mitochondrial membrane potential	2.89564E-05	0.000148852	6

GO:0072395	signal transduction involved in cell cycle checkpoint	2.89564E-05	0.000148852	6
GO:0042113	B cell activation	2.91721E-05	0.000149536	6
GO:1903034	regulation of response to wounding	2.93498E-05	0.000150022	6
GO:0060969	negative regulation of gene silencing	2.96162E-05	0.000150957	6
GO:0010623	programmed cell death involved in cell development	2.9982E-05	0.000151541	6
GO:0019336	phenol-containing compound catabolic process	2.9982E-05	0.000151541	6
GO:0031650	regulation of heat generation	2.9982E-05	0.000151541	6
GO:0002688	regulation of leukocyte chemotaxis	3.01572E-05	0.000152002	6
GO:0035924	cellular response to vascular endothelial growth factor stimulus	3.09074E-05	0.00015535	6
GO:0019318	hexose metabolic process	3.13563E-05	0.00015717	6
GO:0001952	regulation of cell-matrix adhesion	3.15559E-05	0.000157734	6
GO:0043029	T cell homeostasis	3.29787E-05	0.000163028	6
GO:1901186	positive regulation of ERBB signaling pathway	3.29787E-05	0.000163028	6
GO:2000144	positive regulation of DNA-templated transcription, initiation	3.29787E-05	0.000163028	6
GO:0018210	peptidyl-threonine modification	3.30063E-05	0.000163028	6
GO:0048872	homeostasis of number of cells	3.31556E-05	0.000163028	6
GO:0090257	regulation of muscle system process	3.31556E-05	0.000163028	6
GO:0071478	cellular response to radiation	3.48707E-05	0.000170996	6
GO:0046902	regulation of mitochondrial membrane permeability	3.51144E-05	0.000171263	6
GO:0150076	neuroinflammatory response	3.51144E-05	0.000171263	6
GO:0007210	serotonin receptor signaling pathway	3.66127E-05	0.00017809	6
GO:0071482	cellular response to light stimulus	3.76817E-05	0.000182799	6

GO:0090399	replicative senescence	3.80256E-05	0.000183974	6
GO:0045930	negative regulation of mitotic cell cycle	3.94982E-05	0.000190589	6
GO:0045637	regulation of myeloid cell differentiation	4.01491E-05	0.000192702	6
GO:0050730	regulation of peptidyl-tyrosine phosphorylation	4.01491E-05	0.000192702	6
GO:0016572	histone phosphorylation	4.05312E-05	0.000194022	6
GO:0032412	regulation of ion transmembrane transporter activity	4.23598E-05	0.00020224	6
GO:0071902	positive regulation of protein serine/threonine kinase activity	4.32396E-05	0.000205897	6
GO:0050731	positive regulation of peptidyl-tyrosine phosphorylation	4.40101E-05	0.000209016	6
GO:0072331	signal transduction by p53 class mediator	4.46712E-05	0.000211601	6
GO:1904062	regulation of cation transmembrane transport	4.72767E-05	0.00022248	6
GO:1900119	positive regulation of execution phase of apoptosis	4.73661E-05	0.00022248	6
GO:2000402	negative regulation of lymphocyte migration	4.73661E-05	0.00022248	6
GO:0022617	extracellular matrix disassembly	4.75828E-05	0.00022248	6
GO:0030193	regulation of blood coagulation	4.75828E-05	0.00022248	6
GO:1901988	negative regulation of cell cycle phase transition	4.83354E-05	0.000225417	6
GO:0048565	digestive tract development	4.86259E-05	0.000226188	6
GO:1900046	regulation of hemostasis	5.04416E-05	0.000234032	6
GO:0010876	lipid localization	5.12107E-05	0.000236993	6
GO:0009755	hormone-mediated signaling pathway	5.16736E-05	0.000238526	6
GO:0015844	monoamine transport	5.34313E-05	0.000246012	6
GO:0045927	positive regulation of growth	5.36084E-05	0.0002462	6
GO:0150077	regulation of neuroinflammatory response	5.41309E-05	0.000247971	6

GO:0006970	response to osmotic stress	5.65562E-05	0.000257128	6
GO:0010507	negative regulation of autophagy	5.65562E-05	0.000257128	6
GO:0043154	negative regulation of cysteine-type endopeptidase activity involved in apoptotic process	5.65562E-05	0.000257128	6
GO:0043401	steroid hormone mediated signaling pathway	5.72389E-05	0.00025958	6
GO:0002070	epithelial cell maturation	5.80932E-05	0.00026214	6
GO:0019372	lipoxygenase pathway	5.80932E-05	0.00026214	6
GO:1903708	positive regulation of hemopoiesis	5.85635E-05	0.000263605	6
GO:0007193	adenylate cyclase-inhibiting G protein-coupled receptor signaling pathway	5.98204E-05	0.000268594	6
GO:0007586	digestion	6.19806E-05	0.000276919	6
GO:0008584	male gonad development	6.19806E-05	0.000276919	6
GO:0071383	cellular response to steroid hormone stimulus	6.22805E-05	0.000277574	6
GO:0002687	positive regulation of leukocyte migration	6.44664E-05	0.000284921	6
GO:0007292	female gamete generation	6.44664E-05	0.000284921	6
GO:0046546	development of primary male sexual characteristics	6.44664E-05	0.000284921	6
GO:0051204	protein insertion into mitochondrial membrane	6.48739E-05	0.000284921	6
GO:0060443	mammary gland morphogenesis	6.48739E-05	0.000284921	6
GO:0070266	necroptotic process	6.48739E-05	0.000284921	6
GO:0009896	positive regulation of catabolic process	6.65094E-05	0.000291189	6
GO:0050818	regulation of coagulation	6.67838E-05	0.000291189	6
GO:0090559	regulation of membrane permeability	6.67838E-05	0.000291189	6
GO:0050921	positive regulation of chemotaxis	6.96769E-05	0.000302133	6
GO:0060078	regulation of postsynaptic membrane potential	6.96769E-05	0.000302133	6

GO:0006109	regulation of carbohydrate metabolic process	7.02946E-05	0.000302133	6
GO:0010224	response to UV-B	7.02956E-05	0.000302133	6
GO:0070242	thymocyte apoptotic process	7.02956E-05	0.000302133	6
GO:2001267	regulation of cysteine-type endopeptidase activity involved in apoptotic signaling pathway	7.02956E-05	0.000302133	6
GO:2000142	regulation of DNA-templated transcription, initiation	7.07914E-05	0.000303543	6
GO:0043542	endothelial cell migration	7.23875E-05	0.000309653	6
GO:0000082	G1/S transition of mitotic cell cycle	7.41724E-05	0.00031654	6
GO:1901215	negative regulation of neuron death	7.46069E-05	0.000317645	6
GO:0030225	macrophage differentiation	7.70925E-05	0.000325927	6
GO:0045776	negative regulation of blood pressure	7.70925E-05	0.000325927	6
GO:0046677	response to antibiotic	7.70925E-05	0.000325927	6
GO:0018108	peptidyl-tyrosine phosphorylation	8.0443E-05	0.0003393	6
GO:0070374	positive regulation of ERK1 and ERK2 cascade	8.14787E-05	0.000342869	6
GO:0009409	response to cold	8.37921E-05	0.000350971	6
GO:2001239	regulation of extrinsic apoptotic signaling pathway in absence of ligand	8.37921E-05	0.000350971	6
GO:0042136	neurotransmitter biosynthetic process	8.40603E-05	0.000351282	6
GO:0018212	peptidyl-tyrosine modification	8.54949E-05	0.000356453	6
GO:0010810	regulation of cell-substrate adhesion	8.63406E-05	0.000359152	6
GO:0002532	production of molecular mediator involved in inflammatory response	8.69358E-05	0.000359683	6
GO:2000117	negative regulation of cysteine-type endopeptidase activity	8.69358E-05	0.000359683	6
GO:0060249	anatomical structure homeostasis	8.70646E-05	0.000359683	6
GO:0034976	response to endoplasmic reticulum stress	8.77203E-05	0.000361566	6

GO:0002673	regulation of acute inflammatory response	9.09052E-05	0.00036946	6
GO:0003254	regulation of membrane depolarization	9.09052E-05	0.00036946	6
GO:0035196	production of miRNAs involved in gene silencing by miRNA	9.09052E-05	0.00036946	6
GO:0060711	labyrinthine layer development	9.09052E-05	0.00036946	6
GO:0090151	establishment of protein localization to mitochondrial membrane	9.09052E-05	0.00036946	6
GO:0090199	regulation of release of cytochrome c from mitochondria	9.09052E-05	0.00036946	6
GO:0000075	cell cycle checkpoint	9.14367E-05	0.00036946	6
GO:0051591	response to cAMP	9.1473E-05	0.00036946	6
GO:1904035	regulation of epithelial cell apoptotic process	9.1473E-05	0.00036946	6
GO:0005996	monosaccharide metabolic process	9.19479E-05	0.000370551	6
GO:0071356	cellular response to tumor necrosis factor	9.41243E-05	0.000378479	6
GO:0003073	regulation of systemic arterial blood pressure	9.61902E-05	0.000385928	6
GO:0003044	regulation of systemic arterial blood pressure mediated by a chemical signal	9.84473E-05	0.00039324	6
GO:0050873	brown fat cell differentiation	9.84473E-05	0.00039324	6
GO:0002262	myeloid cell homeostasis	0.000101003	0.000401148	6
GO:0062013	positive regulation of small molecule metabolic process	0.000101003	0.000401148	6
GO:0002690	positive regulation of leukocyte chemotaxis	0.000101092	0.000401148	6
GO:0036473	cell death in response to oxidative stress	0.000106184	0.000420431	6
GO:0006984	ER-nucleus signaling pathway	0.000106434	0.0004205	6
GO:0048592	eye morphogenesis	0.000108436	0.000427477	6
GO:0070372	regulation of ERK1 and ERK2 cascade	0.000115707	0.000454951	6
GO:0030168	platelet activation	0.000116297	0.000454951	6

GO:0003085	negative regulation of systemic arterial blood pressure	0.000116619	0.000454951	6
GO:1902004	positive regulation of amyloid-beta formation	0.000116619	0.000454951	6
GO:1901987	regulation of cell cycle phase transition	0.000116662	0.000454951	6
GO:0007044	cell-substrate junction assembly	0.000116956	0.000455116	6
GO:0050920	regulation of chemotaxis	0.000120735	0.000468813	6
GO:0043949	regulation of cAMP-mediated signaling	0.000123802	0.000478572	6
GO:0007160	cell-matrix adhesion	0.000124042	0.000478572	5
GO:0098657	import into cell	0.000124042	0.000478572	5
GO:0016049	cell growth	0.000124747	0.00048027	5
GO:0044843	cell cycle G1/S phase transition	0.000126534	0.000486116	5
GO:0044773	mitotic DNA damage checkpoint	0.000128548	0.000492805	5
GO:0034767	positive regulation of ion transmembrane transport	0.00012893	0.000493227	5
GO:0060326	cell chemotaxis	0.000129368	0.000493858	5
GO:0030857	negative regulation of epithelial cell differentiation	0.000133215	0.000505346	5
GO:0031050	dsRNA processing	0.000133215	0.000505346	5
GO:0070918	production of small RNA involved in gene silencing by RNA	0.000133215	0.000505346	5
GO:0000079	regulation of cyclin-dependent protein serine/threonine kinase activity	0.000134664	0.000508706	5
GO:0150115	cell-substrate junction organization	0.000134664	0.000508706	5
GO:0061042	vascular wound healing	0.000135581	0.000510044	5
GO:0090330	regulation of platelet aggregation	0.000135581	0.000510044	5
GO:0071695	anatomical structure maturation	0.0001417	0.000531955	5
GO:0046661	male sex differentiation	0.00014263	0.000534336	5

GO:0043279	response to alkaloid	0.000147562	0.000551674	5
GO:0006977	DNA damage response, signal transduction by p53 class mediator resulting in cell cycle arrest	0.000153582	0.000570641	5
GO:0010332	response to gamma radiation	0.000153582	0.000570641	5
GO:1904645	response to amyloid-beta	0.000153582	0.000570641	5
GO:0051000	positive regulation of nitric-oxide synthase activity	0.000156442	0.00057889	5
GO:0051900	regulation of mitochondrial depolarization	0.000156442	0.00057889	5
GO:0071156	regulation of cell cycle arrest	0.000161386	0.000594752	5
GO:1904029	regulation of cyclin-dependent protein kinase activity	0.000161386	0.000594752	5
GO:0007204	positive regulation of cytosolic calcium ion concentration	0.000164161	0.000601575	5
GO:0001541	ovarian follicle development	0.000164567	0.000601575	5
GO:0072431	signal transduction involved in mitotic G1 DNA damage checkpoint	0.000164567	0.000601575	5
GO:1902400	intracellular signal transduction involved in G1 DNA damage checkpoint	0.000164567	0.000601575	5
GO:2000241	regulation of reproductive process	0.000173482	0.000632884	5
GO:0070371	ERK1 and ERK2 cascade	0.000174883	0.000636714	5
GO:0007566	embryo implantation	0.000176109	0.000638857	5
GO:0044774	mitotic DNA integrity checkpoint	0.000176178	0.000638857	5
GO:0007584	response to nutrient	0.0001791	0.000646222	5
GO:0035162	embryonic hemopoiesis	0.000179281	0.000646222	5
GO:1904996	positive regulation of leukocyte adhesion to vascular endothelial cell	0.000179281	0.000646222	5
GO:0050806	positive regulation of synaptic transmission	0.000184862	0.000665015	5
GO:0072413	signal transduction involved in mitotic cell cycle checkpoint	0.000188224	0.000673095	5
GO:1902402	signal transduction involved in mitotic DNA damage checkpoint	0.000188224	0.000673095	5

GO:1902403	signal transduction involved in mitotic DNA integrity checkpoint	0.000188224	0.000673095	5
GO:0001558	regulation of cell growth	0.0001928 1	0.000688135	5
GO:0001655	urogenital system development	0.000194035	0.000691143	5
GO:0050994	regulation of lipid catabolic process	0.000200929	0.000714294	5
GO:0043010	camera-type eye development	0.000202163	0.000717273	5
GO:0036003	positive regulation of transcription from RNA polymerase II promoter in response to stress	0.000204178	0.000717388	5
GO:0050995	negative regulation of lipid catabolic process	0.000204178	0.000717388	5
GO:0051882	mitochondrial depolarization	0.000204178	0.000717388	5
GO:0060444	branching involved in mammary gland duct morphogenesis	0.000204178	0.000717388	5
GO:1902993	positive regulation of amyloid precursor protein catabolic process	0.000204178	0.000717388	5
GO:0016241	regulation of macroautophagy	0.000209389	0.000734271	5
GO:1901991	negative regulation of mitotic cell cycle phase transition	0.0002123 9	0.000743354	5
GO:0006816	calcium ion transport	0.000214229	0.000748344	5
GO:0048771	tissue remodeling	0.000222579	0.000775764	5
GO:0031668	cellular response to extracellular stimulus	0.000222936	0.000775764	5
GO:0030888	regulation of B cell proliferation	0.000228177	0.000787088	5
GO:0070527	platelet aggregation	0.000228177	0.000787088	5
GO:2000351	regulation of endothelial cell apoptotic process	0.000228177	0.000787088	5
GO:2001244	positive regulation of intrinsic apoptotic signaling pathway	0.000228177	0.000787088	5
GO:2000027	regulation of animal organ morphogenesis	0.000228366	0.000787088	5
GO:0015874	norepinephrine transport	0.000231212	0.000795384	5
GO:0048640	negative regulation of developmental growth	0.000236238	0.000811131	5

GO:0050796	regulation of insulin secretion	0.000243584	0.000833191	5
GO:1905330	regulation of morphogenesis of an epithelium	0.000243584	0.000833191	5
GO:0031334	positive regulation of protein-containing complex assembly	0.000245297	0.00083747	5
GO:0030518	intracellular steroid hormone receptor signaling pathway	0.000245948	0.000838111	5
GO:0007596	blood coagulation	0.00025204	0.000855646	5
GO:0010721	negative regulation of cell development	0.00025204	0.000855646	5
GO:1904019	epithelial cell apoptotic process	0.000255959	0.000867326	5
GO:0002066	columnar/cuboidal epithelial cell development	0.000257988	0.000871155	5
GO:0006309	apoptotic DNA fragmentation	0.00026046	0.000871155	5
GO:0043951	negative regulation of cAMP-mediated signaling	0.00026046	0.000871155	5
GO:0060307	regulation of ventricular cardiac muscle cell membrane repolarization	0.00026046	0.000871155	5
GO:0060740	prostate gland epithelium morphogenesis	0.00026046	0.000871155	5
GO:1900739	regulation of protein insertion into mitochondrial membrane involved in apoptotic signaling pathway	0.00026046	0.000871155	5
GO:1900740	positive regulation of protein insertion into mitochondrial membrane involved in apoptotic signaling pathway	0.00026046	0.000871155	5
GO:0007611	learning or memory	0.000263228	0.000878789	5
GO:0033559	unsaturated fatty acid metabolic process	0.000266277	0.000887333	5
GO:0001894	tissue homeostasis	0.000269434	0.000896201	5
GO:0010573	vascular endothelial growth factor production	0.000273897	0.000907711	5
GO:0051205	protein insertion into membrane	0.000273897	0.000907711	5
GO:0007599	hemostasis	0.00027783	0.000919062	5
GO:0050817	coagulation	0.000283239	0.000935245	5
GO:0009651	response to salt stress	0.000291996	0.000960654	5

GO:1902175	regulation of oxidative stress-induced intrinsic apoptotic signaling pathway	0.000291996	0.000960654	5
GO:0002065	columnar/cuboidal epithelial cell differentiation	0.000299135	0.0009788	5
GO:0010906	regulation of glucose metabolic process	0.000299135	0.0009788	5
GO:0120254	olefinic compound metabolic process	0.000299135	0.0009788	5
GO:0050708	regulation of protein secretion	0.000299986	0.000979813	5
GO:0050918	positive chemotaxis	0.000307812	0.001003565	5
GO:1901990	regulation of mitotic cell cycle phase transition	0.000310479	0.001010438	5
GO:0043254	regulation of protein-containing complex assembly	0.000315589	0.001025225	5
GO:0002761	regulation of myeloid leukocyte differentiation	0.000322689	0.001038208	5
GO:0032642	regulation of chemokine production	0.000325853	0.001038208	5
GO:0072577	endothelial cell apoptotic process	0.000325853	0.001038208	5
GO:0002360	T cell lineage commitment	0.000325897	0.001038208	5
GO:0010460	positive regulation of heart rate	0.000325897	0.001038208	5
GO:0010575	positive regulation of vascular endothelial growth factor production	0.000325897	0.001038208	5
GO:0032800	receptor biosynthetic process	0.000325897	0.001038208	5
GO:0033598	mammary gland epithelial cell proliferation	0.000325897	0.001038208	5
GO:0060512	prostate gland morphogenesis	0.000325897	0.001038208	5
GO:0090200	positive regulation of release of cytochrome c from mitochondria	0.000325897	0.001038208	5
GO:1990776	response to angiotensin	0.000325897	0.001038208	5
GO:0051480	regulation of cytosolic calcium ion concentration	0.000329693	0.001048456	5
GO:0050804	modulation of chemical synaptic transmission	0.000342198	0.001084513	5
GO:0010948	negative regulation of cell cycle process	0.00034223	0.001084513	5

GO:00991 77	regulation of trans-synaptic signaling	0.0003477 36	0.0011000 34	5
GO:00157 21	bile acid and bile salt transport	0.0003622 35	0.0011438 99	5
GO:19054 75	regulation of protein localization to membrane	0.0003631 32	0.0011447 33	5
GO:19054 77	positive regulation of protein localization to membrane	0.0003739 89	0.0011769 09	5
GO:00356 90	cellular response to drug	0.0003845 16	0.0012058 43	5
GO:00429 82	amyloid precursor protein metabolic process	0.0003845 16	0.0012058 43	5
GO:00347 63	negative regulation of transmembrane transport	0.0003877 25	0.0012138 04	5
GO:00093 06	protein secretion	0.0003886 21	0.0012145 06	5
GO:00434 91	protein kinase B signaling	0.0003942 7	0.0012294 86	5
GO:00355 92	establishment of protein localization to extracellular region	0.0003947 73	0.0012294 86	5
GO:00463 94	carboxylic acid biosynthetic process	0.0003963 37	0.0012322 37	5
GO:00059 79	regulation of glycogen biosynthetic process	0.0004010 82	0.0012342 64	5
GO:00109 62	regulation of glucan biosynthetic process	0.0004010 82	0.0012342 64	5
GO:00341 10	regulation of homotypic cell-cell adhesion	0.0004010 82	0.0012342 64	5
GO:00434 57	regulation of cellular respiration	0.0004010 82	0.0012342 64	5
GO:00600 55	angiogenesis involved in wound healing	0.0004010 82	0.0012342 64	5
GO:00996 25	ventricular cardiac muscle cell membrane repolarization	0.0004010 82	0.0012342 64	5
GO:00160 53	organic acid biosynthetic process	0.0004035 65	0.0012397 97	5
GO:00059 77	glycogen metabolic process	0.0004275 88	0.0013113 72	5
GO:00716 92	protein localization to extracellular region	0.0004401 32	0.0013434 72	5
GO:00327 70	positive regulation of monooxygenase activity	0.0004425 09	0.0013434 72	5
GO:00457 37	positive regulation of cyclin-dependent protein serine/threonine kinase activity	0.0004425 09	0.0013434 72	5

GO:0045987	positive regulation of smooth muscle contraction	0.000442509	0.001343472	5
GO:0099623	regulation of cardiac muscle cell membrane repolarization	0.000442509	0.001343472	5
GO:1900181	negative regulation of protein localization to nucleus	0.000442509	0.001343472	5
GO:0001101	response to acid chemical	0.00044652	0.001352413	5
GO:0070838	divalent metal ion transport	0.000446949	0.001352413	5
GO:0006073	cellular glucan metabolic process	0.000450367	0.001358213	5
GO:0044042	glucan metabolic process	0.000450367	0.001358213	5
GO:0051346	negative regulation of hydrolase activity	0.000460845	0.0013875	5
GO:0031100	animal organ regeneration	0.000473999	0.001420014	5
GO:0032602	chemokine production	0.000473999	0.001420014	5
GO:0043627	response to estrogen	0.000473999	0.001420014	5
GO:0021700	developmental maturation	0.000477015	0.001426688	5
GO:0060603	mammary gland duct morphogenesis	0.000486587	0.001450522	5
GO:0098810	neurotransmitter reuptake	0.000486587	0.001450522	5
GO:0003158	endothelium development	0.00049485	0.001472726	5
GO:0072511	divalent inorganic cation transport	0.000497151	0.001477146	5
GO:0046890	regulation of lipid biosynthetic process	0.000498477	0.001478656	5
GO:0002791	regulation of peptide secretion	0.000507713	0.001503591	5
GO:0045766	positive regulation of angiogenesis	0.000511317	0.001511788	5
GO:0061418	regulation of transcription from RNA polymerase II promoter in response to hypoxia	0.000523892	0.001546442	5
GO:0040018	positive regulation of multicellular organism growth	0.000533384	0.001569339	5
GO:1902003	regulation of amyloid-beta formation	0.000533384	0.001569339	5

GO:0042110	T cell activation	0.000535781	0.001573833	5
GO:0061045	negative regulation of wound healing	0.00055019	0.001610747	5
GO:0072332	intrinsic apoptotic signaling pathway by p53 class mediator	0.00055019	0.001610747	5
GO:0070555	response to interleukin-1	0.00055139	0.001610747	5
GO:0050769	positive regulation of neurogenesis	0.000551908	0.001610747	5
GO:0030278	regulation of ossification	0.000565277	0.001647107	5
GO:0051348	negative regulation of transferase activity	0.000573085	0.001661594	5
GO:0006809	nitric oxide biosynthetic process	0.000577413	0.001661594	5
GO:0031016	pancreas development	0.000577413	0.001661594	5
GO:0071260	cellular response to mechanical stimulus	0.000577413	0.001661594	5
GO:0090398	cellular senescence	0.000577413	0.001661594	5
GO:0030073	insulin secretion	0.000579434	0.001661594	5
GO:0090276	regulation of peptide hormone secretion	0.000579434	0.001661594	5
GO:0014706	striated muscle tissue development	0.000581984	0.001661594	5
GO:0000737	DNA catabolic process, endonucleolytic	0.000582967	0.001661594	5
GO:0035633	maintenance of blood-brain barrier	0.000582967	0.001661594	5
GO:0043276	anoikis	0.000582967	0.001661594	5
GO:0098664	G protein-coupled serotonin receptor signaling pathway	0.000582967	0.001661594	5
GO:1902692	regulation of neuroblast proliferation	0.000582967	0.001661594	5
GO:0060048	cardiac muscle contraction	0.000584023	0.001661594	5
GO:0072655	establishment of protein localization to mitochondrion	0.000584023	0.001661594	5
GO:0048678	response to axon injury	0.00060558	0.001717526	5

GO:1900182	positive regulation of protein localization to nucleus	0.00060558	0.001717526	5
GO:0006869	lipid transport	0.00062229	0.001761064	5
GO:0007006	mitochondrial membrane organization	0.000622878	0.001761064	5
GO:0071158	positive regulation of cell cycle arrest	0.000634709	0.001788093	5
GO:0030262	apoptotic nuclear changes	0.000635402	0.001788093	5
GO:0060251	regulation of glial cell proliferation	0.000635402	0.001788093	5
GO:1902749	regulation of cell cycle G2/M phase transition	0.000638843	0.001794985	5
GO:0008286	insulin receptor signaling pathway	0.000643017	0.001799054	5
GO:0045598	regulation of fat cell differentiation	0.000643017	0.001799054	5
GO:0030198	extracellular matrix organization	0.000643274	0.001799054	5
GO:0090068	positive regulation of cell cycle process	0.00064527	0.00180185	5
GO:0043062	extracellular structure organization	0.000653979	0.001823355	5
GO:0070585	protein localization to mitochondrion	0.000663641	0.001847447	5
GO:0050670	regulation of lymphocyte proliferation	0.000670267	0.001863028	5
GO:1901653	cellular response to peptide	0.000675821	0.001875583	4
GO:0007612	learning	0.000684756	0.00189457	4
GO:0038127	ERBB signaling pathway	0.000684756	0.00189457	4
GO:0001893	maternal placenta development	0.000690756	0.001896669	4
GO:0010543	regulation of platelet activation	0.000690756	0.001896669	4
GO:0042554	superoxide anion generation	0.000690756	0.001896669	4
GO:0070873	regulation of glycogen metabolic process	0.000690756	0.001896669	4
GO:1905332	positive regulation of morphogenesis of an epithelium	0.000690756	0.001896669	4

GO:0048041	focal adhesion assembly	0.000695926	0.001907969	4
GO:0032944	regulation of mononuclear cell proliferation	0.000702878	0.001924113	4
GO:0019722	calcium-mediated signaling	0.000719637	0.001967016	4
GO:0046209	nitric oxide metabolic process	0.000728051	0.001985224	4
GO:1904064	positive regulation of cation transmembrane transport	0.000728493	0.001985224	4
GO:0006921	cellular component disassembly involved in execution phase of apoptosis	0.000749092	0.002022541	4
GO:0071542	dopaminergic neuron differentiation	0.000749092	0.002022541	4
GO:0086010	membrane depolarization during action potential	0.000749092	0.002022541	4
GO:0090050	positive regulation of cell migration involved in sprouting angiogenesis	0.000749092	0.002022541	4
GO:1904994	regulation of leukocyte adhesion to vascular endothelial cell	0.000749092	0.002022541	4
GO:2000273	positive regulation of signaling receptor activity	0.000749092	0.002022541	4
GO:0010675	regulation of cellular carbohydrate metabolic process	0.000751129	0.002022541	4
GO:0071901	negative regulation of protein serine/threonine kinase activity	0.000751129	0.002022541	4
GO:0031056	regulation of histone modification	0.000774287	0.002078711	4
GO:0043524	negative regulation of neuron apoptotic process	0.000774287	0.002078711	4
GO:2001020	regulation of response to DNA damage stimulus	0.000789793	0.0021172	4
GO:0006112	energy reserve metabolic process	0.000795425	0.002122862	4
GO:0009791	post-embryonic development	0.000795425	0.002122862	4
GO:0034109	homotypic cell-cell adhesion	0.000795425	0.002122862	4
GO:0060537	muscle tissue development	0.000806741	0.002140919	4
GO:0030224	monocyte differentiation	0.000810473	0.002140919	4
GO:0032094	response to food	0.000810473	0.002140919	4

GO:0032885	regulation of polysaccharide biosynthetic process	0.000810473	0.002140919	4
GO:0045777	positive regulation of blood pressure	0.000810473	0.002140919	4
GO:0060306	regulation of membrane repolarization	0.000810473	0.002140919	4
GO:1903131	mononuclear cell differentiation	0.000810473	0.002140919	4
GO:1904705	regulation of vascular associated smooth muscle cell proliferation	0.000830712	0.002184812	4
GO:1990874	vascular associated smooth muscle cell proliferation	0.000830712	0.002184812	4
GO:2001057	reactive nitrogen species metabolic process	0.000830712	0.002184812	4
GO:0046889	positive regulation of lipid biosynthetic process	0.000867089	0.002277177	4
GO:0008203	cholesterol metabolic process	0.000872289	0.002287514	4
GO:0034205	amyloid-beta formation	0.000874961	0.002287889	4
GO:0086091	regulation of heart rate by cardiac conduction	0.000874961	0.002287889	4
GO:0001649	osteoblast differentiation	0.000884799	0.002306947	4
GO:0048738	cardiac muscle tissue development	0.000884799	0.002306947	4
GO:0030595	leukocyte chemotaxis	0.000904815	0.002355739	4
GO:0042307	positive regulation of protein import into nucleus	0.000942618	0.002438113	4
GO:0042417	dopamine metabolic process	0.000942618	0.002438113	4
GO:1902991	regulation of amyloid precursor protein catabolic process	0.000942618	0.002438113	4
GO:0042058	regulation of epidermal growth factor receptor signaling pathway	0.000943191	0.002438113	4
GO:0070542	response to fatty acid	0.000943191	0.002438113	4
GO:0007050	cell cycle arrest	0.000945893	0.00244161	4
GO:0030902	hindbrain development	0.000951648	0.002452965	4
GO:1903039	positive regulation of leukocyte cell-cell adhesion	0.000966963	0.002485362	4

GO:1904018	positive regulation of vasculature development	0.000966963	0.002485362	4
GO:0033273	response to vitamin	0.000982952	0.002515737	4
GO:0034103	regulation of tissue remodeling	0.000982952	0.002515737	4
GO:2000177	regulation of neural precursor cell proliferation	0.000982952	0.002515737	4
GO:0060402	calcium ion transport into cytosol	0.001007459	0.002557748	4
GO:0032070	regulation of deoxyribonuclease activity	0.001009783	0.002557748	4
GO:0051901	positive regulation of mitochondrial depolarization	0.001009783	0.002557748	4
GO:0060068	vagina development	0.001009783	0.002557748	4
GO:0070391	response to lipoteichoic acid	0.001009783	0.002557748	4
GO:0071223	cellular response to lipoteichoic acid	0.001009783	0.002557748	4
GO:0090037	positive regulation of protein kinase C signaling	0.001009783	0.002557748	4
GO:0099622	cardiac muscle cell membrane repolarization	0.001013502	0.002557748	4
GO:1900117	regulation of execution phase of apoptosis	0.001013502	0.002557748	4
GO:1902042	negative regulation of extrinsic apoptotic signaling pathway via death domain receptors	0.001013502	0.002557748	4
GO:1900407	regulation of cellular response to oxidative stress	0.001023878	0.002580336	4
GO:1902107	positive regulation of leukocyte differentiation	0.00103626	0.002607906	4
GO:1903037	regulation of leukocyte cell-cell adhesion	0.001064454	0.002675141	4
GO:1903035	negative regulation of response to wounding	0.001065988	0.002675281	4
GO:0070663	regulation of leukocyte proliferation	0.001077797	0.00270117	4
GO:0001504	neurotransmitter uptake	0.001087673	0.002714643	4
GO:1904037	positive regulation of epithelial cell apoptotic process	0.001087673	0.002714643	4
GO:1904591	positive regulation of protein import	0.001087673	0.002714643	4

GO:0006814	sodium ion transport	0.001101089	0.002743937	4
GO:0032147	activation of protein kinase activity	0.001102443	0.002743937	4
GO:0008202	steroid metabolic process	0.001121834	0.002788364	4
GO:1902652	secondary alcohol metabolic process	0.001126338	0.00279572	4
GO:0006308	DNA catabolic process	0.001165189	0.002884239	4
GO:0045823	positive regulation of heart contraction	0.001165189	0.002884239	4
GO:0034329	cell junction assembly	0.001180932	0.002919215	4
GO:0001820	serotonin secretion	0.001230309	0.003000285	4
GO:0009629	response to gravity	0.001230309	0.003000285	4
GO:0019371	cyclooxygenase pathway	0.001230309	0.003000285	4
GO:0033327	Leydig cell differentiation	0.001230309	0.003000285	4
GO:0033483	gas homeostasis	0.001230309	0.003000285	4
GO:0040015	negative regulation of multicellular organism growth	0.001230309	0.003000285	4
GO:0045899	positive regulation of RNA polymerase II transcription preinitiation complex assembly	0.001230309	0.003000285	4
GO:0060525	prostate glandular acinus development	0.001230309	0.003000285	4
GO:1903800	positive regulation of production of miRNAs involved in gene silencing by miRNA	0.001230309	0.003000285	4
GO:2001269	positive regulation of cysteine-type endopeptidase activity involved in apoptotic signaling pathway	0.001230309	0.003000285	4
GO:0032881	regulation of polysaccharide metabolic process	0.001246106	0.00301168	4
GO:0033173	calcineurin-NFAT signaling cascade	0.001246106	0.00301168	4
GO:0030516	regulation of axon extension	0.001246632	0.00301168	4
GO:0071674	mononuclear cell migration	0.001246632	0.00301168	4
GO:0120162	positive regulation of cold-induced thermogenesis	0.001246632	0.00301168	4

GO:1901184	regulation of ERBB signaling pathway	0.001246632	0.00301168	4
GO:1901655	cellular response to ketone	0.001246632	0.00301168	4
GO:0007093	mitotic cell cycle checkpoint	0.001255321	0.003028627	4
GO:0045833	negative regulation of lipid metabolic process	0.001294937	0.003115897	4
GO:0048010	vascular endothelial growth factor receptor signaling pathway	0.001294937	0.003115897	4
GO:0045931	positive regulation of mitotic cell cycle	0.001323762	0.003176079	4
GO:0031018	endocrine pancreas development	0.00133048	0.003176079	4
GO:0032722	positive regulation of chemokine production	0.00133048	0.003176079	4
GO:0042771	intrinsic apoptotic signaling pathway in response to DNA damage by p53 class mediator	0.00133048	0.003176079	4
GO:0045429	positive regulation of nitric oxide biosynthetic process	0.00133048	0.003176079	4
GO:1904646	cellular response to amyloid-beta	0.00133048	0.003176079	4
GO:0016125	sterol metabolic process	0.001358999	0.003239885	4
GO:0001933	negative regulation of protein phosphorylation	0.001364791	0.003249412	4
GO:0043122	regulation of I-kappaB kinase/NF-kappaB signaling	0.001383649	0.003289982	4
GO:0051924	regulation of calcium ion transport	0.001411844	0.003350514	4
GO:0005978	glycogen biosynthetic process	0.001418365	0.003350514	4
GO:0009250	glucan biosynthetic process	0.001418365	0.003350514	4
GO:0061028	establishment of endothelial barrier	0.001418365	0.003350514	4
GO:1904407	positive regulation of nitric oxide metabolic process	0.001418365	0.003350514	4
GO:0001819	positive regulation of cytokine production	0.001424184	0.0033555	4
GO:0043434	response to peptide hormone	0.001424184	0.0033555	4
GO:0030856	regulation of epithelial cell differentiation	0.001431545	0.003364081	4

GO:1905952	regulation of lipid localization	0.001431545	0.003364081	4
GO:0032637	interleukin-8 production	0.001447692	0.003370985	4
GO:0044264	cellular polysaccharide metabolic process	0.001447692	0.003370985	4
GO:1902882	regulation of response to oxidative stress	0.001447692	0.003370985	4
GO:0002024	diet induced thermogenesis	0.001471742	0.003370985	4
GO:0006983	ER overload response	0.001471742	0.003370985	4
GO:0007077	mitotic nuclear envelope disassembly	0.001471742	0.003370985	4
GO:0032494	response to peptidoglycan	0.001471742	0.003370985	4
GO:0045760	positive regulation of action potential	0.001471742	0.003370985	4
GO:0046541	saliva secretion	0.001471742	0.003370985	4
GO:0051583	dopamine uptake involved in synaptic transmission	0.001471742	0.003370985	4
GO:0051934	catecholamine uptake involved in synaptic transmission	0.001471742	0.003370985	4
GO:0060442	branching involved in prostate gland morphogenesis	0.001471742	0.003370985	4
GO:0060736	prostate gland growth	0.001471742	0.003370985	4
GO:0060742	epithelial cell differentiation involved in prostate gland development	0.001471742	0.003370985	4
GO:0070243	regulation of thymocyte apoptotic process	0.001471742	0.003370985	4
GO:0090154	positive regulation of sphingolipid biosynthetic process	0.001471742	0.003370985	4
GO:0090331	negative regulation of platelet aggregation	0.001471742	0.003370985	4
GO:1902510	regulation of apoptotic DNA fragmentation	0.001471742	0.003370985	4
GO:1904181	positive regulation of membrane depolarization	0.001471742	0.003370985	4
GO:2000304	positive regulation of ceramide biosynthetic process	0.001471742	0.003370985	4
GO:0008016	regulation of heart contraction	0.001499071	0.003427818	4

GO:00226<br>00	digestive system process	0.0015012<br>85	0.0034278<br>18	4
GO:00215<br>43	pallium development	0.0015069<br>09	0.0034278<br>18	4
GO:00226<br>02	ovulation cycle process	0.0015098<br>15	0.0034278<br>18	4
GO:00459<br>33	positive regulation of muscle contraction	0.0015098<br>15	0.0034278<br>18	4
GO:00485<br>99	oocyte development	0.0015098<br>15	0.0034278<br>18	4
GO:00488<br>06	genitalia development	0.0015098<br>15	0.0034278<br>18	4
GO:00300<br>72	peptide hormone secretion	0.0015290<br>42	0.0034671<br>19	4
GO:00032<br>05	cardiac chamber development	0.0015456<br>69	0.0034960<br>6	4
GO:00157<br>18	monocarboxylic acid transport	0.0015456<br>69	0.0034960<br>6	4
GO:00020<br>42	cell migration involved in sprouting angiogenesis	0.0015562<br>46	0.0035024<br>7	4
GO:00347<br>66	negative regulation of ion transmembrane transport	0.0015562<br>46	0.0035024<br>7	4
GO:00600<br>79	excitatory postsynaptic potential	0.0015562<br>46	0.0035024<br>7	4
GO:00988<br>69	cellular oxidant detoxification	0.0015562<br>46	0.0035024<br>7	4
GO:00030<br>07	heart morphogenesis	0.0015594<br>68	0.0035053<br>61	4
GO:00215<br>37	telencephalon development	0.0015903<br>53	0.0035703<br>49	4
GO:00422<br>20	response to cocaine	0.0016048<br>83	0.0035985<br>06	4
GO:00068<br>74	cellular calcium ion homeostasis	0.0016148<br>17	0.0036162<br>98	4
GO:00020<br>67	glandular epithelial cell differentiation	0.0017036<br>21	0.0037870<br>49	4
GO:00026<br>86	negative regulation of leukocyte migration	0.0017036<br>21	0.0037870<br>49	4
GO:00308<br>50	prostate gland development	0.0017036<br>21	0.0037870<br>49	4
GO:00485<br>12	circadian behavior	0.0017036<br>21	0.0037870<br>49	4
GO:00860<br>09	membrane repolarization	0.0017036<br>21	0.0037870<br>49	4

GO:0097720	calcineurin-mediated signaling	0.001703621	0.003787049	4
GO:0006941	striated muscle contraction	0.001708087	0.003787669	4
GO:0060401	cytosolic calcium ion transport	0.001708087	0.003787669	4
GO:0032414	positive regulation of ion transmembrane transporter activity	0.00172952	0.003830503	4
GO:0042308	negative regulation of protein import into nucleus	0.00173388	0.003830782	4
GO:1904590	negative regulation of protein import	0.00173388	0.003830782	4
GO:0031589	cell-substrate adhesion	0.001755147	0.003873039	4
GO:0031330	negative regulation of cellular catabolic process	0.001785571	0.003932674	4
GO:0030330	DNA damage response, signal transduction by p53 class mediator	0.001790136	0.003932674	4
GO:0055017	cardiac muscle tissue growth	0.001790136	0.003932674	4
GO:1901796	regulation of signal transduction by p53 class mediator	0.001793839	0.003932674	4
GO:0001774	microglial cell activation	0.001806079	0.003932674	4
GO:0002269	leukocyte activation involved in inflammatory response	0.001806079	0.003932674	4
GO:0007622	rhythmic behavior	0.001806079	0.003932674	4
GO:0014009	glial cell proliferation	0.001806079	0.003932674	4
GO:0030195	negative regulation of blood coagulation	0.001806079	0.003932674	4
GO:0042987	amyloid precursor protein catabolic process	0.001806079	0.003932674	4
GO:1904707	positive regulation of vascular associated smooth muscle cell proliferation	0.001806079	0.003932674	4
GO:0008361	regulation of cell size	0.00183788	0.003997111	4
GO:0046883	regulation of hormone secretion	0.001854528	0.004028475	4
GO:0071346	cellular response to interferon-gamma	0.00188271	0.004084789	4
GO:0007159	leukocyte cell-cell adhesion	0.001898071	0.004107414	4

GO:0016570	histone modification	0.001899948	0.004107414	4
GO:0055074	calcium ion homeostasis	0.001899948	0.004107414	4
GO:0050435	amyloid-beta metabolic process	0.001912307	0.004121908	4
GO:1900047	negative regulation of hemostasis	0.001912307	0.004121908	4
GO:0018958	phenol-containing compound metabolic process	0.001915764	0.004121908	4
GO:0021761	limbic system development	0.001915764	0.004121908	4
GO:0090596	sensory organ morphogenesis	0.001925489	0.004134209	4
GO:0051052	regulation of DNA metabolic process	0.001927711	0.004134209	4
GO:0071347	cellular response to interleukin-1	0.001928336	0.004134209	4
GO:0045898	regulation of RNA polymerase II transcription preinitiation complex assembly	0.002016522	0.00429003	4
GO:0050930	induction of positive chemotaxis	0.002016522	0.00429003	4
GO:0086069	bundle of His cell to Purkinje myocyte communication	0.002016522	0.00429003	4
GO:0106070	regulation of adenylate cyclase-activating G protein-coupled receptor signaling pathway	0.002016522	0.00429003	4
GO:1903624	regulation of DNA catabolic process	0.002016522	0.00429003	4
GO:2001257	regulation of cation channel activity	0.00202201	0.00429003	4
GO:0009994	oocyte differentiation	0.002022353	0.00429003	4
GO:0031103	axon regeneration	0.002022353	0.00429003	4
GO:0050999	regulation of nitric-oxide synthase activity	0.002022353	0.00429003	4
GO:0034440	lipid oxidation	0.002047374	0.004322835	4
GO:0042303	molting cycle	0.002047374	0.004322835	4
GO:0042633	hair cycle	0.002047374	0.004322835	4
GO:0061387	regulation of extent of cell growth	0.002047374	0.004322835	4

GO:0044839	cell cycle G2/M phase transition	0.002073573	0.004373048	4
GO:0005976	polysaccharide metabolic process	0.002115468	0.004451027	4
GO:0099565	chemical synaptic transmission, postsynaptic	0.002115468	0.004451027	4
GO:0045333	cellular respiration	0.002118972	0.004453221	4
GO:0032731	positive regulation of interleukin-1 beta production	0.002136266	0.004473979	4
GO:0045744	negative regulation of G protein-coupled receptor signaling pathway	0.002136266	0.004473979	4
GO:0061756	leukocyte adhesion to vascular endothelial cell	0.002136266	0.004473979	4
GO:0043123	positive regulation of I-kappaB kinase/NF-kappaB signaling	0.002168706	0.004536667	4
GO:0032963	collagen metabolic process	0.002185111	0.004565705	4
GO:0022412	cellular process involved in reproduction in multicellular organism	0.00224432	0.004671343	4
GO:0016569	covalent chromatin modification	0.002252358	0.004671343	4
GO:0006584	catecholamine metabolic process	0.002254093	0.004671343	4
GO:0009712	catechol-containing compound metabolic process	0.002254093	0.004671343	4
GO:0032757	positive regulation of interleukin-8 production	0.002254093	0.004671343	4
GO:0022037	metencephalon development	0.002256321	0.004671343	4
GO:0043200	response to amino acid	0.002256321	0.004671343	4
GO:1990748	cellular detoxification	0.002256321	0.004671343	4
GO:0045165	cell fate commitment	0.002270621	0.004690432	4
GO:0030308	negative regulation of cell growth	0.002270726	0.004690432	4
GO:0022409	positive regulation of cell-cell adhesion	0.002311671	0.004731701	4
GO:0006089	lactate metabolic process	0.00231947	0.004731701	4
GO:0034111	negative regulation of homotypic cell-cell adhesion	0.00231947	0.004731701	4

GO:00448<br>49	estrous cycle	0.0023194<br>7	0.0047317<br>01	4
GO:00456<br>51	positive regulation of macrophage differentiation	0.0023194<br>7	0.0047317<br>01	4
GO:00480<br>70	regulation of developmental pigmentation	0.0023194<br>7	0.0047317<br>01	4
GO:00485<br>69	post-embryonic animal organ development	0.0023194<br>7	0.0047317<br>01	4
GO:00510<br>44	positive regulation of membrane protein ectodomain proteolysis	0.0023194<br>7	0.0047317<br>01	4
GO:19015<br>50	regulation of endothelial cell development	0.0023194<br>7	0.0047317<br>01	4
GO:19031<br>40	regulation of establishment of endothelial barrier	0.0023194<br>7	0.0047317<br>01	4
GO:20010<br>28	positive regulation of endothelial cell chemotaxis	0.0023194<br>7	0.0047317<br>01	4
GO:00224<br>08	negative regulation of cell-cell adhesion	0.0023230<br>28	0.0047336<br>23	4
GO:00604<br>19	heart growth	0.0023291<br>17	0.0047406<br>93	4
GO:00423<br>26	negative regulation of phosphorylation	0.0023405<br>63	0.0047586<br>38	4
GO:00019<br>54	positive regulation of cell-matrix adhesion	0.0023758<br>79	0.0048088<br>26	4
GO:00029<br>31	response to ischemia	0.0023758<br>79	0.0048088<br>26	4
GO:00508<br>19	negative regulation of coagulation	0.0023758<br>79	0.0048088<br>26	4
GO:00513<br>53	positive regulation of oxidoreductase activity	0.0023758<br>79	0.0048088<br>26	4
GO:00466<br>20	regulation of organ growth	0.0024035<br>16	0.0048539<br>05	4
GO:00609<br>64	regulation of gene silencing by miRNA	0.0024035<br>16	0.0048539<br>05	4
GO:00454<br>46	endothelial cell differentiation	0.0024795<br>36	0.0050002<br>29	4
GO:00328<br>68	response to insulin	0.0024814<br>98	0.0050002<br>29	4
GO:00702<br>28	regulation of lymphocyte apoptotic process	0.0025016<br>69	0.0050296<br>72	4
GO:00860<br>02	cardiac muscle cell action potential involved in contraction	0.0025016<br>69	0.0050296<br>72	4
GO:00420<br>98	T cell proliferation	0.0025410<br>48	0.0050918<br>7	4

GO:0050864	regulation of B cell activation	0.002541048	0.00509187	4
GO:1902275	regulation of chromatin organization	0.002541048	0.00509187	4
GO:0006805	xenobiotic metabolic process	0.002557194	0.005112901	4
GO:0021987	cerebral cortex development	0.002557194	0.005112901	4
GO:0072503	cellular divalent inorganic cation homeostasis	0.002589121	0.005171023	4
GO:0006066	alcohol metabolic process	0.002599778	0.005186091	4
GO:0007249	I-kappaB kinase/NF-kappaB signaling	0.002614913	0.005186091	4
GO:0030520	intracellular estrogen receptor signaling pathway	0.002631508	0.005186091	4
GO:0042743	hydrogen peroxide metabolic process	0.002631508	0.005186091	4
GO:0048016	inositol phosphate-mediated signaling	0.002631508	0.005186091	4
GO:0098900	regulation of action potential	0.002631508	0.005186091	4
GO:0030397	membrane disassembly	0.002642523	0.005186091	4
GO:0032225	regulation of synaptic transmission, dopaminergic	0.002642523	0.005186091	4
GO:0043116	negative regulation of vascular permeability	0.002642523	0.005186091	4
GO:0045725	positive regulation of glycogen biosynthetic process	0.002642523	0.005186091	3
GO:0051081	nuclear envelope disassembly	0.002642523	0.005186091	3
GO:0051770	positive regulation of nitric-oxide synthase biosynthetic process	0.002642523	0.005186091	3
GO:0060576	intestinal epithelial cell development	0.002642523	0.005186091	3
GO:0071391	cellular response to estrogen stimulus	0.002642523	0.005186091	3
GO:1900451	positive regulation of glutamate receptor signaling pathway	0.002642523	0.005186091	3
GO:1903729	regulation of plasma membrane organization	0.002642523	0.005186091	3
GO:0060047	heart contraction	0.002660563	0.005215837	3

GO:0043112	receptor metabolic process	0.002714055	0.005310192	3
GO:0060147	regulation of posttranscriptional gene silencing	0.002717496	0.005310192	3
GO:0060966	regulation of gene silencing by RNA	0.002717496	0.005310192	3
GO:1903749	positive regulation of establishment of protein localization to mitochondrion	0.002765439	0.005398047	3
GO:1903706	regulation of hemopoiesis	0.002788784	0.00543775	3
GO:0030218	erythrocyte differentiation	0.002800175	0.005454083	3
GO:0030900	forebrain development	0.002832977	0.00551204	3
GO:0010811	positive regulation of cell-substrate adhesion	0.002884563	0.005600365	3
GO:0097237	cellular response to toxic substance	0.002884563	0.005600365	3
GO:0006636	unsaturated fatty acid biosynthetic process	0.002903504	0.005619053	3
GO:0031102	neuron projection regeneration	0.002903504	0.005619053	3
GO:0048008	platelet-derived growth factor receptor signaling pathway	0.002903504	0.005619053	3
GO:0034341	response to interferon-gamma	0.002957828	0.005668584	3
GO:0007173	epidermal growth factor receptor signaling pathway	0.002970676	0.005668584	3
GO:0048675	axon extension	0.002970676	0.005668584	3
GO:0071466	cellular response to xenobiotic stimulus	0.002970676	0.005668584	3
GO:0006978	DNA damage response, signal transduction by p53 class mediator resulting in transcription of p21 class mediator	0.002985487	0.005668584	3
GO:0030540	female genitalia development	0.002985487	0.005668584	3
GO:0031065	positive regulation of histone deacetylation	0.002985487	0.005668584	3
GO:0033599	regulation of mammary gland epithelial cell proliferation	0.002985487	0.005668584	3
GO:0043117	positive regulation of vascular permeability	0.002985487	0.005668584	3
GO:0045986	negative regulation of smooth muscle contraction	0.002985487	0.005668584	3

GO:00506 65	hydrogen peroxide biosynthetic process	0.0029854 87	0.0056685 84	3
GO:00550 12	ventricular cardiac muscle cell differentiation	0.0029854 87	0.0056685 84	3
GO:00606 44	mammary gland epithelial cell differentiation	0.0029854 87	0.0056685 84	3
GO:00708 75	positive regulation of glycogen metabolic process	0.0029854 87	0.0056685 84	3
GO:00718 50	mitotic cell cycle arrest	0.0029854 87	0.0056685 84	3
GO:00903 36	positive regulation of brown fat cell differentiation	0.0029854 87	0.0056685 84	3
GO:00904 94	dopamine uptake	0.0029854 87	0.0056685 84	3
GO:20008 11	negative regulation of anoikis	0.0029854 87	0.0056685 84	3
GO:00442 62	cellular carbohydrate metabolic process	0.0029970 93	0.0056846 55	3
GO:00327 32	positive regulation of interleukin-1 production	0.0030457 45	0.0057504 18	3
GO:00619 00	glial cell activation	0.0030457 45	0.0057504 18	3
GO:00977 55	positive regulation of blood vessel diameter	0.0030457 45	0.0057504 18	3
GO:19034 28	positive regulation of reactive oxygen species biosynthetic process	0.0030457 45	0.0057504 18	3
GO:00507 68	negative regulation of neurogenesis	0.0030476 54	0.0057504 18	3
GO:00421 76	regulation of protein catabolic process	0.0030819 46	0.0058090 63	3
GO:00309 68	endoplasmic reticulum unfolded protein response	0.0031481 49	0.0059078 69	3
GO:00352 70	endocrine system development	0.0031481 49	0.0059078 69	3
GO:00485 93	camera-type eye morphogenesis	0.0031481 49	0.0059078 69	3
GO:19035 78	regulation of ATP metabolic process	0.0031481 49	0.0059078 69	3
GO:00030 15	heart process	0.0031506 92	0.0059078 69	3
GO:00105 74	regulation of vascular endothelial growth factor production	0.0031922 02	0.0059548 52	3
GO:00330 13	tetrapyrrole metabolic process	0.0031922 02	0.0059548 52	3

GO:0033619	membrane protein proteolysis	0.003192202	0.005954852	3
GO:0046324	regulation of glucose import	0.003192202	0.005954852	3
GO:1902041	regulation of extrinsic apoptotic signaling pathway via death domain receptors	0.003192202	0.005954852	3
GO:0055007	cardiac muscle cell differentiation	0.003239544	0.00603694	3
GO:0019079	viral genome replication	0.003332732	0.006182058	3
GO:0007405	neuroblast proliferation	0.003342916	0.006182058	3
GO:0042772	DNA damage response, signal transduction resulting in transcription	0.003348165	0.006182058	3
GO:0051023	regulation of immunoglobulin secretion	0.003348165	0.006182058	3
GO:0070230	positive regulation of lymphocyte apoptotic process	0.003348165	0.006182058	3
GO:0090036	regulation of protein kinase C signaling	0.003348165	0.006182058	3
GO:0090493	catecholamine uptake	0.003348165	0.006182058	3
GO:0150078	positive regulation of neuroinflammatory response	0.003348165	0.006182058	3
GO:2000303	regulation of ceramide biosynthetic process	0.003348165	0.006182058	3
GO:0007162	negative regulation of cell adhesion	0.003364562	0.006206	3
GO:0014013	regulation of gliogenesis	0.003427733	0.006316082	3
GO:0046622	positive regulation of organ growth	0.003497924	0.006419271	3
GO:0051893	regulation of focal adhesion assembly	0.003497924	0.006419271	3
GO:0070059	intrinsic apoptotic signaling pathway in response to endoplasmic reticulum stress	0.003497924	0.006419271	3
GO:0090109	regulation of cell-substrate junction assembly	0.003497924	0.006419271	3
GO:0034101	erythrocyte homeostasis	0.003524561	0.006461602	3
GO:0009410	response to xenobiotic stimulus	0.003623235	0.006635778	3
GO:0001885	endothelial cell development	0.003657265	0.006664375	3

GO:0045453	bone resorption	0.003657265	0.006664375	3
GO:0051926	negative regulation of calcium ion transport	0.003657265	0.006664375	3
GO:1900449	regulation of glutamate receptor signaling pathway	0.003657265	0.006664375	3
GO:2000401	regulation of lymphocyte migration	0.003657265	0.006664375	3
GO:0042063	gliogenesis	0.003705457	0.006709738	3
GO:0003206	cardiac chamber morphogenesis	0.003723771	0.006709738	3
GO:0003231	cardiac ventricle development	0.003723771	0.006709738	3
GO:0010508	positive regulation of autophagy	0.003723771	0.006709738	3
GO:0010544	negative regulation of platelet activation	0.003730364	0.006709738	3
GO:0042053	regulation of dopamine metabolic process	0.003730364	0.006709738	3
GO:0042069	regulation of catecholamine metabolic process	0.003730364	0.006709738	3
GO:0060252	positive regulation of glial cell proliferation	0.003730364	0.006709738	3
GO:0070989	oxidative demethylation	0.003730364	0.006709738	3
GO:0090153	regulation of sphingolipid biosynthetic process	0.003730364	0.006709738	3
GO:1902176	negative regulation of oxidative stress-induced intrinsic apoptotic signaling pathway	0.003730364	0.006709738	3
GO:1905038	regulation of membrane lipid metabolic process	0.003730364	0.006709738	3
GO:2000010	positive regulation of protein localization to cell surface	0.003730364	0.006709738	3
GO:1905953	negative regulation of lipid localization	0.003820976	0.006865894	3
GO:0016236	macroautophagy	0.003885239	0.006974443	3
GO:0032768	regulation of monooxygenase activity	0.003989094	0.00713257	3
GO:0046626	regulation of insulin receptor signaling pathway	0.003989094	0.00713257	3
GO:0150116	regulation of cell-substrate junction organization	0.003989094	0.00713257	3

GO:2000378	negative regulation of reactive oxygen species metabolic process	0.003989094	0.00713257	3
GO:0034754	cellular hormone metabolic process	0.004036714	0.007210591	3
GO:0007252	I-kappaB phosphorylation	0.004131892	0.007335352	3
GO:0019373	epoxygenase P450 pathway	0.004131892	0.007335352	3
GO:0032495	response to muramyl dipeptide	0.004131892	0.007335352	3
GO:0051767	nitric-oxide synthase biosynthetic process	0.004131892	0.007335352	3
GO:0051769	regulation of nitric-oxide synthase biosynthetic process	0.004131892	0.007335352	3
GO:1900409	positive regulation of cellular response to oxidative stress	0.004131892	0.007335352	3
GO:0046879	hormone secretion	0.004134937	0.007335352	3
GO:1903672	positive regulation of sprouting angiogenesis	0.004161654	0.007375518	3
GO:0045727	positive regulation of translation	0.004254956	0.007526129	3
GO:0050671	positive regulation of lymphocyte proliferation	0.004254956	0.007526129	3
GO:0031669	cellular response to nutrient levels	0.004336358	0.007636914	3
GO:0042445	hormone metabolic process	0.004336358	0.007636914	3
GO:0016239	positive regulation of macroautophagy	0.004338691	0.007636914	3
GO:0045428	regulation of nitric oxide biosynthetic process	0.004338691	0.007636914	3
GO:0071479	cellular response to ionizing radiation	0.004338691	0.007636914	3
GO:0030183	B cell differentiation	0.004367009	0.007671832	3
GO:0032946	positive regulation of mononuclear cell proliferation	0.004367009	0.007671832	3
GO:0097529	myeloid leukocyte migration	0.004419693	0.007756855	3
GO:0051961	negative regulation of nervous system development	0.004463566	0.007826264	3
GO:0001508	action potential	0.004481038	0.007841703	3

GO:0098754	detoxification	0.004481038	0.007841703	3
GO:0060038	cardiac muscle cell proliferation	0.004520239	0.007875459	3
GO:0002363	alpha-beta T cell lineage commitment	0.004552556	0.007875459	3
GO:0006837	serotonin transport	0.004552556	0.007875459	3
GO:0021854	hypothalamus development	0.004552556	0.007875459	3
GO:0030220	platelet formation	0.004552556	0.007875459	3
GO:0030728	ovulation	0.004552556	0.007875459	3
GO:0036499	PERK-mediated unfolded protein response	0.004552556	0.007875459	3
GO:0046823	negative regulation of nucleocytoplasmic transport	0.004552556	0.007875459	3
GO:0046827	positive regulation of protein export from nucleus	0.004552556	0.007875459	3
GO:0048305	immunoglobulin secretion	0.004552556	0.007875459	3
GO:0090312	positive regulation of protein deacetylation	0.004552556	0.007875459	3
GO:0140467	integrated stress response signaling	0.004552556	0.007875459	3
GO:0006879	cellular iron ion homeostasis	0.004706331	0.008133698	3
GO:0060968	regulation of gene silencing	0.004715091	0.008141062	3
GO:0009914	hormone transport	0.004740072	0.008176392	3
GO:0032869	cellular response to insulin stimulus	0.004764492	0.008210688	3
GO:0001889	liver development	0.004835146	0.008324518	3
GO:0033692	cellular polysaccharide biosynthetic process	0.004896999	0.008422995	3
GO:0032102	negative regulation of response to external stimulus	0.004946129	0.00849942	3
GO:0006925	inflammatory cell apoptotic process	0.004992167	0.00851385	3
GO:0009235	cobalamin metabolic process	0.004992167	0.00851385	3

GO:0010888	negative regulation of lipid storage	0.004992167	0.00851385	3
GO:0032069	regulation of nuclease activity	0.004992167	0.00851385	3
GO:0036344	platelet morphogenesis	0.004992167	0.00851385	3
GO:0043369	CD4-positive or CD8-positive, alpha-beta T cell lineage commitment	0.004992167	0.00851385	3
GO:1902884	positive regulation of response to oxidative stress	0.004992167	0.00851385	3
GO:2000479	regulation of cAMP-dependent protein kinase activity	0.004992167	0.00851385	3
GO:0006836	neurotransmitter transport	0.005035361	0.008571358	3
GO:0048762	mesenchymal cell differentiation	0.005035361	0.008571358	3
GO:0045600	positive regulation of fat cell differentiation	0.005092275	0.008651961	3
GO:0051937	catecholamine transport	0.005092275	0.008651961	3
GO:0061008	hepaticobiliary system development	0.005207612	0.008823069	3
GO:0106106	cold-induced thermogenesis	0.005207612	0.008823069	3
GO:0120161	regulation of cold-induced thermogenesis	0.005207612	0.008823069	3
GO:0051966	regulation of synaptic transmission, glutamatergic	0.005292191	0.008949607	3
GO:1903747	regulation of establishment of protein localization to mitochondrion	0.005292191	0.008949607	3
GO:0002052	positive regulation of neuroblast proliferation	0.005450537	0.009157478	3
GO:0031281	positive regulation of cyclase activity	0.005450537	0.009157478	3
GO:0045649	regulation of macrophage differentiation	0.005450537	0.009157478	3
GO:0051043	regulation of membrane protein ectodomain proteolysis	0.005450537	0.009157478	3
GO:0086012	membrane depolarization during cardiac muscle cell action potential	0.005450537	0.009157478	3
GO:1900017	positive regulation of cytokine production involved in inflammatory response	0.005450537	0.009157478	3
GO:2000178	negative regulation of neural precursor cell proliferation	0.005450537	0.009157478	3

GO:0034620	cellular response to unfolded protein	0.00546633	0.009175492	3
GO:0021536	diencephalon development	0.005496775	0.009200989	3
GO:0086003	cardiac muscle cell contraction	0.005496775	0.009200989	3
GO:2001259	positive regulation of cation channel activity	0.005496775	0.009200989	3
GO:0051592	response to calcium ion	0.005598857	0.009363202	3
GO:0046323	glucose import	0.005706057	0.009504583	3
GO:0050805	negative regulation of synaptic transmission	0.005706057	0.009504583	3
GO:0086001	cardiac muscle cell action potential	0.005706057	0.009504583	3
GO:1900076	regulation of cellular response to insulin stimulus	0.005706057	0.009504583	3
GO:0060560	developmental growth involved in morphogenesis	0.005709661	0.009504583	3
GO:0008277	regulation of G protein-coupled receptor signaling pathway	0.005733516	0.00953552	3
GO:0046777	protein autophosphorylation	0.005810943	0.009646558	3
GO:0048588	developmental cell growth	0.005810943	0.009646558	3
GO:0033555	multicellular organismal response to stress	0.005920066	0.009759434	3
GO:0072091	regulation of stem cell proliferation	0.005920066	0.009759434	3
GO:1901616	organic hydroxy compound catabolic process	0.005920066	0.009759434	3
GO:0010640	regulation of platelet-derived growth factor receptor signaling pathway	0.005927479	0.009759434	3
GO:0032469	endoplasmic reticulum calcium ion homeostasis	0.005927479	0.009759434	3
GO:0042738	exogenous drug catabolic process	0.005927479	0.009759434	3
GO:0090335	regulation of brown fat cell differentiation	0.005927479	0.009759434	3
GO:2000209	regulation of anoikis	0.005927479	0.009759434	3
GO:2000637	positive regulation of gene silencing by miRNA	0.005927479	0.009759434	3

GO:0070665	positive regulation of leukocyte proliferation	0.006009291	0.00988514	3
GO:0014015	positive regulation of gliogenesis	0.00613883	0.010079901	3
GO:1903201	regulation of oxidative stress-induced cell death	0.00613883	0.010079901	3
GO:0051224	negative regulation of protein transport	0.006150436	0.010089803	3
GO:0038093	Fc receptor signaling pathway	0.006228784	0.010209076	3
GO:0000271	polysaccharide biosynthetic process	0.006362376	0.010409178	3
GO:0043900	regulation of multi-organism process	0.006362376	0.010409178	3
GO:0010738	regulation of protein kinase A signaling	0.006422807	0.010432585	3
GO:0051894	positive regulation of focal adhesion assembly	0.006422807	0.010432585	3
GO:0060148	positive regulation of posttranscriptional gene silencing	0.006422807	0.010432585	3
GO:0060571	morphogenesis of an epithelial fold	0.006422807	0.010432585	3
GO:0060575	intestinal epithelial cell differentiation	0.006422807	0.010432585	3
GO:0060669	embryonic placenta morphogenesis	0.006422807	0.010432585	3
GO:1904385	cellular response to angiotensin	0.006422807	0.010432585	3
GO:2001026	regulation of endothelial cell chemotaxis	0.006422807	0.010432585	3
GO:0045834	positive regulation of lipid metabolic process	0.006439316	0.010450021	3
GO:0007265	Ras protein signal transduction	0.006581534	0.010647992	3
GO:0061351	neural precursor cell proliferation	0.00658708	0.010647992	3
GO:0010827	regulation of glucose transmembrane transport	0.006590729	0.010647992	3
GO:0042246	tissue regeneration	0.006590729	0.010647992	3
GO:0099601	regulation of neurotransmitter receptor activity	0.006590729	0.010647992	3
GO:1904950	negative regulation of establishment of protein localization	0.006737079	0.010865033	3

GO:1990845	adaptive thermogenesis	0.006737079	0.010865033	3
GO:0006469	negative regulation of protein kinase activity	0.006780305	0.010925008	3
GO:0003151	outflow tract morphogenesis	0.006823917	0.010975732	3
GO:1901224	positive regulation of NIK/NF-kappaB signaling	0.006823917	0.010975732	3
GO:0009615	response to virus	0.006855311	0.011016434	3
GO:0008360	regulation of cell shape	0.006889327	0.011061275	3
GO:0002068	glandular epithelial cell development	0.006936336	0.011077773	3
GO:0018904	ether metabolic process	0.006936336	0.011077773	3
GO:0019430	removal of superoxide radicals	0.006936336	0.011077773	3
GO:0035902	response to immobilization stress	0.006936336	0.011077773	3
GO:0042737	drug catabolic process	0.006936336	0.011077773	3
GO:0051123	RNA polymerase II preinitiation complex assembly	0.006936336	0.011077773	3
GO:0021766	hippocampus development	0.007061963	0.011268463	3
GO:0035051	cardiocyte differentiation	0.007200628	0.011469495	3
GO:0050729	positive regulation of inflammatory response	0.007200628	0.011469495	3
GO:0001556	oocyte maturation	0.007467883	0.011812008	3
GO:0002021	response to dietary excess	0.007467883	0.011812008	3
GO:0002026	regulation of the force of heart contraction	0.007467883	0.011812008	3
GO:0010971	positive regulation of G2/M transition of mitotic cell cycle	0.007467883	0.011812008	3
GO:0045672	positive regulation of osteoclast differentiation	0.007467883	0.011812008	3
GO:0048143	astrocyte activation	0.007467883	0.011812008	3
GO:1903203	regulation of oxidative stress-induced neuron death	0.007467883	0.011812008	3

GO:20003<br>53	positive regulation of endothelial cell apoptotic process	0.0074678<br>83	0.0118120<br>08	3
GO:00148<br>55	striated muscle cell proliferation	0.0075527<br>29	0.0118942<br>25	3
GO:00550<br>13	cardiac muscle cell development	0.0075527<br>29	0.0118942<br>25	3
GO:00551<br>17	regulation of cardiac muscle contraction	0.0075527<br>29	0.0118942<br>25	3
GO:00712<br>77	cellular response to calcium ion	0.0075527<br>29	0.0118942<br>25	3
GO:20002<br>43	positive regulation of reproductive process	0.0075527<br>29	0.0118942<br>25	3
GO:00518<br>96	regulation of protein kinase B signaling	0.0076087<br>94	0.0119720<br>98	3
GO:00512<br>51	positive regulation of lymphocyte activation	0.0076258<br>08	0.0119884<br>44	3
GO:00140<br>31	mesenchymal cell development	0.0078054<br>95	0.0122390<br>28	3
GO:00302<br>79	negative regulation of ossification	0.0078054<br>95	0.0122390<br>28	3
GO:00459<br>13	positive regulation of carbohydrate metabolic process	0.0078054<br>95	0.0122390<br>28	3
GO:00459<br>32	negative regulation of muscle contraction	0.0080172<br>66	0.0125091<br>08	3
GO:00714<br>50	cellular response to oxygen radical	0.0080172<br>66	0.0125091<br>08	3
GO:00714<br>51	cellular response to superoxide	0.0080172<br>66	0.0125091<br>08	3
GO:00723<br>76	protein activation cascade	0.0080172<br>66	0.0125091<br>08	3
GO:00723<br>78	blood coagulation, fibrin clot formation	0.0080172<br>66	0.0125091<br>08	3
GO:00092<br>67	cellular response to starvation	0.0080192<br>18	0.0125091<br>08	3
GO:00458<br>44	positive regulation of striated muscle tissue development	0.0080632<br>13	0.0125452<br>89	3
GO:00486<br>36	positive regulation of muscle organ development	0.0080632<br>13	0.0125452<br>89	3
GO:01101<br>10	positive regulation of animal organ morphogenesis	0.0080632<br>13	0.0125452<br>89	3
GO:00109<br>51	negative regulation of endopeptidase activity	0.0082421<br>84	0.0128127<br>28	3
GO:00346<br>37	cellular carbohydrate biosynthetic process	0.0083259<br>04	0.0129096<br>01	3

GO:19018 63	positive regulation of muscle tissue development	0.0083259 04	0.0129096 01	3
GO:20001 06	regulation of leukocyte apoptotic process	0.0083259 04	0.0129096 01	3
GO:00094 08	response to heat	0.0085385 75	0.0132002 33	3
GO:00359 67	cellular response to topologically incorrect protein	0.0085385 75	0.0132002 33	3
GO:00072 63	nitric oxide mediated signal transduction	0.0085843 06	0.0132002 33	3
GO:00190 48	modulation by virus of host process	0.0085843 06	0.0132002 33	3
GO:00310 63	regulation of histone deacetylation	0.0085843 06	0.0132002 33	3
GO:00488 73	homeostasis of number of cells within a tissue	0.0085843 06	0.0132002 33	3
GO:01501 17	positive regulation of cell-substrate junction organization	0.0085843 06	0.0132002 33	3
GO:20001 08	positive regulation of leukocyte apoptotic process	0.0085843 06	0.0132002 33	3
GO:00019 42	hair follicle development	0.0085935 89	0.0132002 33	3
GO:00550 72	iron ion homeostasis	0.0085935 89	0.0132002 33	3
GO:00900 49	regulation of cell migration involved in sprouting angiogenesis	0.0085935 89	0.0132002 33	3
GO:00020 28	regulation of sodium ion transport	0.0088662 89	0.0135960 32	3
GO:00700 98	chemokine-mediated signaling pathway	0.0088662 89	0.0135960 32	3
GO:00458 61	negative regulation of proteolysis	0.0088964 43	0.0136307 19	3
GO:00380 95	Fc-epsilon receptor signaling pathway	0.0090795 18	0.0138834 66	3
GO:00550 02	striated muscle cell development	0.0090795 18	0.0138834 66	3
GO:00224 04	molting cycle process	0.0091440 24	0.0138834 66	3
GO:00224 05	hair cycle process	0.0091440 24	0.0138834 66	3
GO:00550 06	cardiac cell development	0.0091440 24	0.0138834 66	3
GO:00003 03	response to superoxide	0.0091688 22	0.0138834 66	3

GO:0001516	prostaglandin biosynthetic process	0.009168822	0.013883466	3
GO:0007271	synaptic transmission, cholinergic	0.009168822	0.013883466	3
GO:0019934	cGMP-mediated signaling	0.009168822	0.013883466	3
GO:0036475	neuron death in response to oxidative stress	0.009168822	0.013883466	3
GO:0046457	prostanoid biosynthetic process	0.009168822	0.013883466	3
GO:0048147	negative regulation of fibroblast proliferation	0.009168822	0.013883466	3
GO:0071480	cellular response to gamma radiation	0.009168822	0.013883466	3
GO:1902751	positive regulation of cell cycle G2/M phase transition	0.009168822	0.013883466	3
GO:0043470	regulation of carbohydrate catabolic process	0.009426811	0.014250264	3
GO:0098773	skin epidermis development	0.009426811	0.014250264	3
GO:0030307	positive regulation of cell growth	0.009452309	0.014276882	3
GO:0033673	negative regulation of kinase activity	0.009617288	0.014513952	3
GO:0050714	positive regulation of protein secretion	0.009642391	0.014527605	3
GO:1990138	neuron projection extension	0.009642391	0.014527605	3
GO:0046849	bone remodeling	0.0097146 7	0.014599264	3
GO:0048477	oogenesis	0.0097146 7	0.014599264	3
GO:1904063	negative regulation of cation transmembrane transport	0.0097146 7	0.014599264	3
GO:0000305	response to oxygen radical	0.009770636	0.014599264	3
GO:0014072	response to isoquinoline alkaloid	0.009770636	0.014599264	3
GO:0043278	response to morphine	0.009770636	0.014599264	3
GO:0045948	positive regulation of translational initiation	0.009770636	0.014599264	3
GO:0060261	positive regulation of transcription initiation from RNA polymerase II promoter	0.009770636	0.014599264	3

GO:0086011	membrane repolarization during action potential	0.009770636	0.014599264	3
GO:1902253	regulation of intrinsic apoptotic signaling pathway by p53 class mediator	0.009770636	0.014599264	3
GO:0033209	tumor necrosis factor-mediated signaling pathway	0.009834947	0.014683232	3
GO:0031058	positive regulation of histone modification	0.010007618	0.014928707	3
GO:0035725	sodium ion transmembrane transport	0.010029989	0.014949753	3
GO:0006839	mitochondrial transport	0.010058874	0.014980468	3
GO:0010466	negative regulation of peptidase activity	0.01020912	0.015191721	3
GO:0032677	regulation of interleukin-8 production	0.010305672	0.015322796	3
GO:0008210	estrogen metabolic process	0.010389573	0.015359198	3
GO:0035767	endothelial cell chemotaxis	0.010389573	0.015359198	3
GO:0051968	positive regulation of synaptic transmission, glutamatergic	0.010389573	0.015359198	3
GO:0070528	protein kinase C signaling	0.010389573	0.015359198	3
GO:0086019	cell-cell signaling involved in cardiac conduction	0.010389573	0.015359198	3
GO:0097421	liver regeneration	0.010389573	0.015359198	3
GO:1901889	negative regulation of cell junction assembly	0.010389573	0.015359198	3
GO:0032535	regulation of cellular component size	0.010563355	0.015603358	3
GO:1901992	positive regulation of mitotic cell cycle phase transition	0.010608849	0.015657776	3
GO:0030324	lung development	0.01063015	0.015676428	3
GO:0007281	germ cell development	0.010669108	0.015721067	3
GO:0031349	positive regulation of defense response	0.010818438	0.015928135	3
GO:0051897	positive regulation of protein kinase B signaling	0.010835255	0.015939926	3
GO:0033028	myeloid cell apoptotic process	0.011025456	0.01618027	3

GO:0045736	negative regulation of cyclin-dependent protein serine/threonine kinase activity	0.011025456	0.0161802 7	3
GO:0046627	negative regulation of insulin receptor signaling pathway	0.011025456	0.0161802 7	3
GO:0035249	synaptic transmission, glutamatergic	0.011230635	0.016468021	3
GO:0030323	respiratory tube development	0.011465884	0.016799365	3
GO:0032651	regulation of interleukin-1 beta production	0.011549273	0.016880538	3
GO:1990868	response to chemokine	0.011549273	0.016880538	3
GO:1990869	cellular response to chemokine	0.011549273	0.016880538	3
GO:0010765	positive regulation of sodium ion transport	0.011678112	0.0169320 8	3
GO:0016242	negative regulation of macroautophagy	0.011678112	0.0169320 8	3
GO:0030431	sleep	0.011678112	0.0169320 8	3
GO:0032148	activation of protein kinase B activity	0.011678112	0.0169320 8	3
GO:0042311	vasodilation	0.011678112	0.0169320 8	3
GO:0045648	positive regulation of erythrocyte differentiation	0.011678112	0.0169320 8	3
GO:0048566	embryonic digestive tract development	0.011678112	0.0169320 8	3
GO:0070050	neuron cellular homeostasis	0.011678112	0.0169320 8	3
GO:0070232	regulation of T cell apoptotic process	0.011678112	0.0169320 8	3
GO:1904030	negative regulation of cyclin-dependent protein kinase activity	0.011678112	0.0169320 8	3
GO:0006942	regulation of striated muscle contraction	0.011873094	0.017183759	3
GO:0030316	osteoclast differentiation	0.011873094	0.017183759	3
GO:0001959	regulation of cytokine-mediated signaling pathway	0.011899178	0.017183759	3
GO:0006986	response to unfolded protein	0.011899178	0.017183759	3
GO:0055001	muscle cell development	0.011899178	0.017183759	3

GO:0001822	kidney development	0.011964864	0.017251081	3
GO:0016579	protein deubiquitination	0.011964864	0.017251081	3
GO:0022900	electron transport chain	0.012119721	0.017446552	3
GO:0038061	NIK/NF-kappaB signaling	0.012119721	0.017446552	3
GO:0043255	regulation of carbohydrate biosynthetic process	0.012202112	0.017537252	3
GO:0051817	modulation of process of other organism involved in symbiotic interaction	0.012202112	0.017537252	3
GO:0006691	leukotriene metabolic process	0.012347367	0.017661846	3
GO:0060674	placenta blood vessel development	0.012347367	0.017661846	3
GO:0070884	regulation of calcineurin-NFAT signaling cascade	0.012347367	0.017661846	3
GO:0071312	cellular response to alkaloid	0.012347367	0.017661846	3
GO:0110111	negative regulation of animal organ morphogenesis	0.012347367	0.017661846	3
GO:1900077	negative regulation of cellular response to insulin stimulus	0.012347367	0.017661846	3
GO:0045069	regulation of viral genome replication	0.012536338	0.017903847	3
GO:0070301	cellular response to hydrogen peroxide	0.012536338	0.017903847	3
GO:0043488	regulation of mRNA stability	0.012568651	0.017907593	3
GO:0050770	regulation of axonogenesis	0.012568651	0.017907593	3
GO:0051147	regulation of muscle cell differentiation	0.012568651	0.017907593	3
GO:0007215	glutamate receptor signaling pathway	0.012875787	0.018301962	3
GO:0019217	regulation of fatty acid metabolic process	0.012875787	0.018301962	3
GO:0042116	macrophage activation	0.012875787	0.018301962	3
GO:0051783	regulation of nuclear division	0.01302811	0.018409805	3
GO:0001569	branching involved in blood vessel morphogenesis	0.01303305	0.018409805	3

GO:0031572	G2 DNA damage checkpoint	0.01303305	0.018409805	3
GO:0034405	response to fluid shear stress	0.01303305	0.018409805	3
GO:0043368	positive T cell selection	0.01303305	0.018409805	3
GO:0071634	regulation of transforming growth factor beta production	0.01303305	0.018409805	3
GO:0106056	regulation of calcineurin-mediated signaling	0.01303305	0.018409805	3
GO:2000310	regulation of NMDA receptor activity	0.01303305	0.018409805	3
GO:0060485	mesenchyme development	0.013183341	0.018593069	3
GO:1902105	regulation of leukocyte differentiation	0.013183341	0.018593069	3
GO:0043502	regulation of muscle adaptation	0.013220469	0.018616412	3
GO:1903076	regulation of protein localization to plasma membrane	0.013220469	0.018616412	3
GO:0051048	negative regulation of secretion	0.013498182	0.018992694	3
GO:0072001	renal system development	0.013546345	0.019045653	3
GO:0032941	secretion by tissue	0.01373499	0.019221271	3
GO:0043243	positive regulation of protein-containing complex disassembly	0.01373499	0.019221271	3
GO:0060260	regulation of transcription initiation from RNA polymerase II promoter	0.01373499	0.019221271	3
GO:0070423	nucleotide-binding oligomerization domain containing signaling pathway	0.01373499	0.019221271	3
GO:0070897	transcription preinitiation complex assembly	0.01373499	0.019221271	3
GO:0090322	regulation of superoxide metabolic process	0.01373499	0.019221271	3
GO:0002696	positive regulation of leukocyte activation	0.013766881	0.019251011	3
GO:0007517	muscle organ development	0.013919994	0.019450087	3
GO:0002793	positive regulation of peptide secretion	0.014223363	0.019858643	3
GO:0021549	cerebellum development	0.014286026	0.019924457	3

GO:0031647	regulation of protein stability	0.014292524	0.019924457	3
GO:0035872	nucleotide-binding domain, leucine rich repeat containing receptor signaling pathway	0.014453019	0.020040121	3
GO:0044003	modulation by symbiont of host process	0.014453019	0.020040121	3
GO:0048009	insulin-like growth factor receptor signaling pathway	0.014453019	0.020040121	3
GO:0060045	positive regulation of cardiac muscle cell proliferation	0.014453019	0.020040121	3
GO:0071392	cellular response to estradiol stimulus	0.014453019	0.020040121	3
GO:0071604	transforming growth factor beta production	0.014453019	0.020040121	3
GO:0086005	ventricular cardiac muscle cell action potential	0.014453019	0.020040121	3
GO:0019233	sensory perception of pain	0.014651749	0.020253594	3
GO:0019395	fatty acid oxidation	0.014651749	0.020253594	3
GO:0032611	interleukin-1 beta production	0.014651749	0.020253594	3
GO:1905269	positive regulation of chromatin organization	0.014651749	0.020253594	3
GO:0043487	regulation of RNA stability	0.014720313	0.020332839	3
GO:0002040	sprouting angiogenesis	0.014972866	0.020650157	3
GO:0060759	regulation of response to cytokine stimulus	0.014972866	0.020650157	3
GO:1903426	regulation of reactive oxygen species biosynthetic process	0.015022757	0.020703186	3
GO:0070646	protein modification by small protein removal	0.015065942	0.020746899	3
GO:0007190	activation of adenylate cyclase activity	0.015186968	0.020771292	3
GO:0010737	protein kinase A signaling	0.015186968	0.020771292	3
GO:0010831	positive regulation of myotube differentiation	0.015186968	0.020771292	3
GO:0014037	Schwann cell differentiation	0.015186968	0.020771292	3
GO:0033146	regulation of intracellular estrogen receptor signaling pathway	0.015186968	0.020771292	2

GO:0035886	vascular associated smooth muscle cell differentiation	0.015186968	0.020771292	2
GO:0046326	positive regulation of glucose import	0.015186968	0.020771292	2
GO:0060416	response to growth hormone	0.015186968	0.020771292	2
GO:2000279	negative regulation of DNA biosynthetic process	0.015186968	0.020771292	2
GO:0010639	negative regulation of organelle organization	0.015352122	0.020981315	2
GO:0032526	response to retinoic acid	0.015399058	0.021029578	2
GO:0010613	positive regulation of cardiac muscle hypertrophy	0.015936671	0.021730962	2
GO:0043902	positive regulation of multi-organism process	0.015936671	0.021730962	2
GO:0010389	regulation of G2/M transition of mitotic cell cycle	0.016010471	0.021765985	2
GO:0016052	carbohydrate catabolic process	0.016010471	0.021765985	2
GO:0051054	positive regulation of DNA metabolic process	0.016010471	0.021765985	2
GO:1901888	regulation of cell junction assembly	0.016010471	0.021765985	2
GO:1901989	positive regulation of cell cycle phase transition	0.016167573	0.021946585	2
GO:1904659	glucose transmembrane transport	0.016167573	0.021946585	2
GO:0050867	positive regulation of cell activation	0.016190604	0.021961373	2
GO:0009746	response to hexose	0.016545835	0.022426407	2
GO:0032652	regulation of interleukin-1 production	0.0165598	0.022428535	2
GO:0002251	organ or tissue specific immune response	0.016701961	0.0224697 1	2
GO:0007212	dopamine receptor signaling pathway	0.016701961	0.0224697 1	2
GO:0007618	mating	0.016701961	0.0224697 1	2
GO:0014742	positive regulation of muscle hypertrophy	0.016701961	0.0224697 1	2
GO:0030890	positive regulation of B cell proliferation	0.016701961	0.0224697 1	2

GO:0038083	peptidyl-tyrosine autophosphorylation	0.016701961	0.02246971	2
GO:0042596	fear response	0.016701961	0.02246971	2
GO:0046825	regulation of protein export from nucleus	0.016701961	0.02246971	2
GO:2000008	regulation of protein localization to cell surface	0.016701961	0.02246971	2
GO:0043588	skin development	0.016883895	0.022697596	2
GO:0060541	respiratory system development	0.017092332	0.022960746	2
GO:0046034	ATP metabolic process	0.017336568	0.023264361	2
GO:0007009	plasma membrane organization	0.01736023	0.023264361	2
GO:0035821	modulation of process of other organism	0.01736023	0.023264361	2
GO:0035966	response to topologically incorrect protein	0.017369776	0.023264361	2
GO:0006509	membrane protein ectodomain proteolysis	0.017482676	0.023329234	2
GO:0010907	positive regulation of glucose metabolic process	0.017482676	0.023329234	2
GO:0046006	regulation of activated T cell proliferation	0.017482676	0.023329234	2
GO:0048246	macrophage chemotaxis	0.017482676	0.023329234	2
GO:0071470	cellular response to osmotic stress	0.017482676	0.023329234	2
GO:0070588	calcium ion transmembrane transport	0.017553636	0.023406663	2
GO:0042594	response to starvation	0.017650027	0.023517864	2
GO:0008645	hexose transmembrane transport	0.017768444	0.023646232	2
GO:1903532	positive regulation of secretion by cell	0.017772503	0.023646232	2
GO:0034284	response to monosaccharide	0.017933094	0.023842368	2
GO:0031623	receptor internalization	0.018181997	0.02415554	2
GO:0061013	regulation of mRNA catabolic process	0.018218985	0.02417744	2

GO:0045124	regulation of bone resorption	0.018278651	0.02417744	2
GO:0045773	positive regulation of axon extension	0.018278651	0.02417744	2
GO:0090317	negative regulation of intracellular protein transport	0.018278651	0.02417744	2
GO:1903146	regulation of autophagy of mitochondrion	0.018278651	0.02417744	2
GO:2000648	positive regulation of stem cell proliferation	0.018278651	0.02417744	2
GO:0015749	monosaccharide transmembrane transport	0.018600895	0.02454984	2
GO:0046916	cellular transition metal ion homeostasis	0.018600895	0.02454984	2
GO:1903828	negative regulation of cellular protein localization	0.018600895	0.02454984	2
GO:0030282	bone mineralization	0.019025141	0.025054944	2
GO:0051341	regulation of oxidoreductase activity	0.019025141	0.025054944	2
GO:0072676	lymphocyte migration	0.019025141	0.025054944	2
GO:0035329	hippo signaling	0.019089724	0.025121712	2
GO:0034219	carbohydrate transmembrane transport	0.01945474	0.025583459	2
GO:1901222	regulation of NIK/NF-kappaB signaling	0.019889694	0.026075977	2
GO:0010828	positive regulation of glucose transmembrane transport	0.019915736	0.026075977	2
GO:0014002	astrocyte development	0.019915736	0.026075977	2
GO:0034105	positive regulation of tissue remodeling	0.019915736	0.026075977	2
GO:0043114	regulation of vascular permeability	0.019915736	0.026075977	2
GO:0090311	regulation of protein deacetylation	0.019915736	0.026075977	2
GO:0050870	positive regulation of T cell activation	0.019994045	0.026159579	2
GO:0006470	protein dephosphorylation	0.020061399	0.026228738	2
GO:0051235	maintenance of location	0.020300432	0.026522091	2

GO:0010863	positive regulation of phospholipase C activity	0.020756525	0.026987112	2
GO:0017144	drug metabolic process	0.020756525	0.026987112	2
GO:0035987	endodermal cell differentiation	0.020756525	0.026987112	2
GO:0044275	cellular carbohydrate catabolic process	0.020756525	0.026987112	2
GO:0048066	developmental pigmentation	0.020756525	0.026987112	2
GO:0050798	activated T cell proliferation	0.020756525	0.026987112	2
GO:0032612	interleukin-1 production	0.020775681	0.026987112	2
GO:1900542	regulation of purine nucleotide metabolic process	0.020775681	0.026987112	2
GO:0050792	regulation of viral process	0.020920285	0.027155455	2
GO:0021782	glial cell development	0.021226717	0.027494049	2
GO:0042752	regulation of circadian rhythm	0.021226717	0.027494049	2
GO:0051928	positive regulation of calcium ion transport	0.021226717	0.027494049	2
GO:0030574	collagen catabolic process	0.021611935	0.027933019	2
GO:0042572	retinol metabolic process	0.021611935	0.027933019	2
GO:0043268	positive regulation of potassium ion transport	0.021611935	0.027933019	2
GO:0006140	regulation of nucleotide metabolic process	0.021683118	0.027965098	2
GO:0070252	actin-mediated cell contraction	0.021683118	0.027965098	2
GO:1904375	regulation of protein localization to cell periphery	0.021683118	0.027965098	2
GO:0071375	cellular response to peptide hormone stimulus	0.021773978	0.02806228	2
GO:0006721	terpenoid metabolic process	0.022144885	0.02851999	2
GO:0050863	regulation of T cell activation	0.022280272	0.028673945	2
GO:0031952	regulation of protein autophosphorylation	0.022481806	0.028851232	2

GO:0045601	regulation of endothelial cell differentiation	0.022481806	0.028851232	2
GO:0055023	positive regulation of cardiac muscle tissue growth	0.022481806	0.028851232	2
GO:1900274	regulation of phospholipase C activity	0.022481806	0.028851232	2
GO:0034605	cellular response to heat	0.022612018	0.028977232	2
GO:0043500	muscle adaptation	0.022612018	0.028977232	2
GO:0016485	protein processing	0.022851083	0.029262868	2
GO:0006692	prostanoid metabolic process	0.023365985	0.029753789	2
GO:0006693	prostaglandin metabolic process	0.023365985	0.029753789	2
GO:0015695	organic cation transport	0.023365985	0.029753789	2
GO:0015872	dopamine transport	0.023365985	0.029753789	2
GO:0031279	regulation of cyclase activity	0.023365985	0.029753789	2
GO:0042149	cellular response to glucose starvation	0.023365985	0.029753789	2
GO:0045058	T cell selection	0.023365985	0.029753789	2
GO:1900087	positive regulation of G1/S transition of mitotic cell cycle	0.023365985	0.029753789	2
GO:0045732	positive regulation of protein catabolic process	0.023518066	0.029926387	2
GO:0071621	granulocyte chemotaxis	0.023562383	0.029961709	2
GO:0072089	stem cell proliferation	0.024045614	0.0305547 1	2
GO:0045646	regulation of erythrocyte differentiation	0.024264314	0.030660361	2
GO:0045981	positive regulation of nucleotide metabolic process	0.024264314	0.030660361	2
GO:0046850	regulation of bone remodeling	0.024264314	0.030660361	2
GO:0048546	digestive tract morphogenesis	0.024264314	0.030660361	2
GO:0101023	vascular endothelial cell proliferation	0.024264314	0.030660361	2

GO:1900544	positive regulation of purine nucleotide metabolic process	0.024264314	0.030660361	2
GO:1903580	positive regulation of ATP metabolic process	0.024264314	0.030660361	2
GO:1905562	regulation of vascular endothelial cell proliferation	0.024264314	0.030660361	2
GO:0051047	positive regulation of secretion	0.024382038	0.030787617	2
GO:0002576	platelet degranulation	0.024534211	0.030958165	2
GO:0043405	regulation of MAP kinase activity	0.024926811	0.031431643	2
GO:0035272	exocrine system development	0.025176641	0.031636437	2
GO:0038084	vascular endothelial growth factor signaling pathway	0.025176641	0.031636437	2
GO:0045912	negative regulation of carbohydrate metabolic process	0.025176641	0.031636437	2
GO:0071354	cellular response to interleukin-6	0.025176641	0.031636437	2
GO:0071675	regulation of mononuclear cell migration	0.025176641	0.031636437	2
GO:0016042	lipid catabolic process	0.025202121	0.031646478	2
GO:0010001	glial cell differentiation	0.025237104	0.031668429	2
GO:1903311	regulation of mRNA metabolic process	0.025479387	0.031950298	2
GO:0006766	vitamin metabolic process	0.025527493	0.031966316	2
GO:0044106	cellular amine metabolic process	0.025527493	0.031966316	2
GO:0043903	regulation of symbiotic process	0.025589795	0.032022171	2
GO:0010256	endomembrane system organization	0.025670946	0.032101521	2
GO:0006998	nuclear envelope organization	0.026102812	0.03250678	2
GO:0010883	regulation of lipid storage	0.026102812	0.03250678	2
GO:0038066	p38MAPK cascade	0.026102812	0.03250678	2
GO:0045839	negative regulation of mitotic nuclear division	0.026102812	0.03250678	2

GO:0060421	positive regulation of heart growth	0.026102812	0.03250678	2
GO:0071622	regulation of granulocyte chemotaxis	0.026102812	0.03250678	2
GO:0009743	response to carbohydrate	0.026304098	0.032734919	2
GO:0042742	defense response to bacterium	0.026608086	0.033090468	2
GO:0043124	negative regulation of I-kappaB kinase/NF-kappaB signaling	0.027042677	0.033561737	2
GO:0045599	negative regulation of fat cell differentiation	0.027042677	0.033561737	2
GO:0045661	regulation of myoblast differentiation	0.027042677	0.033561737	2
GO:0030336	negative regulation of cell migration	0.027184274	0.033691253	2
GO:0046942	carboxylic acid transport	0.027184274	0.033691253	2
GO:0032368	regulation of lipid transport	0.027578351	0.034146129	2
GO:0048285	organelle fission	0.027589039	0.034146129	2
GO:0006968	cellular defense response	0.027996084	0.03460258	2
GO:2000179	positive regulation of neural precursor cell proliferation	0.027996084	0.03460258	2
GO:0015849	organic acid transport	0.028063445	0.034662161	2
GO:0033044	regulation of chromosome organization	0.028960574	0.035651413	2
GO:0001706	endoderm formation	0.028962884	0.035651413	2
GO:0042304	regulation of fatty acid biosynthetic process	0.028962884	0.035651413	2
GO:0051452	intracellular pH reduction	0.028962884	0.035651413	2
GO:0070741	response to interleukin-6	0.028962884	0.035651413	2
GO:0007409	axonogenesis	0.029088644	0.035781874	2
GO:0002705	positive regulation of leukocyte mediated immunity	0.029172651	0.035836487	2
GO:0055076	transition metal ion homeostasis	0.029172651	0.035836487	2

GO:0035304	regulation of protein dephosphorylation	0.02971476	0.036477664	2
GO:0001755	neural crest cell migration	0.029942929	0.036683102	2
GO:0086065	cell communication involved in cardiac conduction	0.029942929	0.036683102	2
GO:0090183	regulation of kidney development	0.029942929	0.036683102	2
GO:0009308	amine metabolic process	0.030262198	0.037024105	2
GO:0097553	calcium ion transmembrane import into cytosol	0.030262198	0.037024105	2
GO:0043312	neutrophil degranulation	0.030378806	0.037141656	2
GO:0042593	glucose homeostasis	0.0308415	0.037670261	2
GO:0019748	secondary metabolic process	0.030936072	0.037670261	2
GO:0032615	interleukin-12 production	0.030936072	0.037670261	2
GO:0061178	regulation of insulin secretion involved in cellular response to glucose stimulus	0.030936072	0.037670261	2
GO:0071398	cellular response to fatty acid	0.030936072	0.037670261	2
GO:1905517	macrophage migration	0.030936072	0.037670261	2
GO:0002283	neutrophil activation involved in immune response	0.031170735	0.03793048	2
GO:0033500	carbohydrate homeostasis	0.031239206	0.037988253	2
GO:0031333	negative regulation of protein-containing complex assembly	0.03137304	0.03812538	2
GO:0016311	dephosphorylation	0.031706163	0.038504341	2
GO:2000146	negative regulation of cell motility	0.031760678	0.038544676	2
GO:0031638	zymogen activation	0.031942166	0.038661209	2
GO:0045851	pH reduction	0.031942166	0.038661209	2
GO:1900408	negative regulation of cellular response to oxidative stress	0.031942166	0.038661209	2
GO:1903202	negative regulation of oxidative stress-induced cell death	0.031942166	0.038661209	2

GO:00067<br>20	isoprenoid metabolic process	0.0325051<br>31	0.0392900<br>33	2
GO:00464<br>34	organophosphate catabolic process	0.0325051<br>31	0.0392900<br>33	2
GO:00016<br>58	branching involved in ureteric bud morphogenesis	0.0329610<br>66	0.0397085<br>1	2
GO:00323<br>87	negative regulation of intracellular transport	0.0329610<br>66	0.0397085<br>1	2
GO:00454<br>54	cell redox homeostasis	0.0329610<br>66	0.0397085<br>1	2
GO:00600<br>43	regulation of cardiac muscle cell proliferation	0.0329610<br>66	0.0397085<br>1	2
GO:00989<br>30	axonal transport	0.0329610<br>66	0.0397085<br>1	2
GO:00226<br>04	regulation of cell morphogenesis	0.0336276<br>03	0.0404845<br>39	2
GO:00715<br>60	cellular response to transforming growth factor beta stimulus	0.0336891<br>37	0.0405316<br>54	2
GO:00108<br>23	negative regulation of mitochondrion organization	0.0339926<br>29	0.0407073<br>24	2
GO:00325<br>15	negative regulation of phosphoprotein phosphatase activity	0.0339926<br>29	0.0407073<br>24	2
GO:00487<br>47	muscle fiber development	0.0339926<br>29	0.0407073<br>24	2
GO:00517<br>84	negative regulation of nuclear division	0.0339926<br>29	0.0407073<br>24	2
GO:00610<br>98	positive regulation of protein tyrosine kinase activity	0.0339926<br>29	0.0407073<br>24	2
GO:19028<br>08	positive regulation of cell cycle G1/S phase transition	0.0339926<br>29	0.0407073<br>24	2
GO:19028<br>83	negative regulation of response to oxidative stress	0.0339926<br>29	0.0407073<br>24	2
GO:00000<br>86	G2/M transition of mitotic cell cycle	0.0345301<br>06	0.0413236<br>23	2
GO:00613<br>37	cardiac conduction	0.0348328<br>44	0.0416308<br>54	2
GO:19036<br>70	regulation of sprouting angiogenesis	0.0348328<br>44	0.0416308<br>54	2
GO:00619<br>12	selective autophagy	0.0350367<br>11	0.0417916<br>98	2
GO:00903<br>03	positive regulation of wound healing	0.0350367<br>11	0.0417916<br>98	2
GO:19030<br>78	positive regulation of protein localization to plasma membrane	0.0350367<br>11	0.0417916<br>98	2

GO:0006959	humoral immune response	0.035751123	0.042615755	2
GO:0035148	tube formation	0.036028345	0.042797931	2
GO:0060041	retina development in camera-type eye	0.036028345	0.042797931	2
GO:0097530	granulocyte migration	0.036028345	0.042797931	2
GO:0010518	positive regulation of phospholipase activity	0.036093171	0.042797931	2
GO:0010676	positive regulation of cellular carbohydrate metabolic process	0.036093171	0.042797931	2
GO:0010803	regulation of tumor necrosis factor-mediated signaling pathway	0.036093171	0.042797931	2
GO:0048857	neural nucleus development	0.036093171	0.042797931	2
GO:0055025	positive regulation of cardiac muscle tissue development	0.036093171	0.042797931	2
GO:0071559	response to transforming growth factor beta	0.036248629	0.042926008	2
GO:1901617	organic hydroxy compound biosynthetic process	0.036248629	0.042926008	2
GO:0008643	carbohydrate transport	0.036633978	0.043297335	2
GO:0016331	morphogenesis of embryonic epithelium	0.036633978	0.043297335	2
GO:0050871	positive regulation of B cell activation	0.036633978	0.043297335	2
GO:0010830	regulation of myotube differentiation	0.037161868	0.043835346	2
GO:0032613	interleukin-10 production	0.037161868	0.043835346	2
GO:0055008	cardiac muscle tissue morphogenesis	0.037161868	0.043835346	2
GO:0001837	epithelial to mesenchymal transition	0.037244855	0.043876032	2
GO:1903169	regulation of calcium ion transmembrane transport	0.037244855	0.043876032	2
GO:0016202	regulation of striated muscle tissue development	0.037860966	0.044543837	2
GO:0030048	actin filament-based movement	0.037860966	0.044543837	2
GO:0002437	inflammatory response to antigenic stimulus	0.038242661	0.044963671	2

GO:0014065	phosphatidylinositol 3-kinase signaling	0.038482301	0.045216046	2
GO:0043406	positive regulation of MAP kinase activity	0.038918065	0.045668751	2
GO:0048863	stem cell differentiation	0.038918065	0.045668751	2
GO:0006687	glycosphingolipid metabolic process	0.039335413	0.046009305	2
GO:0035308	negative regulation of protein dephosphorylation	0.039335413	0.046009305	2
GO:0035773	insulin secretion involved in cellular response to glucose stimulus	0.039335413	0.046009305	2
GO:0060675	ureteric bud morphogenesis	0.039335413	0.046009305	2
GO:0098586	cellular response to virus	0.039335413	0.046009305	2
GO:1901861	regulation of muscle tissue development	0.039740607	0.046453219	2
GO:0048634	regulation of muscle organ development	0.040377557	0.047088217	2
GO:0098739	import across plasma membrane	0.040377557	0.047088217	2
GO:1903900	regulation of viral life cycle	0.040377557	0.047088217	2
GO:0032922	circadian regulation of gene expression	0.040439986	0.047088217	2
GO:0034394	protein localization to cell surface	0.040439986	0.047088217	2
GO:0072171	mesonephric tubule morphogenesis	0.040439986	0.047088217	2
GO:0007519	skeletal muscle tissue development	0.041019691	0.047732509	2
GO:0002548	monocyte chemotaxis	0.041556242	0.048201876	2
GO:0045123	cellular extravasation	0.041556242	0.048201876	2
GO:0045670	regulation of osteoclast differentiation	0.041556242	0.048201876	2
GO:0046513	ceramide biosynthetic process	0.041556242	0.048201876	2
GO:0072678	T cell migration	0.041556242	0.048201876	2
GO:0051092	positive regulation of NF-kappaB transcription factor activity	0.041666998	0.048268462	2

GO:00550<br>67	monovalent inorganic cation homeostasis	0.0416669<br>98	0.0482684<br>62	2
GO:00302<br>58	lipid modification	0.0421716<br>7	0.0488218<br>35	2
GO:00026<br>37	regulation of immunoglobulin production	0.0426840<br>46	0.0492888<br>7	2
GO:00027<br>53	cytoplasmic pattern recognition receptor signaling pathway	0.0426840<br>46	0.0492888<br>7	2
GO:00507<br>66	positive regulation of phagocytosis	0.0426840<br>46	0.0492888<br>7	2
GO:19043<br>77	positive regulation of protein localization to cell periphery	0.0426840<br>46	0.0492888<br>7	2
GO:00400<br>13	negative regulation of locomotion	0.0430663<br>14	0.0496985<br>73	2
GO:19033<br>05	regulation of regulated secretory pathway	0.0436398<br>48	0.0503283<br>34	2
GO:00486<br>62	negative regulation of smooth muscle cell proliferation	0.0438232<br>65	0.0505076<br>71	2
GO:00512<br>71	negative regulation of cellular component movement	0.0442360<br>91	0.0509039<br>23	2
GO:00066<br>65	sphingolipid metabolic process	0.0443077<br>34	0.0509039<br>23	2
GO:00070<br>88	regulation of mitotic nuclear division	0.0443077<br>34	0.0509039<br>23	2
GO:00312<br>14	biomineral tissue development	0.0443077<br>34	0.0509039<br>23	2
GO:01101<br>48	biomineralization	0.0443077<br>34	0.0509039<br>23	2
GO:00080<br>88	axo-dendritic transport	0.0449737<br>63	0.0515788<br>71	2
GO:00323<br>70	positive regulation of lipid transport	0.0449737<br>63	0.0515788<br>71	2
GO:00072<br>59	receptor signaling pathway via JAK-STAT	0.0449807<br>36	0.0515788<br>71	2
GO:00148<br>23	response to activity	0.0461354<br>08	0.0528359<br>54	2
GO:00425<br>31	positive regulation of tyrosine phosphorylation of STAT protein	0.0461354<br>08	0.0528359<br>54	2
GO:19035<br>31	negative regulation of secretion by cell	0.0463420<br>36	0.0528974<br>92	2
GO:00421<br>29	regulation of T cell proliferation	0.0470303<br>09	0.0528974<br>92	2
GO:00026<br>76	regulation of chronic inflammatory response	0.0472123<br>19	0.0528974<br>92	2

GO:0002934	desmosome organization	0.047212319	0.052897492	2
GO:0010269	response to selenium ion	0.047212319	0.052897492	2
GO:0010749	regulation of nitric oxide mediated signal transduction	0.047212319	0.052897492	2
GO:0014041	regulation of neuron maturation	0.047212319	0.052897492	2
GO:0014877	response to muscle inactivity involved in regulation of muscle adaptation	0.047212319	0.052897492	2
GO:0014894	response to denervation involved in regulation of muscle adaptation	0.047212319	0.052897492	2
GO:0021548	pons development	0.047212319	0.052897492	2
GO:0021936	regulation of cerebellar granule cell precursor proliferation	0.047212319	0.052897492	2
GO:0042541	hemoglobin biosynthetic process	0.047212319	0.052897492	2
GO:0045792	negative regulation of cell size	0.047212319	0.052897492	2
GO:0048739	cardiac muscle fiber development	0.047212319	0.052897492	2
GO:0051342	regulation of cyclic-nucleotide phosphodiesterase activity	0.047212319	0.052897492	2
GO:0051541	elastin metabolic process	0.047212319	0.052897492	2
GO:0060513	prostatic bud formation	0.047212319	0.052897492	2
GO:0060768	regulation of epithelial cell proliferation involved in prostate gland development	0.047212319	0.052897492	2
GO:0070091	glucagon secretion	0.047212319	0.052897492	2
GO:0070092	regulation of glucagon secretion	0.047212319	0.052897492	2
GO:0070099	regulation of chemokine-mediated signaling pathway	0.047212319	0.052897492	2
GO:0071394	cellular response to testosterone stimulus	0.047212319	0.052897492	2
GO:0086070	SA node cell to atrial cardiac muscle cell communication	0.047212319	0.052897492	2
GO:0097050	type B pancreatic cell apoptotic process	0.047212319	0.052897492	2
GO:1900222	negative regulation of amyloid-beta clearance	0.047212319	0.052897492	2

GO:1901725	regulation of histone deacetylase activity	0.047212319	0.052897492	2
GO:1903431	positive regulation of cell maturation	0.047212319	0.052897492	2
GO:1903862	positive regulation of oxidative phosphorylation	0.047212319	0.052897492	2
GO:1904338	regulation of dopaminergic neuron differentiation	0.047212319	0.052897492	2
GO:1904526	regulation of microtubule binding	0.047212319	0.052897492	2
GO:1904779	regulation of protein localization to centrosome	0.047212319	0.052897492	2
GO:2000048	negative regulation of cell-cell adhesion mediated by cadherin	0.047212319	0.052897492	2
GO:2000425	regulation of apoptotic cell clearance	0.047212319	0.052897492	2
GO:2000659	regulation of interleukin-1-mediated signaling pathway	0.047212319	0.052897492	2
GO:2001054	negative regulation of mesenchymal cell apoptotic process	0.047212319	0.052897492	2
GO:0050663	cytokine secretion	0.047308069	0.052906493	2
GO:0070988	demethylation	0.047308069	0.052906493	2
GO:1904427	positive regulation of calcium ion transmembrane transport	0.047308069	0.052906493	2
GO:0060538	skeletal muscle organ development	0.047723646	0.053338283	2
GO:0003208	cardiac ventricle morphogenesis	0.048491615	0.053996493	2
GO:0010517	regulation of phospholipase activity	0.048491615	0.053996493	2
GO:0010611	regulation of cardiac muscle hypertrophy	0.048491615	0.053996493	2
GO:0051145	smooth muscle cell differentiation	0.048491615	0.053996493	2
GO:0051155	positive regulation of striated muscle cell differentiation	0.048491615	0.053996493	2
GO:0072078	nephron tubule morphogenesis	0.048491615	0.053996493	2
GO:0032635	interleukin-6 production	0.049125459	0.05466865	2
GO:0051403	stress-activated MAPK cascade	0.04964888	0.055183256	2

GO:0140014	mitotic nuclear division	0.04964888	0.055183256	2
GO:1903036	positive regulation of response to wounding	0.049685916	0.05519052	2
GO:0002244	hematopoietic progenitor cell differentiation	0.050547367	0.055342251	2
GO:0033143	regulation of intracellular steroid hormone receptor signaling pathway	0.050890844	0.055342251	2
GO:0045685	regulation of glial cell differentiation	0.050890844	0.055342251	2
GO:0072088	nephron epithelium morphogenesis	0.050890844	0.055342251	2
GO:1901983	regulation of protein acetylation	0.050890844	0.055342251	2
GO:0006527	arginine catabolic process	0.05181052	0.055342251	2
GO:0007028	cytoplasm organization	0.05181052	0.055342251	2
GO:0007191	adenylate cyclase-activating dopamine receptor signaling pathway	0.05181052	0.055342251	2
GO:0008611	ether lipid biosynthetic process	0.05181052	0.055342251	2
GO:0010739	positive regulation of protein kinase A signaling	0.05181052	0.055342251	2
GO:0010918	positive regulation of mitochondrial membrane potential	0.05181052	0.055342251	2
GO:0014870	response to muscle inactivity	0.05181052	0.055342251	2
GO:0017085	response to insecticide	0.05181052	0.055342251	2
GO:0018401	peptidyl-proline hydroxylation to 4-hydroxy-L-proline	0.05181052	0.055342251	2
GO:0031284	positive regulation of guanylate cyclase activity	0.05181052	0.055342251	2
GO:0031442	positive regulation of mRNA 3'-end processing	0.05181052	0.055342251	2
GO:0031915	positive regulation of synaptic plasticity	0.05181052	0.055342251	2
GO:0032253	dense core granule localization	0.05181052	0.055342251	2
GO:0033148	positive regulation of intracellular estrogen receptor signaling pathway	0.05181052	0.055342251	2
GO:0033197	response to vitamin E	0.05181052	0.055342251	2

GO:0035331	negative regulation of hippo signaling	0.05181052	0.055342251	2
GO:0035747	natural killer cell chemotaxis	0.05181052	0.055342251	2
GO:0043471	regulation of cellular carbohydrate catabolic process	0.05181052	0.055342251	2
GO:0043619	regulation of transcription from RNA polymerase II promoter in response to oxidative stress	0.05181052	0.055342251	2
GO:0045657	positive regulation of monocyte differentiation	0.05181052	0.055342251	2
GO:0045945	positive regulation of transcription by RNA polymerase III	0.05181052	0.055342251	2
GO:0046504	glycerol ether biosynthetic process	0.05181052	0.055342251	2
GO:0048742	regulation of skeletal muscle fiber development	0.05181052	0.055342251	2
GO:0048875	chemical homeostasis within a tissue	0.05181052	0.055342251	2
GO:0051024	positive regulation of immunoglobulin secretion	0.05181052	0.055342251	2
GO:0051610	serotonin uptake	0.05181052	0.055342251	2
GO:0051712	positive regulation of killing of cells of other organism	0.05181052	0.055342251	2
GO:0051974	negative regulation of telomerase activity	0.05181052	0.055342251	2
GO:0060281	regulation of oocyte development	0.05181052	0.055342251	2
GO:0060354	negative regulation of cell adhesion molecule production	0.05181052	0.055342251	2
GO:0060767	epithelial cell proliferation involved in prostate gland development	0.05181052	0.055342251	2
GO:0060947	cardiac vascular smooth muscle cell differentiation	0.05181052	0.055342251	2
GO:0070673	response to interleukin-18	0.05181052	0.055342251	2
GO:0071281	cellular response to iron ion	0.05181052	0.055342251	2
GO:0071803	positive regulation of podosome assembly	0.05181052	0.055342251	2
GO:0086016	AV node cell action potential	0.05181052	0.055342251	2
GO:0086027	AV node cell to bundle of His cell signaling	0.05181052	0.055342251	2

GO:0090269	fibroblast growth factor production	0.05181052	0.055342251	2
GO:0090270	regulation of fibroblast growth factor production	0.05181052	0.055342251	2
GO:0090557	establishment of endothelial intestinal barrier	0.05181052	0.055342251	2
GO:0097384	cellular lipid biosynthetic process	0.05181052	0.055342251	2
GO:0099519	dense core granule cytoskeletal transport	0.05181052	0.055342251	2
GO:0099624	atrial cardiac muscle cell membrane repolarization	0.05181052	0.055342251	2
GO:0106049	regulation of cellular response to osmotic stress	0.05181052	0.055342251	2
GO:0106072	negative regulation of adenylate cyclase-activating G protein-coupled receptor signaling pathway	0.05181052	0.055342251	2
GO:0140052	cellular response to oxidised low-density lipoprotein particle stimulus	0.05181052	0.055342251	2
GO:1901033	positive regulation of response to reactive oxygen species	0.05181052	0.055342251	2
GO:1901950	dense core granule transport	0.05181052	0.055342251	2
GO:1902513	regulation of organelle transport along microtubule	0.05181052	0.055342251	2
GO:1904321	response to forskolin	0.05181052	0.055342251	2
GO:1904322	cellular response to forskolin	0.05181052	0.055342251	2
GO:1905879	regulation of oogenesis	0.05181052	0.055342251	2
GO:1990440	positive regulation of transcription from RNA polymerase II promoter in response to endoplasmic reticulum stress	0.05181052	0.055342251	2
GO:2000480	negative regulation of cAMP-dependent protein kinase activity	0.05181052	0.055342251	2
GO:2000551	regulation of T-helper 2 cell cytokine production	0.05181052	0.055342251	2
GO:2000574	regulation of microtubule motor activity	0.05181052	0.055342251	2
GO:2000615	regulation of histone H3-K9 acetylation	0.05181052	0.055342251	2
GO:2000628	regulation of miRNA metabolic process	0.05181052	0.055342251	2
GO:2001138	regulation of phospholipid transport	0.05181052	0.055342251	2

GO:2001140	positive regulation of phospholipid transport	0.05181052	0.055342251	2
GO:0051099	positive regulation of binding	0.051989257	0.055396548	2
GO:0097696	receptor signaling pathway via STAT	0.051989257	0.055396548	2
GO:0008306	associative learning	0.05210627	0.055396548	2
GO:0014743	regulation of muscle hypertrophy	0.05210627	0.055396548	2
GO:0033077	T cell differentiation in thymus	0.05210627	0.055396548	2
GO:0060193	positive regulation of lipase activity	0.05210627	0.055396548	2
GO:0060415	muscle tissue morphogenesis	0.05210627	0.055396548	2
GO:0071229	cellular response to acid chemical	0.05210627	0.055396548	2
GO:0050680	negative regulation of epithelial cell proliferation	0.052717659	0.056013633	2
GO:0007422	peripheral nervous system development	0.053332068	0.05646751	2
GO:0030500	regulation of bone mineralization	0.053332068	0.05646751	2
GO:0032413	negative regulation of ion transmembrane transporter activity	0.053332068	0.05646751	2
GO:0060411	cardiac septum morphogenesis	0.053332068	0.05646751	2
GO:0061333	renal tubule morphogenesis	0.053332068	0.05646751	2
GO:0072028	nephron morphogenesis	0.053332068	0.05646751	2
GO:0051604	protein maturation	0.05337339	0.056478213	2
GO:0060395	SMAD protein signal transduction	0.054568113	0.057708687	2
GO:0000422	autophagy of mitochondrion	0.055814279	0.058269778	2
GO:0006446	regulation of translational initiation	0.055814279	0.058269778	2
GO:0007492	endoderm development	0.055814279	0.058269778	2
GO:0016575	histone deacetylation	0.055814279	0.058269778	2

GO:0032272	negative regulation of protein polymerization	0.055814279	0.058269778	2
GO:0043462	regulation of ATPase activity	0.055814279	0.058269778	2
GO:0061726	mitochondrion disassembly	0.055814279	0.058269778	2
GO:0000280	nuclear division	0.056075866	0.058269778	2
GO:0000185	activation of MAPKKK activity	0.056386773	0.058269778	2
GO:0001976	nervous system process involved in regulation of systemic arterial blood pressure	0.056386773	0.058269778	2
GO:0002551	mast cell chemotaxis	0.056386773	0.058269778	2
GO:0002863	positive regulation of inflammatory response to antigenic stimulus	0.056386773	0.058269778	2
GO:0002923	regulation of humoral immune response mediated by circulating immunoglobulin	0.056386773	0.058269778	2
GO:0003334	keratinocyte development	0.056386773	0.058269778	2
GO:0006069	ethanol oxidation	0.056386773	0.058269778	2
GO:0007320	insemination	0.056386773	0.058269778	2
GO:0014831	gastro-intestinal system smooth muscle contraction	0.056386773	0.058269778	2
GO:0031392	regulation of prostaglandin biosynthetic process	0.056386773	0.058269778	2
GO:0033234	negative regulation of protein sumoylation	0.056386773	0.058269778	2
GO:0035404	histone-serine phosphorylation	0.056386773	0.058269778	2
GO:0036462	TRAIL-activated apoptotic signaling pathway	0.056386773	0.058269778	2
GO:0038003	opioid receptor signaling pathway	0.056386773	0.058269778	2
GO:0042368	vitamin D biosynthetic process	0.056386773	0.058269778	2
GO:0042416	dopamine biosynthetic process	0.056386773	0.058269778	2
GO:0046322	negative regulation of fatty acid oxidation	0.056386773	0.058269778	2
GO:0051095	regulation of helicase activity	0.056386773	0.058269778	2

GO:0051549	positive regulation of keratinocyte migration	0.056386773	0.058269778	2
GO:0051798	positive regulation of hair follicle development	0.056386773	0.058269778	2
GO:0055015	ventricular cardiac muscle cell development	0.056386773	0.058269778	2
GO:0060453	regulation of gastric acid secretion	0.056386773	0.058269778	2
GO:0071679	commissural neuron axon guidance	0.056386773	0.058269778	2
GO:0072520	seminiferous tubule development	0.056386773	0.058269778	2
GO:0072540	T-helper 17 cell lineage commitment	0.056386773	0.058269778	2
GO:0086067	AV node cell to bundle of His cell communication	0.056386773	0.058269778	2
GO:0097201	negative regulation of transcription from RNA polymerase II promoter in response to stress	0.056386773	0.058269778	2
GO:0150065	regulation of deacetylase activity	0.056386773	0.058269778	2
GO:1901503	ether biosynthetic process	0.056386773	0.058269778	2
GO:1902337	regulation of apoptotic process involved in morphogenesis	0.056386773	0.058269778	2
GO:2001053	regulation of mesenchymal cell apoptotic process	0.056386773	0.058269778	2
GO:2001214	positive regulation of vasculogenesis	0.056386773	0.058269778	2
GO:0006110	regulation of glycolytic process	0.057070442	0.058841859	2
GO:0014032	neural crest cell development	0.057070442	0.058841859	2
GO:0055021	regulation of cardiac muscle tissue growth	0.057070442	0.058841859	2
GO:2000300	regulation of synaptic vesicle exocytosis	0.057070442	0.058841859	2
GO:0031098	stress-activated protein kinase signaling cascade	0.057246708	0.058989984	2
GO:2001252	positive regulation of chromosome organization	0.057954269	0.059685103	2
GO:0001937	negative regulation of endothelial cell proliferation	0.058336479	0.059942262	2
GO:0019915	lipid storage	0.058336479	0.059942262	2

GO:0033238	regulation of cellular amine metabolic process	0.058336479	0.059942262	2
GO:0050829	defense response to Gram-negative bacterium	0.058336479	0.059942262	2
GO:0006611	protein export from nucleus	0.059494265	0.060990853	2
GO:1902930	regulation of alcohol biosynthetic process	0.059612269	0.060990853	2
GO:0001570	vasculogenesis	0.06089769	0.060990853	2
GO:0002718	regulation of cytokine production involved in immune response	0.06089769	0.060990853	2
GO:0048644	muscle organ morphogenesis	0.06089769	0.060990853	2
GO:0048708	astrocyte differentiation	0.06089769	0.060990853	2
GO:0000722	telomere maintenance via recombination	0.060941181	0.060990853	2
GO:0002679	respiratory burst involved in defense response	0.060941181	0.060990853	2
GO:0003084	positive regulation of systemic arterial blood pressure	0.060941181	0.060990853	2
GO:0006684	sphingomyelin metabolic process	0.060941181	0.060990853	2
GO:0007171	activation of transmembrane receptor protein tyrosine kinase activity	0.060941181	0.060990853	2
GO:0009886	post-embryonic animal morphogenesis	0.060941181	0.060990853	2
GO:0010459	negative regulation of heart rate	0.060941181	0.060990853	2
GO:0010642	negative regulation of platelet-derived growth factor receptor signaling pathway	0.060941181	0.060990853	2
GO:0010870	positive regulation of receptor biosynthetic process	0.060941181	0.060990853	2
GO:0019627	urea metabolic process	0.060941181	0.060990853	2
GO:0021984	adenohypophysis development	0.060941181	0.060990853	2
GO:0030002	cellular anion homeostasis	0.060941181	0.060990853	2
GO:0030320	cellular monovalent inorganic anion homeostasis	0.060941181	0.060990853	2
GO:0030656	regulation of vitamin metabolic process	0.060941181	0.060990853	2

GO:00310<br>53	primary miRNA processing	0.0609411<br>81	0.0609908<br>53	2
GO:00311<br>15	negative regulation of microtubule polymerization	0.0609411<br>81	0.0609908<br>53	2
GO:00319<br>53	negative regulation of protein autophosphorylation	0.0609411<br>81	0.0609908<br>53	2
GO:00322<br>30	positive regulation of synaptic transmission, GABAergic	0.0609411<br>81	0.0609908<br>53	2
GO:00323<br>52	positive regulation of hormone metabolic process	0.0609411<br>81	0.0609908<br>53	2
GO:00331<br>27	regulation of histone phosphorylation	0.0609411<br>81	0.0609908<br>53	2
GO:00331<br>45	positive regulation of intracellular steroid hormone receptor signaling pathway	0.0609411<br>81	0.0609908<br>53	2
GO:00426<br>35	positive regulation of hair cycle	0.0609411<br>81	0.0609908<br>53	2
GO:00435<br>68	positive regulation of insulin-like growth factor receptor signaling pathway	0.0609411<br>81	0.0609908<br>53	2
GO:00439<br>70	histone H3-K9 acetylation	0.0609411<br>81	0.0609908<br>53	2
GO:00456<br>02	negative regulation of endothelial cell differentiation	0.0609411<br>81	0.0609908<br>53	2
GO:00457<br>41	positive regulation of epidermal growth factor-activated receptor activity	0.0609411<br>81	0.0609908<br>53	2
GO:00480<br>96	chromatin-mediated maintenance of transcription	0.0609411<br>81	0.0609908<br>53	2
GO:00519<br>88	regulation of attachment of spindle microtubules to kinetochore	0.0609411<br>81	0.0609908<br>53	2
GO:00606<br>01	lateral sprouting from an epithelium	0.0609411<br>81	0.0609908<br>53	2
GO:00610<br>29	eyelid development in camera-type eye	0.0609411<br>81	0.0609908<br>53	2
GO:00610<br>43	regulation of vascular wound healing	0.0609411<br>81	0.0609908<br>53	2
GO:00719<br>41	nitrogen cycle metabolic process	0.0609411<br>81	0.0609908<br>53	2
GO:00726<br>83	T cell extravasation	0.0609411<br>81	0.0609908<br>53	2
GO:00971<br>52	mesenchymal cell apoptotic process	0.0609411<br>81	0.0609908<br>53	2
GO:00975<br>31	mast cell migration	0.0609411<br>81	0.0609908<br>53	2
GO:00989<br>03	regulation of membrane repolarization during action potential	0.0609411<br>81	0.0609908<br>53	2

GO:1900103	positive regulation of endoplasmic reticulum unfolded protein response	0.060941181	0.060990853	2
GO:1901096	regulation of autophagosome maturation	0.060941181	0.060990853	2
GO:1901387	positive regulation of voltage-gated calcium channel activity	0.060941181	0.060990853	2
GO:1903651	positive regulation of cytoplasmic transport	0.060941181	0.060990853	2
GO:2000121	regulation of removal of superoxide radicals	0.060941181	0.060990853	2
GO:0050821	protein stabilization	0.061840289	0.061856482	2
GO:0048864	stem cell development	0.062192621	0.062174537	2
GO:0006163	purine nucleotide metabolic process	0.062622551	0.062569772	2
GO:0010769	regulation of cell morphogenesis involved in differentiation	0.063036319	0.062948433	2
GO:0007369	gastrulation	0.063428129	0.063199147	2
GO:0010565	regulation of cellular ketone metabolic process	0.063428129	0.063199147	2
GO:0006767	water-soluble vitamin metabolic process	0.063496944	0.063199147	2
GO:0032092	positive regulation of protein binding	0.063496944	0.063199147	2
GO:0070509	calcium ion import	0.063496944	0.063199147	2
GO:0072384	organelle transport along microtubule	0.063496944	0.063199147	2
GO:0070507	regulation of microtubule cytoskeleton organization	0.064229144	0.063661044	2
GO:0014910	regulation of smooth muscle cell migration	0.064810541	0.063661044	2
GO:0042509	regulation of tyrosine phosphorylation of STAT protein	0.064810541	0.063661044	2
GO:0045445	myoblast differentiation	0.064810541	0.063661044	2
GO:0050772	positive regulation of axonogenesis	0.064810541	0.063661044	2
GO:0000054	ribosomal subunit export from nucleus	0.065473847	0.063661044	2
GO:0007100	mitotic centrosome separation	0.065473847	0.063661044	2

GO:0007512	adult heart development	0.065473847	0.063661044	2
GO:0009214	cyclic nucleotide catabolic process	0.065473847	0.063661044	2
GO:0010248	establishment or maintenance of transmembrane electrochemical gradient	0.065473847	0.063661044	2
GO:0010752	regulation of cGMP-mediated signaling	0.065473847	0.063661044	2
GO:0010917	negative regulation of mitochondrial membrane potential	0.065473847	0.063661044	2
GO:0014854	response to inactivity	0.065473847	0.063661044	2
GO:0014874	response to stimulus involved in regulation of muscle adaptation	0.065473847	0.063661044	2
GO:0019896	axonal transport of mitochondrion	0.065473847	0.063661044	2
GO:0031282	regulation of guanylate cyclase activity	0.065473847	0.063661044	2
GO:0032042	mitochondrial DNA metabolic process	0.065473847	0.063661044	2
GO:0032310	prostaglandin secretion	0.065473847	0.063661044	2
GO:0033604	negative regulation of catecholamine secretion	0.065473847	0.063661044	2
GO:0033750	ribosome localization	0.065473847	0.063661044	2
GO:0034356	NAD biosynthesis via nicotinamide riboside salvage pathway	0.065473847	0.063661044	2
GO:0035112	genitalia morphogenesis	0.065473847	0.063661044	2
GO:0042659	regulation of cell fate specification	0.065473847	0.063661044	2
GO:0043922	negative regulation by host of viral transcription	0.065473847	0.063661044	2
GO:0046007	negative regulation of activated T cell proliferation	0.065473847	0.063661044	2
GO:0047484	regulation of response to osmotic stress	0.065473847	0.063661044	2
GO:0051547	regulation of keratinocyte migration	0.065473847	0.063661044	2
GO:0051709	regulation of killing of cells of other organism	0.065473847	0.063661044	2
GO:0055064	chloride ion homeostasis	0.065473847	0.063661044	2

GO:0060452	positive regulation of cardiac muscle contraction	0.065473847	0.063661044	2
GO:0070234	positive regulation of T cell apoptotic process	0.065473847	0.063661044	2
GO:0070431	nucleotide-binding oligomerization domain containing 2 signaling pathway	0.065473847	0.063661044	2
GO:0071236	cellular response to antibiotic	0.065473847	0.063661044	2
GO:0072425	signal transduction involved in G2 DNA damage checkpoint	0.065473847	0.063661044	2
GO:0090231	regulation of spindle checkpoint	0.065473847	0.063661044	2
GO:0090266	regulation of mitotic cell cycle spindle assembly checkpoint	0.065473847	0.063661044	2
GO:1901841	regulation of high voltage-gated calcium channel activity	0.065473847	0.063661044	2
GO:1902894	negative regulation of pri-miRNA transcription by RNA polymerase II	0.065473847	0.063661044	2
GO:1903504	regulation of mitotic spindle checkpoint	0.065473847	0.063661044	2
GO:1903894	regulation of IRE1-mediated unfolded protein response	0.065473847	0.063661044	2
GO:1904748	regulation of apoptotic process involved in development	0.065473847	0.063661044	2
GO:1905064	negative regulation of vascular associated smooth muscle cell differentiation	0.065473847	0.063661044	2
GO:2001279	regulation of unsaturated fatty acid biosynthetic process	0.065473847	0.063661044	2
GO:0006413	translational initiation	0.065845278	0.063953461	2
GO:0048015	phosphatidylinositol-mediated signaling	0.065845278	0.063953461	2
GO:0009060	aerobic respiration	0.066133292	0.06406127	2
GO:0032436	positive regulation of proteasomal ubiquitin-dependent protein catabolic process	0.066133292	0.06406127	2
GO:0051453	regulation of intracellular pH	0.066133292	0.06406127	2
GO:0051781	positive regulation of cell division	0.066133292	0.06406127	2
GO:0060420	regulation of heart growth	0.066133292	0.06406127	2
GO:0006417	regulation of translation	0.066550945	0.064431345	2

GO:0014068	positive regulation of phosphatidylinositol 3-kinase signaling	0.067465083	0.065176878	2
GO:0032410	negative regulation of transporter activity	0.067465083	0.065176878	2
GO:0032755	positive regulation of interleukin-6 production	0.067465083	0.065176878	2
GO:0046470	phosphatidylcholine metabolic process	0.067465083	0.065176878	2
GO:0007260	tyrosine phosphorylation of STAT protein	0.068805797	0.066197484	2
GO:0014033	neural crest cell differentiation	0.068805797	0.066197484	2
GO:1901019	regulation of calcium ion transmembrane transporter activity	0.068805797	0.066197484	2
GO:0009566	fertilization	0.069133457	0.066197484	2
GO:0048017	inositol lipid-mediated signaling	0.069133457	0.066197484	2
GO:0071897	DNA biosynthetic process	0.069133457	0.066197484	2
GO:0009749	response to glucose	0.069967044	0.066197484	2
GO:0051216	cartilage development	0.069967044	0.066197484	2
GO:0006122	mitochondrial electron transport, ubiquinol to cytochrome c	0.069984874	0.066197484	2
GO:0009404	toxin metabolic process	0.069984874	0.066197484	2
GO:0010763	positive regulation of fibroblast migration	0.069984874	0.066197484	2
GO:0020027	hemoglobin metabolic process	0.069984874	0.066197484	2
GO:0021924	cell proliferation in external granule layer	0.069984874	0.066197484	2
GO:0021930	cerebellar granule cell precursor proliferation	0.069984874	0.066197484	2
GO:0030238	male sex determination	0.069984874	0.066197484	2
GO:0032252	secretory granule localization	0.069984874	0.066197484	2
GO:0033689	negative regulation of osteoblast proliferation	0.069984874	0.066197484	2
GO:0035635	entry of bacterium into host cell	0.069984874	0.066197484	2

GO:0035745	T-helper 2 cell cytokine production	0.069984874	0.066197484	2
GO:0042362	fat-soluble vitamin biosynthetic process	0.069984874	0.066197484	2
GO:0043374	CD8-positive, alpha-beta T cell differentiation	0.069984874	0.066197484	2
GO:0045475	locomotor rhythm	0.069984874	0.066197484	2
GO:0045779	negative regulation of bone resorption	0.069984874	0.066197484	2
GO:0045837	negative regulation of membrane potential	0.069984874	0.066197484	2
GO:0046479	glycosphingolipid catabolic process	0.069984874	0.066197484	2
GO:0050746	regulation of lipoprotein metabolic process	0.069984874	0.066197484	2
GO:0050862	positive regulation of T cell receptor signaling pathway	0.069984874	0.066197484	2
GO:0051299	centrosome separation	0.069984874	0.066197484	2
GO:0060134	prepulse inhibition	0.069984874	0.066197484	2
GO:0060347	heart trabecula formation	0.069984874	0.066197484	2
GO:0060841	venous blood vessel development	0.069984874	0.066197484	2
GO:0070885	negative regulation of calcineurin-NFAT signaling cascade	0.069984874	0.066197484	2
GO:0071428	rRNA-containing ribonucleoprotein complex export from nucleus	0.069984874	0.066197484	2
GO:0071801	regulation of podosome assembly	0.069984874	0.066197484	2
GO:0072567	chemokine (C-X-C motif) ligand 2 production	0.069984874	0.066197484	2
GO:0090594	inflammatory response to wounding	0.069984874	0.066197484	2
GO:0097202	activation of cysteine-type endopeptidase activity	0.069984874	0.066197484	2
GO:0106057	negative regulation of calcineurin-mediated signaling	0.069984874	0.066197484	2
GO:1903960	negative regulation of anion transmembrane transport	0.069984874	0.066197484	2
GO:2000341	regulation of chemokine (C-X-C motif) ligand 2 production	0.069984874	0.066197484	2

GO:0046427	positive regulation of receptor signaling pathway via JAK-STAT	0.070155319	0.066324036	2
GO:0007626	locomotory behavior	0.070805212	0.066903482	2
GO:0042475	odontogenesis of dentin-containing tooth	0.071513536	0.067467081	2
GO:0060333	interferon-gamma-mediated signaling pathway	0.071513536	0.067467081	2
GO:0097306	cellular response to alcohol	0.071513536	0.067467081	2
GO:1903321	negative regulation of protein modification by small protein conjugation or removal	0.072880335	0.068720713	2
GO:0002683	negative regulation of immune system process	0.073223927	0.06893064	2
GO:0002221	pattern recognition receptor signaling pathway	0.073347006	0.06893064	2
GO:0007179	transforming growth factor beta receptor signaling pathway	0.074203305	0.06893064	2
GO:0006898	receptor-mediated endocytosis	0.074206686	0.06893064	2
GO:0014909	smooth muscle cell migration	0.074255602	0.06893064	2
GO:0051702	interaction with symbiont	0.074255602	0.06893064	2
GO:0061097	regulation of protein tyrosine kinase activity	0.074255602	0.06893064	2
GO:0072080	nephron tubule development	0.074255602	0.06893064	2
GO:1904894	positive regulation of receptor signaling pathway via STAT	0.074255602	0.06893064	2
GO:0001780	neutrophil homeostasis	0.074474364	0.06893064	2
GO:0002295	T-helper cell lineage commitment	0.074474364	0.06893064	2
GO:0002830	positive regulation of type 2 immune response	0.074474364	0.06893064	2
GO:0006098	pentose-phosphate shunt	0.074474364	0.06893064	2
GO:0010225	response to UV-C	0.074474364	0.06893064	2
GO:0014061	regulation of norepinephrine secretion	0.074474364	0.06893064	2
GO:0017014	protein nitrosylation	0.074474364	0.06893064	2

GO:0018119	peptidyl-cysteine S-nitrosylation	0.074474364	0.06893064	2
GO:0019511	peptidyl-proline hydroxylation	0.074474364	0.06893064	2
GO:0021534	cell proliferation in hindbrain	0.074474364	0.06893064	2
GO:0033033	negative regulation of myeloid cell apoptotic process	0.074474364	0.06893064	2
GO:0034116	positive regulation of heterotypic cell-cell adhesion	0.074474364	0.06893064	2
GO:0034374	low-density lipoprotein particle remodeling	0.074474364	0.06893064	2
GO:0045838	positive regulation of membrane potential	0.074474364	0.06893064	2
GO:0045989	positive regulation of striated muscle contraction	0.074474364	0.06893064	2
GO:0046325	negative regulation of glucose import	0.074474364	0.06893064	2
GO:0060572	morphogenesis of an epithelial bud	0.074474364	0.06893064	2
GO:0061450	trophoblast cell migration	0.074474364	0.06893064	2
GO:0070886	positive regulation of calcineurin-NFAT signaling cascade	0.074474364	0.06893064	2
GO:0071380	cellular response to prostaglandin E stimulus	0.074474364	0.06893064	2
GO:0099170	postsynaptic modulation of chemical synaptic transmission	0.074474364	0.06893064	2
GO:0106058	positive regulation of calcineurin-mediated signaling	0.074474364	0.06893064	2
GO:1901163	regulation of trophoblast cell migration	0.074474364	0.06893064	2
GO:1902166	negative regulation of intrinsic apoptotic signaling pathway in response to DNA damage by p53 class mediator	0.074474364	0.06893064	2
GO:1902514	regulation of calcium ion transmembrane transport via high voltage-gated calcium channel	0.074474364	0.06893064	2
GO:1905050	positive regulation of metallopeptidase activity	0.074474364	0.06893064	2
GO:2001212	regulation of vasculogenesis	0.074474364	0.06893064	2
GO:0006473	protein acetylation	0.075064089	0.069440948	2
GO:0010660	regulation of muscle cell apoptotic process	0.075639227	0.069759017	2

GO:0030641	regulation of cellular pH	0.075639227	0.069759017	2
GO:0034308	primary alcohol metabolic process	0.075639227	0.069759017	2
GO:0045778	positive regulation of ossification	0.075639227	0.069759017	2
GO:0060993	kidney morphogenesis	0.075639227	0.069759017	2
GO:1905954	positive regulation of lipid localization	0.075639227	0.069759017	2
GO:0051168	nuclear export	0.07592934	0.069990903	2
GO:0050709	negative regulation of protein secretion	0.077031099	0.070862098	2
GO:0061326	renal tubule development	0.077031099	0.070862098	2
GO:0070167	regulation of biomineral tissue development	0.077031099	0.070862098	2
GO:0110149	regulation of biomineralization	0.077031099	0.070862098	2
GO:0035637	multicellular organismal signaling	0.077673159	0.071416432	2
GO:0072521	purine-containing compound metabolic process	0.078052178	0.07167297	2
GO:0050866	negative regulation of cell activation	0.07855169	0.07167297	2
GO:1901343	negative regulation of vasculature development	0.07855169	0.07167297	2
GO:0003184	pulmonary valve morphogenesis	0.078942419	0.07167297	2
GO:0008340	determination of adult lifespan	0.078942419	0.07167297	2
GO:0009299	mRNA transcription	0.078942419	0.07167297	2
GO:0010715	regulation of extracellular matrix disassembly	0.078942419	0.07167297	2
GO:0015732	prostaglandin transport	0.078942419	0.07167297	2
GO:0019377	glycolipid catabolic process	0.078942419	0.07167297	2
GO:0030730	sequestering of triglyceride	0.078942419	0.07167297	2
GO:0030889	negative regulation of B cell proliferation	0.078942419	0.07167297	2

GO:0030949	positive regulation of vascular endothelial growth factor receptor signaling pathway	0.078942419	0.07167297	2
GO:0032682	negative regulation of chemokine production	0.078942419	0.07167297	2
GO:0032966	negative regulation of collagen biosynthetic process	0.078942419	0.07167297	2
GO:0035729	cellular response to hepatocyte growth factor stimulus	0.078942419	0.07167297	2
GO:0043923	positive regulation by host of viral transcription	0.078942419	0.07167297	2
GO:0044539	long-chain fatty acid import into cell	0.078942419	0.07167297	2
GO:0046068	cGMP metabolic process	0.078942419	0.07167297	2
GO:0046851	negative regulation of bone remodeling	0.078942419	0.07167297	2
GO:0048243	norepinephrine secretion	0.078942419	0.07167297	2
GO:0090141	positive regulation of mitochondrial fission	0.078942419	0.07167297	2
GO:0090185	negative regulation of kidney development	0.078942419	0.07167297	2
GO:0140354	lipid import into cell	0.078942419	0.07167297	2
GO:1903209	positive regulation of oxidative stress-induced cell death	0.078942419	0.07167297	1
GO:1905331	negative regulation of morphogenesis of an epithelium	0.078942419	0.07167297	1
GO:0006476	protein deacetylation	0.079839148	0.072305724	1
GO:0042775	mitochondrial ATP synthesis coupled electron transport	0.079839148	0.072305724	1
GO:0050764	regulation of phagocytosis	0.079839148	0.072305724	1
GO:0098659	inorganic cation import across plasma membrane	0.079839148	0.072305724	1
GO:0099587	inorganic ion import across plasma membrane	0.079839148	0.072305724	1
GO:0035303	regulation of dephosphorylation	0.080321892	0.072706528	1
GO:0008544	epidermis development	0.080807695	0.073109699	1
GO:0045216	cell-cell junction organization	0.081213525	0.073294605	1

GO:0001657	ureteric bud development	0.081255107	0.073294605	1
GO:0010657	muscle cell apoptotic process	0.081255107	0.073294605	1
GO:0014020	primary neural tube formation	0.081255107	0.073294605	1
GO:0042773	ATP synthesis coupled electron transport	0.081255107	0.073294605	1
GO:0050810	regulation of steroid biosynthetic process	0.081255107	0.073294605	1
GO:0002703	regulation of leukocyte mediated immunity	0.082109484	0.073893464	1
GO:0043473	pigmentation	0.08267888	0.073893464	1
GO:0050830	defense response to Gram-positive bacterium	0.08267888	0.073893464	1
GO:0072163	mesonephric epithelium development	0.08267888	0.073893464	1
GO:0072164	mesonephric tubule development	0.08267888	0.073893464	1
GO:0019058	viral life cycle	0.082860216	0.073893464	1
GO:0006643	membrane lipid metabolic process	0.08300975	0.073893464	1
GO:0051701	interaction with host	0.08300975	0.073893464	1
GO:0006349	regulation of gene expression by genetic imprinting	0.083389139	0.073893464	1
GO:0006677	glycosylceramide metabolic process	0.083389139	0.073893464	1
GO:0006740	NADPH regeneration	0.083389139	0.073893464	1
GO:0010523	negative regulation of calcium ion transport into cytosol	0.083389139	0.073893464	1
GO:0010713	negative regulation of collagen metabolic process	0.083389139	0.073893464	1
GO:0031293	membrane protein intracellular domain proteolysis	0.083389139	0.073893464	1
GO:0032740	positive regulation of interleukin-17 production	0.083389139	0.073893464	1
GO:0032780	negative regulation of ATPase activity	0.083389139	0.073893464	1
GO:0033194	response to hydroperoxide	0.083389139	0.073893464	1

GO:0035330	regulation of hippo signaling	0.083389139	0.073893464	1
GO:0043217	myelin maintenance	0.083389139	0.073893464	1
GO:0045187	regulation of circadian sleep/wake cycle, sleep	0.083389139	0.073893464	1
GO:0046885	regulation of hormone biosynthetic process	0.083389139	0.073893464	1
GO:0051895	negative regulation of focal adhesion assembly	0.083389139	0.073893464	1
GO:0060004	reflex	0.083389139	0.073893464	1
GO:0060033	anatomical structure regression	0.083389139	0.073893464	1
GO:0060457	negative regulation of digestive system process	0.083389139	0.073893464	1
GO:0060977	coronary vasculature morphogenesis	0.083389139	0.073893464	1
GO:0061298	retina vasculature development in camera-type eye	0.083389139	0.073893464	1
GO:0061577	calcium ion transmembrane transport via high voltage-gated calcium channel	0.083389139	0.073893464	1
GO:0071318	cellular response to ATP	0.083389139	0.073893464	1
GO:0071605	monocyte chemotactic protein-1 production	0.083389139	0.073893464	1
GO:0071637	regulation of monocyte chemotactic protein-1 production	0.083389139	0.073893464	1
GO:0090190	positive regulation of branching involved in ureteric bud morphogenesis	0.083389139	0.073893464	1
GO:0150118	negative regulation of cell-substrate junction organization	0.083389139	0.073893464	1
GO:1900221	regulation of amyloid-beta clearance	0.083389139	0.073893464	1
GO:1901524	regulation of mitophagy	0.083389139	0.073893464	1
GO:1902165	regulation of intrinsic apoptotic signaling pathway in response to DNA damage by p53 class mediator	0.083389139	0.073893464	1
GO:0090150	establishment of protein localization to membrane	0.083545818	0.073996064	1
GO:0016051	carbohydrate biosynthetic process	0.083914303	0.074286068	1
GO:0006672	ceramide metabolic process	0.084110361	0.074386845	1

GO:0050848	regulation of calcium-mediated signaling	0.084110361	0.074386845	1
GO:0002792	negative regulation of peptide secretion	0.085549443	0.075475117	1
GO:0010522	regulation of calcium ion transport into cytosol	0.085549443	0.075475117	1
GO:0015908	fatty acid transport	0.085549443	0.075475117	1
GO:0042102	positive regulation of T cell proliferation	0.085549443	0.075475117	1
GO:0060191	regulation of lipase activity	0.085549443	0.075475117	1
GO:0051651	maintenance of location in cell	0.086653475	0.076210195	1
GO:0055024	regulation of cardiac muscle tissue development	0.086996023	0.076210195	1
GO:2000060	positive regulation of ubiquitin-dependent protein catabolic process	0.086996023	0.076210195	1
GO:0001696	gastric acid secretion	0.087814627	0.076210195	1
GO:0002076	osteoblast development	0.087814627	0.076210195	1
GO:0002922	positive regulation of humoral immune response	0.087814627	0.076210195	1
GO:0003159	morphogenesis of an endothelium	0.087814627	0.076210195	1
GO:0010663	positive regulation of striated muscle cell apoptotic process	0.087814627	0.076210195	1
GO:0010666	positive regulation of cardiac muscle cell apoptotic process	0.087814627	0.076210195	1
GO:0010759	positive regulation of macrophage chemotaxis	0.087814627	0.076210195	1
GO:0019054	modulation by virus of host cellular process	0.087814627	0.076210195	1
GO:0030011	maintenance of cell polarity	0.087814627	0.076210195	1
GO:0032026	response to magnesium ion	0.087814627	0.076210195	1
GO:0032095	regulation of response to food	0.087814627	0.076210195	1
GO:0032930	positive regulation of superoxide anion generation	0.087814627	0.076210195	1
GO:0035728	response to hepatocyte growth factor	0.087814627	0.076210195	1

GO:0043373	CD4-positive, alpha-beta T cell lineage commitment	0.087814627	0.076210195	1
GO:0045091	regulation of single stranded viral RNA replication via double stranded DNA intermediate	0.087814627	0.076210195	1
GO:0045780	positive regulation of bone resorption	0.087814627	0.076210195	1
GO:0046485	ether lipid metabolic process	0.087814627	0.076210195	1
GO:0046852	positive regulation of bone remodeling	0.087814627	0.076210195	1
GO:0048245	eosinophil chemotaxis	0.087814627	0.076210195	1
GO:0048490	anterograde synaptic vesicle transport	0.087814627	0.076210195	1
GO:0051546	keratinocyte migration	0.087814627	0.076210195	1
GO:0060602	branch elongation of an epithelium	0.087814627	0.076210195	1
GO:0061154	endothelial tube morphogenesis	0.087814627	0.076210195	1
GO:0070233	negative regulation of T cell apoptotic process	0.087814627	0.076210195	1
GO:0071800	podosome assembly	0.087814627	0.076210195	1
GO:0097623	potassium ion export across plasma membrane	0.087814627	0.076210195	1
GO:0098915	membrane repolarization during ventricular cardiac muscle cell action potential	0.087814627	0.076210195	1
GO:0099514	synaptic vesicle cytoskeletal transport	0.087814627	0.076210195	1
GO:0099517	synaptic vesicle transport along microtubule	0.087814627	0.076210195	1
GO:2000647	negative regulation of stem cell proliferation	0.087814627	0.076210195	1
GO:2000696	regulation of epithelial cell differentiation involved in kidney development	0.087814627	0.076210195	1
GO:0001823	mesonephros development	0.088449995	0.076577961	1
GO:0006885	regulation of pH	0.088449995	0.076577961	1
GO:0030593	neutrophil chemotaxis	0.088449995	0.076577961	1
GO:0070498	interleukin-1-mediated signaling pathway	0.088449995	0.076577961	1

GO:1901890	positive regulation of cell junction assembly	0.088449995	0.076577961	1
GO:0030178	negative regulation of Wnt signaling pathway	0.089430484	0.077389817	1
GO:0002367	cytokine production involved in immune response	0.089911258	0.077657306	1
GO:0003300	cardiac muscle hypertrophy	0.089911258	0.077657306	1
GO:0010923	negative regulation of phosphatase activity	0.089911258	0.077657306	1
GO:0048525	negative regulation of viral process	0.089911258	0.077657306	1
GO:0002824	positive regulation of adaptive immune response based on somatic recombination of immune receptors built from immunoglobulin superfamily domains	0.09137971	0.078400411	1
GO:0007631	feeding behavior	0.09137971	0.078400411	1
GO:0044070	regulation of anion transport	0.09137971	0.078400411	1
GO:0062014	negative regulation of small molecule metabolic process	0.09137971	0.078400411	1
GO:0002320	lymphoid progenitor cell differentiation	0.092218981	0.078400411	1
GO:0002544	chronic inflammatory response	0.092218981	0.078400411	1
GO:0002689	negative regulation of leukocyte chemotaxis	0.092218981	0.078400411	1
GO:0002726	positive regulation of T cell cytokine production	0.092218981	0.078400411	1
GO:0002902	regulation of B cell apoptotic process	0.092218981	0.078400411	1
GO:0003323	type B pancreatic cell development	0.092218981	0.078400411	1
GO:0006525	arginine metabolic process	0.092218981	0.078400411	1
GO:0007620	copulation	0.092218981	0.078400411	1
GO:0010042	response to manganese ion	0.092218981	0.078400411	1
GO:0010875	positive regulation of cholesterol efflux	0.092218981	0.078400411	1
GO:0031290	retinal ganglion cell axon guidance	0.092218981	0.078400411	1
GO:0031998	regulation of fatty acid beta-oxidation	0.092218981	0.078400411	1

GO:0032332	positive regulation of chondrocyte differentiation	0.092218981	0.078400411	1
GO:0032793	positive regulation of CREB transcription factor activity	0.092218981	0.078400411	1
GO:0034138	toll-like receptor 3 signaling pathway	0.092218981	0.078400411	1
GO:0036303	lymph vessel morphogenesis	0.092218981	0.078400411	1
GO:0039692	single stranded viral RNA replication via double stranded DNA intermediate	0.092218981	0.078400411	1
GO:0043950	positive regulation of cAMP-mediated signaling	0.092218981	0.078400411	1
GO:0060353	regulation of cell adhesion molecule production	0.092218981	0.078400411	1
GO:0060546	negative regulation of necroptotic process	0.092218981	0.078400411	1
GO:0062099	negative regulation of programmed necrotic cell death	0.092218981	0.078400411	1
GO:0071636	positive regulation of transforming growth factor beta production	0.092218981	0.078400411	1
GO:0071731	response to nitric oxide	0.092218981	0.078400411	1
GO:0086014	atrial cardiac muscle cell action potential	0.092218981	0.078400411	1
GO:0086026	atrial cardiac muscle cell to AV node cell signaling	0.092218981	0.078400411	1
GO:0086066	atrial cardiac muscle cell to AV node cell communication	0.092218981	0.078400411	1
GO:0097709	connective tissue replacement	0.092218981	0.078400411	1
GO:0098703	calcium ion import across plasma membrane	0.092218981	0.078400411	1
GO:1901741	positive regulation of myoblast fusion	0.092218981	0.078400411	1
GO:0017157	regulation of exocytosis	0.092244765	0.078400411	1
GO:0001841	neural tube formation	0.092855247	0.078734361	1
GO:0002708	positive regulation of lymphocyte mediated immunity	0.092855247	0.078734361	1
GO:0014812	muscle cell migration	0.092855247	0.078734361	1
GO:0046632	alpha-beta T cell differentiation	0.092855247	0.078734361	1

GO:0046928	regulation of neurotransmitter secretion	0.092855247	0.078734361	1
GO:0002699	positive regulation of immune effector process	0.093191042	0.078982079	1
GO:0014897	striated muscle hypertrophy	0.094337771	0.079804448	1
GO:0030148	sphingolipid biosynthetic process	0.094337771	0.079804448	1
GO:0035601	protein deacylation	0.094337771	0.079804448	1
GO:0043266	regulation of potassium ion transport	0.094337771	0.079804448	1
GO:0045665	negative regulation of neuron differentiation	0.095095745	0.080146412	1
GO:0007178	transmembrane receptor protein serine/threonine kinase signaling pathway	0.095626887	0.080146412	1
GO:0001523	retinoid metabolic process	0.095827181	0.080146412	1
GO:0002062	chondrocyte differentiation	0.095827181	0.080146412	1
GO:1902750	negative regulation of cell cycle G2/M phase transition	0.095827181	0.080146412	1
GO:0001502	cartilage condensation	0.096602303	0.080146412	1
GO:0002029	desensitization of G protein-coupled receptor signaling pathway	0.096602303	0.080146412	1
GO:0003177	pulmonary valve development	0.096602303	0.080146412	1
GO:0006123	mitochondrial electron transport, cytochrome c to oxygen	0.096602303	0.080146412	1
GO:0006662	glycerol ether metabolic process	0.096602303	0.080146412	1
GO:0009110	vitamin biosynthetic process	0.096602303	0.080146412	1
GO:0009713	catechol-containing compound biosynthetic process	0.096602303	0.080146412	1
GO:0010829	negative regulation of glucose transmembrane transport	0.096602303	0.080146412	1
GO:0010893	positive regulation of steroid biosynthetic process	0.096602303	0.080146412	1
GO:0014821	phasic smooth muscle contraction	0.096602303	0.080146412	1
GO:0019370	leukotriene biosynthetic process	0.096602303	0.080146412	1

GO:0019646	aerobic electron transport chain	0.096602303	0.080146412	1
GO:0022401	negative adaptation of signaling pathway	0.096602303	0.080146412	1
GO:0030502	negative regulation of bone mineralization	0.096602303	0.080146412	1
GO:0035357	peroxisome proliferator activated receptor signaling pathway	0.096602303	0.080146412	1
GO:0042423	catecholamine biosynthetic process	0.096602303	0.080146412	1
GO:0043651	linoleic acid metabolic process	0.096602303	0.080146412	1
GO:0046514	ceramide catabolic process	0.096602303	0.080146412	1
GO:0046716	muscle cell cellular homeostasis	0.096602303	0.080146412	1
GO:0051797	regulation of hair follicle development	0.096602303	0.080146412	1
GO:0060065	uterus development	0.096602303	0.080146412	1
GO:0060713	labyrinthine layer morphogenesis	0.096602303	0.080146412	1
GO:0060716	labyrinthine layer blood vessel development	0.096602303	0.080146412	1
GO:0061213	positive regulation of mesonephros development	0.096602303	0.080146412	1
GO:0071498	cellular response to fluid shear stress	0.096602303	0.080146412	1
GO:0072574	hepatocyte proliferation	0.096602303	0.080146412	1
GO:0072575	epithelial cell proliferation involved in liver morphogenesis	0.096602303	0.080146412	1
GO:0090201	negative regulation of release of cytochrome c from mitochondria	0.096602303	0.080146412	1
GO:0090280	positive regulation of calcium ion import	0.096602303	0.080146412	1
GO:1902656	calcium ion import into cytosol	0.096602303	0.080146412	1
GO:1903055	positive regulation of extracellular matrix organization	0.096602303	0.080146412	1
GO:1903204	negative regulation of oxidative stress-induced neuron death	0.096602303	0.080146412	1
GO:1903599	positive regulation of autophagy of mitochondrion	0.096602303	0.080146412	1

GO:1903978	regulation of microglial cell activation	0.096602303	0.080146412	1
GO:1904886	beta-catenin destruction complex disassembly	0.096602303	0.080146412	1
GO:2000047	regulation of cell-cell adhesion mediated by cadherin	0.096602303	0.080146412	1
GO:2000269	regulation of fibroblast apoptotic process	0.096602303	0.080146412	1
GO:0002286	T cell activation involved in immune response	0.097323377	0.080633741	1
GO:0014896	muscle hypertrophy	0.097323377	0.080633741	1
GO:2000278	regulation of DNA biosynthetic process	0.097323377	0.080633741	1
GO:0002821	positive regulation of adaptive immune response	0.098826262	0.081729214	1
GO:0006664	glycolipid metabolic process	0.098826262	0.081729214	1
GO:0072009	nephron epithelium development	0.098826262	0.081729214	1
GO:1901800	positive regulation of proteasomal protein catabolic process	0.098826262	0.081729214	1
GO:0050807	regulation of synapse organization	0.098953142	0.081796759	1
GO:0035305	negative regulation of dephosphorylation	0.100335739	0.082368327	1
GO:0051149	positive regulation of muscle cell differentiation	0.100335739	0.082368327	1
GO:0098732	macromolecule deacylation	0.100335739	0.082368327	1
GO:1903008	organelle disassembly	0.100335739	0.082368327	1
GO:1903509	liposaccharide metabolic process	0.100335739	0.082368327	1
GO:0002089	lens morphogenesis in camera-type eye	0.10096469	0.082368327	1
GO:0002313	mature B cell differentiation involved in immune response	0.10096469	0.082368327	1
GO:0005980	glycogen catabolic process	0.10096469	0.082368327	1
GO:0006067	ethanol metabolic process	0.10096469	0.082368327	1
GO:0006312	mitotic recombination	0.10096469	0.082368327	1

GO:0007342	fusion of sperm to egg plasma membrane involved in single fertilization	0.10096469	0.082368327	1
GO:0023058	adaptation of signaling pathway	0.10096469	0.082368327	1
GO:0032928	regulation of superoxide anion generation	0.10096469	0.082368327	1
GO:0034104	negative regulation of tissue remodeling	0.10096469	0.082368327	1
GO:0035024	negative regulation of Rho protein signal transduction	0.10096469	0.082368327	1
GO:0035584	calcium-mediated signaling using intracellular calcium source	0.10096469	0.082368327	1
GO:0042359	vitamin D metabolic process	0.10096469	0.082368327	1
GO:0042749	regulation of circadian sleep/wake cycle	0.10096469	0.082368327	1
GO:0045655	regulation of monocyte differentiation	0.10096469	0.082368327	1
GO:0045663	positive regulation of myoblast differentiation	0.10096469	0.082368327	1
GO:0046058	cAMP metabolic process	0.10096469	0.082368327	1
GO:0050802	circadian sleep/wake cycle, sleep	0.10096469	0.082368327	1
GO:0055093	response to hyperoxia	0.10096469	0.082368327	1
GO:0060352	cell adhesion molecule production	0.10096469	0.082368327	1
GO:0071157	negative regulation of cell cycle arrest	0.10096469	0.082368327	1
GO:0071379	cellular response to prostaglandin stimulus	0.10096469	0.082368327	1
GO:0072576	liver morphogenesis	0.10096469	0.082368327	1
GO:0090189	regulation of branching involved in ureteric bud morphogenesis	0.10096469	0.082368327	1
GO:1901739	regulation of myoblast fusion	0.10096469	0.082368327	1
GO:0006275	regulation of DNA replication	0.101851709	0.083054524	1
GO:0030098	lymphocyte differentiation	0.102340933	0.083415867	1
GO:0002223	stimulatory C-type lectin receptor signaling pathway	0.103374078	0.084220025	1

GO:0003279	cardiac septum development	0.104902751	0.084500609	1
GO:0016101	diterpenoid metabolic process	0.104902751	0.084500609	1
GO:0030004	cellular monovalent inorganic cation homeostasis	0.104902751	0.084500609	1
GO:0098693	regulation of synaptic vesicle cycle	0.104902751	0.084500609	1
GO:0000002	mitochondrial genome maintenance	0.105306242	0.084500609	1
GO:0001919	regulation of receptor recycling	0.105306242	0.084500609	1
GO:0006359	regulation of transcription by RNA polymerase III	0.105306242	0.084500609	1
GO:0006582	melanin metabolic process	0.105306242	0.084500609	1
GO:0007274	neuromuscular synaptic transmission	0.105306242	0.084500609	1
GO:0007413	axonal fasciculation	0.105306242	0.084500609	1
GO:0007617	mating behavior	0.105306242	0.084500609	1
GO:0009251	glucan catabolic process	0.105306242	0.084500609	1
GO:0010226	response to lithium ion	0.105306242	0.084500609	1
GO:0010288	response to lead ion	0.105306242	0.084500609	1
GO:0030539	male genitalia development	0.105306242	0.084500609	1
GO:0031639	plasminogen activation	0.105306242	0.084500609	1
GO:0032098	regulation of appetite	0.105306242	0.084500609	1
GO:0034643	establishment of mitochondrion localization, microtubule-mediated	0.105306242	0.084500609	1
GO:0035743	CD4-positive, alpha-beta T cell cytokine production	0.105306242	0.084500609	1
GO:0045723	positive regulation of fatty acid biosynthetic process	0.105306242	0.084500609	1
GO:0047497	mitochondrion transport along microtubule	0.105306242	0.084500609	1
GO:0051151	negative regulation of smooth muscle cell differentiation	0.105306242	0.084500609	1

GO:0060445	branching involved in salivary gland morphogenesis	0.105306242	0.084500609	1
GO:0060547	negative regulation of necrotic cell death	0.105306242	0.084500609	1
GO:0070584	mitochondrion morphogenesis	0.105306242	0.084500609	1
GO:0070935	3'-UTR-mediated mRNA stabilization	0.105306242	0.084500609	1
GO:0072677	eosinophil migration	0.105306242	0.084500609	1
GO:0090343	positive regulation of cell aging	0.105306242	0.084500609	1
GO:0106030	neuron projection fasciculation	0.105306242	0.084500609	1
GO:1901984	negative regulation of protein acetylation	0.105306242	0.084500609	1
GO:1902001	fatty acid transmembrane transport	0.105306242	0.084500609	1
GO:1902254	negative regulation of intrinsic apoptotic signaling pathway by p53 class mediator	0.105306242	0.084500609	1
GO:1903589	positive regulation of blood vessel endothelial cell proliferation involved in sprouting angiogenesis	0.105306242	0.084500609	1
GO:2000810	regulation of bicellular tight junction assembly	0.105306242	0.084500609	1
GO:0006096	glycolytic process	0.106437631	0.085370608	1
GO:0032872	regulation of stress-activated MAPK cascade	0.106855021	0.085667412	1
GO:0030111	regulation of Wnt signaling pathway	0.107710589	0.086315092	1
GO:0002220	innate immune response activating cell surface receptor signaling pathway	0.107978626	0.086415074	1
GO:0006757	ATP generation from ADP	0.107978626	0.086415074	1
GO:0022904	respiratory electron transport chain	0.107978626	0.086415074	1
GO:0006402	mRNA catabolic process	0.108488044	0.086784377	1
GO:0002758	innate immune response-activating signal transduction	0.109525644	0.086850822	1
GO:0014902	myotube differentiation	0.109525644	0.086850822	1
GO:0043666	regulation of phosphoprotein phosphatase activity	0.109525644	0.086850822	1

GO:0001759	organ induction	0.109627058	0.086850822	1
GO:0002438	acute inflammatory response to antigenic stimulus	0.109627058	0.086850822	1
GO:0002719	negative regulation of cytokine production involved in immune response	0.109627058	0.086850822	1
GO:0007095	mitotic G2 DNA damage checkpoint	0.109627058	0.086850822	1
GO:0010869	regulation of receptor biosynthetic process	0.109627058	0.086850822	1
GO:0014850	response to muscle activity	0.109627058	0.086850822	1
GO:0032891	negative regulation of organic acid transport	0.109627058	0.086850822	1
GO:0033622	integrin activation	0.109627058	0.086850822	1
GO:0034695	response to prostaglandin E	0.109627058	0.086850822	1
GO:0044247	cellular polysaccharide catabolic process	0.109627058	0.086850822	1
GO:0044321	response to leptin	0.109627058	0.086850822	1
GO:0044346	fibroblast apoptotic process	0.109627058	0.086850822	1
GO:0044550	secondary metabolite biosynthetic process	0.109627058	0.086850822	1
GO:0045821	positive regulation of glycolytic process	0.109627058	0.086850822	1
GO:0046628	positive regulation of insulin receptor signaling pathway	0.109627058	0.086850822	1
GO:0051349	positive regulation of lyase activity	0.109627058	0.086850822	1
GO:0098743	cell aggregation	0.109627058	0.086850822	1
GO:1903306	negative regulation of regulated secretory pathway	0.109627058	0.086850822	1
GO:1903429	regulation of cell maturation	0.109627058	0.086850822	1
GO:0032886	regulation of microtubule-based process	0.109880722	0.086937593	1
GO:0050803	regulation of synapse structure or activity	0.109880722	0.086937593	1
GO:0070302	regulation of stress-activated protein kinase signaling cascade	0.109880722	0.086937593	1

GO:0051209	release of sequestered calcium ion into cytosol	0.11107859	0.087846934	1
GO:0045666	positive regulation of neuron differentiation	0.111623435	0.088239261	1
GO:1903320	regulation of protein modification by small protein conjugation or removal	0.111916311	0.088432148	1
GO:0051153	regulation of striated muscle cell differentiation	0.112637375	0.088924243	1
GO:0051283	negative regulation of sequestering of calcium ion	0.112637375	0.088924243	1
GO:0000423	mitophagy	0.113927235	0.089280353	1
GO:0002053	positive regulation of mesenchymal cell proliferation	0.113927235	0.089280353	1
GO:0002092	positive regulation of receptor internalization	0.113927235	0.089280353	1
GO:0003309	type B pancreatic cell differentiation	0.113927235	0.089280353	1
GO:0007530	sex determination	0.113927235	0.089280353	1
GO:0014829	vascular associated smooth muscle contraction	0.113927235	0.089280353	1
GO:0022410	circadian sleep/wake cycle process	0.113927235	0.089280353	1
GO:0032104	regulation of response to extracellular stimulus	0.113927235	0.089280353	1
GO:0032107	regulation of response to nutrient levels	0.113927235	0.089280353	1
GO:0045662	negative regulation of myoblast differentiation	0.113927235	0.089280353	1
GO:0046697	decidualization	0.113927235	0.089280353	1
GO:0050857	positive regulation of antigen receptor-mediated signaling pathway	0.113927235	0.089280353	1
GO:0061217	regulation of mesonephros development	0.113927235	0.089280353	1
GO:0061436	establishment of skin barrier	0.113927235	0.089280353	1
GO:0071677	positive regulation of mononuclear cell migration	0.113927235	0.089280353	1
GO:1905564	positive regulation of vascular endothelial cell proliferation	0.113927235	0.089280353	1
GO:2000311	regulation of AMPA receptor activity	0.113927235	0.089280353	1

GO:0051588	regulation of neurotransmitter transport	0.114201906	0.089456859	1
GO:0007613	memory	0.115772095	0.0905692	1
GO:0016079	synaptic vesicle exocytosis	0.115772095	0.0905692	1
GO:0051282	regulation of sequestering of calcium ion	0.115772095	0.0905692	1
GO:0043244	regulation of protein-containing complex disassembly	0.117347851	0.091328874	1
GO:1990266	neutrophil migration	0.117347851	0.091328874	1
GO:0043543	protein acylation	0.118109692	0.091328874	1
GO:0000272	polysaccharide catabolic process	0.118206871	0.091328874	1
GO:0001783	B cell apoptotic process	0.118206871	0.091328874	1
GO:0001945	lymph vessel development	0.118206871	0.091328874	1
GO:0002227	innate immune response in mucosa	0.118206871	0.091328874	1
GO:0030318	melanocyte differentiation	0.118206871	0.091328874	1
GO:0030810	positive regulation of nucleotide biosynthetic process	0.118206871	0.091328874	1
GO:0031664	regulation of lipopolysaccharide-mediated signaling pathway	0.118206871	0.091328874	1
GO:0033233	regulation of protein sumoylation	0.118206871	0.091328874	1
GO:0034114	regulation of heterotypic cell-cell adhesion	0.118206871	0.091328874	1
GO:0042104	positive regulation of activated T cell proliferation	0.118206871	0.091328874	1
GO:0042730	fibrinolysis	0.118206871	0.091328874	1
GO:0043567	regulation of insulin-like growth factor receptor signaling pathway	0.118206871	0.091328874	1
GO:0044068	modulation by symbiont of host cellular process	0.118206871	0.091328874	1
GO:0045026	plasma membrane fusion	0.118206871	0.091328874	1
GO:0048643	positive regulation of skeletal muscle tissue development	0.118206871	0.091328874	1

GO:0051156	glucose 6-phosphate metabolic process	0.118206871	0.091328874	1
GO:0051654	establishment of mitochondrion localization	0.118206871	0.091328874	1
GO:0055083	monovalent inorganic anion homeostasis	0.118206871	0.091328874	1
GO:0060343	trabecula formation	0.118206871	0.091328874	1
GO:0060561	apoptotic process involved in morphogenesis	0.118206871	0.091328874	1
GO:0060706	cell differentiation involved in embryonic placenta development	0.118206871	0.091328874	1
GO:0090023	positive regulation of neutrophil chemotaxis	0.118206871	0.091328874	1
GO:0097066	response to thyroid hormone	0.118206871	0.091328874	1
GO:1900373	positive regulation of purine nucleotide biosynthetic process	0.118206871	0.091328874	1
GO:1903649	regulation of cytoplasmic transport	0.118206871	0.091328874	1
GO:1905523	positive regulation of macrophage migration	0.118206871	0.091328874	1
GO:0031929	TOR signaling	0.120515713	0.092953969	1
GO:0046031	ADP metabolic process	0.120515713	0.092953969	1
GO:0051101	regulation of DNA binding	0.120515713	0.092953969	1
GO:0051208	sequestering of calcium ion	0.120515713	0.092953969	1
GO:0002833	positive regulation of response to biotic stimulus	0.121253985	0.093483551	1
GO:0001838	embryonic epithelial tube formation	0.122107643	0.093659813	1
GO:0003014	renal system process	0.122107643	0.093659813	1
GO:0006706	steroid catabolic process	0.122466063	0.093659813	1
GO:0008053	mitochondrial fusion	0.122466063	0.093659813	1
GO:0009065	glutamine family amino acid catabolic process	0.122466063	0.093659813	1
GO:0018126	protein hydroxylation	0.122466063	0.093659813	1

GO:0031954	positive regulation of protein autophosphorylation	0.122466063	0.093659813	1
GO:0032897	negative regulation of viral transcription	0.122466063	0.093659813	1
GO:0036037	CD8-positive, alpha-beta T cell activation	0.122466063	0.093659813	1
GO:0050996	positive regulation of lipid catabolic process	0.122466063	0.093659813	1
GO:0051953	negative regulation of amine transport	0.122466063	0.093659813	1
GO:0060544	regulation of necroptotic process	0.122466063	0.093659813	1
GO:0062098	regulation of programmed necrotic cell death	0.122466063	0.093659813	1
GO:0071514	genetic imprinting	0.122466063	0.093659813	1
GO:0072539	T-helper 17 cell differentiation	0.122466063	0.093659813	1
GO:0090140	regulation of mitochondrial fission	0.122466063	0.093659813	1
GO:1900078	positive regulation of cellular response to insulin stimulus	0.122466063	0.093659813	1
GO:1900120	regulation of receptor binding	0.122466063	0.093659813	1
GO:1901623	regulation of lymphocyte chemotaxis	0.122466063	0.093659813	1
GO:0030217	T cell differentiation	0.123367393	0.094309274	1
GO:0001704	formation of primary germ layer	0.125307069	0.095489044	1
GO:0014066	regulation of phosphatidylinositol 3-kinase signaling	0.125307069	0.095489044	1
GO:0051053	negative regulation of DNA metabolic process	0.125307069	0.095489044	1
GO:1903052	positive regulation of proteolysis involved in cellular protein catabolic process	0.125307069	0.095489044	1
GO:0000470	maturation of LSU-rRNA	0.126704907	0.095489044	1
GO:0001964	startle response	0.126704907	0.095489044	1
GO:0002335	mature B cell differentiation	0.126704907	0.095489044	1
GO:0003180	aortic valve morphogenesis	0.126704907	0.095489044	1

GO:0006929	substrate-dependent cell migration	0.126704907	0.095489044	1
GO:0007205	protein kinase C-activating G protein-coupled receptor signaling pathway	0.126704907	0.095489044	1
GO:0022011	myelination in peripheral nervous system	0.126704907	0.095489044	1
GO:0030194	positive regulation of blood coagulation	0.126704907	0.095489044	1
GO:0031069	hair follicle morphogenesis	0.126704907	0.095489044	1
GO:0031440	regulation of mRNA 3'-end processing	0.126704907	0.095489044	1
GO:0032292	peripheral nervous system axon ensheathment	0.126704907	0.095489044	1
GO:0033561	regulation of water loss via skin	0.126704907	0.095489044	1
GO:0035883	enteroendocrine cell differentiation	0.126704907	0.095489044	1
GO:0042745	circadian sleep/wake cycle	0.126704907	0.095489044	1
GO:0044331	cell-cell adhesion mediated by cadherin	0.126704907	0.095489044	1
GO:0045745	positive regulation of G protein-coupled receptor signaling pathway	0.126704907	0.095489044	1
GO:0048753	pigment granule organization	0.126704907	0.095489044	1
GO:0060143	positive regulation of syncytium formation by plasma membrane fusion	0.126704907	0.095489044	1
GO:0071168	protein localization to chromatin	0.126704907	0.095489044	1
GO:0071280	cellular response to copper ion	0.126704907	0.095489044	1
GO:0086013	membrane repolarization during cardiac muscle cell action potential	0.126704907	0.095489044	1
GO:0099560	synaptic membrane adhesion	0.126704907	0.095489044	1
GO:1900048	positive regulation of hemostasis	0.126704907	0.095489044	1
GO:1900101	regulation of endoplasmic reticulum unfolded protein response	0.126704907	0.095489044	1
GO:1902932	positive regulation of alcohol biosynthetic process	0.126704907	0.095489044	1
GO:1903579	negative regulation of ATP metabolic process	0.126704907	0.095489044	1

GO:1905063	regulation of vascular associated smooth muscle cell differentiation	0.126704907	0.095489044	1
GO:1905563	negative regulation of vascular endothelial cell proliferation	0.126704907	0.095489044	1
GO:2000191	regulation of fatty acid transport	0.126704907	0.095489044	1
GO:2000463	positive regulation of excitatory postsynaptic potential	0.126704907	0.095489044	1
GO:0051607	defense response to virus	0.128709834	0.096938675	1
GO:0009150	purine ribonucleotide metabolic process	0.128735576	0.096938675	1
GO:0042180	cellular ketone metabolic process	0.130869897	0.097934117	1
GO:0008209	androgen metabolic process	0.1309235	0.097934117	1
GO:0009435	NAD biosynthetic process	0.1309235	0.097934117	1
GO:0032373	positive regulation of sterol transport	0.1309235	0.097934117	1
GO:0032376	positive regulation of cholesterol transport	0.1309235	0.097934117	1
GO:0033032	regulation of myeloid cell apoptotic process	0.1309235	0.097934117	1
GO:0036296	response to increased oxygen levels	0.1309235	0.097934117	1
GO:0042634	regulation of hair cycle	0.1309235	0.097934117	1
GO:0043153	entrainment of circadian clock by photoperiod	0.1309235	0.097934117	1
GO:0045822	negative regulation of heart contraction	0.1309235	0.097934117	1
GO:0050820	positive regulation of coagulation	0.1309235	0.097934117	1
GO:0050901	leukocyte tethering or rolling	0.1309235	0.097934117	1
GO:0055075	potassium ion homeostasis	0.1309235	0.097934117	1
GO:0060441	epithelial tube branching involved in lung morphogenesis	0.1309235	0.097934117	1
GO:0071624	positive regulation of granulocyte chemotaxis	0.1309235	0.097934117	1
GO:0080154	regulation of fertilization	0.1309235	0.097934117	1

GO:0019218	regulation of steroid metabolic process	0.131765803	0.098442094	1
GO:0032434	regulation of proteasomal ubiquitin-dependent protein catabolic process	0.131765803	0.098442094	1
GO:0045667	regulation of osteoblast differentiation	0.131765803	0.098442094	1
GO:0043547	positive regulation of GTPase activity	0.132965282	0.099297226	1
GO:0061448	connective tissue development	0.133042879	0.099314186	1
GO:0042476	odontogenesis	0.133392474	0.099534091	1
GO:0006165	nucleoside diphosphate phosphorylation	0.135023776	0.100081699	1
GO:0006997	nucleus organization	0.135023776	0.100081699	1
GO:0042177	negative regulation of protein catabolic process	0.135023776	0.100081699	1
GO:0072175	epithelial tube formation	0.135023776	0.100081699	1
GO:0003401	axis elongation	0.135121938	0.100081699	1
GO:0007176	regulation of epidermal growth factor-activated receptor activity	0.135121938	0.100081699	1
GO:0010586	miRNA metabolic process	0.135121938	0.100081699	1
GO:0010758	regulation of macrophage chemotaxis	0.135121938	0.100081699	1
GO:0019098	reproductive behavior	0.135121938	0.100081699	1
GO:0033137	negative regulation of peptidyl-serine phosphorylation	0.135121938	0.100081699	1
GO:0033688	regulation of osteoblast proliferation	0.135121938	0.100081699	1
GO:0035666	TRIF-dependent toll-like receptor signaling pathway	0.135121938	0.100081699	1
GO:0048265	response to pain	0.135121938	0.100081699	1
GO:0060142	regulation of syncytium formation by plasma membrane fusion	0.135121938	0.100081699	1
GO:0070168	negative regulation of biomineral tissue development	0.135121938	0.100081699	1
GO:0070723	response to cholesterol	0.135121938	0.100081699	1

GO:0110150	negative regulation of biomineralization	0.135121938	0.100081699	1
GO:0120033	negative regulation of plasma membrane bounded cell projection assembly	0.135121938	0.100081699	1
GO:0071333	cellular response to glucose stimulus	0.136659627	0.101179214	1
GO:0009165	nucleotide biosynthetic process	0.137426859	0.101705638	1
GO:0046939	nucleotide phosphorylation	0.138299947	0.10226813	1
GO:0050853	B cell receptor signaling pathway	0.138299947	0.10226813	1
GO:0002828	regulation of type 2 immune response	0.139300314	0.102338714	1
GO:0009154	purine ribonucleotide catabolic process	0.139300314	0.102338714	1
GO:0014044	Schwann cell development	0.139300314	0.102338714	1
GO:0034368	protein-lipid complex remodeling	0.139300314	0.102338714	1
GO:0034369	plasma lipoprotein particle remodeling	0.139300314	0.102338714	1
GO:0043171	peptide catabolic process	0.139300314	0.102338714	1
GO:0045940	positive regulation of steroid metabolic process	0.139300314	0.102338714	1
GO:0048710	regulation of astrocyte differentiation	0.139300314	0.102338714	1
GO:0070229	negative regulation of lymphocyte apoptotic process	0.139300314	0.102338714	1
GO:0071539	protein localization to centrosome	0.139300314	0.102338714	1
GO:0071549	cellular response to dexamethasone stimulus	0.139300314	0.102338714	1
GO:0072538	T-helper 17 type immune response	0.139300314	0.102338714	1
GO:1900745	positive regulation of p38MAPK cascade	0.139300314	0.102338714	1
GO:1902230	negative regulation of intrinsic apoptotic signaling pathway in response to DNA damage	0.139300314	0.102338714	1
GO:1902624	positive regulation of neutrophil migration	0.139300314	0.102338714	1
GO:1903792	negative regulation of anion transport	0.139300314	0.102338714	1

GO:00091<br>35	purine nucleoside diphosphate metabolic process	0.1399446<br>56	0.1026870<br>12	1
GO:00091<br>79	purine ribonucleoside diphosphate metabolic process	0.1399446<br>56	0.1026870<br>12	1
GO:00713<br>31	cellular response to hexose stimulus	0.1399446<br>56	0.1026870<br>12	1
GO:00064<br>01	RNA catabolic process	0.1407224<br>09	0.1031923<br>38	1
GO:19012<br>93	nucleoside phosphate biosynthetic process	0.1407473<br>86	0.1031923<br>38	1
GO:00713<br>26	cellular response to monosaccharide stimulus	0.1415936<br>75	0.1036880<br>91	1
GO:19030<br>38	negative regulation of leukocyte cell-cell adhesion	0.1415936<br>75	0.1036880<br>91	1
GO:00092<br>59	ribonucleotide metabolic process	0.1415954<br>68	0.1036880<br>91	1
GO:00020<br>82	regulation of oxidative phosphorylation	0.1434587<br>25	0.1040837<br>16	1
GO:00027<br>24	regulation of T cell cytokine production	0.1434587<br>25	0.1040837<br>16	1
GO:00031<br>76	aortic valve development	0.1434587<br>25	0.1040837<br>16	1
GO:00092<br>61	ribonucleotide catabolic process	0.1434587<br>25	0.1040837<br>16	1
GO:00193<br>59	nicotinamide nucleotide biosynthetic process	0.1434587<br>25	0.1040837<br>16	1
GO:00193<br>63	pyridine nucleotide biosynthetic process	0.1434587<br>25	0.1040837<br>16	1
GO:00323<br>50	regulation of hormone metabolic process	0.1434587<br>25	0.1040837<br>16	1
GO:00343<br>67	protein-containing complex remodeling	0.1434587<br>25	0.1040837<br>16	1
GO:00346<br>94	response to prostaglandin	0.1434587<br>25	0.1040837<br>16	1
GO:00381<br>28	ERBB2 signaling pathway	0.1434587<br>25	0.1040837<br>16	1
GO:00427<br>44	hydrogen peroxide catabolic process	0.1434587<br>25	0.1040837<br>16	1
GO:00448<br>18	mitotic G2/M transition checkpoint	0.1434587<br>25	0.1040837<br>16	1
GO:00463<br>20	regulation of fatty acid oxidation	0.1434587<br>25	0.1040837<br>16	1
GO:00469<br>49	fatty-acyl-CoA biosynthetic process	0.1434587<br>25	0.1040837<br>16	1

GO:0048730	epidermis morphogenesis	0.143458725	0.104083716	1
GO:0048741	skeletal muscle fiber development	0.143458725	0.104083716	1
GO:0048841	regulation of axon extension involved in axon guidance	0.143458725	0.104083716	1
GO:0050849	negative regulation of calcium-mediated signaling	0.143458725	0.104083716	1
GO:0050869	negative regulation of B cell activation	0.143458725	0.104083716	1
GO:0061036	positive regulation of cartilage development	0.143458725	0.104083716	1
GO:0071353	cellular response to interleukin-4	0.143458725	0.104083716	1
GO:1901976	regulation of cell cycle checkpoint	0.143458725	0.104083716	1
GO:1903959	regulation of anion transmembrane transport	0.143458725	0.104083716	1
GO:0009185	ribonucleoside diphosphate metabolic process	0.144904329	0.105090409	1
GO:0006260	DNA replication	0.145216963	0.105274949	1
GO:0007266	Rho protein signal transduction	0.146565809	0.106149987	1
GO:0002861	regulation of inflammatory response to antigenic stimulus	0.147597265	0.106149987	1
GO:0009303	rRNA transcription	0.147597265	0.106149987	1
GO:0009648	photoperiodism	0.147597265	0.106149987	1
GO:0010464	regulation of mesenchymal cell proliferation	0.147597265	0.106149987	1
GO:0030149	sphingolipid catabolic process	0.147597265	0.106149987	1
GO:0040020	regulation of meiotic nuclear division	0.147597265	0.106149987	1
GO:0042573	retinoic acid metabolic process	0.147597265	0.106149987	1
GO:0042755	eating behavior	0.147597265	0.106149987	1
GO:0045920	negative regulation of exocytosis	0.147597265	0.106149987	1
GO:0048011	neurotrophin TRK receptor signaling pathway	0.147597265	0.106149987	1

GO:0048384	retinoic acid receptor signaling pathway	0.147597265	0.106149987	1
GO:0050685	positive regulation of mRNA processing	0.147597265	0.106149987	1
GO:0051984	positive regulation of chromosome segregation	0.147597265	0.106149987	1
GO:0090022	regulation of neutrophil chemotaxis	0.147597265	0.106149987	1
GO:0120255	olefinic compound biosynthetic process	0.147597265	0.106149987	1
GO:1901380	negative regulation of potassium ion transmembrane transport	0.147597265	0.106149987	1
GO:1901797	negative regulation of signal transduction by p53 class mediator	0.147597265	0.106149987	1
GO:1905048	regulation of metallopeptidase activity	0.147597265	0.106149987	1
GO:1905508	protein localization to microtubule organizing center	0.147597265	0.106149987	1
GO:0030010	establishment of cell polarity	0.148231289	0.106563614	1
GO:0046425	regulation of receptor signaling pathway via JAK-STAT	0.149900692	0.107678189	1
GO:0072073	kidney epithelium development	0.149900692	0.107678189	1
GO:0019693	ribose phosphate metabolic process	0.151341789	0.107826457	1
GO:0072006	nephron development	0.151573944	0.107826457	1
GO:0001881	receptor recycling	0.151716027	0.107826457	1
GO:0002756	MyD88-independent toll-like receptor signaling pathway	0.151716027	0.107826457	1
GO:0007094	mitotic spindle assembly checkpoint	0.151716027	0.107826457	1
GO:0007202	activation of phospholipase C activity	0.151716027	0.107826457	1
GO:0008156	negative regulation of DNA replication	0.151716027	0.107826457	1
GO:0008608	attachment of spindle microtubules to kinetochore	0.151716027	0.107826457	1
GO:0009649	entrainment of circadian clock	0.151716027	0.107826457	1
GO:0010762	regulation of fibroblast migration	0.151716027	0.107826457	1

GO:0018149	peptide cross-linking	0.151716027	0.107826457	1
GO:0019228	neuronal action potential	0.151716027	0.107826457	1
GO:0030851	granulocyte differentiation	0.151716027	0.107826457	1
GO:0031577	spindle checkpoint	0.151716027	0.107826457	1
GO:0033198	response to ATP	0.151716027	0.107826457	1
GO:0035774	positive regulation of insulin secretion involved in cellular response to glucose stimulus	0.151716027	0.107826457	1
GO:0043516	regulation of DNA damage response, signal transduction by p53 class mediator	0.151716027	0.107826457	1
GO:0046685	response to arsenic-containing substance	0.151716027	0.107826457	1
GO:0048536	spleen development	0.151716027	0.107826457	1
GO:0048854	brain morphogenesis	0.151716027	0.107826457	1
GO:0050715	positive regulation of cytokine secretion	0.151716027	0.107826457	1
GO:0055094	response to lipoprotein particle	0.151716027	0.107826457	1
GO:0061384	heart trabecula morphogenesis	0.151716027	0.107826457	1
GO:0070102	interleukin-6-mediated signaling pathway	0.151716027	0.107826457	1
GO:0071173	spindle assembly checkpoint	0.151716027	0.107826457	1
GO:0071174	mitotic spindle checkpoint	0.151716027	0.107826457	1
GO:2000352	negative regulation of endothelial cell apoptotic process	0.151716027	0.107826457	1
GO:0002218	activation of innate immune response	0.15325097	0.108831867	1
GO:0002700	regulation of production of molecular mediator of immune response	0.15325097	0.108831867	1
GO:0072659	protein localization to plasma membrane	0.154295308	0.109530522	1
GO:0071322	cellular response to carbohydrate stimulus	0.154931695	0.109606492	1
GO:0016054	organic acid catabolic process	0.155442702	0.109606492	1

GO:0046395	carboxylic acid catabolic process	0.155442702	0.109606492	1
GO:0006739	NADP metabolic process	0.155815105	0.109606492	1
GO:0007435	salivary gland morphogenesis	0.155815105	0.109606492	1
GO:0010259	multicellular organism aging	0.155815105	0.109606492	1
GO:0014904	myotube cell development	0.155815105	0.109606492	1
GO:0019362	pyridine nucleotide metabolic process	0.155815105	0.109606492	1
GO:0030947	regulation of vascular endothelial growth factor receptor signaling pathway	0.155815105	0.109606492	1
GO:0032228	regulation of synaptic transmission, GABAergic	0.155815105	0.109606492	1
GO:0032735	positive regulation of interleukin-12 production	0.155815105	0.109606492	1
GO:0033687	osteoblast proliferation	0.155815105	0.109606492	1
GO:0036314	response to sterol	0.155815105	0.109606492	1
GO:0039694	viral RNA genome replication	0.155815105	0.109606492	1
GO:0042462	eye photoreceptor cell development	0.155815105	0.109606492	1
GO:0045922	negative regulation of fatty acid metabolic process	0.155815105	0.109606492	1
GO:0046496	nicotinamide nucleotide metabolic process	0.155815105	0.109606492	1
GO:0070306	lens fiber cell differentiation	0.155815105	0.109606492	1
GO:0070670	response to interleukin-4	0.155815105	0.109606492	1
GO:0072525	pyridine-containing compound biosynthetic process	0.155815105	0.109606492	1
GO:1900016	negative regulation of cytokine production involved in inflammatory response	0.155815105	0.109606492	1
GO:1901020	negative regulation of calcium ion transmembrane transporter activity	0.155815105	0.109606492	1
GO:1901223	negative regulation of NIK/NF-kappaB signaling	0.155815105	0.109606492	1
GO:0051656	establishment of organelle localization	0.155856095	0.109606492	1

GO:0044344	cellular response to fibroblast growth factor stimulus	0.156616046	0.110012661	1
GO:0046631	alpha-beta T cell activation	0.156616046	0.110012661	1
GO:0048813	dendrite morphogenesis	0.156616046	0.110012661	1
GO:0050808	synapse organization	0.156765094	0.110074626	1
GO:0007043	cell-cell junction assembly	0.15830395	0.111068954	1
GO:0046467	membrane lipid biosynthetic process	0.15830395	0.111068954	1
GO:0000460	maturation of 5.8S rRNA	0.159894593	0.111665402	1
GO:0002701	negative regulation of production of molecular mediator of immune response	0.159894593	0.111665402	1
GO:0003382	epithelial cell morphogenesis	0.159894593	0.111665402	1
GO:0006007	glucose catabolic process	0.159894593	0.111665402	1
GO:0010092	specification of animal organ identity	0.159894593	0.111665402	1
GO:0031128	developmental induction	0.159894593	0.111665402	1
GO:0033280	response to vitamin D	0.159894593	0.111665402	1
GO:0045841	negative regulation of mitotic metaphase/anaphase transition	0.159894593	0.111665402	1
GO:0046676	negative regulation of insulin secretion	0.159894593	0.111665402	1
GO:0050931	pigment cell differentiation	0.159894593	0.111665402	1
GO:0071402	cellular response to lipoprotein particle stimulus	0.159894593	0.111665402	1
GO:1901385	regulation of voltage-gated calcium channel activity	0.159894593	0.111665402	1
GO:0031644	regulation of nervous system process	0.159995335	0.11169265	1
GO:0006119	oxidative phosphorylation	0.161690129	0.112788756	1
GO:1903364	positive regulation of cellular protein catabolic process	0.161690129	0.112788756	1
GO:0030212	hyaluronan metabolic process	0.163954582	0.114016718	1

GO:0045730	respiratory burst	0.163954582	0.114016718	1
GO:0048846	axon extension involved in axon guidance	0.163954582	0.114016718	1
GO:0097242	amyloid-beta clearance	0.163954582	0.114016718	1
GO:1902100	negative regulation of metaphase/anaphase transition of cell cycle	0.163954582	0.114016718	1
GO:1902284	neuron projection extension involved in neuron projection guidance	0.163954582	0.114016718	1
GO:1903514	release of sequestered calcium ion into cytosol by endoplasmic reticulum	0.163954582	0.114016718	1
GO:1905314	semi-lunar valve development	0.163954582	0.114016718	1
GO:1904892	regulation of receptor signaling pathway via STAT	0.165089661	0.114761966	1
GO:0002224	toll-like receptor signaling pathway	0.166794259	0.115857897	1
GO:0006090	pyruvate metabolic process	0.166794259	0.115857897	1
GO:0002385	mucosal immune response	0.167995164	0.116068281	1
GO:0003156	regulation of animal organ formation	0.167995164	0.116068281	1
GO:0003298	physiological muscle hypertrophy	0.167995164	0.116068281	1
GO:0003301	physiological cardiac muscle hypertrophy	0.167995164	0.116068281	1
GO:0006084	acetyl-CoA metabolic process	0.167995164	0.116068281	1
GO:0007223	Wnt signaling pathway, calcium modulating pathway	0.167995164	0.116068281	1
GO:0010661	positive regulation of muscle cell apoptotic process	0.167995164	0.116068281	1
GO:0032733	positive regulation of interleukin-10 production	0.167995164	0.116068281	1
GO:0045923	positive regulation of fatty acid metabolic process	0.167995164	0.116068281	1
GO:0046466	membrane lipid catabolic process	0.167995164	0.116068281	1
GO:0051930	regulation of sensory perception of pain	0.167995164	0.116068281	1
GO:0061049	cell growth involved in cardiac muscle cell development	0.167995164	0.116068281	1

GO:1903523	negative regulation of blood circulation	0.167995164	0.116068281	1
GO:1905898	positive regulation of response to endoplasmic reticulum stress	0.167995164	0.116068281	1
GO:0001678	cellular glucose homeostasis	0.168501984	0.116285243	1
GO:0002822	regulation of adaptive immune response based on somatic recombination of immune receptors built from immunoglobulin superfamily domains	0.168501984	0.116285243	1
GO:0071774	response to fibroblast growth factor	0.168501984	0.116285243	1
GO:0002706	regulation of lymphocyte mediated immunity	0.17021277	0.117421093	1
GO:0009132	nucleoside diphosphate metabolic process	0.171926546	0.117901215	1
GO:0001662	behavioral fear response	0.172016433	0.117901215	1
GO:0006656	phosphatidylcholine biosynthetic process	0.172016433	0.117901215	1
GO:0006778	porphyrin-containing compound metabolic process	0.172016433	0.117901215	1
GO:0007431	salivary gland development	0.172016433	0.117901215	1
GO:0010453	regulation of cell fate commitment	0.172016433	0.117901215	1
GO:0030866	cortical actin cytoskeleton organization	0.172016433	0.117901215	1
GO:0031111	negative regulation of microtubule polymerization or depolymerization	0.172016433	0.117901215	1
GO:0032660	regulation of interleukin-17 production	0.172016433	0.117901215	1
GO:0038179	neurotrophin signaling pathway	0.172016433	0.117901215	1
GO:0042092	type 2 immune response	0.172016433	0.117901215	1
GO:0046329	negative regulation of JNK cascade	0.172016433	0.117901215	1
GO:0051931	regulation of sensory perception	0.172016433	0.117901215	1
GO:1901021	positive regulation of calcium ion transmembrane transporter activity	0.172016433	0.117901215	1
GO:1902229	regulation of intrinsic apoptotic signaling pathway in response to DNA damage	0.172016433	0.117901215	1
GO:1904706	negative regulation of vascular associated smooth muscle cell proliferation	0.172016433	0.117901215	1

GO:2000816	negative regulation of mitotic sister chromatid separation	0.172016433	0.117901215	1
GO:0000187	activation of MAPK activity	0.173643247	0.118926149	1
GO:1902904	negative regulation of supramolecular fiber organization	0.173643247	0.118926149	1
GO:0044282	small molecule catabolic process	0.174408211	0.119404869	1
GO:0010770	positive regulation of cell morphogenesis involved in differentiation	0.175362804	0.119737144	1
GO:0002209	behavioral defense response	0.176018478	0.119737144	1
GO:0002714	positive regulation of B cell mediated immunity	0.176018478	0.119737144	1
GO:0002891	positive regulation of immunoglobulin mediated immune response	0.176018478	0.119737144	1
GO:0009187	cyclic nucleotide metabolic process	0.176018478	0.119737144	1
GO:0010939	regulation of necrotic cell death	0.176018478	0.119737144	1
GO:0030501	positive regulation of bone mineralization	0.176018478	0.119737144	1
GO:0045740	positive regulation of DNA replication	0.176018478	0.119737144	1
GO:0045746	negative regulation of Notch signaling pathway	0.176018478	0.119737144	1
GO:0050892	intestinal absorption	0.176018478	0.119737144	1
GO:0051281	positive regulation of release of sequestered calcium ion into cytosol	0.176018478	0.119737144	1
GO:0090184	positive regulation of kidney development	0.176018478	0.119737144	1
GO:0097352	autophagosome maturation	0.176018478	0.119737144	1
GO:0140353	lipid export from cell	0.176018478	0.119737144	1
GO:1901381	positive regulation of potassium ion transmembrane transport	0.176018478	0.119737144	1
GO:1902742	apoptotic process involved in development	0.176018478	0.119737144	1
GO:1905819	negative regulation of chromosome separation	0.176018478	0.119737144	1
GO:0016573	histone acetylation	0.178810224	0.121499209	1

GO:0032675	regulation of interleukin-6 production	0.178810224	0.121499209	1
GO:2000058	regulation of ubiquitin-dependent protein catabolic process	0.178810224	0.121499209	1
GO:0006734	NADH metabolic process	0.180001391	0.121805452	1
GO:0033574	response to testosterone	0.180001391	0.121805452	1
GO:0035337	fatty-acyl-CoA metabolic process	0.180001391	0.121805452	1
GO:0043267	negative regulation of potassium ion transport	0.180001391	0.121805452	1
GO:0048286	lung alveolus development	0.180001391	0.121805452	1
GO:0050434	positive regulation of viral transcription	0.180001391	0.121805452	1
GO:0050832	defense response to fungus	0.180001391	0.121805452	1
GO:0070317	negative regulation of G0 to G1 transition	0.180001391	0.121805452	1
GO:0071548	response to dexamethasone	0.180001391	0.121805452	1
GO:0072524	pyridine-containing compound metabolic process	0.180001391	0.121805452	1
GO:1903170	negative regulation of calcium ion transmembrane transport	0.180001391	0.121805452	1
GO:0010970	transport along microtubule	0.18226828	0.12329333	1
GO:0000266	mitochondrial fission	0.183965262	0.123909644	1
GO:0001709	cell fate determination	0.183965262	0.123909644	1
GO:0021983	pituitary gland development	0.183965262	0.123909644	1
GO:0033048	negative regulation of mitotic sister chromatid segregation	0.183965262	0.123909644	1
GO:0044058	regulation of digestive system process	0.183965262	0.123909644	1
GO:0046621	negative regulation of organ growth	0.183965262	0.123909644	1
GO:0072595	maintenance of protein localization in organelle	0.183965262	0.123909644	1
GO:0090278	negative regulation of peptide hormone secretion	0.183965262	0.123909644	1

GO:00902 79	regulation of calcium ion import	0.1839652 62	0.1239096 44	1
GO:19003 71	regulation of purine nucleotide biosynthetic process	0.1839652 62	0.1239096 44	1
GO:19026 22	regulation of neutrophil migration	0.1839652 62	0.1239096 44	1
GO:00073 38	single fertilization	0.1840011 36	0.1239096 44	1
GO:00219 15	neural tube development	0.1857364 58	0.1249852 11	1
GO:00514 94	negative regulation of cytoskeleton organization	0.1857364 58	0.1249852 11	1
GO:00017 64	neuron migration	0.1874741 82	0.1256071 4	1
GO:00183 93	internal peptidyl-lysine acetylation	0.1874741 82	0.1256071 4	1
GO:00461 65	alcohol biosynthetic process	0.1874741 82	0.1256071 4	1
GO:00019 53	negative regulation of cell-matrix adhesion	0.1879101 81	0.1256071 4	1
GO:00023 69	T cell cytokine production	0.1879101 81	0.1256071 4	1
GO:00171 58	regulation of calcium ion-dependent exocytosis	0.1879101 81	0.1256071 4	1
GO:00217 62	substantia nigra development	0.1879101 81	0.1256071 4	1
GO:00305 17	negative regulation of axon extension	0.1879101 81	0.1256071 4	1
GO:00305 21	androgen receptor signaling pathway	0.1879101 81	0.1256071 4	1
GO:00308 08	regulation of nucleotide biosynthetic process	0.1879101 81	0.1256071 4	1
GO:00400 19	positive regulation of embryonic development	0.1879101 81	0.1256071 4	1
GO:00466 88	response to copper ion	0.1879101 81	0.1256071 4	1
GO:00467 17	acid secretion	0.1879101 81	0.1256071 4	1
GO:00508 50	positive regulation of calcium-mediated signaling	0.1879101 81	0.1256071 4	1
GO:00508 56	regulation of T cell receptor signaling pathway	0.1879101 81	0.1256071 4	1
GO:00713 64	cellular response to epidermal growth factor stimulus	0.1879101 81	0.1256071 4	1

GO:0086004	regulation of cardiac muscle cell contraction	0.187910181	0.12560714	1
GO:1901031	regulation of response to reactive oxygen species	0.187910181	0.12560714	1
GO:0006475	internal protein amino acid acetylation	0.190956592	0.127478011	1
GO:0006195	purine nucleotide catabolic process	0.191836238	0.127478011	1
GO:0010463	mesenchymal cell proliferation	0.191836238	0.127478011	1
GO:0031670	cellular response to nutrient	0.191836238	0.127478011	1
GO:0032620	interleukin-17 production	0.191836238	0.127478011	1
GO:0032965	regulation of collagen biosynthetic process	0.191836238	0.127478011	1
GO:0033046	negative regulation of sister chromatid segregation	0.191836238	0.127478011	1
GO:0035307	positive regulation of protein dephosphorylation	0.191836238	0.127478011	1
GO:0045332	phospholipid translocation	0.191836238	0.127478011	1
GO:0045684	positive regulation of epidermis development	0.191836238	0.127478011	1
GO:0045687	positive regulation of glial cell differentiation	0.191836238	0.127478011	1
GO:0048489	synaptic vesicle transport	0.191836238	0.127478011	1
GO:1902667	regulation of axon guidance	0.191836238	0.127478011	1
GO:1903053	regulation of extracellular matrix organization	0.191836238	0.127478011	1
GO:1905521	regulation of macrophage migration	0.191836238	0.127478011	1
GO:2000404	regulation of T cell migration	0.191836238	0.127478011	1
GO:0090305	nucleic acid phosphodiester bond hydrolysis	0.192249847	0.127705961	1
GO:0002819	regulation of adaptive immune response	0.19444787	0.129118644	1
GO:0001974	blood vessel remodeling	0.195743523	0.129409117	1
GO:0002639	positive regulation of immunoglobulin production	0.195743523	0.129409117	1

GO:0006775	fat-soluble vitamin metabolic process	0.195743523	0.129409117	1
GO:0007520	myoblast fusion	0.195743523	0.129409117	1
GO:0010677	negative regulation of cellular carbohydrate metabolic process	0.195743523	0.129409117	1
GO:0034198	cellular response to amino acid starvation	0.195743523	0.129409117	1
GO:0046189	phenol-containing compound biosynthetic process	0.195743523	0.129409117	1
GO:0051985	negative regulation of chromosome segregation	0.195743523	0.129409117	1
GO:0052372	modulation by symbiont of entry into host	0.195743523	0.129409117	1
GO:0060412	ventricular septum morphogenesis	0.195743523	0.129409117	1
GO:0070316	regulation of G0 to G1 transition	0.195743523	0.129409117	1
GO:0098815	modulation of excitatory postsynaptic potential	0.195743523	0.129409117	1
GO:0032874	positive regulation of stress-activated MAPK cascade	0.19794753	0.130818425	1
GO:0001754	eye photoreceptor cell differentiation	0.199632122	0.131400922	1
GO:0010761	fibroblast migration	0.199632122	0.131400922	1
GO:0010874	regulation of cholesterol efflux	0.199632122	0.131400922	1
GO:0031641	regulation of myelination	0.199632122	0.131400922	1
GO:0032007	negative regulation of TOR signaling	0.199632122	0.131400922	1
GO:0032309	icosanoid secretion	0.199632122	0.131400922	1
GO:0045023	G0 to G1 transition	0.199632122	0.131400922	1
GO:0048538	thymus development	0.199632122	0.131400922	1
GO:0051489	regulation of filopodium assembly	0.199632122	0.131400922	1
GO:0051646	mitochondrion localization	0.199632122	0.131400922	1
GO:1900744	regulation of p38MAPK cascade	0.199632122	0.131400922	1

GO:0051302	regulation of cell division	0.199700354	0.131400922	1
GO:0018394	peptidyl-lysine acetylation	0.201455095	0.132459191	1
GO:0070304	positive regulation of stress-activated protein kinase signaling cascade	0.201455095	0.132459191	1
GO:0002429	immune response-activating cell surface receptor signaling pathway	0.202578942	0.132983656	1
GO:0002757	immune response-activating signal transduction	0.202578942	0.132983656	1
GO:0043087	regulation of GTPase activity	0.202578942	0.132983656	1
GO:0006383	transcription by RNA polymerase III	0.203502127	0.132983656	1
GO:0007080	mitotic metaphase plate congression	0.203502127	0.132983656	1
GO:0008542	visual learning	0.203502127	0.132983656	1
GO:0010559	regulation of glycoprotein biosynthetic process	0.203502127	0.132983656	1
GO:0010569	regulation of double-strand break repair via homologous recombination	0.203502127	0.132983656	1
GO:0030261	chromosome condensation	0.203502127	0.132983656	1
GO:0032369	negative regulation of lipid transport	0.203502127	0.132983656	1
GO:0034204	lipid translocation	0.203502127	0.132983656	1
GO:0035850	epithelial cell differentiation involved in kidney development	0.203502127	0.132983656	1
GO:0042551	neuron maturation	0.203502127	0.132983656	1
GO:0048483	autonomic nervous system development	0.203502127	0.132983656	1
GO:0051150	regulation of smooth muscle cell differentiation	0.203502127	0.132983656	1
GO:0070849	response to epidermal growth factor	0.203502127	0.132983656	1
GO:1903115	regulation of actin filament-based movement	0.203502127	0.132983656	1
GO:0007269	neurotransmitter secretion	0.206730239	0.134625376	1
GO:0010921	regulation of phosphatase activity	0.206730239	0.134625376	1

GO:0099643	signal release from synapse	0.206730239	0.134625376	1
GO:0050851	antigen receptor-mediated signaling pathway	0.207056569	0.134625376	1
GO:0008089	anterograde axonal transport	0.207353623	0.134625376	1
GO:0014911	positive regulation of smooth muscle cell migration	0.207353623	0.134625376	1
GO:0033628	regulation of cell adhesion mediated by integrin	0.207353623	0.134625376	1
GO:0042461	photoreceptor cell development	0.207353623	0.134625376	1
GO:0051932	synaptic transmission, GABAergic	0.207353623	0.134625376	1
GO:0051972	regulation of telomerase activity	0.207353623	0.134625376	1
GO:0055010	ventricular cardiac muscle tissue morphogenesis	0.207353623	0.134625376	1
GO:0060632	regulation of microtubule-based movement	0.207353623	0.134625376	1
GO:0070169	positive regulation of biomineral tissue development	0.207353623	0.134625376	1
GO:0110151	positive regulation of biomineralization	0.207353623	0.134625376	1
GO:1900271	regulation of long-term synaptic potentiation	0.207353623	0.134625376	1
GO:1904036	negative regulation of epithelial cell apoptotic process	0.207353623	0.134625376	1
GO:1990573	potassium ion import across plasma membrane	0.207353623	0.134625376	1
GO:1990928	response to amino acid starvation	0.207353623	0.134625376	1
GO:0009395	phospholipid catabolic process	0.211186699	0.136672666	1
GO:0010712	regulation of collagen metabolic process	0.211186699	0.136672666	1
GO:0018198	peptidyl-cysteine modification	0.211186699	0.136672666	1
GO:0047496	vesicle transport along microtubule	0.211186699	0.136672666	1
GO:0048641	regulation of skeletal muscle tissue development	0.211186699	0.136672666	1
GO:0061383	trabecula morphogenesis	0.211186699	0.136672666	1

GO:0061647	histone H3-K9 modification	0.211186699	0.136672666	1
GO:1990090	cellular response to nerve growth factor stimulus	0.211186699	0.136672666	1
GO:2000107	negative regulation of leukocyte apoptotic process	0.211186699	0.136672666	1
GO:0032984	protein-containing complex disassembly	0.213303078	0.137992959	1
GO:0001961	positive regulation of cytokine-mediated signaling pathway	0.215001441	0.13859617	1
GO:0002711	positive regulation of T cell mediated immunity	0.215001441	0.13859617	1
GO:0008038	neuron recognition	0.215001441	0.13859617	1
GO:0010718	positive regulation of epithelial to mesenchymal transition	0.215001441	0.13859617	1
GO:0019674	NAD metabolic process	0.215001441	0.13859617	1
GO:0045540	regulation of cholesterol biosynthetic process	0.215001441	0.13859617	1
GO:0071715	icosanoid transport	0.215001441	0.13859617	1
GO:0106118	regulation of sterol biosynthetic process	0.215001441	0.13859617	1
GO:1901571	fatty acid derivative transport	0.215001441	0.13859617	1
GO:1903727	positive regulation of phospholipid metabolic process	0.215001441	0.13859617	1
GO:0031113	regulation of microtubule polymerization	0.218797937	0.140542815	1
GO:0032873	negative regulation of stress-activated MAPK cascade	0.218797937	0.140542815	1
GO:0035176	social behavior	0.218797937	0.140542815	1
GO:0043277	apoptotic cell clearance	0.218797937	0.140542815	1
GO:0048255	mRNA stabilization	0.218797937	0.140542815	1
GO:0060976	coronary vasculature development	0.218797937	0.140542815	1
GO:0070303	negative regulation of stress-activated protein kinase signaling cascade	0.218797937	0.140542815	1
GO:0071827	plasma lipoprotein particle organization	0.218797937	0.140542815	1

GO:0072523	purine-containing compound catabolic process	0.218797937	0.140542815	1
GO:1903587	regulation of blood vessel endothelial cell proliferation involved in sprouting angiogenesis	0.218797937	0.140542815	1
GO:0043433	negative regulation of DNA-binding transcription factor activity	0.220867423	0.141821785	1
GO:0001912	positive regulation of leukocyte mediated cytotoxicity	0.222576273	0.142253738	1
GO:0003179	heart valve morphogenesis	0.222576273	0.142253738	1
GO:0032330	regulation of chondrocyte differentiation	0.222576273	0.142253738	1
GO:0033059	cellular pigmentation	0.222576273	0.142253738	1
GO:0046580	negative regulation of Ras protein signal transduction	0.222576273	0.142253738	1
GO:0048260	positive regulation of receptor-mediated endocytosis	0.222576273	0.142253738	1
GO:0051339	regulation of lyase activity	0.222576273	0.142253738	1
GO:0051445	regulation of meiotic cell cycle	0.222576273	0.142253738	1
GO:0060425	lung morphogenesis	0.222576273	0.142253738	1
GO:1990089	response to nerve growth factor	0.222576273	0.142253738	1
GO:2001238	positive regulation of extrinsic apoptotic signaling pathway	0.222576273	0.142253738	1
GO:2001258	negative regulation of cation channel activity	0.222576273	0.142253738	1
GO:0046328	regulation of JNK cascade	0.222640754	0.142253738	1
GO:0090090	negative regulation of canonical Wnt signaling pathway	0.222640754	0.142253738	1
GO:1990778	protein localization to cell periphery	0.225917577	0.143954307	1
GO:0007632	visual behavior	0.226336534	0.143954307	1
GO:0008333	endosome to lysosome transport	0.226336534	0.143954307	1
GO:0032964	collagen biosynthetic process	0.226336534	0.143954307	1
GO:0035384	thioester biosynthetic process	0.226336534	0.143954307	1

GO:0043392	negative regulation of DNA binding	0.226336534	0.143954307	1
GO:0045668	negative regulation of osteoblast differentiation	0.226336534	0.143954307	1
GO:0051703	intraspecies interaction between organisms	0.226336534	0.143954307	1
GO:0071320	cellular response to cAMP	0.226336534	0.143954307	1
GO:0071616	acyl-CoA biosynthetic process	0.226336534	0.143954307	1
GO:0072698	protein localization to microtubule cytoskeleton	0.226336534	0.143954307	1
GO:0097035	regulation of membrane lipid distribution	0.226336534	0.143954307	1
GO:2000772	regulation of cellular senescence	0.226336534	0.143954307	1
GO:0002285	lymphocyte activation involved in immune response	0.227967717	0.144889914	1
GO:0043409	negative regulation of MAPK cascade	0.227967717	0.144889914	1
GO:0031345	negative regulation of cell projection organization	0.229745522	0.145968566	1
GO:0009620	response to fungus	0.230078807	0.146026498	1
GO:0010524	positive regulation of calcium ion transport into cytosol	0.230078807	0.146026498	1
GO:1903018	regulation of glycoprotein metabolic process	0.230078807	0.146026498	1
GO:0099111	microtubule-based transport	0.233304054	0.14792332	1
GO:0002720	positive regulation of cytokine production involved in immune response	0.233803176	0.14792332	1
GO:0003229	ventricular cardiac muscle tissue development	0.233803176	0.14792332	1
GO:0008347	glial cell migration	0.233803176	0.14792332	1
GO:0032655	regulation of interleukin-12 production	0.233803176	0.14792332	1
GO:0032781	positive regulation of ATPase activity	0.233803176	0.14792332	1
GO:0035065	regulation of histone acetylation	0.233803176	0.14792332	1
GO:0050433	regulation of catecholamine secretion	0.233803176	0.14792332	1

GO:0071825	protein-lipid complex subunit organization	0.233803176	0.14792332	1
GO:0048167	regulation of synaptic plasticity	0.23508468	0.148682119	1
GO:0016525	negative regulation of angiogenesis	0.236866148	0.149756485	1
GO:0002712	regulation of B cell mediated immunity	0.237509726	0.149797002	1
GO:0002889	regulation of immunoglobulin mediated immune response	0.237509726	0.149797002	1
GO:0010665	regulation of cardiac muscle cell apoptotic process	0.237509726	0.149797002	1
GO:0019320	hexose catabolic process	0.237509726	0.149797002	1
GO:0030071	regulation of mitotic metaphase/anaphase transition	0.237509726	0.149797002	1
GO:0031295	T cell costimulation	0.237509726	0.149797002	1
GO:0050732	negative regulation of peptidyl-tyrosine phosphorylation	0.237509726	0.149797002	1
GO:0061136	regulation of proteasomal protein catabolic process	0.238648407	0.150462722	1
GO:2000181	negative regulation of blood vessel morphogenesis	0.24043141	0.151534068	1
GO:0002090	regulation of receptor internalization	0.241198541	0.151595142	1
GO:0006576	cellular biogenic amine metabolic process	0.241198541	0.151595142	1
GO:0044380	protein localization to cytoskeleton	0.241198541	0.151595142	1
GO:0045840	positive regulation of mitotic nuclear division	0.241198541	0.151595142	1
GO:0050432	catecholamine secretion	0.241198541	0.151595142	1
GO:0060760	positive regulation of response to cytokine stimulus	0.241198541	0.151595142	1
GO:0061005	cell differentiation involved in kidney development	0.241198541	0.151595142	1
GO:0097479	synaptic vesicle localization	0.241198541	0.151595142	1
GO:1901136	carbohydrate derivative catabolic process	0.242215109	0.152181203	1
GO:0050728	negative regulation of inflammatory response	0.243999456	0.153104799	1

GO:0000768	syncytium formation by plasma membrane fusion	0.244869706	0.153104799	1
GO:0002043	blood vessel endothelial cell proliferation involved in sprouting angiogenesis	0.244869706	0.153104799	1
GO:0007091	metaphase/anaphase transition of mitotic cell cycle	0.244869706	0.153104799	1
GO:0010656	negative regulation of muscle cell apoptotic process	0.244869706	0.153104799	1
GO:0010662	regulation of striated muscle cell apoptotic process	0.244869706	0.153104799	1
GO:0031294	lymphocyte costimulation	0.244869706	0.153104799	1
GO:0035306	positive regulation of dephosphorylation	0.244869706	0.153104799	1
GO:0043489	RNA stabilization	0.244869706	0.153104799	1
GO:0140253	cell-cell fusion	0.244869706	0.153104799	1
GO:1902099	regulation of metaphase/anaphase transition of cell cycle	0.244869706	0.153104799	1
GO:1902373	negative regulation of mRNA catabolic process	0.244869706	0.153104799	1
GO:1903793	positive regulation of anion transport	0.244869706	0.153104799	1
GO:2000272	negative regulation of signaling receptor activity	0.244869706	0.153104799	1
GO:0006164	purine nucleotide biosynthetic process	0.245784404	0.153570584	1
GO:0006694	steroid biosynthetic process	0.245784404	0.153570584	1
GO:0010659	cardiac muscle cell apoptotic process	0.248523303	0.154907467	1
GO:0043030	regulation of macrophage activation	0.248523303	0.154907467	1
GO:0045815	positive regulation of gene expression, epigenetic	0.248523303	0.154907467	1
GO:0046605	regulation of centrosome cycle	0.248523303	0.154907467	1
GO:0050707	regulation of cytokine secretion	0.248523303	0.154907467	1
GO:0051058	negative regulation of small GTPase mediated signal transduction	0.248523303	0.154907467	1
GO:0071385	cellular response to glucocorticoid stimulus	0.248523303	0.154907467	1

GO:0099504	synaptic vesicle cycle	0.249355916	0.155372923	1
GO:0030705	cytoskeleton-dependent intracellular transport	0.251142387	0.1564322	1
GO:0006949	syncytium formation	0.252159416	0.156580586	1
GO:0018208	peptidyl-proline modification	0.252159416	0.156580586	1
GO:0030865	cortical cytoskeleton organization	0.252159416	0.156580586	1
GO:0032653	regulation of interleukin-10 production	0.252159416	0.156580586	1
GO:0042093	T-helper cell differentiation	0.252159416	0.156580586	1
GO:0043388	positive regulation of DNA binding	0.252159416	0.156580586	1
GO:0043966	histone H3 acetylation	0.252159416	0.156580586	1
GO:0044784	metaphase/anaphase transition of cell cycle	0.252159416	0.156580586	1
GO:0071806	protein transmembrane transport	0.252159416	0.156580586	1
GO:0043687	post-translational protein modification	0.255445703	0.158176279	1
GO:0003170	heart valve development	0.255778128	0.158176279	1
GO:0010658	striated muscle cell apoptotic process	0.255778128	0.158176279	1
GO:0010965	regulation of mitotic sister chromatid separation	0.255778128	0.158176279	1
GO:0031343	positive regulation of cell killing	0.255778128	0.158176279	1
GO:0042733	embryonic digit morphogenesis	0.255778128	0.158176279	1
GO:0045071	negative regulation of viral genome replication	0.255778128	0.158176279	1
GO:0046847	filopodium assembly	0.255778128	0.158176279	1
GO:0046888	negative regulation of hormone secretion	0.255778128	0.158176279	1
GO:0051310	metaphase plate congression	0.255778128	0.158176279	1
GO:0090342	regulation of cell aging	0.255778128	0.158176279	1

GO:2000756	regulation of peptidyl-lysine acetylation	0.255778128	0.158176279	1
GO:0002449	lymphocyte mediated immunity	0.259340647	0.160020599	1
GO:0002294	CD4-positive, alpha-beta T cell differentiation involved in immune response	0.25937952	0.160020599	1
GO:0034113	heterotypic cell-cell adhesion	0.25937952	0.160020599	1
GO:0051851	modulation by host of symbiont process	0.25937952	0.160020599	1
GO:0055081	anion homeostasis	0.25937952	0.160020599	1
GO:0071384	cellular response to corticosteroid stimulus	0.25937952	0.160020599	1
GO:0140115	export across plasma membrane	0.25937952	0.160020599	1
GO:0002287	alpha-beta T cell activation involved in immune response	0.262963676	0.162010845	1
GO:0002293	alpha-beta T cell differentiation involved in immune response	0.262963676	0.162010845	1
GO:0006081	cellular aldehyde metabolic process	0.262963676	0.162010845	1
GO:0046530	photoreceptor cell differentiation	0.262963676	0.162010845	1
GO:0002460	adaptive immune response based on somatic recombination of immune receptors built from immunoglobulin superfamily domains	0.264546762	0.162930703	1
GO:0072522	purine-containing compound biosynthetic process	0.265445424	0.16342855	1
GO:0001947	heart looping	0.266530676	0.163706804	1
GO:0032507	maintenance of protein location in cell	0.266530676	0.163706804	1
GO:0032835	glomerulus development	0.266530676	0.163706804	1
GO:0033344	cholesterol efflux	0.266530676	0.163706804	1
GO:0042130	negative regulation of T cell proliferation	0.266530676	0.163706804	1
GO:0051306	mitotic sister chromatid separation	0.266530676	0.163706804	1
GO:0051898	negative regulation of protein kinase B signaling	0.266530676	0.163706804	1
GO:0040029	regulation of gene expression, epigenetic	0.267234057	0.164083133	1

GO:0002377	immunoglobulin production	0.269022716	0.165125348	1
GO:0007588	excretion	0.270080602	0.165158413	1
GO:0017156	calcium-ion regulated exocytosis	0.270080602	0.165158413	1
GO:0030837	negative regulation of actin filament polymerization	0.270080602	0.165158413	1
GO:0031640	killing of cells of other organism	0.270080602	0.165158413	1
GO:0031646	positive regulation of nervous system process	0.270080602	0.165158413	1
GO:0032729	positive regulation of interferon-gamma production	0.270080602	0.165158413	1
GO:0046782	regulation of viral transcription	0.270080602	0.165158413	1
GO:0048247	lymphocyte chemotaxis	0.270080602	0.165158413	1
GO:0048645	animal organ formation	0.270080602	0.165158413	1
GO:0090181	regulation of cholesterol metabolic process	0.270080602	0.165158413	1
GO:1902305	regulation of sodium ion transmembrane transport	0.270080602	0.165158413	1
GO:0031396	regulation of protein ubiquitination	0.270811359	0.165549334	1
GO:0032715	negative regulation of interleukin-6 production	0.273613535	0.166980267	1
GO:0045669	positive regulation of osteoblast differentiation	0.273613535	0.166980267	1
GO:0046365	monosaccharide catabolic process	0.273613535	0.166980267	1
GO:0050922	negative regulation of chemotaxis	0.273613535	0.166980267	1
GO:1905818	regulation of chromosome separation	0.273613535	0.166980267	1
GO:0007254	JNK cascade	0.274388435	0.167340294	1
GO:0045089	positive regulation of innate immune response	0.274388435	0.167340294	1
GO:0009166	nucleotide catabolic process	0.277129555	0.168500886	1
GO:0032890	regulation of organic acid transport	0.277129555	0.168500886	1

GO:0033866	nucleoside bisphosphate biosynthetic process	0.277129555	0.168500886	1
GO:0034030	ribonucleoside bisphosphate biosynthetic process	0.277129555	0.168500886	1
GO:0034033	purine nucleoside bisphosphate biosynthetic process	0.277129555	0.168500886	1
GO:0034332	adherens junction organization	0.277129555	0.168500886	1
GO:0036498	IRE1-mediated unfolded protein response	0.277129555	0.168500886	1
GO:0042255	ribosome assembly	0.277129555	0.168500886	1
GO:0071300	cellular response to retinoic acid	0.277129555	0.168500886	1
GO:0006909	phagocytosis	0.280241761	0.17017102	1
GO:0006625	protein targeting to peroxisome	0.280628741	0.17017102	1
GO:0010812	negative regulation of cell-substrate adhesion	0.280628741	0.17017102	1
GO:0042446	hormone biosynthetic process	0.280628741	0.17017102	1
GO:0051148	negative regulation of muscle cell differentiation	0.280628741	0.17017102	1
GO:0072662	protein localization to peroxisome	0.280628741	0.17017102	1
GO:0072663	establishment of protein localization to peroxisome	0.280628741	0.17017102	1
GO:1902369	negative regulation of RNA catabolic process	0.280628741	0.17017102	1
GO:0035914	skeletal muscle cell differentiation	0.284111175	0.172052106	1
GO:0050854	regulation of antigen receptor-mediated signaling pathway	0.284111175	0.172052106	1
GO:0061371	determination of heart left/right asymmetry	0.284111175	0.172052106	1
GO:0071230	cellular response to amino acid stimulus	0.284111175	0.172052106	1
GO:0007163	establishment or maintenance of cell polarity	0.286901806	0.173625842	1
GO:0099003	vesicle-mediated transport in synapse	0.286901806	0.173625842	1
GO:0001756	somitogenesis	0.287576934	0.173685879	1

GO:0003143	embryonic heart tube morphogenesis	0.287576934	0.173685879	1
GO:0032374	regulation of cholesterol transport	0.287576934	0.173685879	1
GO:0043967	histone H4 acetylation	0.287576934	0.173685879	1
GO:0050771	negative regulation of axonogenesis	0.287576934	0.173685879	1
GO:0050795	regulation of behavior	0.287576934	0.173685879	1
GO:0002292	T cell differentiation involved in immune response	0.291026099	0.175476197	1
GO:0032371	regulation of sterol transport	0.291026099	0.175476197	1
GO:0033627	cell adhesion mediated by integrin	0.291026099	0.175476197	1
GO:0050891	multicellular organismal water homeostasis	0.291026099	0.175476197	1
GO:0051785	positive regulation of nuclear division	0.291026099	0.175476197	1
GO:0019226	transmission of nerve impulse	0.294458747	0.177250617	1
GO:0033047	regulation of mitotic sister chromatid segregation	0.294458747	0.177250617	1
GO:0042440	pigment metabolic process	0.294458747	0.177250617	1
GO:0043574	peroxisomal transport	0.294458747	0.177250617	1
GO:0061035	regulation of cartilage development	0.294458747	0.177250617	1
GO:1903050	regulation of proteolysis involved in cellular protein catabolic process	0.295828452	0.178015896	1
GO:0002709	regulation of T cell mediated immunity	0.297874957	0.179068736	1
GO:1901292	nucleoside phosphate catabolic process	0.297874957	0.179068736	1
GO:1902117	positive regulation of organelle assembly	0.297874957	0.179068736	1
GO:0016064	immunoglobulin mediated immune response	0.299395224	0.179922876	1
GO:0006305	DNA alkylation	0.301274807	0.180572652	1
GO:0006306	DNA methylation	0.301274807	0.180572652	1

GO:0006635	fatty acid beta-oxidation	0.301274807	0.180572652	1
GO:0032024	positive regulation of insulin secretion	0.301274807	0.180572652	1
GO:0032481	positive regulation of type I interferon production	0.301274807	0.180572652	1
GO:0034121	regulation of toll-like receptor signaling pathway	0.301274807	0.180572652	1
GO:0042273	ribosomal large subunit biogenesis	0.301274807	0.180572652	1
GO:0043507	positive regulation of JUN kinase activity	0.301274807	0.180572652	1
GO:0001910	regulation of leukocyte mediated cytotoxicity	0.304658374	0.182106872	1
GO:0006695	cholesterol biosynthetic process	0.304658374	0.182106872	1
GO:0009064	glutamine family amino acid metabolic process	0.304658374	0.182106872	1
GO:0015909	long-chain fatty acid transport	0.304658374	0.182106872	1
GO:0048844	artery morphogenesis	0.304658374	0.182106872	1
GO:0061844	antimicrobial humoral immune response mediated by antimicrobial peptide	0.304658374	0.182106872	1
GO:0099518	vesicle cytoskeletal trafficking	0.304658374	0.182106872	1
GO:1902653	secondary alcohol biosynthetic process	0.304658374	0.182106872	1
GO:0019724	B cell mediated immunity	0.30474033	0.182106872	1
GO:0032271	regulation of protein polymerization	0.306520547	0.183110222	1
GO:0003281	ventricular septum development	0.308025736	0.183887979	1
GO:0006278	RNA-dependent DNA biosynthetic process	0.308025736	0.183887979	1
GO:0018205	peptidyl-lysine modification	0.310549995	0.185333788	1
GO:0046785	microtubule polymerization	0.311376968	0.185704822	1
GO:0070830	bicellular tight junction assembly	0.311376968	0.185704822	1
GO:0043367	CD4-positive, alpha-beta T cell differentiation	0.314712148	0.187632076	1

GO:0044242	cellular lipid catabolic process	0.315408981	0.18798559	1
GO:0002831	regulation of response to biotic stimulus	0.31583908	0.188179949	1
GO:0006826	iron ion transport	0.318031352	0.188926251	1
GO:0007032	endosome organization	0.318031352	0.188926251	1
GO:0030104	water homeostasis	0.318031352	0.188926251	1
GO:0043407	negative regulation of MAP kinase activity	0.318031352	0.188926251	1
GO:0050000	chromosome localization	0.318031352	0.188926251	1
GO:0050672	negative regulation of lymphocyte proliferation	0.318031352	0.188926251	1
GO:0051303	establishment of chromosome localization	0.318031352	0.188926251	1
GO:0120192	tight junction assembly	0.318031352	0.188926251	1
GO:1900034	regulation of cellular response to heat	0.318031352	0.188926251	1
GO:0002312	B cell activation involved in immune response	0.321334654	0.19063823	1
GO:0031397	negative regulation of protein ubiquitination	0.321334654	0.19063823	1
GO:0032945	negative regulation of mononuclear cell proliferation	0.321334654	0.19063823	1
GO:0035050	embryonic heart tube development	0.321334654	0.19063823	1
GO:0006323	DNA packaging	0.322502596	0.191268424	1
GO:0001960	negative regulation of cytokine-mediated signaling pathway	0.324622131	0.192273389	1
GO:0016126	sterol biosynthetic process	0.324622131	0.192273389	1
GO:0043242	negative regulation of protein-containing complex disassembly	0.324622131	0.192273389	1
GO:0051279	regulation of release of sequestered calcium ion into cytosol	0.324622131	0.192273389	1
GO:0006813	potassium ion transport	0.326042999	0.193051776	1
GO:0065004	protein-DNA complex assembly	0.327811492	0.193767246	1

GO:0016925	protein sumoylation	0.327893858	0.193767246	1
GO:0021954	central nervous system neuron development	0.327893858	0.193767246	1
GO:0045652	regulation of megakaryocyte differentiation	0.327893858	0.193767246	1
GO:0120193	tight junction organization	0.327893858	0.193767246	1
GO:1903313	positive regulation of mRNA metabolic process	0.327893858	0.193767246	1
GO:0002088	lens development in camera-type eye	0.331149909	0.195436088	1
GO:0007031	peroxisome organization	0.331149909	0.195436088	1
GO:0031145	anaphase-promoting complex-dependent catabolic process	0.331149909	0.195436088	1
GO:2001021	negative regulation of response to DNA damage stimulus	0.331149909	0.195436088	1
GO:0033045	regulation of sister chromatid segregation	0.334390358	0.197284171	1
GO:0016358	dendrite development	0.334873382	0.19750475	1
GO:0043161	proteasome-mediated ubiquitin-dependent protein catabolic process	0.33568796	0.197920668	1
GO:0030101	natural killer cell activation	0.33761528	0.198733235	1
GO:0031110	regulation of microtubule polymerization or depolymerization	0.33761528	0.198733235	1
GO:0034502	protein localization to chromosome	0.33761528	0.198733235	1
GO:0043297	apical junction assembly	0.33761528	0.198733235	1
GO:2000779	regulation of double-strand break repair	0.33761528	0.198733235	1
GO:0035023	regulation of Rho protein signal transduction	0.340824747	0.200426848	1
GO:0070664	negative regulation of leukocyte proliferation	0.340824747	0.200426848	1
GO:1903901	negative regulation of viral life cycle	0.340824747	0.200426848	1
GO:0030512	negative regulation of transforming growth factor beta receptor signaling pathway	0.344018834	0.201911459	1
GO:0048013	ephrin receptor signaling pathway	0.344018834	0.201911459	1

GO:0051262	protein tetramerization	0.344018834	0.201911459	1
GO:0060218	hematopoietic stem cell differentiation	0.344018834	0.201911459	1
GO:0060291	long-term synaptic potentiation	0.344018834	0.201911459	1
GO:0060761	negative regulation of response to cytokine stimulus	0.344018834	0.201911459	1
GO:1903312	negative regulation of mRNA metabolic process	0.347197613	0.203711071	1
GO:0043506	regulation of JUN kinase activity	0.350361156	0.205300937	1
GO:0043537	negative regulation of blood vessel endothelial cell migration	0.350361156	0.205300937	1
GO:0061053	somite development	0.350361156	0.205300937	1
GO:1903845	negative regulation of cellular response to transforming growth factor beta stimulus	0.350361156	0.205300937	1
GO:0001656	metanephros development	0.356642825	0.208509146	1
GO:0001843	neural tube closure	0.356642825	0.208509146	1
GO:0015914	phospholipid transport	0.356642825	0.208509146	1
GO:0030901	midbrain development	0.356642825	0.208509146	1
GO:0045682	regulation of epidermis development	0.356642825	0.208509146	1
GO:0051952	regulation of amine transport	0.356642825	0.208509146	1
GO:1901379	regulation of potassium ion transmembrane transport	0.356642825	0.208509146	1
GO:1903362	regulation of cellular protein catabolic process	0.357672301	0.209043481	1
GO:0031341	regulation of cell killing	0.359761094	0.209992978	1
GO:0060606	tube closure	0.359761094	0.209992978	1
GO:0106027	neuron projection organization	0.359761094	0.209992978	1
GO:1905897	regulation of response to endoplasmic reticulum stress	0.359761094	0.209992978	1
GO:0006302	double-strand break repair	0.36115662	0.210739568	1

GO:0045185	maintenance of protein location	0.362864415	0.21166783	1
GO:0051304	chromosome separation	0.365952858	0.213400601	1
GO:0048814	regulation of dendrite morphogenesis	0.369026494	0.215054341	1
GO:1903725	regulation of phospholipid metabolic process	0.369026494	0.215054341	1
GO:0010972	negative regulation of G2/M transition of mitotic cell cycle	0.372085392	0.216767136	1
GO:0031348	negative regulation of defense response	0.373297237	0.21740313	1
GO:0035710	CD4-positive, alpha-beta T cell activation	0.375129624	0.218399995	1
GO:0006644	phospholipid metabolic process	0.376602646	0.219116584	1
GO:0007389	pattern specification process	0.376602646	0.219116584	1
GO:0010771	negative regulation of cell morphogenesis involved in differentiation	0.378159258	0.219669094	1
GO:0035278	miRNA mediated inhibition of translation	0.378159258	0.219669094	1
GO:0040033	negative regulation of translation, ncRNA-mediated	0.378159258	0.219669094	1
GO:0044728	DNA methylation or demethylation	0.378159258	0.219669094	1
GO:0045974	regulation of translation, ncRNA-mediated	0.378159258	0.219669094	1
GO:0010717	regulation of epithelial to mesenchymal transition	0.381174364	0.221136576	1
GO:0015837	amine transport	0.381174364	0.221136576	1
GO:0062207	regulation of pattern recognition receptor signaling pathway	0.381174364	0.221136576	1
GO:0090277	positive regulation of peptide hormone secretion	0.381174364	0.221136576	1
GO:0002702	positive regulation of production of molecular mediator of immune response	0.384175011	0.22252067	1
GO:0030219	megakaryocyte differentiation	0.384175011	0.22252067	1
GO:0035282	segmentation	0.384175011	0.22252067	1
GO:0045807	positive regulation of endocytosis	0.384175011	0.22252067	1

GO:0061640	cytoskeleton-dependent cytokinesis	0.384175011	0.22252067	1
GO:0001578	microtubule bundle formation	0.387161266	0.224035217	1
GO:0001708	cell fate specification	0.387161266	0.224035217	1
GO:0031124	mRNA 3'-end processing	0.387161266	0.224035217	1
GO:0032006	regulation of TOR signaling	0.390133199	0.225610661	1
GO:0060840	artery development	0.390133199	0.225610661	1
GO:0071824	protein-DNA complex subunit organization	0.395597602	0.228584934	1
GO:0002456	T cell mediated immunity	0.396034369	0.228584934	1
GO:0006637	acyl-CoA metabolic process	0.396034369	0.228584934	1
GO:0032649	regulation of interferon-gamma production	0.396034369	0.228584934	1
GO:0035383	thioester metabolic process	0.396034369	0.228584934	1
GO:1901606	alpha-amino acid catabolic process	0.396034369	0.228584934	1
GO:0002697	regulation of immune effector process	0.396250325	0.22863665	1
GO:0030301	cholesterol transport	0.398963741	0.229855387	1
GO:0048259	regulation of receptor-mediated endocytosis	0.398963741	0.229855387	1
GO:0097006	regulation of plasma lipoprotein particle levels	0.398963741	0.229855387	1
GO:2001022	positive regulation of response to DNA damage stimulus	0.398963741	0.229855387	1
GO:0007411	axon guidance	0.398997662	0.229855387	1
GO:0097485	neuron projection guidance	0.400694457	0.230759413	1
GO:0007218	neuropeptide signaling pathway	0.40187906	0.231294397	1
GO:0034446	substrate adhesion-dependent cell spreading	0.40187906	0.231294397	1
GO:0000018	regulation of DNA recombination	0.404780393	0.232446674	1

GO:0010596	negative regulation of endothelial cell migration	0.404780393	0.232446674	1
GO:0034404	nucleobase-containing small molecule biosynthetic process	0.404780393	0.232446674	1
GO:0048524	positive regulation of viral process	0.404780393	0.232446674	1
GO:0050905	neuromuscular process	0.404780393	0.232446674	1
GO:0051983	regulation of chromosome segregation	0.404780393	0.232446674	1
GO:0099175	regulation of postsynapse organization	0.404780393	0.232446674	1
GO:0001909	leukocyte mediated cytotoxicity	0.407667806	0.233882108	1
GO:0007229	integrin-mediated signaling pathway	0.407667806	0.233882108	1
GO:0008593	regulation of Notch signaling pathway	0.407667806	0.233882108	1
GO:0010976	positive regulation of neuron projection development	0.409145347	0.234655385	1
GO:0021510	spinal cord development	0.410541366	0.235306873	1
GO:0098781	ncRNA transcription	0.410541366	0.235306873	1
GO:0010498	proteasomal protein catabolic process	0.413153897	0.236721038	1
GO:0006289	nucleotide-excision repair	0.413401137	0.236721038	1
GO:0007041	lysosomal transport	0.413401137	0.236721038	1
GO:0060828	regulation of canonical Wnt signaling pathway	0.41753946	0.239015079	1
GO:1903510	mucopolysaccharide metabolic process	0.421898377	0.241433906	1
GO:0006310	DNA recombination	0.424212445	0.242681396	1
GO:0009062	fatty acid catabolic process	0.424703648	0.242885612	1
GO:0051258	protein polymerization	0.42587468	0.243478365	1
GO:0034660	ncRNA metabolic process	0.426061311	0.243508127	1
GO:0032609	interferon-gamma production	0.427495455	0.24425064	1

GO:0032392	DNA geometric change	0.430273863	0.24576049	1
GO:0031109	microtubule polymerization or depolymerization	0.433038933	0.247261772	1
GO:0007052	mitotic spindle organization	0.435790731	0.248444281	1
GO:0015918	sterol transport	0.435790731	0.248444281	1
GO:0050868	negative regulation of T cell activation	0.435790731	0.248444281	1
GO:0002440	production of molecular mediator of immune response	0.435796416	0.248444281	1
GO:0030216	keratinocyte differentiation	0.435796416	0.248444281	1
GO:0045088	regulation of innate immune response	0.437441288	0.249303464	1
GO:0006304	DNA modification	0.441254759	0.251397631	1
GO:0090287	regulation of cellular response to growth factor stimulus	0.442360619	0.251948347	1
GO:0031398	positive regulation of protein ubiquitination	0.443967115	0.252783767	1
GO:0002698	negative regulation of immune effector process	0.452026292	0.257129733	1
GO:0009063	cellular amino acid catabolic process	0.452026292	0.257129733	1
GO:0010633	negative regulation of epithelial cell migration	0.452026292	0.257129733	1
GO:0007098	centrosome cycle	0.454686926	0.258399506	1
GO:0007368	determination of left/right symmetry	0.454686926	0.258399506	1
GO:0032479	regulation of type I interferon production	0.454686926	0.258399506	1
GO:0017015	regulation of transforming growth factor beta receptor signaling pathway	0.459969924	0.261073862	1
GO:0030326	embryonic limb morphogenesis	0.459969924	0.261073862	1
GO:0032606	type I interferon production	0.459969924	0.261073862	1
GO:0035113	embryonic appendage morphogenesis	0.459969924	0.261073862	1
GO:0000041	transition metal ion transport	0.465202297	0.263878155	1

GO:1903844	regulation of cellular response to transforming growth factor beta stimulus	0.465202297	0.263878155	1
GO:0071426	ribonucleoprotein complex export from nucleus	0.467799649	0.265268303	1
GO:0046887	positive regulation of hormone secretion	0.470384525	0.266566996	1
GO:0071166	ribonucleoprotein complex localization	0.470384525	0.266566996	1
GO:0006282	regulation of DNA repair	0.472956983	0.267940894	1
GO:0090101	negative regulation of transmembrane receptor protein serine/threonine kinase signaling pathway	0.475517084	0.269306932	1
GO:0002920	regulation of humoral immune response	0.478064884	0.270411324	1
GO:0007498	mesoderm development	0.478064884	0.270411324	1
GO:0009855	determination of bilateral symmetry	0.478064884	0.270411324	1
GO:0045995	regulation of embryonic development	0.478064884	0.270411324	1
GO:0009799	specification of symmetry	0.480600443	0.271590835	1
GO:0015748	organophosphate ester transport	0.480600443	0.271590835	1
GO:0042157	lipoprotein metabolic process	0.480600443	0.271590835	1
GO:0031023	microtubule organizing center organization	0.483123819	0.272931576	1
GO:0019730	antimicrobial humoral response	0.48563507	0.274093537	1
GO:0030534	adult behavior	0.48563507	0.274093537	1
GO:0042552	myelination	0.48563507	0.274093537	1
GO:0000724	double-strand break repair via homologous recombination	0.488134254	0.274989442	1
GO:0033865	nucleoside bisphosphate metabolic process	0.488134254	0.274989442	1
GO:0033875	ribonucleoside bisphosphate metabolic process	0.488134254	0.274989442	1
GO:0034032	purine nucleoside bisphosphate metabolic process	0.488134254	0.274989442	1
GO:0046330	positive regulation of JNK cascade	0.488134254	0.274989442	1

GO:0072329	monocarboxylic acid catabolic process	0.488134254	0.274989442	1
GO:0060070	canonical Wnt signaling pathway	0.488668471	0.275204712	1
GO:0016050	vesicle organization	0.49022321	0.275875252	1
GO:0002433	immune response-regulating cell surface receptor signaling pathway involved in phagocytosis	0.490621427	0.275875252	1
GO:0007272	ensheathment of neurons	0.490621427	0.275875252	1
GO:0008366	axon ensheathment	0.490621427	0.275875252	1
GO:0038096	Fc-gamma receptor signaling pathway involved in phagocytosis	0.490621427	0.275875252	1
GO:0000725	recombinational repair	0.493096648	0.277094847	1
GO:0048284	organelle fusion	0.493096648	0.277094847	1
GO:0006650	glycerophospholipid metabolic process	0.494869965	0.278005023	1
GO:1903322	positive regulation of protein modification by small protein conjugation or removal	0.495559972	0.278306247	1
GO:0038094	Fc-gamma receptor signaling pathway	0.498011456	0.279596222	1
GO:0006405	RNA export from nucleus	0.50287913	0.28215397	1
GO:1902850	microtubule cytoskeleton organization involved in mitosis	0.50287913	0.28215397	1
GO:0002431	Fc receptor mediated stimulatory signaling pathway	0.505295432	0.283421818	1
GO:0044409	entry into host	0.510093241	0.286024263	1
GO:2001251	negative regulation of chromosome organization	0.51247486	0.287270685	1
GO:0003002	regionalization	0.513191285	0.287494156	1
GO:0071103	DNA conformation change	0.513191285	0.287494156	1
GO:0007034	vacuolar transport	0.517203797	0.28938363	1
GO:0035107	appendage morphogenesis	0.517203797	0.28938363	1
GO:0035108	limb morphogenesis	0.517203797	0.28938363	1

GO:1905039	carboxylic acid transmembrane transport	0.517203797	0.28938363	1
GO:0035567	non-canonical Wnt signaling pathway	0.519551225	0.290517388	1
GO:1903825	organic acid transmembrane transport	0.519551225	0.290517388	1
GO:0001818	negative regulation of cytokine production	0.520697685	0.291068507	1
GO:0007605	sensory perception of sound	0.521887364	0.291553396	1
GO:0050684	regulation of mRNA processing	0.521887364	0.291553396	1
GO:0031123	RNA 3'-end processing	0.524212267	0.292761822	1
GO:0010977	negative regulation of neuron projection development	0.52652599	0.293872586	1
GO:0051250	negative regulation of lymphocyte activation	0.52652599	0.293872586	1
GO:0009913	epidermal cell differentiation	0.528127942	0.294675798	1
GO:0002455	humoral immune response mediated by circulating immunoglobulin	0.528828583	0.294884871	1
GO:0050773	regulation of dendrite development	0.528828583	0.294884871	1
GO:0034401	chromatin organization involved in regulation of transcription	0.531120102	0.296071426	1
GO:0007018	microtubule-based movement	0.532549262	0.296776679	1
GO:0030203	glycosaminoglycan metabolic process	0.535670122	0.29842395	1
GO:0032259	methylation	0.536942792	0.29904089	1
GO:0050777	negative regulation of immune response	0.537928729	0.299405684	1
GO:1902600	proton transmembrane transport	0.537928729	0.299405684	1
GO:1902903	regulation of supramolecular fiber organization	0.539856311	0.300386158	1
GO:0000070	mitotic sister chromatid segregation	0.542413396	0.301623468	1
GO:0000723	telomere maintenance	0.542413396	0.301623468	1
GO:0016482	cytosolic transport	0.551253981	0.306445337	1

GO:0048515	spermatid differentiation	0.553437602	0.307564735	1
GO:0052126	movement in host environment	0.559925607	0.310979328	1
GO:0061025	membrane fusion	0.559925607	0.310979328	1
GO:0000910	cytokinesis	0.56632037	0.314241584	1
GO:0001906	cell killing	0.56632037	0.314241584	1
GO:0006022	aminoglycan metabolic process	0.56632037	0.314241584	1
GO:0009152	purine ribonucleotide biosynthetic process	0.568431467	0.315123095	1
GO:0050954	sensory perception of mechanical stimulus	0.568431467	0.315123095	1
GO:0099173	postsynapse organization	0.568431467	0.315123095	1
GO:0031346	positive regulation of cell projection organization	0.569691554	0.315724923	1
GO:0030833	regulation of actin filament polymerization	0.5705324	0.315997356	1
GO:0032200	telomere organization	0.5705324	0.315997356	1
GO:0140013	meiotic nuclear division	0.576774696	0.319356987	1
GO:0034470	ncRNA processing	0.577959764	0.319915259	1
GO:0019083	viral transcription	0.578835454	0.320301994	1
GO:0032970	regulation of actin filament-based process	0.584762341	0.323393454	1
GO:0048736	appendage development	0.584958369	0.323393454	1
GO:0060173	limb development	0.584958369	0.323393454	1
GO:0090288	negative regulation of cellular response to growth factor stimulus	0.586979712	0.324411833	1
GO:0007051	spindle organization	0.588991318	0.325324875	1
GO:0120032	regulation of plasma membrane bounded cell projection assembly	0.588991318	0.325324875	1
GO:0002695	negative regulation of leukocyte activation	0.590993234	0.326231453	1

GO:0007416	synapse assembly	0.590993234	0.326231453	1
GO:0060491	regulation of cell projection assembly	0.592985505	0.327231371	1
GO:0009260	ribonucleotide biosynthetic process	0.594968178	0.328225385	1
GO:0009100	glycoprotein metabolic process	0.598127954	0.329867965	1
GO:0008064	regulation of actin polymerization or depolymerization	0.6028038	0.332345403	1
GO:0030832	regulation of actin filament length	0.604739164	0.333310875	1
GO:0021953	central nervous system neuron differentiation	0.606665201	0.334168863	1
GO:0044272	sulfur compound biosynthetic process	0.606665201	0.334168863	1
GO:0030041	actin filament polymerization	0.608581955	0.335020697	1
GO:1903046	meiotic cell cycle process	0.608581955	0.335020697	1
GO:0046390	ribose phosphate biosynthetic process	0.610489471	0.335764326	1
GO:0046578	regulation of Ras protein signal transduction	0.610489471	0.335764326	1
GO:1902115	regulation of organelle assembly	0.610489471	0.335764326	1
GO:0007219	Notch signaling pathway	0.612387792	0.336501547	1
GO:0019080	viral gene expression	0.612387792	0.336501547	1
GO:0031497	chromatin assembly	0.612387792	0.336501547	1
GO:0000819	sister chromatid segregation	0.614276963	0.337437158	1
GO:0050657	nucleic acid transport	0.621743011	0.341331195	1
GO:0050658	RNA transport	0.621743011	0.341331195	1
GO:0007015	actin filament organization	0.622641422	0.341617122	1
GO:0046486	glycerolipid metabolic process	0.622641422	0.341617122	1
GO:1901605	alpha-amino acid metabolic process	0.625422257	0.343038835	1

GO:0051236	establishment of RNA localization	0.627248584	0.343936305	1
GO:0060348	bone development	0.629066105	0.344828404	1
GO:0006888	endoplasmic reticulum to Golgi vesicle-mediated transport	0.632674891	0.346596599	1
GO:0050852	T cell receptor signaling pathway	0.632674891	0.346596599	1
GO:0030100	regulation of endocytosis	0.639788612	0.350387608	1
GO:0051650	establishment of vesicle localization	0.643294204	0.352200888	1
GO:0006338	chromatin remodeling	0.653609792	0.35763221	1
GO:0071805	potassium ion transmembrane transport	0.653609792	0.35763221	1
GO:0009952	anterior/posterior pattern specification	0.655300134	0.358448715	1
GO:0006333	chromatin assembly or disassembly	0.656982318	0.359151727	1
GO:0017148	negative regulation of translation	0.656982318	0.359151727	1
GO:0008154	actin polymerization or depolymerization	0.658656383	0.359958137	1
GO:0043583	ear development	0.663630246	0.362566868	1
GO:0008037	cell recognition	0.666906263	0.364246705	1
GO:0006364	rRNA processing	0.668532419	0.365024689	1
GO:0048705	skeletal system morphogenesis	0.670150722	0.365797917	1
GO:0051648	vesicle localization	0.67176121	0.366566415	1
GO:0006457	protein folding	0.673363921	0.367219498	1
GO:0046474	glycerophospholipid biosynthetic process	0.673363921	0.367219498	1
GO:0051259	protein complex oligomerization	0.674958892	0.367978413	1
GO:0022618	ribonucleoprotein complex assembly	0.678125762	0.369593591	1
GO:0006403	RNA localization	0.682818935	0.372039414	1

GO:0016072	rRNA metabolic process	0.685910052	0.373498703	1
GO:0034249	negative regulation of cellular amide metabolic process	0.685910052	0.373498703	1
GO:0071826	ribonucleoprotein complex subunit organization	0.688971372	0.375052818	1
GO:0051321	meiotic cell cycle	0.709589826	0.386044533	1
GO:0090092	regulation of transmembrane receptor protein serine/threonine kinase signaling pathway	0.709589826	0.386044533	1
GO:0015931	nucleobase-containing compound transport	0.71938613	0.391256468	1
GO:0045017	glycerolipid biosynthetic process	0.731502487	0.397726713	1
GO:0008654	phospholipid biosynthetic process	0.732816387	0.398321408	1
GO:0098813	nuclear chromosome segregation	0.734123927	0.39891229	1
GO:0098742	cell-cell adhesion via plasma-membrane adhesion molecules	0.740567298	0.402292716	1
GO:0110053	regulation of actin filament organization	0.741837317	0.402861676	1
GO:0031503	protein-containing complex localization	0.757798559	0.411406106	1
GO:0048562	embryonic organ morphogenesis	0.77053899	0.418197368	1
GO:0042254	ribosome biogenesis	0.77938622	0.42287222	1
GO:0098656	anion transmembrane transport	0.784743525	0.425651306	1
GO:0043414	macromolecule methylation	0.787895935	0.427233132	1
GO:0051056	regulation of small GTPase mediated signal transduction	0.793048794	0.429898419	1
GO:0007059	chromosome segregation	0.803953303	0.435679049	1
GO:0000209	protein polyubiquitination	0.809659655	0.438508781	1
GO:0006520	cellular amino acid metabolic process	0.809659655	0.438508781	1
GO:0009101	glycoprotein biosynthetic process	0.811525022	0.439387544	1
GO:0032956	regulation of actin cytoskeleton organization	0.827520098	0.447913802	1

GO:0006790	sulfur compound metabolic process	0.836626003	0.452707158	1
GO:0048193	Golgi vesicle transport	0.839025809	0.453869991	1
GO:0006605	protein targeting	0.886105463	0.479194416	1
GO:0007608	sensory perception of smell	0.890530117	0.481443326	1
GO:0022613	ribonucleoprotein complex biogenesis	0.90565977	0.48947656	1

Table S2. Results of CC GO terms enrichment of Scutellaria baicalensis in breast cancer.

ID	Description	pvalue	qvalue	Count
GO:0045121	membrane raft	4.02216E-09	3.66649E-07	13
GO:0098857	membrane microdomain	4.17145E-09	3.66649E-07	13
GO:0098589	membrane region	6.62638E-09	3.88282E-07	13
GO:0042734	presynaptic membrane	4.64859E-08	1.83355E-06	10
GO:0099056	integral component of presynaptic membrane	5.21519E-08	1.83355E-06	9
GO:0098889	intrinsic component of presynaptic membrane	1.16317E-07	3.40789E-06	9
GO:0099055	integral component of postsynaptic membrane	1.22265E-06	3.07043E-05	8
GO:0098936	intrinsic component of postsynaptic membrane	1.62059E-06	3.56104E-05	8
GO:0005901	caveola	2.26278E-06	4.41969E-05	8
GO:0099699	integral component of synaptic membrane	7.61425E-06	0.000126047	8
GO:0031968	organelle outer membrane	8.04921E-06	0.000126047	8
GO:0019867	outer membrane	8.60442E-06	0.000126047	7
GO:0099240	intrinsic component of synaptic membrane	1.24327E-05	0.00015894	7
GO:0097060	synaptic membrane	1.26581E-05	0.00015894	7
GO:0044853	plasma membrane raft	1.45482E-05	0.000170495	7
GO:0005741	mitochondrial outer membrane	3.17801E-05	0.000349163	7
GO:0045211	postsynaptic membrane	4.85648E-05	0.000502187	7
GO:0005667	transcription regulator complex	0.000130061	0.001270191	7
GO:0098978	glutamatergic synapse	0.000279469	0.002585673	7
GO:0034399	nuclear periphery	0.000366184	0.003218569	7
GO:0042383	sarcolemma	0.00042043	0.003519391	6
GO:0060205	cytoplasmic vesicle lumen	0.00081931	0.006489825	6
GO:0031983	vesicle lumen	0.000849118	0.006489825	6
GO:0090575	RNA polymerase II transcription regulator complex	0.000934121	0.006842024	6
GO:0000307	cyclin-dependent protein kinase holoenzyme complex	0.001050845	0.007389098	5
GO:0098691	dopaminergic synapse	0.001615165	0.010561098	5
GO:0005635	nuclear envelope	0.001622109	0.010561098	5
GO:0030315	T-tubule	0.002035625	0.012780054	5
GO:0031965	nuclear membrane	0.002820252	0.017095536	5
GO:0031093	platelet alpha granule lumen	0.003764251	0.022057189	4
GO:0034774	secretory granule lumen	0.003925275	0.022258777	4
GO:0046930	pore complex	0.005082967	0.02792288	4
GO:0032809	neuronal cell body membrane	0.007480715	0.039849423	4
GO:0120111	neuron projection cytoplasm	0.007791766	0.0402856	4
GO:1902554	serine/threonine protein kinase complex	0.008292834	0.041651228	4
GO:0031091	platelet alpha granule	0.008812476	0.043031682	4
GO:0044298	cell body membrane	0.009119741	0.0433285	4
GO:0032839	dendrite cytoplasm	0.010903724	0.050441049	4
GO:1902911	protein kinase complex	0.012651981	0.057027824	4
GO:0016363	nuclear matrix	0.014345947	0.06304666	3
GO:0034703	cation channel complex	0.019752972	0.082446891	3

GO:0101002	ficolin-1-rich granule	0.020167398	0.082446891	3
GO:1904813	ficolin-1-rich granule lumen	0.020167398	0.082446891	3
GO:0099061	integral component of postsynaptic density membrane	0.023550587	0.094089666	3
GO:0099146	intrinsic component of postsynaptic density membrane	0.026196402	0.102334482	3
GO:1904724	tertiary granule lumen	0.027103969	0.103578097	3
GO:0061695	transferase complex, transferring phosphorus-containing groups	0.030473829	0.113978264	3
GO:0072562	blood microparticle	0.031811899	0.116504104	3
GO:0070820	tertiary granule	0.041156192	0.147649496	3
GO:0098981	cholinergic synapse	0.045574055	0.15703743	3
GO:0099060	integral component of postsynaptic specialization membrane	0.046591354	0.15703743	3
GO:0005775	vacuolar lumen	0.046955673	0.15703743	3
GO:0017053	transcription repressor complex	0.048871309	0.15703743	3
GO:0062023	collagen-containing extracellular matrix	0.049067084	0.15703743	3
GO:0005652	nuclear lamina	0.050016879	0.15703743	3
GO:0098948	intrinsic component of postsynaptic specialization membrane	0.050026295	0.15703743	3
GO:0034702	ion channel complex	0.050927576	0.157061962	3
GO:0030877	beta-catenin destruction complex	0.054439247	0.159497444	3
GO:1990454	L-type voltage-gated calcium channel complex	0.054439247	0.159497444	3
GO:1990909	Wnt signalosome	0.054439247	0.159497444	3
GO:0005788	endoplasmic reticulum lumen	0.05563261	0.160321757	3
GO:0098685	Schaffer collateral - CA1 synapse	0.058380686	0.161619892	3
GO:0033391	chromatoid body	0.058841254	0.161619892	2
GO:0044292	dendrite terminus	0.058841254	0.161619892	2
GO:0000940	condensed chromosome outer kinetochore	0.063222992	0.168875319	2
GO:1902495	transmembrane transporter complex	0.063404087	0.168875319	2
GO:0035578	azurophil granule lumen	0.067179564	0.17444094	2
GO:1990351	transporter complex	0.06808959	0.17444094	2
GO:0098839	postsynaptic density membrane	0.068470681	0.17444094	2
GO:0014069	postsynaptic density	0.072333495	0.176361972	2
GO:0032838	plasma membrane bounded cell projection cytoplasm	0.075532841	0.176361972	2
GO:0032279	asymmetric synapse	0.076076985	0.176361972	2
GO:0000778	condensed nuclear chromosome kinetochore	0.076247511	0.176361972	2
GO:0001518	voltage-gated sodium channel complex	0.076247511	0.176361972	2
GO:0043083	synaptic cleft	0.076247511	0.176361972	2
GO:0043194	axon initial segment	0.076247511	0.176361972	2
GO:0005911	cell-cell junction	0.080010581	0.181534389	2
GO:0097440	apical dendrite	0.080549091	0.181534389	2
GO:0000794	condensed nuclear chromosome	0.083193345	0.182806429	2
GO:0005604	basement membrane	0.083193345	0.182806429	2
GO:0000159	protein phosphatase type 2A complex	0.084830859	0.18410336	2
GO:0099572	postsynaptic specialization	0.08788261	0.18840046	2
GO:0071682	endocytic vesicle lumen	0.089092906	0.188693916	2
GO:0098984	neuron to neuron synapse	0.092701629	0.193027846	2

GO:0005697	telomerase holoenzyme complex	0.093335321	0.193027846	2
GO:0030687	preribosome, large subunit precursor	0.101761613	0.208007214	2
GO:0099634	postsynaptic specialization membrane	0.104638306	0.211428882	2
GO:0035327	transcriptionally active chromatin	0.105945668	0.211637877	2
GO:0034706	sodium channel complex	0.114256036	0.224644757	2
GO:0099568	cytoplasmic region	0.115012735	0.224644757	2
GO:0001891	phagocytic cup	0.118382524	0.226200018	2
GO:0034364	high-density lipoprotein particle	0.118382524	0.226200018	2
GO:0030018	Z disc	0.11967757	0.226215666	2
GO:0005640	nuclear outer membrane	0.122489999	0.227134243	2
GO:0043679	axon terminus	0.122747697	0.227134243	2
GO:0031674	I band	0.13837551	0.251417879	2
GO:0000780	condensed nuclear chromosome, centromeric region	0.138731482	0.251417879	2
GO:0005669	transcription factor TFIID complex	0.146740373	0.260559727	2
GO:0005719	nuclear euchromatin	0.146740373	0.260559727	1
GO:0044306	neuron projection terminus	0.152791731	0.266571986	1
GO:0034358	plasma lipoprotein particle	0.154675601	0.266571986	1
GO:1990777	lipoprotein particle	0.154675601	0.266571986	1
GO:0043198	dendritic shaft	0.162537836	0.272157877	1
GO:0005766	primary lysosome	0.162561366	0.272157877	1
GO:0042582	azurophil granule	0.162561366	0.272157877	1
GO:0043204	perikaryon	0.164200762	0.27230911	1
GO:0032994	protein-lipid complex	0.166441788	0.273445929	1
GO:0150034	distal axon	0.174229708	0.282940811	1
GO:0000781	chromosome, telomeric region	0.17741905	0.282940811	1
GO:0000791	euchromatin	0.178045975	0.282940811	1
GO:0000922	spindle pole	0.179083314	0.282940811	1
GO:0005891	voltage-gated calcium channel complex	0.181878419	0.282940811	1
GO:0032590	dendrite membrane	0.181878419	0.282940811	1
GO:0043209	myelin sheath	0.197032264	0.303826298	1
GO:0008287	protein serine/threonine phosphatase complex	0.211908446	0.313035917	1
GO:0009925	basal plasma membrane	0.211908446	0.313035917	1
GO:0014704	intercalated disc	0.211908446	0.313035917	1
GO:0098878	neurotransmitter receptor complex	0.211908446	0.313035917	1
GO:1903293	phosphatase complex	0.211908446	0.313035917	1
GO:0005782	peroxisomal matrix	0.215584698	0.31320265	1
GO:0031907	microbody lumen	0.215584698	0.31320265	1
GO:0098687	chromosomal region	0.222883761	0.318545385	1
GO:0032154	cleavage furrow	0.222886397	0.318545385	1
GO:1904115	axon cytoplasm	0.233713084	0.331325001	1
GO:0005819	spindle	0.243818993	0.337838653	1
GO:0005637	nuclear inner membrane	0.24439057	0.337838653	1
GO:0032993	protein-DNA complex	0.247101393	0.337838653	1

GO:0032589	neuron projection membrane	0.247916928	0.337838653	1
GO:0035861	site of double-strand break	0.247916928	0.337838653	1
GO:0030017	sarcomere	0.250546735	0.33879599	1
GO:0005769	early endosome	0.2562852	0.343910232	1
GO:0016328	lateral plasma membrane	0.26186033	0.348729467	1
GO:0034704	calcium channel complex	0.268735845	0.355194984	1
GO:0044304	main axon	0.272149812	0.356834412	1
GO:0045178	basal part of cell	0.275548015	0.356834412	1
GO:0000793	condensed chromosome	0.276416242	0.356834412	1
GO:0005657	replication fork	0.282297415	0.356834412	1
GO:0016529	sarcoplasmic reticulum	0.282297415	0.356834412	1
GO:0030016	myofibril	0.285037082	0.356834412	1
GO:0032153	cell division site	0.285648756	0.356834412	1
GO:0016607	nuclear speck	0.286540008	0.356834412	1
GO:0070821	tertiary granule membrane	0.28898462	0.356834412	1
GO:0044291	cell-cell contact zone	0.292305075	0.356834412	1
GO:0098982	GABA-ergic synapse	0.292305075	0.356834412	1
GO:0031594	neuromuscular junction	0.298900046	0.362368839	1
GO:0043292	contractile fiber	0.303966926	0.362784608	1
GO:0005925	focal adhesion	0.304336974	0.362784608	1
GO:0030684	preribosome	0.305434227	0.362784608	1
GO:0016591	RNA polymerase II, holoenzyme	0.308678695	0.363113733	1
GO:0005758	mitochondrial intermembrane space	0.311908174	0.363113733	1
GO:0016528	sarcoplasm	0.311908174	0.363113733	1
GO:0030055	cell-substrate junction	0.314534455	0.363361904	1
GO:0016323	basolateral plasma membrane	0.317683524	0.363361904	1
GO:0090734	site of DNA damage	0.318322435	0.363361904	1
GO:0008076	voltage-gated potassium channel complex	0.33721252	0.382441364	1
GO:0001750	photoreceptor outer segment	0.340310135	0.383480382	1
GO:0031970	organelle envelope lumen	0.343393432	0.384490131	1
GO:0035579	specific granule membrane	0.346462475	0.385471241	1
GO:1905368	peptidase complex	0.34951733	0.386424323	1
GO:0034705	potassium channel complex	0.361596161	0.394812415	1
GO:0043202	lysosomal lumen	0.361596161	0.394812415	1
GO:0070469	respirasome	0.376383166	0.399442072	1
GO:0005759	mitochondrial matrix	0.378229003	0.399442072	1
GO:0016605	PML body	0.379299659	0.399442072	1
GO:0005796	Golgi lumen	0.382202662	0.399442072	1
GO:0055029	nuclear DNA-directed RNA polymerase complex	0.382202662	0.399442072	1
GO:0000428	DNA-directed RNA polymerase complex	0.385092237	0.399442072	1
GO:0042470	melanosome	0.387968444	0.399442072	1
GO:0048770	pigment granule	0.387968444	0.399442072	1
GO:0000777	condensed chromosome kinetochore	0.390831344	0.399442072	1

GO:0030175	filopodium	0.390831344	0.399442072	1
GO:0030863	cortical cytoskeleton	0.390831344	0.399442072	1
GO:0030880	RNA polymerase complex	0.396517471	0.402910968	1
GO:0022626	cytosolic ribosome	0.402151099	0.406286954	1
GO:0098802	plasma membrane signaling receptor complex	0.419403855	0.420958119	1
GO:0097733	photoreceptor cell cilium	0.42146226	0.420958119	1
GO:0030665	clathrin-coated vesicle membrane	0.424170157	0.421269201	1
GO:0000779	condensed chromosome, centromeric region	0.434876959	0.427077049	1
GO:0005923	bicellular tight junction	0.434876959	0.427077049	1
GO:0070160	tight junction	0.453142153	0.442542336	1
GO:0005793	endoplasmic reticulum-Golgi intermediate compartment	0.45570335	0.442584818	1
GO:0000776	kinetochore	0.473302563	0.452182654	1
GO:0005777	peroxisome	0.473302563	0.452182654	1
GO:0042579	microbody	0.473302563	0.452182654	1
GO:0097731	9+0 non-motile cilium	0.478226712	0.454285067	1
GO:0045335	phagocytic vesicle	0.480671684	0.454285067	1
GO:0043296	apical junction complex	0.48793882	0.458687211	1
GO:0016324	apical plasma membrane	0.502858043	0.47019761	1
GO:0036064	ciliary basal body	0.516015148	0.479947255	1
GO:0005875	microtubule associated complex	0.525031252	0.485270246	1
GO:0042581	specific granule	0.527259199	0.485270246	1
GO:0005912	adherens junction	0.540411464	0.494185238	1
GO:0097730	non-motile cilium	0.542568045	0.494185238	1
GO:0031225	anchored component of membrane	0.551094734	0.499364192	1
GO:0031256	leading edge membrane	0.559464198	0.501775086	1
GO:0043197	dendritic spine	0.559464198	0.501775086	1
GO:0044309	neuron spine	0.563590868	0.502910366	1
GO:0030662	coated vesicle membrane	0.575742865	0.510350765	1
GO:0030426	growth cone	0.577735402	0.510350765	1
GO:0009897	external side of plasma membrane	0.580974744	0.510646222	1
GO:0055037	recycling endosome	0.589497738	0.514685795	1
GO:0030427	site of polarized growth	0.591426371	0.514685795	1
GO:0030136	clathrin-coated vesicle	0.597158665	0.515881161	1
GO:0000775	chromosome, centromeric region	0.60093593	0.515881161	1
GO:0045177	apical part of cell	0.601603929	0.515881161	1
GO:0031514	motile cilium	0.606536243	0.517585859	1
GO:0005882	intermediate filament	0.635117365	0.539357234	1
GO:0098858	actin-based cell projection	0.643620454	0.543950485	1
GO:0036464	cytoplasmic ribonucleoprotein granule	0.664822204	0.559180599	1
GO:0035770	ribonucleoprotein granule	0.680277935	0.569455715	1
GO:0045111	intermediate filament cytoskeleton	0.69213321	0.576633804	1
GO:0098798	mitochondrial protein complex	0.711842886	0.590257011	1
GO:0005840	ribosome	0.718575231	0.593042074	1

GO:0030135	coated vesicle	0.753499607	0.618959342	1
GO:0005938	cell cortex	0.761538462	0.62068781	1
GO:0030667	secretory granule membrane	0.762665501	0.62068781	1
GO:0030139	endocytic vesicle	0.77040861	0.624100111	1
GO:0031253	cell projection membrane	0.801802754	0.646552679	1
GO:0031252	cell leading edge	0.862584809	0.692389633	1
GO:0005874	microtubule	0.868982236	0.694354227	1
GO:0005743	mitochondrial inner membrane	0.900681688	0.716426968	1

Table S3. Results of MF GO terms enrichment of Scutellaria baicalensis in breast cancer.

ID	Description	pvalue	qvalue	Count
GO:0008227	G protein-coupled amine receptor activity	1.4282E-10	3.35252E-08	11
GO:0001223	transcription coactivator binding	5.45016E-09	3.81772E-07	11
GO:0004879	nuclear receptor activity	6.50553E-09	3.81772E-07	10
GO:0098531	ligand-activated transcription factor activity	6.50553E-09	3.81772E-07	10
GO:0099528	G protein-coupled neurotransmitter receptor activity	1.16114E-07	5.45124E-06	10
GO:0003707	steroid hormone receptor activity	1.62524E-07	6.34906E-06	9
GO:0001221	transcription cofactor binding	1.89333E-07	6.34906E-06	8
GO:0020037	heme binding	4.19123E-07	1.2023E-05	8
GO:0044389	ubiquitin-like protein ligase binding	4.6097E-07	1.2023E-05	8
GO:0046906	tetrapyrrole binding	7.14631E-07	1.6775E-05	8
GO:0061629	RNA polymerase II-specific DNA-binding transcription factor binding	8.19321E-07	1.74841E-05	8
GO:0140297	DNA-binding transcription factor binding	1.1571E-06	2.26344E-05	8
GO:0001091	RNA polymerase II general transcription initiation factor binding	2.07114E-06	3.59193E-05	7
GO:0031625	ubiquitin protein ligase binding	2.14227E-06	3.59193E-05	7
GO:0046982	protein heterodimerization activity	4.27855E-06	6.69556E-05	7
GO:0002020	protease binding	5.31956E-06	7.80436E-05	7
GO:0008144	drug binding	1.29264E-05	0.000178124	7
GO:0097199	cysteine-type endopeptidase activity involved in apoptotic signaling pathway	1.38015E-05	0.000178124	7
GO:0005496	steroid binding	1.44177E-05	0.000178124	7
GO:0051721	protein phosphatase 2A binding	1.82911E-05	0.000192984	7
GO:001	sodium:chloride symporter activity	1.8909E	0.00019	6

5378		-05	2984	
GO:0051400	BH domain binding	1.8909E-05	0.000192984	6
GO:0070513	death domain binding	1.8909E-05	0.000192984	6
GO:0015373	anion:sodium symporter activity	4.12666E-05	0.000387472	6
GO:1901338	catecholamine binding	4.12666E-05	0.000387472	6
GO:0017171	serine hydrolase activity	4.58734E-05	0.00041416	6
GO:0097153	cysteine-type endopeptidase activity involved in apoptotic process	5.13981E-05	0.000446853	6
GO:0051879	Hsp90 protein binding	5.48182E-05	0.000459566	6
GO:0005126	cytokine receptor binding	6.01205E-05	0.000486638	6
GO:0140296	general transcription initiation factor binding	6.59834E-05	0.000516291	6
GO:0015377	cation:chloride symporter activity	7.62645E-05	0.000559441	6
GO:0035173	histone kinase activity	7.62645E-05	0.000559441	6
GO:0032813	tumor necrosis factor receptor superfamily binding	9.31579E-05	0.000662654	6
GO:0005326	neurotransmitter transmembrane transporter activity	0.000107899	0.000733913	6
GO:0016538	cyclin-dependent protein serine/threonine kinase regulator activity	0.000109429	0.000733913	6
GO:0005123	death receptor binding	0.000126485	0.000824739	5
GO:0016705	oxidoreductase activity, acting on paired donors, with incorporation or reduction of molecular oxygen	0.000154792	0.000982039	5
GO:0015296	anion:cation symporter activity	0.000169643	0.0010012	5
GO:0015464	acetylcholine receptor activity	0.000169643	0.0010012	5
GO:0001085	RNA polymerase II transcription factor binding	0.000170608	0.0010012	5
GO:0047485	protein N-terminus binding	0.000191556	0.001088927	5
GO:0004252	serine-type endopeptidase activity	0.000194835	0.001088927	5

GO:0030594	neurotransmitter receptor activity	0.000227377	0.001241251	5
GO:0098960	postsynaptic neurotransmitter receptor activity	0.000303982	0.001621723	5
GO:0019887	protein kinase regulator activity	0.000317199	0.001643029	5
GO:0002039	p53 binding	0.000322375	0.001643029	4
GO:0004175	endopeptidase activity	0.000332633	0.001643029	4
GO:0008236	serine-type peptidase activity	0.000335974	0.001643029	4
GO:0031072	heat shock protein binding	0.000423871	0.002030577	4
GO:0001098	basal transcription machinery binding	0.000449878	0.002070644	4
GO:0001099	basal RNA polymerase II transcription machinery binding	0.000449878	0.002070644	4
GO:0005164	tumor necrosis factor receptor binding	0.000479429	0.002123389	4
GO:0030291	protein serine/threonine kinase inhibitor activity	0.000479429	0.002123389	4
GO:0004993	G protein-coupled serotonin receptor activity	0.00063142	0.002694865	4
GO:0099589	serotonin receptor activity	0.00063142	0.002694865	4
GO:0019207	kinase regulator activity	0.000719535	0.003016096	4
GO:0035257	nuclear hormone receptor binding	0.000750819	0.003092016	4
GO:0019903	protein phosphatase binding	0.000875542	0.003543482	4
GO:0008081	phosphoric diester hydrolase activity	0.001001481	0.003984482	4
GO:0001094	TFIID-class transcription factor complex binding	0.001066388	0.00403743	4
GO:0016725	oxidoreductase activity, acting on CH or CH2 groups	0.001066388	0.00403743	4
GO:0016803	ether hydrolase activity	0.001066388	0.00403743	4
GO:0051213	dioxygenase activity	0.001088032	0.004053988	4
GO:0005125	cytokine activity	0.001113529	0.004084161	4

GO:0016801	hydrolase activity, acting on ether bonds	0.001299162	0.004691709	4
GO:0001046	core promoter sequence-specific DNA binding	0.001534468	0.005286579	4
GO:0004861	cyclin-dependent protein serine/threonine kinase inhibitor activity	0.00155397	0.005286579	4
GO:0043176	amine binding	0.00155397	0.005286579	4
GO:0051378	serotonin binding	0.00155397	0.005286579	4
GO:0016922	nuclear receptor binding	0.001600829	0.005368195	3
GO:1901618	organic hydroxy compound transmembrane transporter activity	0.001735919	0.005729435	3
GO:0051087	chaperone binding	0.001782633	0.005729435	3
GO:0001162	RNA polymerase II intronic transcription regulatory region sequence-specific DNA binding	0.001830593	0.005729435	3
GO:0034236	protein kinase A catalytic subunit binding	0.001830593	0.005729435	3
GO:0042166	acetylcholine binding	0.001830593	0.005729435	3
GO:0051427	hormone receptor binding	0.001878782	0.005802885	3
GO:0050998	nitric-oxide synthase binding	0.002128814	0.006489755	3
GO:0004601	peroxidase activity	0.00218663	0.006580546	3
GO:0050661	NADP binding	0.002436724	0.007240366	3
GO:0042826	histone deacetylase binding	0.00257185	0.007461089	3
GO:0015267	channel activity	0.002600455	0.007461089	3
GO:0022803	passive transmembrane transporter activity	0.002633959	0.007461089	3
GO:0016684	oxidoreductase activity, acting on peroxide as acceptor	0.002703839	0.007461089	3
GO:0001161	intronic transcription regulatory region sequence-specific DNA binding	0.002789186	0.007461089	3
GO:0004697	protein kinase C activity	0.002789186	0.007461089	3
GO:0004698	calcium-dependent protein kinase C activity	0.002789186	0.007461089	3

GO:001<br>6641	oxidoreductase activity, acting on the CH-NH2 group of donors, oxygen as acceptor	0.00278<br>9186	0.00746<br>1089	3
GO:001<br>9902	phosphatase binding	0.00279<br>7072	0.00746<br>1089	3
GO:003<br>3218	amide binding	0.00285<br>7665	0.00753<br>707	3
GO:009<br>7110	scaffold protein binding	0.00329<br>0617	0.00858<br>2546	3
GO:000<br>4860	protein kinase inhibitor activity	0.00412<br>6195	0.01064<br>3625	3
GO:001<br>6638	oxidoreductase activity, acting on the CH-NH2 group of donors	0.00435<br>9691	0.01100<br>4088	3
GO:003<br>1690	adrenergic receptor binding	0.00435<br>9691	0.01100<br>4088	3
GO:004<br>2165	neurotransmitter binding	0.00480<br>3129	0.01199<br>4376	3
GO:001<br>9210	kinase inhibitor activity	0.00487<br>9444	0.01205<br>6688	3
GO:000<br>5518	collagen binding	0.00507<br>9838	0.01242<br>1096	3
GO:004<br>6873	metal ion transmembrane transporter activity	0.00538<br>6065	0.01303<br>4102	3
GO:001<br>5370	solute:sodium symporter activity	0.00549<br>5362	0.01316<br>2896	3
GO:000<br>5178	integrin binding	0.00573<br>3607	0.01323<br>164	3
GO:000<br>4114	3',5'-cyclic-nucleotide phosphodiesterase activity	0.00574<br>9533	0.01323<br>164	3
GO:000<br>9931	calcium-dependent protein serine/threonine kinase activity	0.00574<br>9533	0.01323<br>164	3
GO:007<br>0412	R-SMAD binding	0.00574<br>9533	0.01323<br>164	3
GO:000<br>0979	RNA polymerase II core promoter sequence-specific DNA binding	0.00625<br>2094	0.01411<br>1508	3
GO:001<br>0857	calcium-dependent protein kinase activity	0.00625<br>2094	0.01411<br>1508	3
GO:000<br>5261	cation channel activity	0.00634<br>2654	0.01417<br>9567	3
GO:000<br>5506	iron ion binding	0.00676<br>6927	0.01458<br>8053	3
GO:000<br>4112	cyclic-nucleotide phosphodiesterase activity	0.00677<br>3959	0.01458<br>8053	3
GO:001<br>6702	oxidoreductase activity, acting on single donors with incorporation of molecular oxygen, incorporation of two atoms of oxygen	0.00677<br>3959	0.01458<br>8053	3

GO:0070330	aromatase activity	0.006773959	0.014588053	3
GO:0015081	sodium ion transmembrane transporter activity	0.00692394	0.014775489	3
GO:0004435	phosphatidylinositol phospholipase C activity	0.007314928	0.01515176	3
GO:0016701	oxidoreductase activity, acting on single donors with incorporation of molecular oxygen	0.007314928	0.01515176	3
GO:0017025	TBP-class protein binding	0.007314928	0.01515176	3
GO:0033613	activating transcription factor binding	0.007358456	0.01515176	3
GO:0050660	flavin adenine dinucleotide binding	0.007614424	0.015542486	3
GO:0030295	protein kinase activator activity	0.007875609	0.015937031	3
GO:0004629	phospholipase C activity	0.00845339	0.016816289	3
GO:0035035	histone acetyltransferase binding	0.00845339	0.016816289	3
GO:0008013	beta-catenin binding	0.008690696	0.01714308	3
GO:0016209	antioxidant activity	0.008972977	0.017552403	3
GO:0051117	ATPase binding	0.009553547	0.018533631	3
GO:0019209	kinase activator activity	0.009851875	0.018955721	3
GO:0042578	phosphoric ester hydrolase activity	0.01010156	0.019278117	3
GO:0051059	NF-kappaB binding	0.010299463	0.019497285	3
GO:0048018	receptor ligand activity	0.010592597	0.019891782	3
GO:0030546	signaling receptor activator activity	0.011159932	0.02079085	3
GO:0051019	mitogen-activated protein kinase binding	0.011620189	0.021447154	3
GO:0015077	monovalent inorganic cation transmembrane transporter activity	0.01169495	0.021447154	2
GO:0015294	solute:cation symporter activity	0.012781317	0.023257721	2
GO:0016712	oxidoreductase activity, acting on paired donors, with incorporation or reduction of molecular oxygen, reduced flavin or flavoprotein as one donor, and incorporation of one atom of oxygen	0.013011159	0.023493833	2

GO:0097718	disordered domain specific binding	0.01373252	0.024607086	2
GO:0004497	monooxygenase activity	0.013856787	0.024641654	2
GO:0015108	chloride transmembrane transporter activity	0.014226388	0.025108703	2
GO:0008395	steroid hydroxylase activity	0.015226074	0.026474967	2
GO:0043539	protein serine/threonine kinase activator activity	0.015226074	0.026474967	2
GO:0005244	voltage-gated ion channel activity	0.01667837	0.028576845	2
GO:0022832	voltage-gated channel activity	0.01667837	0.028576845	2
GO:0005516	calmodulin binding	0.017530026	0.029818428	2
GO:0009055	electron transfer activity	0.017804729	0.030067811	2
GO:0030331	estrogen receptor binding	0.01841145	0.030870325	2
GO:0042277	peptide binding	0.018573441	0.030921069	2
GO:0004197	cysteine-type endopeptidase activity	0.019098862	0.031571877	2
GO:0005245	voltage-gated calcium channel activity	0.020100426	0.032995179	2
GO:0005216	ion channel activity	0.020264799	0.033033992	2
GO:0004712	protein serine/threonine/tyrosine kinase activity	0.020968375	0.0334597	2
GO:0016709	oxidoreductase activity, acting on paired donors, with incorporation or reduction of molecular oxygen, NAD(P)H as one donor, and incorporation of one atom of oxygen	0.020968375	0.0334597	2
GO:0048156	tau protein binding	0.020968375	0.0334597	2
GO:0004674	protein serine/threonine kinase activity	0.021096116	0.0334597	2
GO:0001102	RNA polymerase II activating transcription factor binding	0.022750334	0.035841219	2
GO:0008009	chemokine activity	0.024592577	0.038485226	2
GO:0070888	E-box binding	0.025535886	0.039435614	2
GO:0099529	neurotransmitter receptor activity involved in regulation of postsynaptic membrane potential	0.025535886	0.039435614	2

GO:0022836	gated channel activity	0.026154389	0.040126789	2
GO:0051018	protein kinase A binding	0.026493766	0.040383525	2
GO:0030374	nuclear receptor transcription coactivator activity	0.029453189	0.04460483	2
GO:0008234	cysteine-type peptidase activity	0.031865404	0.047948617	2
GO:0022843	voltage-gated cation channel activity	0.032449462	0.048036764	2
GO:0043621	protein self-association	0.032537907	0.048036764	2
GO:0050840	extracellular matrix binding	0.032537907	0.048036764	2
GO:0015293	symporter activity	0.034235214	0.050226663	2
GO:0019842	vitamin binding	0.034841632	0.050798848	2
GO:0015103	inorganic anion transmembrane transporter activity	0.035453618	0.051372039	2
GO:0008083	growth factor activity	0.046699463	0.067252051	2
GO:0042379	chemokine receptor binding	0.047250621	0.067373043	2
GO:0002162	dystroglycan binding	0.048505569	0.067373043	2
GO:0004955	prostaglandin receptor activity	0.048505569	0.067373043	2
GO:0008603	cAMP-dependent protein kinase regulator activity	0.048505569	0.067373043	2
GO:0018455	alcohol dehydrogenase [NAD(P)+] activity	0.048505569	0.067373043	2
GO:0043560	insulin receptor substrate binding	0.048505569	0.067373043	2
GO:0008201	heparin binding	0.051783854	0.071395157	2
GO:0070491	repressing transcription factor binding	0.05218385	0.071395157	2
GO:0004312	fatty acid synthase activity	0.05322621	0.071395157	2
GO:0004954	prostanoid receptor activity	0.05322621	0.071395157	2
GO:0005527	macrolide binding	0.05322621	0.071395157	2

GO:0031996	thioesterase binding	0.05322621	0.071395157	2
GO:0001540	amyloid-beta binding	0.05599928	0.0731012	2
GO:0001664	G protein-coupled receptor binding	0.057916639	0.0731012	2
GO:0003964	RNA-directed DNA polymerase activity	0.057923687	0.0731012	2
GO:0004115	3',5'-cyclic-AMP phosphodiesterase activity	0.057923687	0.0731012	2
GO:0016004	phospholipase activator activity	0.057923687	0.0731012	2
GO:0031543	peptidyl-proline dioxygenase activity	0.057923687	0.0731012	2
GO:0034452	dynactin binding	0.057923687	0.0731012	2
GO:0042301	phosphate ion binding	0.057923687	0.0731012	2
GO:0043023	ribosomal large subunit binding	0.057923687	0.0731012	2
GO:0047555	3',5'-cyclic-GMP phosphodiesterase activity	0.057923687	0.0731012	2
GO:1902282	voltage-gated potassium channel activity involved in ventricular cardiac muscle cell action potential repolarization	0.057923687	0.0731012	2
GO:0046332	SMAD binding	0.058595863	0.073554053	2
GO:0035258	steroid hormone receptor binding	0.061233572	0.076456252	2
GO:0008179	adenylate cyclase binding	0.062598111	0.076531682	2
GO:0016175	superoxide-generating NAD(P)H oxidase activity	0.062598111	0.076531682	2
GO:0016653	oxidoreductase activity, acting on NAD(P)H, heme protein as acceptor	0.062598111	0.076531682	2
GO:0031005	filamin binding	0.062598111	0.076531682	2
GO:0005172	vascular endothelial growth factor receptor binding	0.067249595	0.0805406	2
GO:0032794	GTPase activating protein binding	0.067249595	0.0805406	2
GO:0035497	cAMP response element binding	0.067249595	0.0805406	2
GO:0060229	lipase activator activity	0.067249595	0.0805406	2

GO:0008237	metallopeptidase activity	0.06770907	0.080679256	2
GO:0004953	icosanoid receptor activity	0.071878251	0.083527097	2
GO:0005161	platelet-derived growth factor receptor binding	0.071878251	0.083527097	2
GO:0030553	cGMP binding	0.071878251	0.083527097	2
GO:0042809	vitamin D receptor binding	0.071878251	0.083527097	2
GO:0086008	voltage-gated potassium channel activity involved in cardiac muscle cell action potential repolarization	0.071878251	0.083527097	2
GO:0005159	insulin-like growth factor receptor binding	0.076484189	0.086732642	2
GO:0008392	arachidonic acid epoxygenase activity	0.076484189	0.086732642	2
GO:0010181	FMN binding	0.076484189	0.086732642	2
GO:0070402	NADPH binding	0.076484189	0.086732642	2
GO:0071837	HMG box domain binding	0.076484189	0.086732642	2
GO:0004708	MAP kinase kinase activity	0.085628351	0.095261273	2
GO:0045236	CXCR chemokine receptor binding	0.085628351	0.095261273	2
GO:0046965	retinoid X receptor binding	0.085628351	0.095261273	2
GO:0070064	proline-rich region binding	0.085628351	0.095261273	2
GO:0031406	carboxylic acid binding	0.088430236	0.097914313	2
GO:0016722	oxidoreductase activity, oxidizing metal ions	0.090166794	0.098904059	1
GO:0050664	oxidoreductase activity, acting on NAD(P)H, oxygen as acceptor	0.090166794	0.098904059	1
GO:0001972	retinoic acid binding	0.094682957	0.099666271	1
GO:0004707	MAP kinase activity	0.094682957	0.099666271	1
GO:0004745	retinol dehydrogenase activity	0.094682957	0.099666271	1
GO:0008320	protein transmembrane transporter activity	0.094682957	0.099666271	1

GO:0008391	arachidonic acid monooxygenase activity	0.094682957	0.099666271	1
GO:0030506	ankyrin binding	0.094682957	0.099666271	1
GO:0031418	L-ascorbic acid binding	0.094682957	0.099666271	1
GO:0031681	G-protein beta-subunit binding	0.094682957	0.099666271	1
GO:0140318	protein transporter activity	0.094682957	0.099666271	1
GO:0004620	phospholipase activity	0.095728593	0.099871233	1
GO:0016407	acetyltransferase activity	0.095728593	0.099871233	1
GO:0031490	chromatin DNA binding	0.097266438	0.101026622	1
GO:0043177	organic acid binding	0.100182299	0.102877514	1
GO:0004222	metalloendopeptidase activity	0.100363243	0.102877514	1
GO:0099094	ligand-gated cation channel activity	0.100363243	0.102877514	1
GO:0016651	oxidoreductase activity, acting on NAD(P)H	0.101921991	0.103975256	1
GO:0005242	inward rectifier potassium channel activity	0.103648876	0.103975256	1
GO:0022884	macromolecule transmembrane transporter activity	0.103648876	0.103975256	1
GO:0050321	tau-protein kinase activity	0.103648876	0.103975256	1
GO:0070034	telomerase RNA binding	0.103648876	0.103975256	1
GO:0022853	active ion transmembrane transporter activity	0.105256483	0.105138615	1
GO:0005158	insulin receptor binding	0.108098846	0.105973127	1
GO:0005248	voltage-gated sodium channel activity	0.108098846	0.105973127	1
GO:0017134	fibroblast growth factor binding	0.108098846	0.105973127	1
GO:0030552	cAMP binding	0.108098846	0.105973127	1
GO:0005539	glycosaminoglycan binding	0.108349206	0.105973127	1

GO:0015101	organic cation transmembrane transporter activity	0.112526966	0.108701255	1
GO:0070006	metalloaminopeptidase activity	0.112526966	0.108701255	1
GO:0015291	secondary active transmembrane transporter activity	0.112527734	0.108701255	1
GO:0005262	calcium channel activity	0.116242595	0.111579525	1
GO:0001671	ATPase activator activity	0.116933341	0.111579525	1
GO:0004190	aspartic-type endopeptidase activity	0.116933341	0.111579525	1
GO:0016616	oxidoreductase activity, acting on the CH-OH group of donors, NAD or NADP as acceptor	0.119491899	0.112117411	1
GO:0001965	G-protein alpha-subunit binding	0.121318078	0.112117411	1
GO:0008198	ferrous iron binding	0.121318078	0.112117411	1
GO:0015252	proton channel activity	0.121318078	0.112117411	1
GO:0016628	oxidoreductase activity, acting on the CH-CH group of donors, NAD or NADP as acceptor	0.121318078	0.112117411	1
GO:0017075	syntaxin-1 binding	0.121318078	0.112117411	1
GO:0042923	neuropeptide binding	0.121318078	0.112117411	1
GO:0070001	aspartic-type peptidase activity	0.121318078	0.112117411	1
GO:0001968	fibronectin binding	0.12568128	0.115242293	1
GO:0042974	retinoic acid receptor binding	0.12568128	0.115242293	1
GO:0043236	laminin binding	0.134343503	0.122705718	1
GO:0016298	lipase activity	0.136063462	0.123317017	1
GO:0016614	oxidoreductase activity, acting on CH-OH group of donors	0.136063462	0.123317017	1
GO:0030332	cyclin binding	0.13864273	0.124691788	1
GO:0043325	phosphatidylinositol-3,4-bisphosphate binding	0.13864273	0.124691788	1
GO:1901681	sulfur compound binding	0.141112039	0.126428223	1

GO:0015085	calcium ion transmembrane transporter activity	0.142830226	0.127078736	1
GO:1904680	peptide transmembrane transporter activity	0.142920839	0.127078736	1
GO:0004713	protein tyrosine kinase activity	0.144533157	0.128027385	1
GO:0019838	growth factor binding	0.146240415	0.128431535	1
GO:0016417	S-acyltransferase activity	0.147177932	0.128431535	1
GO:0048019	receptor antagonist activity	0.147177932	0.128431535	1
GO:0071889	14-3-3 protein binding	0.147177932	0.128431535	1
GO:0015276	ligand-gated ion channel activity	0.151387316	0.130670847	1
GO:0022834	ligand-gated channel activity	0.151387316	0.130670847	1
GO:0005251	delayed rectifier potassium channel activity	0.151414112	0.130670847	1
GO:0070851	growth factor receptor binding	0.154838707	0.13313681	1
GO:0052689	carboxylic ester hydrolase activity	0.158305445	0.135620877	1
GO:0005501	retinoid binding	0.163998182	0.138476314	1
GO:0034061	DNA polymerase activity	0.163998182	0.138476314	1
GO:0042805	actinin binding	0.163998182	0.138476314	1
GO:0043394	proteoglycan binding	0.163998182	0.138476314	1
GO:0016247	channel regulator activity	0.167035322	0.139474923	1
GO:0019840	isoprenoid binding	0.168151718	0.139474923	1
GO:0030551	cyclic nucleotide binding	0.168151718	0.139474923	1
GO:0042056	chemoattractant activity	0.168151718	0.139474923	1
GO:0048487	beta-tubulin binding	0.168151718	0.139474923	1
GO:0032451	demethylase activity	0.172284843	0.141900351	1

GO:0043014	alpha-tubulin binding	0.172284843	0.141900351	1
GO:0005547	phosphatidylinositol-3,4,5-trisphosphate binding	0.176397657	0.144275363	1
GO:0016248	channel inhibitor activity	0.176397657	0.144275363	1
GO:0016504	peptidase activator activity	0.180490259	0.146601085	1
GO:0016790	thiolester hydrolase activity	0.180490259	0.146601085	1
GO:0030547	receptor inhibitor activity	0.184562746	0.148878613	1
GO:0060590	ATPase regulator activity	0.184562746	0.148878613	1
GO:0030544	Hsp70 protein binding	0.188615217	0.151626508	1
GO:0072509	divalent inorganic cation transmembrane transporter activity	0.191888526	0.153731422	1
GO:0005272	sodium channel activity	0.196660497	0.157018585	1
GO:0008188	neuropeptide receptor activity	0.2006535	0.159663624	1
GO:0004177	aminopeptidase activity	0.204626873	0.161186127	1
GO:0004715	non-membrane spanning protein tyrosine kinase activity	0.204626873	0.161186127	1
GO:0016706	2-oxoglutarate-dependent dioxygenase activity	0.204626873	0.161186127	1
GO:0048020	CCR chemokine receptor binding	0.20858071	0.163751095	1
GO:0015485	cholesterol binding	0.216430161	0.169347108	1
GO:0003743	translation initiation factor activity	0.224202607	0.174266927	1
GO:0042887	amide transmembrane transporter activity	0.224202607	0.174266927	1
GO:0045296	cadherin binding	0.227560059	0.176292837	1
GO:0015459	potassium channel regulator activity	0.231898794	0.179063127	1
GO:0004402	histone acetyltransferase activity	0.239519463	0.182545592	1
GO:0005080	protein kinase C binding	0.239519463	0.182545592	1

GO:0030170	pyridoxal phosphate binding	0.239519463	0.182545592	1
GO:0070279	vitamin B6 binding	0.239519463	0.182545592	1
GO:0008022	protein C-terminus binding	0.240952947	0.183043799	1
GO:0022804	active transmembrane transporter activity	0.243340179	0.184260984	1
GO:0043022	ribosome binding	0.247065347	0.185882498	1
GO:0061733	peptide-lysine-N-acetyltransferase activity	0.247065347	0.185882498	1
GO:1990841	promoter-specific chromatin binding	0.250810471	0.18809731	1
GO:0016597	amino acid binding	0.254537171	0.188483444	1
GO:0016627	oxidoreductase activity, acting on the CH-CH group of donors	0.254537171	0.188483444	1
GO:0016836	hydro-lyase activity	0.254537171	0.188483444	1
GO:0032934	sterol binding	0.254537171	0.188483444	1
GO:0005507	copper ion binding	0.258245536	0.189436693	1
GO:0022824	transmitter-gated ion channel activity	0.258245536	0.189436693	1
GO:0022835	transmitter-gated channel activity	0.258245536	0.189436693	1
GO:0008509	anion transmembrane transporter activity	0.260634683	0.190593652	1
GO:0004714	transmembrane receptor protein tyrosine kinase activity	0.261935655	0.190950151	1
GO:0008235	metalloexopeptidase activity	0.272897423	0.198325323	1
GO:0034212	peptide N-acetyltransferase activity	0.276515442	0.200334449	1
GO:0048029	monosaccharide binding	0.290810321	0.210042758	1
GO:0004857	enzyme inhibitor activity	0.295664851	0.212893968	1
GO:0030971	receptor tyrosine kinase binding	0.301347827	0.215662918	1
GO:0033293	monocarboxylic acid binding	0.301347827	0.215662918	1

GO:000 5230	extracellular ligand-gated ion channel activity	0.30828 6649	0.21929 1619	1
GO:001 6835	carbon-oxygen lyase activity	0.30828 6649	0.21929 1619	1
GO:000 4722	protein serine/threonine phosphatase activity	0.31173 0456	0.22107 1368	1
GO:001 6747	transferase activity, transferring acyl groups other than amino-acyl groups	0.31437 1103	0.22227 253	1
GO:001 9905	syntaxin binding	0.32196 046	0.22695 49	1
GO:001 9199	transmembrane receptor protein kinase activity	0.32869 6769	0.23100 9705	1
GO:000 8080	N-acetyltransferase activity	0.34197 1438	0.23819 969	1
GO:000 8135	translation factor activity, RNA binding	0.34197 1438	0.23819 969	1
GO:004 2562	hormone binding	0.34197 1438	0.23819 969	1
GO:003 0165	PDZ domain binding	0.34524 9334	0.23977 1415	1
GO:004 3178	alcohol binding	0.34851 108	0.24132 2685	1
GO:000 5249	voltage-gated potassium channel activity	0.35498 6436	0.24508 3514	1
GO:007 2341	modified amino acid binding	0.36139 8132	0.24877 8464	1
GO:001 6746	transferase activity, transferring acyl groups	0.36855 1653	0.25296 0968	1
GO:000 8238	exopeptidase activity	0.37715 2905	0.25810 986	1
GO:000 3713	transcription coactivator activity	0.38274 2805	0.26117 3946	1
GO:199 0782	protein tyrosine kinase binding	0.38642 0602	0.26291 9281	1
GO:001 6791	phosphatase activity	0.40029 2853	0.27157 0752	1
GO:001 6410	N-acyltransferase activity	0.41341 3172	0.27966 3696	1
GO:009 0079	translation regulator activity, nucleic acid binding	0.41925 0139	0.28279 7281	1
GO:000 0149	SNARE binding	0.43641 8573	0.29269 5765	1
GO:000 5088	Ras guanyl-nucleotide exchange factor activity	0.43641 8573	0.29269 5765	1

GO:0003730	mRNA 3'-UTR binding	0.442029074	0.295613986	1
GO:0099106	ion channel regulator activity	0.44758433	0.297633236	1
GO:1902936	phosphatidylinositol bisphosphate binding	0.44758433	0.297633236	1
GO:0003712	transcription coregulator activity	0.450258379	0.297779494	1
GO:0005267	potassium channel activity	0.450341409	0.297779494	1
GO:0005179	hormone activity	0.455814804	0.300552044	1
GO:0015078	proton transmembrane transporter activity	0.469263583	0.308553086	1
GO:0017124	SH3 domain binding	0.479785482	0.314590304	1
GO:0044325	ion channel binding	0.482383557	0.315346383	1
GO:0016779	nucleotidyltransferase activity	0.484968798	0.315346383	1
GO:0043021	ribonucleoprotein complex binding	0.484968798	0.315346383	1
GO:0005319	lipid transporter activity	0.487541268	0.316143364	1
GO:0001227	DNA-binding transcription repressor activity, RNA polymerase II-specific	0.497016593	0.32139974	1
GO:0001217	DNA-binding transcription repressor activity	0.49859874	0.321537071	1
GO:0045182	translation regulator activity	0.500214231	0.321695093	1
GO:0060589	nucleoside-triphosphatase regulator activity	0.517339176	0.331799356	1
GO:0008528	G protein-coupled peptide receptor activity	0.522249314	0.334035844	1
GO:0001653	peptide receptor activity	0.531731611	0.339176628	1
GO:0015079	potassium ion transmembrane transporter activity	0.541027732	0.344171115	1
GO:0015631	tubulin binding	0.54309595	0.344553049	1
GO:0005201	extracellular matrix structural constituent	0.569997583	0.360645371	1
GO:0004721	phosphoprotein phosphatase activity	0.591044652	0.371956984	1

GO:190 1981	phosphatidylinositol phosphate binding	0.59104 4652	0.37195 6984	1
GO:001 6829	lyase activity	0.61107 2313	0.38353 258	1
GO:000 3714	transcription corepressor activity	0.62071 9223	0.38854 8454	1
GO:001 6758	transferase activity, transferring hexosyl groups	0.63382 8507	0.39569 9208	1
GO:000 3779	actin binding	0.64148 9924	0.39924 9438	1
GO:005 1015	actin filament binding	0.64291 6921	0.39924 9438	1
GO:014 0097	catalytic activity, acting on DNA	0.64826 2504	0.40150 6842	1
GO:000 5085	guanyl-nucleotide exchange factor activity	0.65871 7356	0.40690 8505	1
GO:000 5543	phospholipid binding	0.66217 7938	0.40797 2593	1
GO:006 1134	peptidase regulator activity	0.68193 183	0.41904 3257	1
GO:003 5091	phosphatidylinositol binding	0.73065 3118	0.44780 9936	1
GO:000 8017	microtubule binding	0.73469 7698	0.44911 6192	1
GO:003 0246	carbohydrate binding	0.73736 0654	0.44957 3276	1
GO:000 5096	GTPase activator activity	0.74775 0676	0.45355 2021	1
GO:001 6757	transferase activity, transferring glycosyl groups	0.74775 0676	0.45355 2021	1
GO:003 0695	GTPase regulator activity	0.78539 8356	0.47515 9613	1
GO:001 9001	guanyl nucleotide binding	0.86400 186	0.52003 3508	1
GO:003 2561	guanyl ribonucleotide binding	0.86400 186	0.52003 3508	1
GO:001 7016	Ras GTPase binding	0.87589 3789	0.52584 2819	1
GO:003 1267	small GTPase binding	0.88383 6971	0.52925 7907	1

Table S4. Results of KEGG terms enrichment of Scutellaria baicalensis in breast cancer.

ID	Description	pvalue	qvalue	Count
hsa05417	Lipid and atherosclerosis	4.61314E-19	3.88475E-17	24
hsa05161	Hepatitis B	8.59501E-17	2.51817E-15	22
hsa04933	AGE-RAGE signaling pathway in diabetic complications	8.97098E-17	2.51817E-15	20
hsa05163	Human cytomegalovirus infection	3.09276E-16	6.51108E-15	20
hsa05167	Kaposi sarcoma-associated herpesvirus infection	3.05986E-15	5.15345E-14	18
hsa04657	IL-17 signaling pathway	1.83733E-14	2.57871E-13	18
hsa05222	Small cell lung cancer	2.92941E-13	3.5241E-12	17
hsa05162	Measles	6.74265E-12	7.09752E-11	17
hsa05205	Proteoglycans in cancer	1.68193E-11	1.57373E-10	17
hsa05170	Human immunodeficiency virus 1 infection	2.88299E-11	2.20707E-10	17
hsa05207	Chemical carcinogenesis - receptor activation	2.88299E-11	2.20707E-10	15
hsa04932	Non-alcoholic fatty liver disease	3.30637E-11	2.32026E-10	15
hsa05210	Colorectal cancer	4.80082E-11	3.10984E-10	15
hsa04210	Apoptosis	6.93656E-11	4.17237E-10	15
hsa04115	p53 signaling pathway	1.38225E-10	7.76001E-10	15
hsa01522	Endocrine resistance	2.31256E-10	1.21714E-09	14
hsa05142	Chagas disease	3.72245E-10	1.84394E-09	14
hsa05160	Hepatitis C	4.80952E-10	2.25007E-09	14
hsa04668	TNF signaling pathway	1.12154E-09	4.9708E-09	14
hsa05169	Epstein-Barr virus infection	1.42444E-09	5.89244E-09	14
hsa05164	Influenza A	1.4934E-09	5.89244E-09	13
hsa04936	Alcoholic liver disease	1.5394E-09	5.89244E-09	13
hsa01524	Platinum drug resistance	2.65084E-09	9.70558E-09	13
hsa05215	Prostate cancer	3.17274E-09	1.11324E-08	13
hsa05135	Yersinia infection	1.15047E-08	3.83807E-08	13
hsa04151	PI3K-Akt signaling pathway	1.185E-08	3.83807E-08	13
hsa04915	Estrogen signaling pathway	1.24977E-08	3.89791E-08	12
hsa05418	Fluid shear stress and atherosclerosis	1.3567E-08	4.08032E-08	12
hsa05224	Breast cancer	2.55584E-08	7.42168E-08	12
hsa04919	Thyroid hormone signaling pathway	3.34334E-08	9.38483E-08	12
hsa05133	Pertussis	6.43187E-08	1.7472E-07	12
hsa05130	Pathogenic Escherichia coli infection	8.15295E-08	2.14551E-07	12
hsa04625	C-type lectin receptor signaling pathway	8.62348E-08	2.20057E-07	12
hsa05134	Legionellosis	9.35542E-08	2.31713E-07	12
hsa04066	HIF-1 signaling pathway	1.35165E-07	3.25208E-07	12
hsa05219	Bladder cancer	1.53837E-07	3.59852E-07	12
hsa05145	Toxoplasmosis	1.75034E-07	3.98369E-07	11
hsa04010	MAPK signaling pathway	2.24107E-07	4.85612E-07	11
hsa04726	Serotonergic synapse	2.24899E-07	4.85612E-07	11
hsa05152	Tuberculosis	2.4158E-07	5.08588E-07	11
hsa04722	Neurotrophin signaling pathway	3.10584E-07	6.37913E-07	11

hsa05166	Human T-cell leukemia virus 1 infection	3.29132E-07	6.59913E-07	11
hsa04218	Cellular senescence	4.56566E-07	8.94131E-07	11
hsa04917	Prolactin signaling pathway	4.80073E-07	9.18801E-07	11
hsa05223	Non-small cell lung cancer	5.98694E-07	1.12036E-06	10
hsa04926	Relaxin signaling pathway	6.60946E-07	1.20997E-06	10
hsa04215	Apoptosis - multiple species	6.98678E-07	1.25183E-06	10
hsa05212	Pancreatic cancer	9.12531E-07	1.60093E-06	10
hsa04620	Toll-like receptor signaling pathway	9.84121E-07	1.69129E-06	10
hsa05022	Pathways of neurodegeneration - multiple diseases	1.04326E-06	1.75707E-06	10
hsa05132	Salmonella infection	1.22078E-06	1.99461E-06	10
hsa01521	EGFR tyrosine kinase inhibitor resistance	1.23167E-06	1.99461E-06	10
hsa04659	Th17 cell differentiation	1.35545E-06	2.15365E-06	10
hsa05213	Endometrial cancer	1.78891E-06	2.78972E-06	10
hsa04370	VEGF signaling pathway	2.0131E-06	3.08225E-06	10
hsa04261	Adrenergic signaling in cardiomyocytes	2.64281E-06	3.97415E-06	10
hsa05323	Rheumatoid arthritis	4.28311E-06	6.32778E-06	9
hsa05165	Human papillomavirus infection	5.5165E-06	7.99153E-06	9
hsa04380	Osteoclast differentiation	5.59907E-06	7.99153E-06	9
hsa05131	Shigellosis	6.8843E-06	9.66217E-06	9
hsa05146	Amoebiasis	8.56232E-06	1.18203E-05	9
hsa04660	T cell receptor signaling pathway	9.89228E-06	1.3436E-05	9
hsa05140	Leishmaniasis	1.22387E-05	1.63591E-05	9
hsa04024	cAMP signaling pathway	1.37223E-05	1.80557E-05	9
hsa04621	NOD-like receptor signaling pathway	1.63037E-05	2.11222E-05	8
hsa04725	Cholinergic synapse	1.82455E-05	2.32797E-05	8
hsa04923	Regulation of lipolysis in adipocytes	2.05854E-05	2.58733E-05	8
hsa05202	Transcriptional misregulation in cancer	2.46873E-05	3.05725E-05	8
hsa04071	Sphingolipid signaling pathway	2.66112E-05	3.24774E-05	8
hsa04020	Calcium signaling pathway	2.96542E-05	3.56743E-05	8
hsa05235	PD-L1 expression and PD-1 checkpoint pathway in cancer	3.1774E-05	3.7686E-05	8
hsa05216	Thyroid cancer	3.34167E-05	3.90838E-05	8
hsa04728	Dopaminergic synapse	5.61263E-05	6.47455E-05	8
hsa05031	Amphetamine addiction	6.83731E-05	7.67698E-05	8
hsa05211	Renal cell carcinoma	6.83731E-05	7.67698E-05	8
hsa05208	Chemical carcinogenesis - reactive oxygen species	8.43683E-05	9.34828E-05	8
hsa05214	Glioma	0.000109378	0.000118768	8
hsa04931	Insulin resistance	0.000110009	0.000118768	8
hsa05171	Coronavirus disease - COVID-19	0.00011726	0.000123991	8
hsa05220	Chronic myeloid leukemia	0.000117791	0.000123991	8
hsa05010	Alzheimer disease	0.000125842	0.00013083	7
hsa05226	Gastric cancer	0.00013184	0.000134731	7
hsa05030	Cocaine addiction	0.000132794	0.000134731	7
hsa04921	Oxytocin signaling pathway	0.000165786	0.000166202	7

hsa04662	B cell receptor signaling pathway	0.000179687	0.000178018	7
hsa04510	Focal adhesion	0.000197598	0.000193486	7
hsa05203	Viral carcinogenesis	0.000220845	0.000213764	7
hsa01523	Antifolate resistance	0.000227715	0.000217909	7
hsa04022	cGMP-PKG signaling pathway	0.000288997	0.000273445	7
hsa05206	MicroRNAs in cancer	0.000292599	0.000273777	7
hsa05225	Hepatocellular carcinoma	0.000300955	0.000278501	6
hsa05416	Viral myocarditis	0.000348324	0.000318832	6
hsa04970	Salivary secretion	0.000357723	0.000323914	6
hsa04068	FoxO signaling pathway	0.000364607	0.000326636	6
hsa00590	Arachidonic acid metabolism	0.000376407	0.000333657	6
hsa05321	Inflammatory bowel disease	0.000506186	0.000444022	6
hsa05143	African trypanosomiasis	0.000519737	0.000451209	6
hsa04064	NF-kappa B signaling pathway	0.000651315	0.00055967	6
hsa04622	RIG-I-like receptor signaling pathway	0.000712702	0.00060017	6
hsa05120	Epithelial cell signaling in Helicobacter pylori infection	0.000712702	0.00060017	6
hsa00982	Drug metabolism - cytochrome P450	0.000810878	0.000669455	6
hsa05218	Melanoma	0.000810878	0.000669455	6
hsa04080	Neuroactive ligand-receptor interaction	0.000874286	0.000714797	6
hsa05168	Herpes simplex virus 1 infection	0.001429049	0.001157124	6
hsa04310	Wnt signaling pathway	0.001539529	0.00123471	5
hsa05144	Malaria	0.001638647	0.001301805	5
hsa04012	ErbB signaling pathway	0.00171407	0.001348998	5
hsa04110	Cell cycle	0.001769035	0.001379364	5
hsa05020	Prion disease	0.001798459	0.001389442	5
hsa04658	Th1 and Th2 cell differentiation	0.002430703	0.001860825	5
hsa05231	Choline metabolism in cancer	0.003200496	0.002428067	5
hsa05217	Basal cell carcinoma	0.003833453	0.002882295	5
hsa04928	Parathyroid hormone synthesis, secretion and action	0.004482388	0.003340392	5
hsa05415	Diabetic cardiomyopathy	0.004603171	0.003400311	5
hsa05221	Acute myeloid leukemia	0.00478303	0.003502448	5
hsa04664	Fc epsilon RI signaling pathway	0.005042988	0.003660971	5
hsa04920	Adipocytokine signaling pathway	0.005312274	0.003823499	5
hsa04217	Necroptosis	0.005613054	0.003998083	5
hsa05012	Parkinson disease	0.005649791	0.003998083	5
hsa00350	Tyrosine metabolism	0.005776591	0.004053748	5
hsa04630	JAK-STAT signaling pathway	0.006140253	0.004264068	5
hsa04137	Mitophagy - animal	0.006177569	0.004264068	5
hsa04935	Growth hormone synthesis, secretion and action	0.007288291	0.004989844	4
hsa00380	Tryptophan metabolism	0.008889092	0.005988441	4
hsa05332	Graft-versus-host disease	0.008889092	0.005988441	4
hsa04014	Ras signaling pathway	0.009378244	0.006267832	4
hsa04650	Natural killer cell mediated cytotoxicity	0.010802383	0.00716279	4

hsa00360	Phenylalanine metabolism	0.011301709	0.007435335	4
hsa05014	Amyotrophic lateral sclerosis	0.011683758	0.007627096	4
hsa04211	Longevity regulating pathway	0.0128685	0.00833587	4
hsa04062	Chemokine signaling pathway	0.013569946	0.008723147	4
hsa05032	Morphine addiction	0.013872307	0.008849957	4
hsa04912	GnRH signaling pathway	0.01492469	0.009449744	4
hsa00330	Arginine and proline metabolism	0.015101139	0.009490111	4
hsa04750	Inflammatory mediator regulation of TRP channels	0.017772846	0.011086376	4
hsa04061	Viral protein interaction with cytokine and cytokine receptor	0.01900071	0.011765145	4
hsa04340	Hedgehog signaling pathway	0.019383861	0.011914782	4
hsa04914	Progesterone-mediated oocyte maturation	0.0202801	0.012286316	4
hsa04972	Pancreatic secretion	0.0202801	0.012286316	4
hsa00340	Histidine metabolism	0.020911778	0.012578513	4
hsa04929	GnRH secretion	0.027494153	0.016420547	4
hsa00830	Retinol metabolism	0.032129778	0.019053982	4
hsa04924	Renin secretion	0.033348755	0.019502196	4
hsa05204	Chemical carcinogenesis - DNA adducts	0.033348755	0.019502196	4
hsa04152	AMPK signaling pathway	0.034188768	0.019855546	4
hsa05230	Central carbon metabolism in cancer	0.034591638	0.019951918	4
hsa00591	Linoleic acid metabolism	0.035104237	0.020109839	3
hsa04613	Neutrophil extracellular trap formation	0.044727514	0.025310717	3
hsa04114	Oocyte meiosis	0.044869847	0.025310717	3
hsa00980	Metabolism of xenobiotics by cytochrome P450	0.045385279	0.025310717	3
hsa04721	Synaptic vesicle cycle	0.045385279	0.025310717	3
hsa04270	Vascular smooth muscle contraction	0.048073579	0.026633562	3
hsa04910	Insulin signaling pathway	0.051401678	0.028291257	3
hsa04960	Aldosterone-regulated sodium reabsorption	0.054657485	0.029887893	3
hsa04140	Autophagy - animal	0.056031875	0.030441766	3
hsa05330	Allograft rejection	0.05732035	0.030942159	3
hsa00260	Glycine, serine and threonine metabolism	0.062778544	0.033672702	3
hsa04015	Rap1 signaling pathway	0.063480294	0.033833601	3
hsa05410	Hypertrophic cardiomyopathy	0.064315953	0.034063398	3
hsa04940	Type I diabetes mellitus	0.071279805	0.037515687	3
hsa04934	Cushing syndrome	0.073947977	0.038678249	3
hsa05414	Dilated cardiomyopathy	0.074947168	0.038916487	3
hsa04150	mTOR signaling pathway	0.075327725	0.038916487	3
hsa04666	Fc gamma R-mediated phagocytosis	0.076790863	0.039430482	3
hsa04930	Type II diabetes mellitus	0.080131306	0.04089636	3
hsa05016	Huntington disease	0.092545419	0.046947581	3
hsa04961	Endocrine and other factor-regulated calcium reabsorption	0.101989153	0.051428505	3
hsa04670	Leukocyte transendothelial migration	0.111037866	0.055658078	3
hsa05034	Alcoholism	0.124278678	0.061926467	2
hsa04611	Platelet activation	0.13348481	0.066122507	2

hsa04623	Cytosolic DNA-sensing pathway	0.135603311	0.066779101	2
hsa00061	Fatty acid biosynthesis	0.169100639	0.082644468	2
hsa04371	Apelin signaling pathway	0.169782728	0.082644468	2
hsa03320	PPAR signaling pathway	0.178593433	0.086083161	2
hsa04550	Signaling pathways regulating pluripotency of stem cells	0.179913806	0.086083161	2
hsa05017	Spinocerebellar ataxia	0.179913806	0.086083161	2
hsa04971	Gastric acid secretion	0.182270379	0.086387152	2
hsa04810	Regulation of actin cytoskeleton	0.183583265	0.086387152	2
hsa04060	Cytokine-cytokine receptor interaction	0.18362669	0.086387152	2
hsa04072	Phospholipase D signaling pathway	0.192805833	0.089703208	2
hsa04723	Retrograde endocannabinoid signaling	0.192805833	0.089703208	2
hsa00220	Arginine biosynthesis	0.202655714	0.093767826	2
hsa04610	Complement and coagulation cascades	0.215808788	0.099308042	2
hsa04742	Taste transduction	0.219574432	0.099948532	2
hsa04911	Insulin secretion	0.219574432	0.099948532	2
hsa04540	Gap junction	0.227122808	0.102828662	2
hsa04713	Circadian entrainment	0.261276811	0.117659133	2
hsa04640	Hematopoietic cell lineage	0.268886457	0.120441862	2
hsa05310	Asthma	0.273337689	0.121787887	2
hsa04916	Melanogenesis	0.276496053	0.122546727	2
hsa04974	Protein digestion and absorption	0.284102485	0.125258742	2
hsa04922	Glucagon signaling pathway	0.299294019	0.131269307	2
hsa00500	Starch and sucrose metabolism	0.309892276	0.135213428	2
hsa04216	Ferroptosis	0.344629041	0.149594809	2
hsa00071	Fatty acid degradation	0.35803492	0.154616969	1
hsa00230	Purine metabolism	0.377829162	0.162332615	1
hsa00620	Pyruvate metabolism	0.384039132	0.163334028	1
hsa04973	Carbohydrate digestion and absorption	0.384039132	0.163334028	1
hsa04672	Intestinal immune network for IgA production	0.396648184	0.167849007	1
hsa05110	Vibrio cholerae infection	0.402856738	0.16962389	1
hsa04913	Ovarian steroidogenesis	0.409002168	0.171354666	1
hsa05320	Autoimmune thyroid disease	0.421106183	0.175552343	1
hsa01212	Fatty acid metabolism	0.444584197	0.184426942	1
hsa04730	Long-term depression	0.461572993	0.190535807	1
hsa00140	Steroid hormone biosynthesis	0.46712103	0.191885404	1
hsa04213	Longevity regulating pathway - multiple species	0.472612582	0.193198807	1
hsa04390	Hippo signaling pathway	0.479827553	0.195200632	1
hsa00010	Glycolysis / Gluconeogenesis	0.499242736	0.201155472	1
hsa04720	Long-term potentiation	0.499242736	0.201155472	1
hsa04530	Tight junction	0.519044427	0.208138116	1
hsa04141	Protein processing in endoplasmic reticulum	0.52539125	0.20968471	1
hsa04918	Thyroid hormone synthesis	0.539113484	0.214146369	1
hsa05100	Bacterial invasion of epithelial cells	0.548580768	0.215870445	1

hsa05412	Arrhythmogenic right ventricular cardiomyopathy	0.548580768	0.215870445	1
hsa04612	Antigen processing and presentation	0.553242101	0.216692133	1
hsa04360	Axon guidance	0.559298765	0.218050201	1
hsa00983	Drug metabolism - other enzymes	0.562422571	0.218257607	1
hsa04146	Peroxisome	0.571416617	0.220730707	1
hsa04260	Cardiac muscle contraction	0.593110493	0.228064597	1
hsa04512	ECM-receptor interaction	0.597317248	0.228157811	1
hsa04727	GABAergic synapse	0.601481028	0.228157811	1
hsa04976	Bile secretion	0.601481028	0.228157811	1
hsa04350	TGF-beta signaling pathway	0.621670349	0.234758687	1
hsa04070	Phosphatidylinositol signaling system	0.633295351	0.237388494	1
hsa00564	Glycerophospholipid metabolism	0.637091371	0.237388494	1
hsa04925	Aldosterone synthesis and secretion	0.637091371	0.237388494	1
hsa03008	Ribosome biogenesis in eukaryotes	0.676369431	0.25091377	1
hsa04724	Glutamatergic synapse	0.692804957	0.255883641	1
hsa00190	Oxidative phosphorylation	0.750695304	0.276054352	1
hsa05322	Systemic lupus erythematosus	0.75585405	0.276742902	1
hsa04145	Phagosome	0.793500231	0.28926871	1
hsa04714	Thermogenesis	0.911069204	0.330696626	1
